# Cheminformatics-Guided Exploration of Synthetic Marine Natural Product-Inspired Brominated Indole-3-Glyoxylamides and Their Potentials for Drug Discovery

**DOI:** 10.3390/molecules29153648

**Published:** 2024-08-01

**Authors:** Darren C. Holland, Dale W. Prebble, Mark J. Calcott, Wayne A. Schroder, Francesca Ferretti, Aaron Lock, Vicky M. Avery, Milton J. Kiefel, Anthony R. Carroll

**Affiliations:** 1 School of Environment and Science, Griffith University, Southport, QLD 4222, Australia; dalepre3@gmail.com (D.W.P.); francesca.ferrettirodriguez@griffithuni.edu.au (F.F.); m.kiefel@griffith.edu.au (M.J.K.); 2 Griffith Institute for Drug Discovery, Griffith University, Nathan, QLD 4111, Australia; 3 School of Biological Sciences, Victoria University of Wellington, Wellington 6102, New Zealand; mark.calcott@vuw.ac.nz; 4 School of Environment and Science, Griffith University, Nathan, QLD 4111, Australia; w.schroder@griffith.edu.au (W.A.S.); a.lock@griffith.edu.au (A.L.); v.avery@griffith.edu.au (V.M.A.); 5 Discovery Biology, Griffith University, Nathan, QLD 4111, Australia; 6 Institute for Glycomics, Griffith University, Southport, QLD 4221, Australia

**Keywords:** indole synthesis, indol-3-yl-glyoxylamides, marine indole alkaloids, marine natural products, cheminformatics, Parkinson’s disease, malaria, SARS-CoV-2, protease

## Abstract

Marine natural products (MNPs) continue to be tested primarily in cellular toxicity assays, both mammalian and microbial, despite most being inactive at concentrations relevant to drug discovery. These MNPs become missed opportunities and represent a wasteful use of precious bioresources. The use of cheminformatics aligned with published bioactivity data can provide insights to direct the choice of bioassays for the evaluation of new MNPs. Cheminformatics analysis of MNPs found in MarinLit (*n* = 39,730) up to the end of 2023 highlighted indol-3-yl-glyoxylamides (IGAs, *n* = 24) as a group of MNPs with no reported bioactivities. However, a recent review of synthetic IGAs highlighted these scaffolds as privileged structures with several compounds under clinical evaluation. Herein, we report the synthesis of a library of 32 MNP-inspired brominated IGAs (**25**–**56**) using a simple one-pot, multistep method affording access to these diverse chemical scaffolds. Directed by a meta-analysis of the biological activities reported for marine indole alkaloids (MIAs) and synthetic IGAs, the brominated IGAs **25**–**56** were examined for their potential bioactivities against the Parkinson’s Disease amyloid protein alpha synuclein (α-syn), antiplasmodial activities against chloroquine-resistant (3D7) and sensitive (Dd2) parasite strains of *Plasmodium falciparum*, and inhibition of mammalian (chymotrypsin and elastase) and viral (SARS-CoV-2 3CL^pro^) proteases. All of the synthetic IGAs tested exhibited binding affinity to the amyloid protein α-syn, while some showed inhibitory activities against *P. falciparum*, and the proteases, SARS-CoV-2 3CL^pro^, and chymotrypsin. The cellular safety of the IGAs was examined against cancerous and non-cancerous human cell lines, with all of the compounds tested inactive, thereby validating cheminformatics and meta-analyses results. The findings presented herein expand our knowledge of marine IGA bioactive chemical space and advocate expanding the scope of biological assays routinely used to investigate NP bioactivities, specifically those more suitable for non-toxic compounds. By integrating cheminformatics tools and functional assays into NP biological testing workflows, we can aim to enhance the potential of NPs and their scaffolds for future drug discovery and development.

## 1. Introduction

Due to their enzymatic biosynthesis and evolutionary encoded interactions with the proteins and receptors of living systems, NPs are a valuable source of potential therapeutics that continue to be leveraged for drug discovery. Currently, MNPs have provided the source and/or inspiration for 15 approved therapeutics worldwide, mainly for the treatment of cancers, with dozens more positioned in various phases of clinical trials [1]. Despite this, the proportion of NP chemical space that is biologically relevant—that is, chemical space exclusive to bioactive NPs—remains vastly underexplored [2]. Recently, we published two meta-analyses that outline limiting factors contributing to this apparent lack of understanding of biologically relevant NP chemical space [3]. Our first meta-analysis examined the biological activities reported for new MNPs between 2016 and 2020 and revealed that 68% were neither cytotoxic, antimicrobial, nor anti-inflammatory and that most MNPs were not active in the screens they were tested in [3]. These findings were supported by a subsequent meta-analysis of MIA diversity and bioactivity, with the majority of MIAs examined for cytotoxicity and antimicrobial activities despite around 90% of compounds not being active at concentrations relevant to drug discovery in these assays [3]. Both meta-analyses clearly showed that MNPs continue to be examined against a narrow range of disease and protein targets, particularly when compared to synthetic drug and drug-leads, and that a lack of coherent strategies aimed at bridging the gap between MNP chemical and biologically relevant chemical space continue to limit our understanding of their biological potentials [3].

A common limitation for expanding NP biologically relevant chemical space (aside from the challenges associated with procuring academic funding, access to core facilities, and the availability of expertise required for investigating new biological targets) is access to sufficient quantities of compounds for random biological screening against diverse targets. Reasons that limit the supply of NPs include low isolation yield, access to producing organism/s, complexities and costs associated with re-isolation (inactivation of microbial biosynthetic gene clusters, for example), and unsustainable bioprospecting of NP-producing plants and animals. The structural complexity of some NPs makes them challenging to synthesise, but if they are amenable to synthesis, this helps to alleviate compound availability and promotes diversified biological evaluations [4]. However, without good reason, synthesising a complex natural product on the off chance that it may show potent activity in an untested assay is an extremely risky and expensive endeavour. Predictive computational approaches, such as cheminformatics-based ones, have become important tools for analysing the information available in what are increasingly large databases, thereby promoting more directed research efforts [3,5,6,7]. Several curated NP databases exist, such as MarinLit [8], SuperNatural 3.0 [9], and COCONUT [10], each of which houses extensive collections of chemical and biological data associated with NPs, can be effectively analysed using cheminformatics and other computational tools. Orthogonal approaches that combine cheminformatics-based analyses of large NP databases with the synthesis of NP scaffolds provide excellent platforms aimed at expanding biological testing opportunities and what is known about the biologically relevant chemical space of these important natural resources. In fact, we have recently shown that cheminformatics tools integrated with NP bioactivity information successfully directed the antibacterial evaluation of a series of marine-inspired bis-indole alkaloids [11].

**Figure 1:**
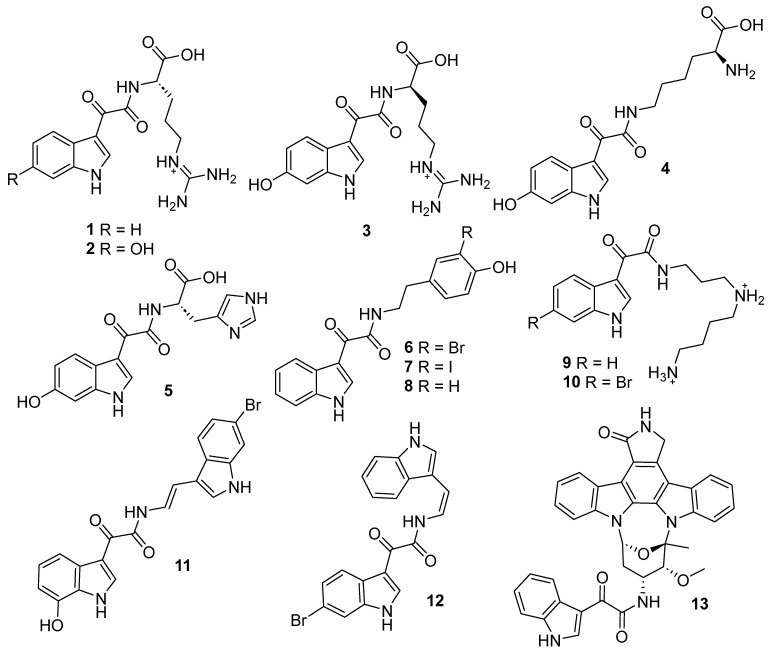
A snapshot of marine indol-3-yl-glyoxylamide structural diversity; leptoclinidamines A and B (**1** and **2**), (−)-leptoclinidamine B (**3**), herdmanine I and K (**4** and **5**), polyandrocarpamides A-C (**6**–**8**), didemindines A and B (**9** and **10**), coscinamide C (**11**), (Z)-coscinamide D (**12**), and staurosporine analogue 13G-291 (**13**).

Despite reduced funding from pharmaceutical and industry sectors, the reporting of MNPs continues to grow annually (MarinLit database contains 40,543 MNPs as of August 2023) [8], driven primarily by a strong global academic network [3]. MIAs represent a diverse sub-class of MNPs ubiquitous to most marine phyla, with 2048 reported in the MarinLit database to the end of 2023 [8]. Moreover, the indole motif is considered a privileged structure in drug discovery due to its potential for biological activity with several indole drugs approved for the treatment of disease and infection targets [3,12]. During our recent meta-analysis of MIA diversity and bioactivity [3], we identified an interesting group of marine indol-3-yl-glyoxylamides (IGAs) with vastly underexplored bioactivities (**1**–**24**; Figure 1 and Figure S1; Table S1). In contrast, several synthetic IGAs have been reported for a range of biological activities, including neuroprotective, antimicrobial, antiprotozoal, antiviral, antiprion, and enzymatic inhibition [13]. Some of these IGAs have also found clinical use, supporting their classification as a privileged structure for drug discovery [13].

Guided by cheminformatics analyses of MNP IGA chemical diversity and their lack of reported bioactivities, we embarked upon the synthesis of marine-inspired IGAs in an effort to expand our understanding of their biologically relevant chemical space. Herein, we report a simple one-pot, multi-step synthesis of 5 or 6-brominated IGAs (**25**–**56**) incorporating a broad range of proteinogenic D and L amino acids. Guided by their structural similarity to other synthetic IGAs the 32 synthetic indoles were evaluated against a panel of mostly functional biological disease and infection targets, including binding to the Parkinson’s disease amyloid protein α-synuclein (α-syn), inhibition of mammalian serine proteases (elastase and chymotrypsin), inhibition of SARS-CoV-2 3CL protease, and antiplasmodial activities. In addition, their effect on the cell viability of human embryonic kidney and three cancerous cell lines (breast, ovarian, and colon) were also examined.

## 2. Results

### 2.1. Cheminformatics Analyses of Marine Natural Product Indol-3-yl-Glyoxylamides

**Figure 2:**
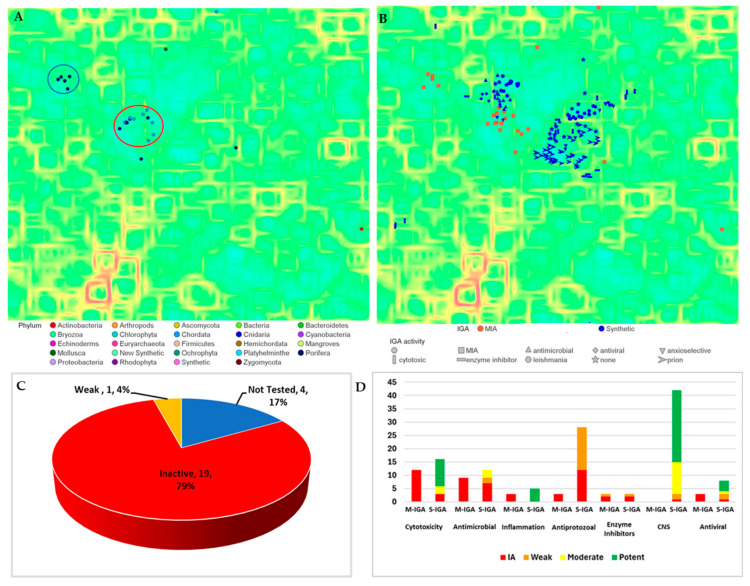
(**A**) Chemical diversity of MIAs (*n* = 2048) and synthetic IGAs (*n* = 147) filtered for marine IGAs only (*n* = 24) and scaled to producing marine phyla visualised in a 50 × 50 self-organising map (SOM) with the SkelSpheres chemical fingerprint descriptor (red circle = amino acid substituted IGAs, blue circle = IGA enamines). (**B**) SOM of chemical diversity of marine IGAs (*n* = 24) and bioactive synthetic IGAs (*n* = 147) scaled to bioactivity. (**C**) The potency of bioactivity reported for marine IGAs **1**–**24**. (**D**) Diversity and potency of bioactivities reported for marine IGAs **1**–**24** (M-IGA) compared with synthetic IGAs (*n* = 147, S-IGA).

Self-organising maps (SOM) provide excellent tools for visualising chemical diversity with structurally related molecules positioned within shared regions or clusters of two-dimensional space based on molecular fragment similarity scoring using neural network algorithms. Using the freely available DataWarrior (version 6.01.05) software [14], a self-organising map (SOM) of MIAs (*n* = 2048) reported to the end of 2021 was generated from the MarinLit database (Figures S2 and S3) [8]. The SOM’s complex background topography (Figure S2) was populated with MIAs (coloured according to producing marine phyla) arranged in-space according to structural similarity, which clearly emphasised the chemical diversity present within MIAs (Figure S3). Structural filtering of the MIA SOM for those containing the IGA motif identified 24 compounds (**1**–**24**, Figure 2A), almost all of which reported from marine invertebrates (Chordata *n* = 11, Porifera *n* = 11, and Molluscs *n* = 1) with the exception of IGA 13G-291 (**13**), a staurosporine-substituted IGA isolated from a *Streptomyces* species (Figure S3) [15].

The chemical diversity of marine IGAs resided mainly within two clusters (red and blue circles, Figure 2A). The two clusters contained two types of IGAs: the first group (red circle, *n* = 14) represented IGA motifs substituted with amino acids (carboxylated or decarboxylated) and amines (red, Figure S1), while the second (blue circle, *n* = 5) contained IGA enamine NPs (blue, Figure S1). Singleton IGAs (*n* = 5) included herdmanine K (**5**), located with other histidine-containing MIAs, 13G-291 (**13**), with the large staurosporine class of compounds, preoxazinin-7 (**16**), with other mollusc-derived oxazinins, hyrtimomine F (**17**), positioned with other structurally related cyclic indoles, and pyrinodermin F (**21**), situated near other long chain alkyne substituted indole amides.

A meta-analysis of the biological activities reported for the 24 IGAs found that 20 had been tested for bioactivity with an average of 1.4 disease or infection targets examined per compound, a testing rate lower than the overall testing rate for MIAs (*n* = 1.9) [3]. The biological activities reported for **1**–**24** were then categorised according to their reported potencies relevant to drug discovery previously described (inactive, weak, moderate, and potent, Table S2) [3]. This showed that 19 of the 20 IGAs examined for bioactivity were inactive, with only one, staurosporine analogue **24**; a structure class broadly recognised for anticancer activity, categorised with weak activity (Figure 2C) [15]. An overview of the biological targets and the potencies of marine versus synthetic IGAs clearly outlined the narrow breadth of disease and infection targets explored and the lack of meaningful bioactivities for marine IGAs. The major testing targets for marine IGAs examined cellular toxicities. Despite the focus on cytotoxicity (*n* = 12) and antimicrobial activities (*n* = 8), all the marine IGAs tested were inactive in these assays (Figure 2D). Moreover, there was a clear lack of non-toxic disease and infection targets examined for marine IGA bioactivities compared to synthetic ones. Synthetic IGAs displayed good potency toward targets not thoroughly examined by marine IGAs, including CNS, protozoan, anti-inflammatory, and viral ones [13]. However, while a small number of marine IGAs were screened in assays other than cytotoxic and antibacterial ones, including antiplasmodial and antiviral assays, all were found to be inactive [16,17,18]. Other marine IGA targets included protein kinase C, where staurosporine IGA 13G-291 (**13**) displayed only weak inhibition [15], and PPAR-γ agonist antidiabetic activity, in which the herdmanines I and K (**5** and **6**) were reported with unquantifiable potencies [19]. Interestingly, synthetic IGAs have shown excellent activities against CNS targets, specifically antiprion [13]. This bioactivity and others, including antileishmanial and antiviral activities, led us to examine the chemical similarity of marine IGAs compared with bioactive synthetic IGAs (Figure 2B). The resultant SOM showed that marine IGAs shared good overlap with synthetic IGAs, particularly those underexamined for marine IGA bioactivity, namely CNS and antiprotozoal bioactivities.

The aforementioned meta- and cheminformatics analyses directed our attention toward the synthesis of leptoclinidamine A–C (**1**–**3**) inspired IGA scaffolds. The IGA scaffolds **1**–**3** overlapped favourably with bioactive synthetic IGAs and also contained structures amenable to synthesis. By synthesising a library of 5- or 6-bromoindole containing IGAs coupled with a broad range of proteinogenic D and L amino acids, we could further explore their potential bioactive chemical space.

### 2.2. Synthesis of Indol-3-yl-Glyoxylamides ***25***–***56***

**Figure 3:**
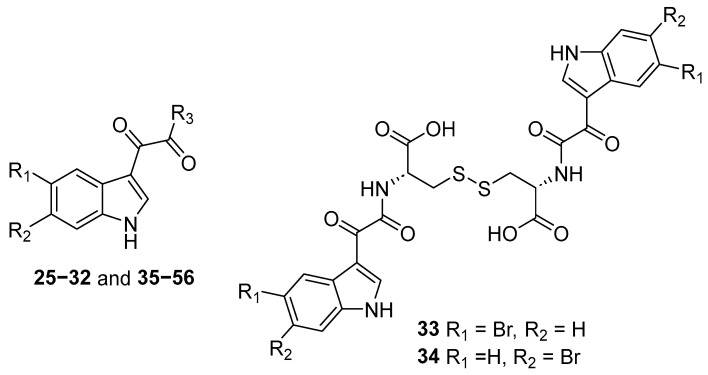
General structure for synthetic indol-3-yl-glyoxlyamides **25**–**56**.

**Scheme 1:**
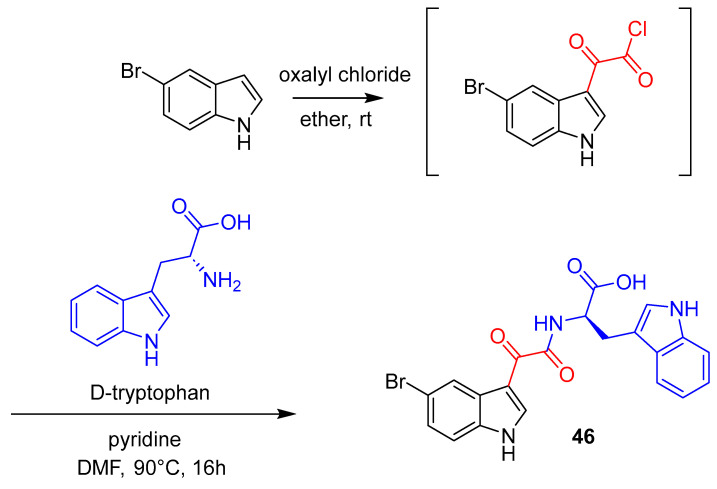
One-pot, two-step synthesis of brominated indol-3-yl-glyoxylamide analogues (**25**–**56**).

The general synthetic procedure undertaken for the preparation of the IGA analogues reported herein (**25**–**56**, Figure 3, Figures S4 and S5, and Table 1) was based on an NMR tube reaction we reported previously for the synthesis of (−)-leptoclinidamine A [16]. Our scaled-up synthetic strategy was modified to incorporate a one-pot multi-step method beginning with diacylation of the appropriate brominated indole with oxalyl chloride under argon at room temperature for 45 min (Scheme 1). The resultant reaction mixture was then carefully heated to 50 °C to remove ether, after which DMF and anhydrous pyridine were added sequentially. The temperature of the stirring reaction mixture was increased to between 80 and 90 °C before the desired D or L amino acid was added after 15 min. The resultant reaction mixture was left heating overnight, then quenched with H_2_O (2.0 mL) and repeatedly partitioned between EtOAc and H_2_O with the organic phase dried over anhydrous sodium sulphate and concentrated in vacuo. For all reactions, except those containing amino acids with basic side chains, the pH of the mixtures was monitored and acidified with 1 M HCl (pH 3–5) to promote migration of compounds into partitioned organic phases.

Following this, the crude reaction mixtures were adsorbed onto C_18_-bonded silica gel, loaded into a refillable guard column, and purified using preparative reversed-phase high-pressure liquid chromatography (RP HPLC) with a decreasing polarity solvent gradient from 100% H_2_O (0.1% TFA) to 100% MeOH (0.1% TFA) over 60 min with fractions collected each min. Analogues requiring further purification were resuspended in MeOH (100 μL), then injected onto a semi-preparative RP HPLC column eluting with an optimised solvent gradient from 40% MeOH/60% H_2_O (0.1% TFA) to 75% MeOH/25% H_2_O (0.1% TFA) over 40 min at a flow rate of 4.0 mL per min with fractions collected each min or by hand. It must be noted that other synthetic methodologies report the synthesis of indole-3-glyoxyl compounds using THF [20,21]. However, due to the poor solubility of polar amino acids in THF, the resultant amide coupling reactions were low yielding with considerable amounts of unreacted amino acid starting material present after purification. It was found that DMF with the addition of heat (~90 °C) provided good solubilisation of all amino acids, particularly the more polar ones.

### 2.3. Exploration of Biological Chemical Space of Brominated Indol-3-yl-Glyoxylamides *(**25***–***56**)*

**Figure 4:**
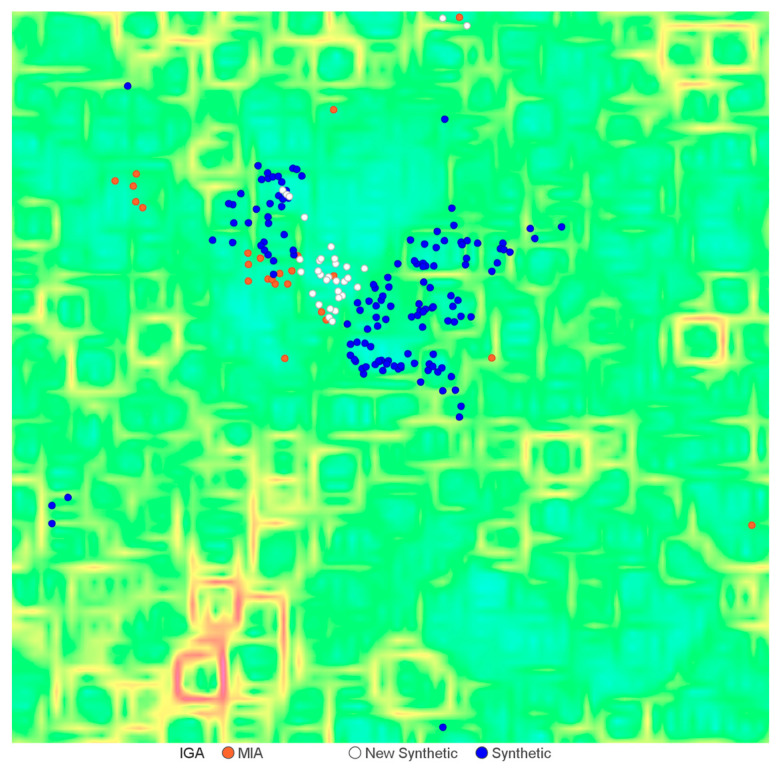
SOM of chemical diversity marine IGAs (**1**–**24**, orange), synthetic IGAs (blue, *n* = 147), and new synthetic IGAs **25**–**56** (white).

The chemical diversity of the brominated synthetic IGAs **25**–**56** (*n* = 32) was incorporated into the dataset of MIAs (*n* = 2048) and synthetic IGAs (*n* = 147) and visualised using an updated SOM (*n* = 2080, 50 × 50 neuron, SkelSpheres chemical descriptor, Figure 4). Encouragingly, the brominated leptoclinidamine-inspired IGAs **25**–**56** occupied chemical space that both overlapped and expanded the chemical diversity already present within the marine and synthetic IGA dataset. In addition, the synthetic IGAs **25**–**56** also showed occupied chemical space corresponding with bioactive synthetic IGAs, including those with CNS (antiprion) and antiprotozoal activities [13]. These findings directed our attention toward diversifying the testing of **25**–**56** against a range of biological targets, including CNS, antiprotozoal, viral and mammalian proteases, and cancer cell cytotoxicity.

#### 2.3.1. Parkinson’s Disease—Amyloid Protein Binding (α-syn)

The misfolding and aggregation of the synaptic protein α-synuclein (syn) into amyloid fibrils termed amyloidosis, is suspected as causal in the debilitating neurodegenerative disorder Parkinson’s disease (PD) [22,23].

**Figure 5:**
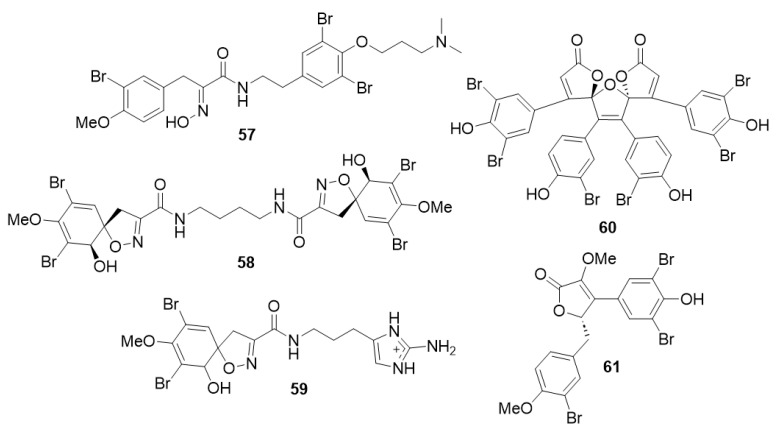
Brominated MNPs with amyloid protein activities; aplysamine-2 (**57**), aerothionin (**58**), aerophobin-2 (**59**), prunolide B (**60**), and procerolide C (**61**).

The α-syn protein aggregation cascade, initialised by the misfolding and fibrilisation of the protein and aggregation of amyloid fibrils, has been hypothesised to lead to cellular damage and dysfunction, ultimately resulting in neuronal cell death [24]. Therefore, inhibition of the progression of the α-syn protein aggregation cascade, postulated to disrupt the development of neuro-toxic forms of the amyloid protein, is an attractive small molecule therapeutic target aimed at the treatment of PD and other neurological diseases caused by amyloidosis (Alzheimer’s disease, Huntington’s disease, and prion protein disorders) [25]. Our ongoing research in this area has reported several NPs with α-syn binding and/or anti-aggregation activities from both marine and terrestrial environments, highlighting the promise of NP scaffolds as potential therapeutics for amyloid protein diseases. We have previously reported two bioisosteres of IGAs, aplysamine-2 (**57**) and purealidine Q, both with micromolar anti-prion activities in a yeast-based assay (Figure 5) [25]. In addition, we have reported several brominated MNPs with α-syn binding and/or protein aggregation inhibitory activities, including aerothionin (**58**), aerophobin-2 (**59**), and butenolide-containing prunolides (prunolide B, **60**) and procerolides (procerolide C, **61**, Figure 5) [26,27]. Several of these MNPs showed dual yeast prion curing and α-syn aggregation inhibition. Given that synthetic IGAs have also been reported to potently inhibit human prion protein aggregation, this prompted us to test the synthetic brominated IGA analogues for binding affinity to α-syn using an affinity mass spectrometry assay.

**Figure 6:**
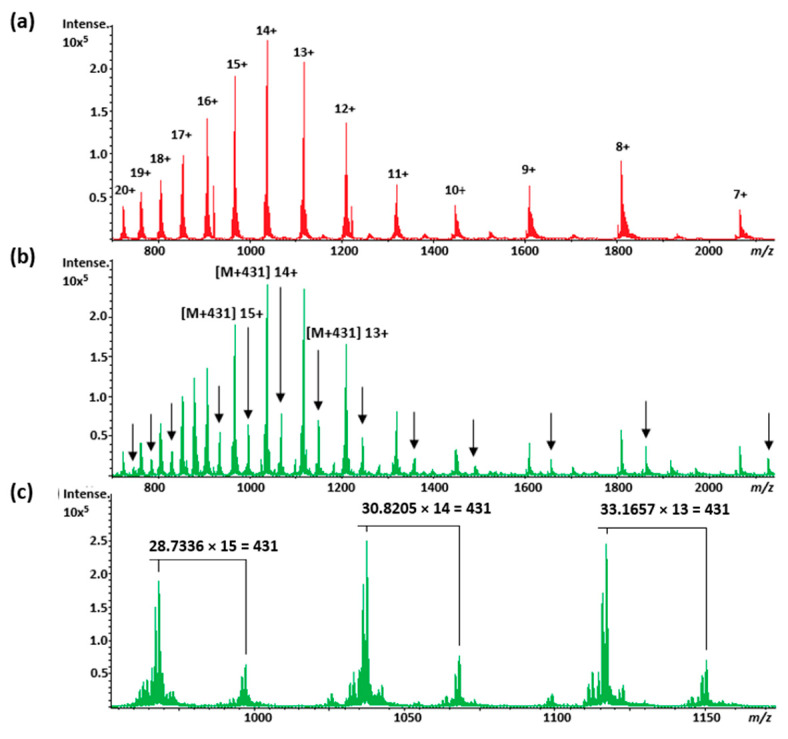
α-synuclein binding assay. (**a**) Mass spectrum for untreated α-syn, with the peaks marked by their charged state. (**b**) Mass spectrum for α-syn treated with 6-bromoindolyl-3-glyoxyl-D-tyrosine (**52**), with arrows indicating the additional peaks representing a 1:1 α-synuclein:6-bromoindolyl-3-glyoxyl-D-tyrosine (**52**) complex. (**c**) The deconvolution process affording the mass of the bound compound.

To broadly cover the diversity of amino acid side-chain physicochemical properties housed within our library of synthetic IGAs (and to minimise expenses associated with screening 32 compounds), 5 and 6-brominated IGAs with arginine (**25** and **26**), cystine (**34**), isoleucine (**41**), tryptophan (**45** and **46**), and tyrosine (**51** and **52**) were selected for screening in an in vitro affinity mass spectrometry (MS) assay. The synthetic indoles were incubated with α-syn for 3 h at a 5:1 molar ratio, after which a (+)- mode high-resolution MS spectrum was acquired. The MS binding results displayed additional peaks indicating that all the analogues tested formed protein-ligand complexes indicative of their binding with α-syn (Figure 6 and Figures S134–S140). The α-syn binding displayed by all of the synthetic IGAs tested, **25**, **26**, **34**, **41**, **45**, **46**, **51**, and **52,** provides compelling data that supports the antiprion CNS activities reported for related IGA analogues [13].

#### 2.3.2. Antiplasmodial Activity—*Plasmodium falciparum*

Malaria, a vector-borne parasitic disease transmitted to humans by mosquitoes, continues to burden the health of lower socioeconomic countries, with an estimated 241 million cases reported in 2020 [28,29]. Traditionally, NP scaffolds have provided breakthrough treatments for malaria. However, ongoing drug resistance continues to emerge in the *Plasmodium falciparum* parasite, posing a significant threat to the effectiveness of current frontline drug treatments and highlighting the importance of discovering new ones. Despite this, the testing of MIAs for antiplasmodial activities has decreased by more than 50% since 2010 [3], a worrying trend considering the recent emergence of other zoonotic diseases (i.e., SARS-CoV-2 virus) and the importance of NP scaffolds for the development of drug leads to treat these diseases.

Although MNP IGAs previously examined for antiplasmodial activities were found to be inactive [16,17], the bioactivities reported for synthetic IGAs against another protozoan target *Leishmania donovani* provided additional rationale for expanded screening [13]. We decided to explore the enhanced chemical diversity provided by our synthetic library, incorporating D and L-amino acids and brominated indole, in an attempt to expand our current knowledge of IGA antiplasmodial biological space.

The synthetic IGAs herein were screened for inhibition of chloroquine-sensitive (3D7) and chloroquine-resistant (Dd2) strains of the malarial parasite *P. falciparum*, and for cytotoxic effects against human embryonic kidney (HEK293) cells (Table 2). The aromatic D-amino acid IGA analogues, D-tryptophan (**46** and **48**) and D-tyrosine (**50**), possessed the highest inhibitory activity against both chloroquine-sensitive and resistant strains, approaching 100% inhibition at 60 µM. Further, 5-bromoindole-3-glyoxyl-D-tryptophan (**46**) was the most potent compound against both 3D7 and Dd2 (IC_50_ 7.4 and 8.2 µM, respectively) parasite strains and also showed excellent cellular safety towards HEK293 cells up to 60 µM. In fact, all of the IGAs tested were inactive against HEK293 cells. Some clear trends were observed for the antiplasmodial activities of brominated IGAs with larger aromatic amino acids side chains (tryptophan and tyrosine) possessing more potent growth inhibition compared to aliphatic ones, while D amino acids displayed higher potencies compared to L configured residues (D-arginine > L-arginine, D-tryptophan > L-tryptophan, and D-tyrosine > L-tyrosine). The activities obtained for the brominated arginine containing IGAs are consistent with those previously reported for the non-brominated arginine enantiomers of leptoclinidamine A (**1**), also tested against both 3D7 and Dd2 strains of *P. falciparum* [16]. Moreover, bromination at C-5 or C-6 of indole does not appear to affect the antiplasmodial activities observed for the synthetic indoles examined herein.

#### 2.3.3. SARS-CoV-2 3CL Protease Activity

**Figure 7:**
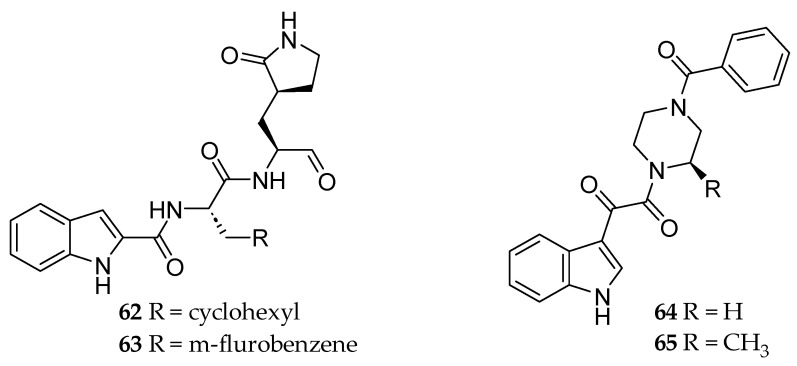
Synthetic functionalised indoles with antiviral activities; SARS-CoV-2 3CL^Pro^ inhibitors **62** and **63**, and HIV-1 inhibitors **64** and **65**.

Despite the World Health Organisation declaring an end to the public health emergency caused by severe acute respiratory syndrome coronavirus 2 (SARS-CoV-2) in May 2023, the virus responsible for the coronavirus disease 2019 (COVID-19) pandemic continues its burden on global health and economic outcomes. With the continuing emergence of drug and vaccine-resistant variants, small-molecule antivirals remain critically important for treating acute infection. The SARS-CoV-2 3-chymotrypsin-like protease (3CL^Pro^), synonymously known as the main protease (M^Pro^), is an attractive small-molecule antiviral drug target for SARS-CoV-2 infection [30]. SARS-CoV-2 3CL^Pro^ is a highly conserved viral protease that, like other coronaviruses, translates its open reading frames into polyproteins that are hydrolysed into mature proteins [30,31,32]. Functionalised indoles have been reported with good antiviral properties [13]. Examples include synthetic C-2 substituted indole amides **62** and **63** displaying promising nanomolar inhibition of SARS-CoV-2 3CL^Pro^ [33], while IGAs **64** and **65** were reported with nanomolar inhibition of HIV-1 along with low cellular toxicity (Figure 7) [34,35].

To measure the potential antiviral activities of **25**–**56** against SARS-CoV-2 3CL^Pro^, the IGAs were assayed by measuring the viral protease catalysed cleavage of two fluorescent proteins in the presence and absence of dithiothreitol (DTT) [31]. The 5-bromo-L-serine IGA (**43**) was the most potent inhibitor of SARS-CoV-2 3CL^Pro^ (IC_50_ 1.2 µM, Table S3), followed by brominated cystine dimers **33** and **34** (IC_50_ 12.5 and 6.4 µM, respectively). Interestingly, 5-brominated-L-serine IGA **43** displayed inhibition 14 times more potent than its 6-brominated constitutional isomer **44**, suggesting that bromination of indole at C-5 is favourable for the SARS-CoV-2 3CL^Pro^ activity for serine-substituted IGAs.

**Figure 8:**
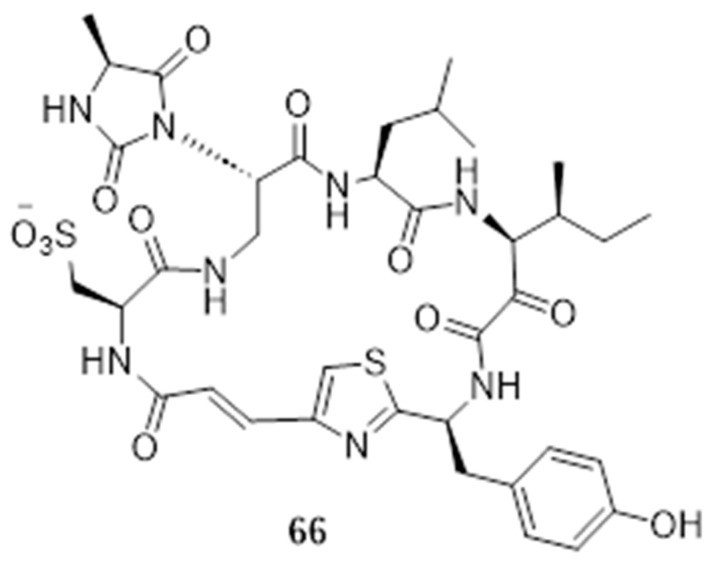
Cyclotheonellazole D (**66**) a nanomolar potent inhibitor of elastase isolated from an Australian marine sponge (*Theonella* sp.) featuring α-keto-β-amide residue.

In the presence of the reducing agent DTT (4 mM), all of the synthetic indoles displayed significantly weaker inhibition of the viral protease. A loss of inhibition in the presence of DTT is proposed to occur in compounds with thiol reactivities, indicating nonspecific covalent binding with cysteine proteases [32]. We recently examined the effect of DTT on the structure of cyclotheonellazole D (**66**, Figure 8), a cyclic marine peptide incorporating an α-keto-β-amide (3-amino-4-methyl-2-oxohexanoic acid) similar to that found in IGAs [36]. ^1^H NMR analyses of samples with and without DTT (4 mM) showed that the reducing agent had no effect on the structure of cyclotheonellazole D; however, its presence led to more than eightfold reduction in the inhibition of SARS-CoV-2 3CL^Pro^ despite the marine peptide containing no obvious sites for thiol reactivity. Therefore, we urge caution when interpreting the SARS-CoV-2 3CL^Pro^ results in the presence of reducing agents, particularly for compounds that might bind proteins allosterically and for those with α-keto-β-amide moieties, that for example, bind via covalent tetrahedral hemiketal complexes with serine proteases [37,38].

#### 2.3.4. Mammalian Serine Protease (Chymotrypsin and Elastase) Activity

Serine proteases are a super-family of enzymes responsible for catalysing the hydrolytic cleavage of amide bonds in peptides and proteins via a nucleophilic active-site serine hydroxyl group [39]. Elastases, proteolytic enzymes that degrade connective tissue, are involved in the pathogenesis of various inflammatory diseases and have been implicated in acute respiratory distress syndrome (ARDS) and acute lung injury (ALI) caused by viral infections such as SARS-CoV-2 [40,41].

Recently, we reported a series of nanomolar potent elastase inhibitors containing α-keto-β-amide residues from an Australia *Theonella* sponge species (Figure 8) [36]. Because synthetic IGAs **25**–**56** all contain α-keto-β-amide functionality, a characteristic residue in other potent MNP serine protease inhibitors [36,37,38,41], we examined their potential inhibitions of elastase and chymotrypsin at 10 µM (Table S4). The 5-bromoindole tryptophan IGAs **45** and **46** were found to inhibit chymotrypsin at IC_50_ 16.3 and 6.3 µM, respectively, with the D-tryptophan analogue being most potent. Interestingly, only 5-brominated tryptophan IGAs displayed quantifiable activity against chymotrypsin up to 10 µM. In addition, none of the IGAs **25**–**56** significantly inhibited elastase despite containing α-keto-β-amide functionalities found in other NP elastase inhibitors.

#### 2.3.5. Cytotoxicity against Human Breast, Ovarian, and Colon Cancer Cell Lines

The brominated IGAs (**25**–**42** and **44**–**56**) were tested for inhibition of cell growth of human breast (MDA-231), ovarian (OVCAR8), and colon (HCT-116) cancer cell lines. Consistent with the lack of cytotoxicity reported for other MNP IGAs, none of the synthetic compounds were cytotoxic at concentrations <20 µM. These results, along with the demonstrated cellular safety of IGAs against non-cancerous HEK293 cells (up to 60 µM), support their assessment in functional assays where cellular toxicity is unwanted.

## 3. Discussion

The marine-inspired brominated IGAs **25**–**56** synthesised herein presented promising bioactivities against several non-toxic disease targets. In addition, because IGAs are amenable to synthetic modification, this would likely improve their potencies against specific disease and infection targets.

The progression of neurodegenerative diseases, such as Parkinson’s, Alzheimer’s, Huntington’s, and Creutzfeldt-Jakob disease, alongside metabolic disorders and cancers, has been linked to the accumulation of misfolded proteins and their aggregation into insoluble amyloid fibrils [42]. The brominated IGAs tested were arginine (**25** and **26**), cystine (**34**), isoleucine (**41**), tryptophan (**45** and **46**), and tyrosine (**51** and **52**), all displayed promising binding activities with α-syn, consistent with clinically relevant antiprion CNS activities reported for other synthetic IGAs [43]. We suggest further exploration of marine IGA scaffolds targeting amyloid protein diseases, particularly those IGAs substituted with aromatic amino acids, as excellent starting points, given the nanomolar antiprion activity reported for structurally related IGAs [13,43].

**Figure 9:**
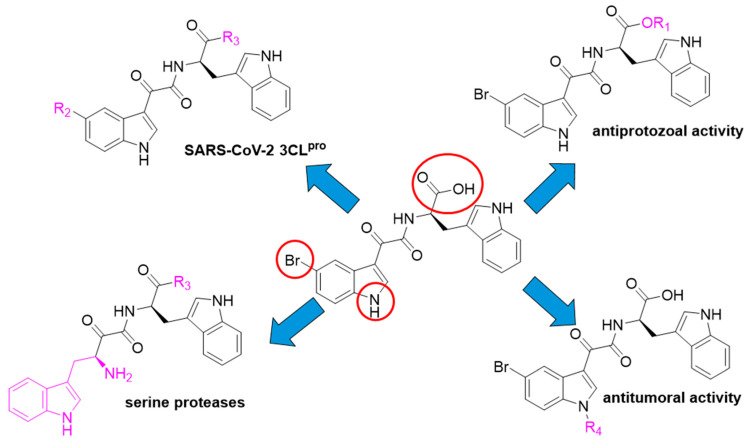
Potential sites for synthetic modification of brominated IGA scaffolds (red circles) to improve potency and disease target specificity. Antiprotozoal activity; R_1_ = esterification, mammalian and viral proteases; R_2_ = Suzuki-coupling to bromine (at C-5 or C-6 of indole) and/or R_3_ = amide coupling (amines or amino acids), and antitumoral activity; R_4_ = *N*-alkylation.

Based on the antileishmanial results reported for IGAs against the human blood parasite *L. donovani*, we would anticipate an improvement in the potency of antiplasmodial activity for brominated IGA amino acid analogues if they were converted to methyl esters and/or the tryptophan amino acid analogues cyclised with benzaldehydes to form aryl tetrahydro-β-carbolines (Figure 9) [13,44]. Moreover, the SAR for antiplasmodial bioactivity obtained here clearly favoured D-aromatic amino acid IGA analogues **46**, **48**, and **50**, scaffolds worthy of further modification and investigation against *P. falciparum* and other human pathogenic protozoan targets. Although only a handful of the IGAs synthesised herein exhibited meaningful inhibition of SARS-CoV-2 3CL^pro^, namely the cysteine dimers **33** and **34** and 5-bromo-serine IGA **43**, further investigation of these scaffolds after synthetic modification is warranted. Because the IGAs **25**–**56** feature 5- or 6-brominated indoles, there is an excellent opportunity to increase their structural diversity using Suzuki coupling synthetic strategies (Figure 9). Furthermore, the C-terminus of the synthetic IGA scaffold is also available for coupling with additional amino acids or amines. These synthetic modifications are expected to expand both the size and structural diversity of marine IGA scaffolds while allowing for the exploration of favourable substrate interactions with the active site of the viral protease [30].

Similar synthetic modifications described for SARS-CoV-2 3CL^pro^ could also form the basis for improving the specificity and potency of IGAs toward mammalian serine proteases. Based on our results, 5-brominated D-tryptophan IGA **46** is an ideal target (alongside other IGAs) for further exploration against chymotrypsin after synthetic modification that extends the IGA substrate at one or both ends utilising Suzuki and/or amide coupling reactions (Figure 9). Interestingly, none of the synthetic IGAs **25**–**46** displayed significant inhibition of elastase despite containing α-keto-β-amide functionality observed in other nanomolar potent marine NP inhibitors [36,37,38,41]. Based on the formation of a tetrahedral hemiketal between the α-keto-β-amide and Ser195 of trypsin [37], we propose that the extended conjugation of indole α-keto-β-amide results in inefficient formation of this transition state, thereby compromising binding efficiency. Synthetic efforts that introduce the α-keto-β-amide distal to the indole C-3 to disrupt resonance with the α-keto-β-amide group could be expected to improve the binding efficiency of IGAs with serine proteases (Figure 9). Finally, although the non-toxic activities of the synthetic IGAs **25**–**56** were a primary focus of this work, it appears that *N*-substituted IGAs coupled with planar aromatic amines are a consistent structural feature required for antitumoral bioactivities [13].

## 4. Materials and Methods

### 4.1. Chemistry

All anhydrous reaction and bulk solvents and reagents were purchased from Merck (Bayswater, Victoria, Australia) except for 5- and 6-bromoindole (Enamine), and bulk solvents were distilled prior to use. All reactions were carried out under inert N_2_ or argon atmospheres under standard reaction conditions. Reactions were heated using a magnetic stirrer hotplate and appropriate heating block. UV spectra were recorded on a Shimadzu UV-1800 spectrophotometer (Kyoto, Japan), optical rotations were acquired on a JASCO P-1020 polarimeter (Kingsgrove, NSW, Australia) with [α]_D_ values given in 10^−1^ deg cm^2^ g^−1^, and IR data was obtained in MeOH using a Thermo Fisher Scientific Nicolet iS5 spectrometer (Parkville, Victoria, Australia) equipped with an iD5 ATR accessory (Thermo Fisher). NMR spectra were recorded on an 800 MHz Bruker^®^ Avance III HDX equipped with a Triple Resonance 5 mm Cryoprobe with Z-gradient and automatic tuning with a SampleJet automatic sample changer, a Bruker Avance III 500 MHz spectrometer (BBFO Smartprobe, 5 mm 31P-109Ag), or a Bruker^®^ 400 MHz Avance III spectrometer equipped with a 5 mm BBFO probe with Z-gradient and automatic tuning with a SampleCase automatic sample changer. All NMR spectrometers were controlled using IconNMR™ automation software version 5.1 (all Bruker equipment from Bruker Pty Ltd., Preston, Victoria, Australia), with all spectra acquired at 25 °C using standard Bruker pulse sequences. The ^1^H and ^13^C chemical shifts were referenced to the residual *d*_6_-DMSO (Cambridge isotope Laboratory, Inc., Heidelburg West, Victoria, Australia) solvent peaks, δ_H_ 2.50 and δ_C_ 39.52 respectively, and processed using the Mnova™ software suite (version 12.0). MS spectra and MS/MS experiments were obtained on an Agilent Technologies 5530 accurate mass QTOF LC/MS controlled by Mass Hunter data acquisition and analytical software (Agilent Technologies, version B.04.00, Mulgrave, Victoria, Australia) and equipped an Agilent Technologies 1290 infinity series binary pump and autosampler (Mulgrave, Victoria, Australia). HPLC fractions were dried using a Genevac HT-12 Series II evaporation system (LabGear Australia, South Melbourne, Victoria, Australia). All solvents used for HPLC were Lab-Scan HPLC grade, and H_2_O was Millipore Milli-Q PF (Bayswater, Victoria, Australia) filtered, while solvents used for the performance of MS were LCMS grade.

### 4.2. Synthetic Procedures

#### 4.2.1. Synthesis of Indol-3-yl-Glyoxlyamides **25**–**56**

Under an inert atmosphere oxalyl chloride (30 μL, 0.35 mmol) was added to a solution of 5- or 6-bromoindole (25 mg, 0.13 mmol) in diethyl ether (1.5 mL) and the reaction mixture stirred at room temperature for 45 min. The reaction mixture was heated to 50 °C to remove diethyl ether over 15 min, then resuspended in DMF (1.5 mL) and anhydrous pyridine (50 μL, 0.50 mmol) and heated to 90 °C. After 15 min, the appropriate D or L amino acid (0.2 mmol) was added, and the crude reaction mixture was stirred overnight at 90 °C. Following this, the reaction mixture was cooled to room temperature and partitioned repeatedly between EtOAc and water, with the organic phase dried over sodium sulfate, filtered, and concentrated to dryness.

#### 4.2.2. HPLC Purification of Analogues **25**–**56**

A Merck Hitachi HPLC system containing a D-7000 interface module, L-7100 pump, a L-7455 UV-PDA detector, and a Gilson 215 liquid handler was used for HPLC separations. Altech Davisil C_18_ bonded silica gel (55–100 μm, 60 Å) was used to adsorb reaction mixtures for preparative RP HPLC purification. Each of the synthetic indol-3-ylglyoxyl amides reported herein was adsorbed onto C_18_ bonded silica gel and loaded into an HPLC pre-column cartridge (10 mm × 20 mm) connected in series to a Kinetex^®^ 5 μm EVO C_18_ 100 Å preparative HPLC column (150 × 21.2 mm) eluting with a solvent gradient from 100% H_2_O (0.1% TFA) to 100% MeOH (0.1% TFA) over 60 min at a flow rate 9.0 mL/min with fractions collected each min. If further purification was required, each semi-pure sample was injected onto a semi-preparative Betasil C_18_ HPLC column (250 × 10 mm), eluting with a solvent gradient from 40% MeOH/60% H_2_O (0.1% TFA) to 75% MeOH/25% H_2_O (0.1% TFA) for 40 min at a flow rate of 4.0 mL per min with fractions collected manually.

*5-bromoindolyl-3-glyoxyl-L-alanine* (**25**): brown amorphous solid, 7.0 mg; [α]^23^_D_ = +17.3 (c 0.05, MeOH); UV (MeOH) λ_max_ (log ε) 320 (3.9), 273 (3.9), 260 (4.1) and 217 (4.3) nm; IR (FTIR) ν_max_ 3270, 2981, 1727, 1672, 1625, 1499, 1445 and 1429 cm^−1^; ^1^H NMR (500 MHz, DMSO) δ 12.43 (br s, 1H), 8.91 (d, *J* = 7.5 Hz, 1H), 8.73 (d, *J* = 2.7 Hz, 1H), 8.35 (d, *J* = 1.9 Hz, 1H), 7.52 (d, *J* = 8.5 Hz, 1H), 7.42 (dd, *J* = 8.5, 1.9 Hz, 1H), 4.36 (qd, *J* = 7.3, 7.5 Hz 1H), 1.40 (d, *J* = 7.3 Hz, 3H); ^13^C NMR (126 MHz, DMSO) δ 181.9, 173.5, 163.1, 139.4, 135.1, 127.9, 126.1, 123.3, 115.4, 114.7, 111.7, 47.6, 16.8; HRMS (ESI-QTOF) *m*/*z* [M − H]^−^ Found 338.9805 (calcd. 338.9804 for C_13_H_10_^81^BrN_2_O_4_) (NMR spectra, Figures S6–S9).

*5-bromoindolyl-3-glyoxyl-D-alanine* (**26**): brown amorphous solid, 7.4 mg; [α]^23^_D_ = −17.0 (c 0.05, MeOH); UV (MeOH) λ_max_ (log ε) 322 (3.9), 273 (3.9), 260 (3.9) and 216 (4.3) nm; IR (FTIR) ν_max_ 3270, 2981, 1727, 1672, 1625, 1499, 1445 and 1429 cm^−1^; ^1^H NMR (400 MHz, DMSO) δ 12.43 (br s), 8.91 (d, *J* = 7.5 Hz, 1H), 8.73 (d, *J* = 3.2 Hz, 1H), 8.35 (d, *J* = 1.9 Hz, 1H), 7.52 (d, *J* = 8.5 Hz, 1H), 7.41 (dd, *J* = 8.5, 1.9 Hz, 1H), 4.37 (qd, *J* = 7.3, 7.5 Hz, 1H), 1.40 (d, *J* = 7.3 Hz, 3H); ^13^C NMR (101 MHz, DMSO) δ 181.9, 173.5, 163.2, 139.4, 135.1, 127.9, 126.1, 123.4, 115.4, 114.7, 111.7, 47.6, 16.8; HRMS (ESI-QTOF) *m*/*z* [M − H]^−^ Found 338.9805 (calcd. 338.9809 for C_13_H_10_^81^BrN_2_O_4_) (NMR spectra, Figures S10–S13).

*6-bromoindolyl-3-glyoxyl-L-alanine* (**27**): brown amorphous solid, 21.3 mg; [α]^23^_D_ = +18.2 (c 0.05, MeOH); UV (MeOH) λ_max_ (log ε) 324 (3.9), 275 (4.0), 260 (4.0) and 213 (4.3) nm; IR (FTIR) ν_max_ 3292, 2980, 1730, 1671, 1626, 1500, 1439 and 1413 cm^−1^; ^1^H NMR (500 MHz, DMSO) δ 12.33 (d, *J* = 3.2 Hz, 1H), 8.91 (d, *J* = 7.5 Hz, 1H), 8.72 (d, *J* = 3.2 Hz, 1H), 8.15 (d, *J* = 8.5 Hz, 1H), 7.74 (d, *J* = 1.8 Hz, 1H), 7.40 (dd, *J* = 8.5, 1.8 Hz, 1H), 4.37 (qd, *J* = 7.3, 7.5 Hz, 1H), 1.40 (d, *J* = 7.3 Hz, 3H); ^13^C NMR (126 MHz, DMSO) δ 182.1, 173.5, 163.3, 139.3, 137.3, 125.5, 125.2, 122.9, 116.1, 115.4, 112.2, 47.6, 16.8; HRMS (ESI-QTOF) *m*/*z* [M − H]^−^ Found 338.9804 (calcd. 338.9804 for C_13_H_10_^81^BrN_2_O_4_) (NMR spectra, Figures S14–S17).

*6-bromoindolyl-3-glyoxyl-D-alanine* (**28**): brown amorphous solid, 14.6 mg; [α]^23^_D_ = −18.2 (c 0.05, MeOH); UV (MeOH) λ_max_ (log ε) 324 (3.8), 276 (3.9), 260 (3.9) and 212 (4.2) nm; IR (FTIR) ν_max_ 3292, 2980, 1730, 1671, 1626, 1500, 1439 and 1413 cm^−1^; ^1^H NMR (400 MHz, DMSO) δ 12.32 (d, *J* = 3.2 Hz, 1H), 8.91 (d, *J* = 7.5 Hz, 1H), 8.71 (d, *J* = 3.2 Hz, 1H), 8.15 (d, *J* = 8.5 Hz, 1H), 7.74 (d, *J* = 1.8 Hz, 1H), 7.41 (dd, *J* = 8.5, 1.8 Hz, 1H), 4.37 (qd, *J* = 7.3, 7.5 Hz, 1H), 1.39 (d, *J* = 7.3 Hz, 3H);^13^C NMR (101 MHz, DMSO) δ 182.1, 173.5, 163.3, 139.2, 137.3, 125.5, 125.1, 122.9, 116.0, 115.4, 112.2, 47.6, 16.8; HRMS (ESI-QTOF) *m*/*z* [M − H]^−^ Found 338.9810 (calcd. 338.9804 for C_13_H_10_^81^BrN_2_O_4_) (NMR spectra, Figures S18–S21).

*5-bromoindolyl-3-glyoxyl-L-arginine* (**29**): brown amorphous solid; 2.5 mg; [α]^23^_D_ = +14.9 (c 0.04, MeOH); UV (MeOH) λ_max_ (log ε) 323 (3.5), 274 (3.5), 260 (3.6) and 215 (4.0) nm; IR (FTIR) ν_max_ 3347, 3195, 1668, 1663, 1202 and 1137 cm^−1^; ^1^H NMR (500 MHz, DMSO) δ 12.49 (d, *J* = 3.2 Hz, 1H), 8.90 (d, *J* = 7.9 Hz, 1H), 8.72 (d, *J* = 3.2 Hz, 1H), 8.35 (d, *J* = 1.9 Hz, 1H), 7.59 (t, *J* = 5.7 Hz, 1H), 7.53 (d, *J* = 8.6 Hz, 1H), 7.42 (dd, *J* = 8.6, 2.0 Hz, 1H), 4.32 (ddd, *J* = 9.3, 7.9, 4.7 Hz, 1H), 3.12 (m, 2H), 1.88 (m, 1H), 1.79 (m, 1H), 1.54 (q, *J* = 7.5 Hz, 2H); ^13^C NMR (126 MHz, DMSO) δ 181.8, 172.7, 163.5, 156.7, 139.4, 135.2, 127.9, 126.2, 123.3, 115.5, 114.8, 111.7, 51.7, 40.4, 27.6, 25.4; HRMS (ESI-QTOF) *m*/*z* [M + H]^+^ Found 426.0605 (calcd. 426.0602 for C_16_H_19_^81^BrN_5_O_4_) (NMR spectra, Figures S22–S25).

*5-bromoindolyl-3-glyoxyl-D-arginine* (**30**): brown amorphous solid; 4.2 mg; [α]^23^_D_ = −17.6 (c 0.04, MeOH); UV (MeOH) λ_max_ (log ε) 322 (3.6), 274 (3.6), 260 (3.7) and 215 (4.1) nm; IR (FTIR) ν_max_ 3347, 3195, 1668, 1663, 1202 and 1137 cm^−1^; ^1^H NMR (800 MHz, DMSO) δ 12.51 (d, *J* = 3.2 Hz, 1H), 8.89 (d, *J* = 7.9 Hz, 1H), 8.72 (d, *J* = 3.2 Hz, 1H), 8.35 (d, *J* = 1.9 Hz, 1H), 7.64 (t, *J* = 5.7 Hz, 1H), 7.53 (d, *J* = 8.6 Hz, 1H), 7.42 (dd, *J* = 8.6, 2.0 Hz, 1H), 4.32 (ddd, *J* = 9.3, 7.9, 4.6 Hz, 1H), 3.12 (m, 2H), 1.89 (m, 1H), 1.79 (m, 1H), 1.54 (m, 2H);^13^C NMR (201 MHz, DMSO) δ 181.8, 172.7, 163.6, 156.7, 139.4, 135.2, 127.9, 126.2, 123.3, 115.4, 114.8, 111.7, 51.7, 40.4, 27.6, 25.4; HRMS (ESI-QTOF) *m*/*z* [M + H]^+^ Found 426.0607 (calcd. 426.0602 for C_16_H_19_^81^BrN_5_O_4_) (NMR spectra, Figures S26–S29).

*6-bromoindolyl-3-glyoxyl-L-arginine* (**31**): brown amorphous solid; 12.0 mg; [α]^23^_D_ = +18.1 (c 0.05, MeOH); UV (MeOH) λ_max_ (log ε) 323 (3.4), 274 (3.4), 260 (3.5) and 215 (3.8) nm; IR (FTIR) ν_max_ 3340, 3195, 2979, 1667, 1631, 1501, 1440, 1417, 1201, and 1137 cm^−1^; ^1^H NMR (800 MHz, DMSO) δ 12.36 (d, *J* = 3.2 Hz, 1H), 8.89 (d, *J* = 7.9 Hz, 1H), 8.70 (d, *J* = 3.2 Hz, 1H), 8.15 (d, *J* = 8.5 Hz, 1H), 7.76 (d, *J* = 1.8 Hz, 1H), 7.53 (t, *J* = 5.7 Hz, 1H), 7.42 (dd, *J* = 8.5, 1.8 Hz, 1H), 4.32 (ddd, *J* = 9.3, 7.9, 4.6 Hz, 1H), 3.11 (m, 2H), 1.88 (m 1H), 1.78 (m, 1H), 1.53 (m, 2H); ^13^C NMR (201 MHz, DMSO) δ 181.9, 172.7, 163.6, 158.1, 156.6, 139.2, 137.3, 125.5, 125.1, 122.8, 116.1, 115.4, 112.2, 51.7, 40.0, 27.6, 25.3; HRMS (ESI-QTOF) *m*/*z* [M + H]^+^ Found 426.0598 (calcd. 426.0602 for C_16_H_19_^81^BrN_5_O_4_) (NMR spectra, Figures S30–S33).

*6-bromoindolyl-3-glyoxyl-D-arginine* (**32**): brown amorphous solid; 10.2 mg; [α]^23^_D_ = −19.0 (c 0.05, MeOH); UV (MeOH) λ_max_ (log ε) 325 (3.7), 276 (3.9), 260 (3.8) and 213 (4.2) nm; IR (FTIR) ν_max_ 3347, 3196, 2979, 1669, 1631, 1501, 1440, 1417, 1201, and 1137 cm^−1^; ^1^H NMR (500 MHz, DMSO) δ 12.43 (d, *J* = 3.2 Hz, 1H), 8.89 (d, *J* = 7.9 Hz, 1H), 8.70 (d, *J* = 3.2 Hz, 1H), 8.15 (d, *J* = 8.5 Hz, 1H), 7.76 (d, *J* = 1.7 Hz, 1H), 7.68 (t, *J* = 5.6 Hz, 1H), 7.41 (dd, *J* = 8.5, 1.7 Hz, 1H), 4.33 (ddd, *J* = 9.3, 7.9, 4.6 Hz, 1H), 3.12 (m, 2H), 1.88 (m 1H), 1.79 (m, 1H), 1.53 (m, 2H); ^13^C NMR (125 MHz, DMSO) δ 182.0, 172.8, 163.7, 156.8, 139.2, 137.4, 125.5, 125.1, 122.9, 116.1, 115.4, 112.2, 51.7, 40.0, 27.6, 25.4; HRMS (ESI-QTOF) *m*/*z* [M + H]^+^ Found 426.0596 (calcd. 426.0602 for C_16_H_19_^81^BrN_5_O_4_) (NMR spectra, Figures S34–S37).

*5-bromoindolyl-3-glyoxyl-L-cystine* (**33**): brown amorphous solid; 12.4 mg; [α]^23^_D_ = −32.0 (c 0.06, MeOH); UV (MeOH) λ_max_ (log ε) 322 (3.5), 274 (3.5), 261 (3.6) and 215 (4.0) nm; IR (FTIR) ν_max_ 3280, 2979, 2889, 1674, 1624, 1498 and 1428 cm^−1^; ^1^H NMR (500 MHz, DMSO) δ 12.41 (d, *J* = 3.2 Hz, 1H), 9.06 (d, *J* = 8.5 Hz, 1H), 8.76 (d, *J* = 3.2 Hz, 1H), 8.34 (d, *J* = 2.0 Hz, 1H), 7.48 (d, *J* = 8.5 Hz, 1H), 7.39 (dd, *J* = 8.5, 2.0 Hz, 1H), 4.70 (ddd, *J* = 9.7, 8.5, 4.2 Hz, 1H), 3.33 (dd, *J* = 13.8, 4.2 Hz, 1H), 3.20 (dd, *J* = 13.8, 9.7 Hz 1H); ^13^C NMR (125 MHz, DMSO) δ 181.1, 171.4, 163.2, 139.6, 135.1, 127.9, 126.1, 123.4, 115.5, 114.7, 111.6, 51.3, 38.6; HRMS (ESI-QTOF) *m*/*z* [M − H]^−^ Found 738.8996 (calcd. 738.8992 for C_26_H_19_^81^Br_2_N_4_O_8_S_2_) (NMR spectra, Figures S38–S41).

*6-bromoindolyl-3-glyoxyl-L-cystine* (**34**): brown amorphous solid; 9.8 mg; [α]^23^_D_ = −34.5 (c 0.06, MeOH); UV (MeOH) λ_max_ (log ε) 323 (3.6), 275 (3.8), 261 (3.8) and 213 (4.2) nm; IR (FTIR) ν_max_ 3280, 2979, 2889, 1674, 1624, 1498 and 1428 cm^−1^; ^1^H NMR (500 MHz, DMSO) δ 12.29 (d, *J* = 3.2 Hz, 1H), 9.07 (d, *J* = 8.3 Hz, 1H), 8.72 (d, *J* = 3.2 Hz, 1H), 8.12 (d, *J* = 8.5 Hz, 1H), 7.68 (d, *J* = 1.8 Hz, 1H), 7.38 (dd, *J* = 8.5, 1.8 Hz, 1H), 4.68 (ddd, *J* = 9.6, 8.3, 4.2 Hz, 1H), 3.30 (dd, *J* = 13.8, 4.2 Hz, 1H), 3.20 (dd, *J* = 13.8, 9.6 Hz 1H); ^13^C NMR (125 MHz, DMSO) δ 181.2, 171.4, 163.2, 139.3, 137.2, 125.5, 125.2, 122.9, 116.0, 115.3, 112.1, 51.3, 38.6; HRMS (ESI-QTOF) *m*/*z* [M − H]^−^ Found 738.8991 (calcd. 738.8992 for C_26_H_19_^81^Br_2_N_4_O_8_S_2_) (NMR spectra, Figures S42–S45).

*5-bromoindolyl-3-glyoxyl-L-glutamic acid* (**35**): brown amorphous solid, 1.4 mg; [α]^23^_D_ = +19.2 (c 0.06, MeOH); UV (MeOH) λ_max_ (log ε) 321 (3.8), 260 (3.9) and 216 (4.3) nm; IR (FTIR) ν_max_ 3265, 2979, 1719, 1678, 1631, 1501, 1428, 1232 and 1149 cm^−1^; ^1^H NMR (500 MHz, DMSO) δ 12.43 (d, *J* = 3.2 Hz, 1H), 8.92 (d, *J* = 8.1 Hz, 1H), 8.71 (d, *J* = 3.2 Hz, 1H), 8.35 (d, *J* = 1.9 Hz, 1H), 7.52 (d, *J* = 8.7 Hz, 1H), 7.42 (dd, *J* = 8.7, 1.9 Hz, 1H), 4.36 (ddd, *J* = 9.5, 8.1, 4.8 Hz, 1H), 2.32 (d, *J* = 7.3 Hz, 2H), 2.13(m, 1H) and 1.97 (m, 1H); ^13^C NMR (126 MHz, DMSO) δ 181.8, 173.8, 172.6, 163.6, 139.4, 135.1, 127.9, 126.1, 123.3, 115.4, 114.8, 111.7, 51.3, 30.2 and 25.7; HRMS (ESI-QTOF) *m*/*z* [M − H]^−^ Found 396.9865 (calcd. 396.9859 for C_15_H_12_^81^BrN_2_O_6_) (NMR spectra, Figures S46–S49).

*5-bromoindolyl-3-glyoxyl-D-glutamic acid* (**36**): brown amorphous solid, 18.6 mg; [α]^23^_D_ = −15.5 (c 0.06, MeOH); UV (MeOH) λ_max_ (log ε) 323 (3.9), 260 (4.1) and 213 (4.4) nm; IR (FTIR) ν_max_ 3269, 2979, 1720, 1678, 1629, 1499, 1428, 1231 and 1149 cm^−1^; ^1^H NMR (500 MHz, DMSO) δ 12.46 (br s, 1H), 8.92 (d, *J* = 8.1 Hz, 1H), 8.73 (d, *J* = 2.6 Hz, 1H), 8.36 (d, *J* = 1.9 Hz, 1H), 7.53 (d, *J* = 8.6 Hz, 1H), 7.42 (dd, *J* = 8.6, 1.9 Hz, 1H), 4.38 (ddd, *J* = 9.4, 8.1, 4.8 Hz, 1H), 2.33 (t, *J* = 7.3 Hz, 2H), 2.13 (m, 1H) and 1.98 (m, 1H); ^13^C NMR (126 MHz, DMSO) δ 181.9, 173.9, 172.7, 163.6, 139.5, 135.2, 127.9, 126.2, 123.4, 115.5, 114.8, 111.8, 51.4, 30.3 and 25.8; HRMS (ESI-QTOF) *m*/*z* [M − H]^−^ Found 396.9858 (calcd. 396.9859 for C_15_H_12_^81^BrN_2_O_6_) (NMR spectra, Figures S50–S53).

*6-bromoindolyl-3-glyoxyl-L-glutamic acid* (**37**): brown amorphous solid, 4.4 mg; [α]^23^_D_ = +14.0 (c 0.05, MeOH); UV (MeOH) λ_max_ (log ε) 325 (3.6), 276 (3.8), 260 (3.7) and 213 (4.1) nm; IR (FTIR) ν_max_ 3654, 3345, 2979, 2888, 1621, 1392, 1250, 1151, 1072 and 954 cm^−1^; ^1^H NMR (500 MHz, DMSO) δ 12.33 (d, *J* = 2.6 Hz, 1H), 8.92 (d, *J* = 8.1 Hz, 1H), 8.69 (d, *J* = 3.2 Hz, 1H), 8.15 (d, *J* = 8.5 Hz, 1H), 7.74 (d, *J* = 1.7 Hz, 1H), 7.41 (dd, *J* = 8.5, 1.7 Hz, 1H), 4.36 (ddd, *J* = 9.4, 8.1, 4.8 Hz, 1H), 2.32 (t, *J* = 7.3, 2H), 2.11 (m, 1H), 1.96 (m, 1H);^13^C NMR (126 MHz, DMSO) δ 182.0, 173.8, 172.7, 163.7, 139.2, 137.3, 125.5, 125.1, 122.9, 116.0, 115.4, 112.2, 51.3, 30.2, 25.7; HRMS (ESI-QTOF) *m*/*z* [M − H]^−^ Found 396.9862 (calcd. 396.9859 for C_15_H_12_^81^BrN_2_O_6_) (NMR spectra, Figures S54–S57).

*6-bromoindolyl-3-glyoxyl-D-glutamic acid* (**38**): brown amorphous solid, 3.0 mg; [α]^23^_D_ = −14.5 (c 0.06, MeOH); UV (MeOH) λ_max_ (log ε) 322 (3.8), 275 (4.0), 260 (4.0) and 213 (4.3) nm; IR (FTIR) ν_max_ 3658, 3241, 2979, 2888, 1614, 1499, 1441, 1393, 1249 and 1154 cm^−1^; ^1^H NMR (500 MHz, DMSO) δ 12.46 (br s, 1H), 8.92 (d, *J* = 8.1 Hz, 1H), 8.73 (d, *J* = 2.6 Hz, 1H), 8.36 (d, *J* = 1.9 Hz, 1H), 7.53 (d, *J* = 8.6 Hz, 1H), 7.42 (dd, *J* = 8.6, 1.9 Hz, 1H), 4.38 (ddd, *J* = 9.5, 8.1, 4.8 Hz, 1H), 2.33 (t, *J* = 7.3 Hz, 2H), 2.13 (m, 1H) and 1.98 (m, 1H); ^13^C NMR (126 MHz, DMSO) δ 181.9, 173.9, 172.7, 163.6, 139.5, 135.2, 127.9, 126.2, 123.4, 115.5, 114.8, 111.8, 51.4, 30.3 and 25.8; HRMS (ESI-QTOF) *m*/*z* [M − H]^−^ Found 396.9863 (calcd. 396.9859 for C_15_H_12_^81^BrN_2_O_6_) (NMR spectra, Figures S58–S61).

*5-bromoindolyl-3-glyoxyl-L-histidine* (**39**): brown amorphous solid; 10.2 mg; [α]^23^_D_ = +29.1 (c 0.03, MeOH); UV (MeOH) λ_max_ (log ε) 321 (3.7), 274 (3.7), 260 (3.8) and 215 (4.2) nm; IR (FTIR) ν_max_ 3655, 2979, 2888, 1676, 1632, 1391 and 1148 cm^−1^; ^1^H NMR (500 MHz, DMSO) δ 12.51 (d, *J* = 3.2 Hz, 1H), 9.07 (d, *J* = 8.3 Hz, 1H), 8.98 (d, *J* = 1.3 Hz, 1H), 8.69 (d, *J* = 3.2 Hz, 1H), 8.33 (d, *J* = 2.0 Hz, 1H), 7.53 (d, *J* = 8.5 Hz, 1H), 7.42 (dd, *J* = 8.5, 2.0 Hz, 1H), 7.40 (br s, 1H), 4.68 (ddd, *J* = 9.8, 8.3, 4.8 Hz, 1H), 3.30 (dd, *J* = 15.3, 4.8 Hz, 1H), 3.21 (dd, *J* = 15.3, 9.8 Hz, 1H); ^13^C NMR (126 MHz, DMSO) δ 181.1, 171.4, 163.1, 139.5, 135.1, 134.0, 129.6, 127.9, 126.2, 123.3, 117.0, 115.5, 114.8, 111.5, 51.2, 25.7; HRMS (ESI-QTOF) *m*/*z* [M − H]^−^ Found 405.0028 (calcd. 405.0023 for C_16_H_12_^81^BrN_4_O_4_) (NMR spectra, Figures S62–S65).

*6-bromoindolyl-3-glyoxyl-L-histidine* (**40**): brown amorphous solid; 10.7 mg; [α]^23^_D_ = +26.7 (c 0.06, MeOH); UV (MeOH) λ_max_ (log ε) 323 (3.6), 276 (3.7), 260 (3.7) and 213 (4.1) nm; IR (FTIR) ν_max_ 3654, 2979, 2888, 1672, 1626, 1392 and 1149 cm^−1^; ^1^H NMR (500 MHz, DMSO) δ 12.42 (d, *J* = 3.2 Hz, 1H), 9.07 (d, *J* = 8.4 Hz, 1H), 8.98 (d, *J* = 1.3 Hz, 1H), 8.67 (d, *J* = 3.2 Hz, 1H), 8.13 (d, *J* = 8.4 Hz, 1H), 7.75 (d, *J* = 1.8 Hz, 1H), 7.41 (dd, *J* = 8.4, 1.8 Hz, 1H), 7.40 (br s, 1H), 4.68 (ddd, *J* = 9.8, 8.4, 4.8 Hz, 1H), 3.29 (dd, *J* = 15.3, 4.8 Hz, 1H), 3.21 (dd, *J* = 15.3, 9.8 Hz, 1H); ^13^C NMR (126 MHz, DMSO) δ 181.2, 171.4, 163.2, 139.3, 137.3, 134.0, 129.6, 125.6, 125.2, 122.9, 117.1, 116.1, 115.4, 112.0, 51.2, 25.7; HRMS (ESI-QTOF) *m*/*z* [M − H]^−^ Found 405.0030 (calcd. 405.0023 for C_16_H_12_^81^BrN_4_O_4_) (NMR spectra, Figures S66–S69).

*5-bromoindolyl-3-glyoxyl-L-isoleucine* (**41**): brown amorphous solid; 7.7 mg; [α]^23^_D_ = +17.7 (c 0.06, MeOH); UV (MeOH) λ_max_ (log ε) 321 (3.8), 273 (3.8), 260 (3.9) and 215 (4.2) nm; IR (FTIR) ν_max_ 3656, 3271, 2979, 2888, 1633, 1408, 1428, 1382, 1232 and 1151 cm^-^1; ^1^H NMR (500 MHz, DMSO) δ 12.44 (d, *J* = 3.2 Hz, 1H), 8.68 (d, *J* = 3.2 Hz, 1H), 8.56 (d, *J* = 8.3 Hz, 1H), 8.34 (d, *J* = 2.0 Hz, 1H), 7.52 (d, *J* = 8.5 Hz, 1H), 7.42 (dd, *J* = 8.5, 2.0 Hz, 1H), 4.29 (dd, *J* = 8.3, 6.0 Hz, 1H), 1.96 (m, 1H), 1.47 (m, 1H), 1.23 (m, 1H), 0.92 (d, *J* = 6.8 Hz, 3H), 0.89 t, *J* = 7.3 Hz, 3H); ^13^C NMR (126 MHz, DMSO) δ 182.0, 172.3, 163.6, 139.3, 135.2, 127.8, 126.2, 123.3, 115.4, 114.8, 111.7, 56.4, 36.1, 24.9, 15.6, 11.2; HRMS (ESI-QTOF) *m*/*z* [M − H]^−^ Found 381.0272 (calcd. 381.0274 for C_16_H_16_^81^BrN_2_O_4_) (NMR spectra, Figures S70–S73).

*6-bromoindolyl-3-glyoxyl-L-isoleucine* (**42**): brown amorphous solid; 8.1 mg; [α]^23^_D_ = +19.7 (c 0.06, MeOH UV (MeOH) λ_max_ (log ε) 323 (3.8), 276 (4.0), 260 (4.0) and 213 (4.3) nm; IR (FTIR) ν_max_ 3657, 3275, 2979, 2888, 1630, 1499, 1439, 1383, 1248 and 1155 cm^−1^; ^1^H NMR (500 MHz, DMSO) δ 12.34 (d, *J* = 3.2 Hz, 1H), 8.65 (d, *J* = 3.2 Hz, 1H), 8.57 (d, *J* = 8.2 Hz, 1H), 8.14 (d, *J* = 8.5 Hz, 1H), 7.74 (d, *J* = 1.8 Hz, 1H), 7.41 (dd, *J* = 8.5, 1.8 Hz, 1H), 4.28 (dd, *J* = 8.2, 6.0 Hz, 1H), 1.95 (m, 1H), 1.47 (m, 1H), 1.23 (m, 1H), 0.92 (d, *J* = 6.8 Hz, 3H), 0.88 (t, *J* = 7.3 Hz, 3H); ^13^C NMR (126 MHz, DMSO) δ 182.1, 172.3, 163.7, 139.1, 137.3, 125.5, 125.0, 122.8, 116.0, 115.4, 112.2, 56.4, 36.1, 24.9, 15.6, 11.2; HRMS (ESI-QTOF) *m*/*z* [M − H]^−^ Found 381.0277 (calcd. 381.0274 for C_16_H_16_^81^BrN_2_O_4_) (NMR spectra, Figures S74–S77).

*5-bromoindolyl-3-glyoxyl-L-serine* (**43**): orange amorphous solid, 10.1 mg; [α]^23^_D_ = +12.5 (c 0.05, MeOH); UV (MeOH) λ_max_ (log ε) 321 (3.6), 273 (3.7), 260 (3.7) and 215 (4.1) nm; IR (FTIR) ν_max_ 3656, 2979, 2888, 1381, 1251, 1152 and 954 cm^−1^; ^1^H NMR (500 MHz, DMSO) δ 12.45 (d, *J* = 2.6 Hz, 1H), 8.83 (d, *J* = 3.2 Hz, 1H), 8.58 (d, *J* = 8.0 Hz, 1H), 8.36 (d, *J* = 1.9 Hz, 1H), 7.53 (d, *J* = 8.5 Hz, 1H), 7.43 (dd, *J* = 8.5, 1.9 Hz, 1H), 4.40 (ddd, *J* = 8.0 Hz, 5.1, 3.6 Hz, 1H), 3.85 (dd, *J* = 11.1, 5.1 Hz, 1H), 3.78 (d, *J* = 11.1, 3.6 Hz, 1H); ^13^C NMR (126 MHz, DMSO) δ 181.2, 171.2, 162.7, 139.7, 135.1, 128.0, 126.2, 123.4, 115.5, 114.8, 111.6, 60.9, 54.8; HRMS (ESI-QTOF) *m*/*z* [M − H]^−^ Found 354.9758 (calcd. 354.9753 for C_13_H_10_^81^BrN_2_O_5_) (NMR spectra, Figures S78–S81).

*6-bromoindolyl-3-glyoxyl-L-serine* (**44**): orange amorphous solid, 12.2 mg; [α]^23^_D_ = +11.7 (c 0.06, MeOH); UV (MeOH) λ_max_ (log ε) 324 (3.9), 276 (4.1), 260 (4.1), and 213 (4.4) nm; IR (FTIR) ν_max_ 3658, 2979, 2886, 1383, 1252, 1153 and 952 cm^−1^; ^1^H NMR (800 MHz, DMSO) δ 12.35 (d, *J* = 3.2 Hz, 1H), 8.81 (d, *J* = 3.2 Hz, 1H), 8.58 (d, *J* = 8.0 Hz, 1H), 8.17 (d, *J* = 8.5 Hz, 1H), 7.75 (d, *J* = 1.8 Hz, 1H), 7.42 (dd, *J* = 8.5, 1.8 Hz, 1H), 4.40 (ddd, *J* = 8.0, 5.1, 3.5 Hz, 1H), 3.85 (dd, *J* = 11.4, 5.1 Hz, 1H), 3.79 (d, *J* = 11.4, 3.5 Hz, 1H); ^13^C NMR (201 MHz, DMSO) δ 181.4, 171.4, 162.6, 139.6, 137.2, 125.5, 125.3, 123.0, 116.0, 115.4, 112.0, 61.1, 54.8; HRMS (ESI-QTOF) *m*/*z* [M − H]^−^ Found 354.9758 (calcd. 354.9753 for C_13_H_10_^81^BrN_2_O_5_) (NMR spectra, Figures S82–S85).

*5-bromoindolyl-3-glyoxyl-L-tryptophan* (**45**): brown amorphous solid, 10.5 mg; [α]^23^_D_ = +21.7 (c 0.05, MeOH); UV (MeOH) λ_max_ (log ε) 318 (4.0), 273 (4.2), 262 (4.2), and 217 (4.6) nm; IR (FTIR) ν_max_ 3656, 3365, 2979, 2888, 1730, 1631, 1498, 1428, 1382, 1232, and 1150 cm^−1^; ^1^H NMR (500 MHz, DMSO) δ 12.42 (d, *J* = 3.2 Hz, 1H), 10.87 (d, *J* = 1.8 Hz, 1H), 8.71 (d, *J* = 8.0 Hz, 1H), 8.64 (d, *J* = 3.2 Hz, 1H), 8.33 (d, *J* = 1.9 Hz, 1H), 7.56 (d, *J* = 7.9 Hz, 1H), 7.51 (d, *J* = 8.5 Hz, 1H), 7.41 (dd, *J* = 8.5, 1.9 Hz, 1H), 7.34 (d, *J* = 8.0 Hz, 1H), 7.19 (d, *J* = 2.3 Hz, 1H), 7.06 (dd*, *J* = 7.5 Hz, 1H), 6.97 (dd*, *J* = 7.5 Hz, 1H), 4.64 (ddd, *J* = 8.5, 7.9, 4.9 Hz, 1H), 3.34 (dd, *J* = 14.5, 4.9 Hz, 1H), 3.28 (dd, *J* = 14.5, 8.5 Hz, 1H); ^13^C NMR (126 MHz, DMSO) δ 181.5, 172.7, 163.0, 139.5, 136.1, 135.1, 127.9, 127.2, 126.1, 123.8, 123.4, 121.0, 118.5, 118.2, 115.4, 114.7, 111.6, 111.5, 109.7, 52.9, 26.5; HRMS (ESI-QTOF) *m*/*z* [M − H]^−^ Found 454.0228 (calcd. 454.0227 for C_21_H_15_^81^BrN_3_O_5_) (NMR spectra, Figures S86–S89).

*5-bromoindolyl-3-glyoxyl-D-tryptophan* (**46**): brown amorphous solid, 15.5 mg; [α]^23^_D_ = −18.5 (c 0.06, MeOH); UV (MeOH) λ_max_ (log ε) 318 (3.8), 275 (3.9), 260 (43.9), and 214 (4.4) nm; IR (FTIR) ν_max_ 3658, 3361, 2980, 2889, 1625, 1461, 1393, 1234, 1150 and 955 cm^−1^; ^1^H NMR (500 MHz, DMSO) δ 12.42 (d, *J* = 3.2 Hz, 1H), 10.86 (d, *J* = 1.8 Hz, 1H), 8.71 (d, *J* = 8.0 Hz, 1H), 8.63 (d, *J* = 3.2 Hz, 1H), 8.31 (d, *J* = 1.9 Hz, 1H), 7.56 (d, *J* = 8.0 Hz, 1H), 7.51 (d, *J* = 8.6 Hz, 1H), 7.41 (dd, *J* = 8.6, 1.9 Hz, 1H), 7.33 (d, *J* = 8.0 Hz, 1H), 7.17 (d, *J* = 2.3 Hz, 1H), 7.05 (ddd*, *J* = 8.0, 7.1, 1.0 Hz, 1H), 6.96 (ddd*, *J* = 8.0, 7.1, 0.9 Hz, 1H), 4.62 (ddd, *J* = 8.6, 8.0, 4.9 Hz, 1H), 3.32 (dd, *J* = 14.9, 4.9 Hz, 1H), 3.26 (dd, *J* = 14.9, 8.6 Hz, 1H); ^13^C NMR (126 MHz, DMSO) δ 181.4, 172.7, 163.0, 139.5, 136.1, 135.1, 127.9, 127.2, 126.1, 123.8, 123.3, 121.0, 118.4, 118.2, 115.4, 114.7, 111.5, 111.5, 109.7, 52.9, 26.4; HRMS (ESI-QTOF) *m*/*z* [M − H]^−^ Found 454.0230 (calcd. 454.0227 for C_21_H_15_^81^BrN_3_O_4_) (NMR spectra, Figures S90–S93).

*6-bromoindolyl-3-glyoxyl-L-tryptophan* (**47**): brown amorphous solid, 6.1 mg; [α]^23^_D_ = +19.3 (c 0.06, MeOH); UV (MeOH) λ_max_ (log ε) 324 (3.8), 277 (4.0) and 215 (4.5) nm; IR (FTIR) ν_max_ 3649, 3229, 2980, 2889, 1722, 1677, 1629, 1495, 1440, 1416, 1242, 1156, 1049, 1024 and 1002 cm^−1^; ^1^H NMR (500 MHz, DMSO) δ 12.31 (d, *J* = 3.2 Hz, 1H), 10.85 (d, *J* = 1.8 Hz, 1H), 8.71 (d, *J* = 8.0 Hz, 1H), 8.61 (d, *J* = 3.2 Hz, 1H), 8.11 (d, *J* = 8.5 Hz, 1H), 7.73 (d, *J* = 1.8 Hz, 1H), 7.56 (d, *J* = 8.0 Hz, 1H), 7.39 (dd, *J* = 8.5 and 1.8 Hz, 1H), 7.33 (d, *J* = 8.0 Hz, 1H), 7.17 (d, *J* = 2.3 Hz, 1H), 7.06 (ddd*, *J* = 7.9, 7.1, 1.0 Hz, 1H), 6.96 (ddd*, *J* = 7.9, 7.1, 1.0 Hz, 1H), 4.62 (ddd, *J* = 8.3, 8.0, 4.8 Hz, 1H), 3.32 (dd, *J* = 14.9, 4.8 Hz, 1H), 3.26 (dd, *J* = 14.9, 8.3 Hz, 1H); ^13^C NMR (126 MHz, DMSO) δ 181.6, 172.7, 163.1, 139.3, 137.2, 136.1, 127.2, 125.5, 125.1, 123.8, 122.9, 121.0, 118.4, 118.2, 116.0, 115.3, 112.0, 111.5, 109.7, 52.9, 26.5; HRMS (ESI-QTOF) *m*/*z* [M − H]^−^ Found 454.02330 (calcd. 454.0227 for C_21_H_15_^81^BrN_3_O_5_) (NMR spectra, Figures S94–S97).

*6-bromoindolyl-3-glyoxyl-D-tryptophan* (**48**): brown amorphous solid, 8.3 mg; [α]^23^_D_ = −23.4 (c 0.04, MeOH); UV (MeOH) λ_max_ (log ε) 323 (3.6), 276 (3.8) and 215 (4.3) nm; IR (FTIR) ν_max_ 3658, 3358, 2979, 2889, 1625, 1461, 1381, 1234, 1150 and 955 cm^−1^; ^1^H NMR (500 MHz, DMSO) δ 12.32 (d, *J* = 3.2 Hz, 1H), 10.86 (d, *J* = 1.8 Hz, 1H), 8.71 (d, *J* = 8.0 Hz, 1H), 8.62 (d, *J* = 3.2 Hz, 1H), 8.11 (d, *J* = 8.5 Hz, 1H), 7.73 (d, *J* = 1.8 Hz, 1H), 7.56 (d, *J* = 8.0 Hz, 1H), 7.39 (dd, *J* = 8.5, 1.8 Hz, 1H), 7.34 (d, *J* = 8.0 Hz, 1H), 7.18 (d, *J* = 2.3 Hz, 1H), 7.06 (ddd*, *J* = 8.1, 7.3, 1.0 Hz, 1H), 6.96 (ddd*, *J* = 8.1, 7.3, 1.0 Hz, 1H), 4.63 (ddd, *J* = 8.3, 8.0, 4.8 Hz, 1H), 3.33 (dd, *J* = 14.9, 4.8 Hz, 1H), 3.27 (dd, *J* = 14.9, 8.3 Hz, 1H); ^13^C NMR (126 MHz, DMSO) δ 181.6, 172.7, 163.1, 139.3, 137.2, 136.1, 127.2, 125.5, 125.1, 123.8, 122.9, 121.0, 118.4, 118.2, 116.0, 115.3, 112.0, 111.4, 109.7, 52.9, 26.4; HRMS (ESI-QTOF) *m*/*z* [M − H]^−^ Found 454.0223 (calcd. 454.0227 for C_21_H_15_^81^BrN_3_O_4_) (NMR spectra, Figures S98–S101).

*5-bromoindolyl-3-glyoxyl-L-tyrosine* (**49**): brown amorphous solid, 4.7 mg; [α]^23^_D_ = +28.9 (c 0.04, MeOH); UV (MeOH) λ_max_ (log ε) 322 (3.7), 275 (3.8), 261 (3.8) and 213 (4.3) nm; IR (FTIR) ν_max_ 3656, 2980, 2888, 1382, 1251, 1151, 1072 and 954 cm^−1^; ^1^H NMR (500 MHz, DMSO) δ 12.42 (d, *J* = 3.2 Hz, 1H), 9.22 (s, 1H), 8.72 (d, *J* = 8.2 Hz, 1H), 8.61 (d, *J* = 3.2 Hz, 1H), 8.32 (d, *J* = 1.9 Hz, 1H), 7.51 (d, *J* = 8.6 Hz, 1H), 7.41 (dd, *J* = 8.6, 1.9 Hz, 1H), 7.04 (d, *J* = 8.3 Hz, 2H), 6.64 (d, *J* = 8.3 Hz, 2H), 4.50 (ddd, *J* = 9.4, 8.2, 4.7 Hz, 1H), 3.07 (dd, *J* = 14.1, 4.7 Hz, 1H), 2.99 (dd, *J* = 14.1, 9.4 Hz, 1H); ^13^C NMR (126 MHz, DMSO) δ 181.5, 172.4, 163.0, 156.0, 139.5, 135.1, 130.1, 127.9, 127.5, 126.2, 123.4, 115.5, 115.1, 114.8, 111.6, 53.7, 35.3; HRMS (ESI-QTOF) *m*/*z* [M − H]^−^ Found 431.0065 (calcd. 431.0066 for C_19_H_14_^81^BrN_2_O_5_) (NMR spectra, Figures S102–S105).

*5-bromoindolyl-3-glyoxyl-D-tyrosine* (**50**): brown amorphous solid, 10.1 mg; [α]^23^_D_ = −31.3 (c 0.06, MeOH); UV (MeOH) λ_max_ (log ε) 322 (3.9), 275 (4.0), 261 (4.0) and 214 (4.5) nm; IR (FTIR) ν_max_ 3657, 3249, 2980, 2888, 1632, 1382, 1251, 1151 and 954 cm^−1^; ^1^H NMR (500 MHz, DMSO) δ 12.42 (d, *J* = 3.2 Hz, 1H), 9.22 (br s, 1H), 8.72 (d, *J* = 8.2 Hz, 1H), 8.63 (d, *J* = 3.2 Hz, 1H), 8.33 (d, *J* = 1.9 Hz, 1H), 7.51 (d, *J* = 8.6 Hz, 1H), 7.41 (dd, *J* = 8.6, 1.9 Hz, 1H), 7.05 (d, *J* = 8.3 Hz, 2H), 6.65 (d, *J* = 8.3 Hz, 2H), 4.51 (ddd, *J* = 9.4, 8.2, 4.7 Hz, 1H), 3.08 (dd, *J* = 14.0, 4.7 Hz, 1H), 3.00 (dd, *J* = 14.0, 9.4 Hz, 1H); ^13^C NMR (126 MHz, DMSO) δ 181.6, 172.3, 162.8, 155.9, 139.5, 135.1, 130.1, 127.9, 127.6, 126.1, 123.4, 115.4, 115.0, 114.7, 111.6, 53.8, 35.3; HRMS (ESI-QTOF) *m*/*z* [M − H]^−^ Found 431.0069 (calcd. 431.0066 for C_19_H_14_^81^BrN_2_O_5_) (NMR spectra, Figures S106–S109).

*6-bromoindolyl-3-glyoxyl-L-tyrosine* (**51**): brown amorphous solid, 6.1 mg; [α]^23^_D_ = +33.7 (c 0.06, MeOH); UV (MeOH) λ_max_ (log ε) 321 (3.9), 276 (4.2), 260 (4.1) and 212 (4.5) nm; IR (FTIR) ν_max_ 3654, 3249, 2980, 2888, 1631, 1382, 1249, 1153 and 955 cm^−1^; ^1^H NMR (500 MHz, DMSO) δ 12.33 (d, *J* = 3.2 Hz, 1H), 9.23 (br s, 1H), 8.73 (d, *J* = 8.2 Hz, 1H), 8.59 (d, *J* = 3.2 Hz, 1H), 8.12 (d, *J* = 8.5 Hz, 1H), 7.73 (d, *J* = 1.8 Hz, 1H), 7.40 (dd, *J* = 8.5 and 1.8 Hz, 1H), 7.05 (d, *J* = 8.4 Hz, 2H), 6.65 (d, *J* = 8.4 Hz, 2H), 4.51 (ddd, *J* = 9.3, 8.2, 4.6 Hz, 1H), 3.08 (dd, *J* = 14.0, 4.6 Hz, 1H), 2.98 (dd, *J* = 14.0, 9.3 Hz, 1H); ^13^C NMR (126 MHz, DMSO) δ 181.7, 172.4, 163.1, 156.0, 139.2, 137.3, 130.1, 127.5, 125.5, 125.1, 122.9, 116.0, 115.4, 115.1, 112.1, 53.7, 35.3; HRMS (ESI-QTOF) *m*/*z* [M − H]^−^ Found 431.0064 (calcd. 431.0066 for C_19_H_14_^81^BrN_2_O_5_) (NMR spectra, Figures S110–S113).

*6-bromoindolyl-3-glyoxyl-D-tyrosine* (**52**): brown amorphous solid, 6.2 mg; [α]^23^_D_ = −35.8 (c 0.06, MeOH); UV (MeOH) λ_max_ (log ε) 323 (3.9), 277 (4.1), 260 (4.0) and 212 (4.5) nm; IR (FTIR) ν_max_ 3658, 3345, 2980, 2888, 1630, 1382, 1250, 1153 and 955 cm^−1^; ^1^H NMR (500 MHz, DMSO) δ 12.32 (d, *J* = 3.2 Hz, 1H), 8.73 (d, *J* = 8.2 Hz, 1H), 8.59 (d, *J* = 3.2 Hz, 1H), 8.12 (d, *J* = 8.5 Hz, 1H), 7.73 (d, *J* = 1.8 Hz, 1H), 7.40 (dd, *J* = 8.5 and 1.8 Hz, 1H), 7.04 (d, *J* = 8.4 Hz, 2H), 6.65 (d, *J* = 8.4 Hz, 2H), 4.50 (ddd, *J* = 9.3, 8.2, 4.6 Hz, 1H), 3.07 (dd, *J* = 13.8, 4.6 Hz, 1H), 2.98 (dd, *J* = 13.9, 9.3 Hz, 1H); ^13^C NMR (126 MHz, DMSO) δ 181.7, 172.5, 163.1, 156.0, 139.2, 137.3, 130.1, 127.5, 125.5, 125.1, 122.9, 116.1, 115.4, 115.1, 112.1, 53.7, 35.3; HRMS (ESI-QTOF) *m*/*z* [M − H]^−^ Found 431.0071 (calcd. 431.0066 for C_19_H_14_^81^BrN_2_O_5_) (NMR spectra, Figures S114–S117).

*5-bromoindolyl-3-glyoxyl-L-valine* (**53**): brown amorphous solid, 5.0 mg; [α]^23^_D_ = +18.9 (c 0.05, MeOH); UV (MeOH) λ_max_ (log ε) 323 (4.0), 273 (4.1), 262 (4.1) and 213(4.4) nm; IR (FTIR) ν_max_ 3653, 3267, 2980, 2888, 1723, 1632, 1392, 1233, 1152 and 966 cm^−1^; ^1^H NMR (500 MHz, DMSO) δ 12.46 (br s, 1H), 8.68 (s, 1H), 8.54 (d, *J* = 8.4 Hz, 1H), 8.34 (d, *J* = 1.9 Hz, 1H), 7.53 (d, *J* = 8.6 Hz, 1H), 7.42 (dd, *J* = 8.6, 1.9 Hz, 1H), 4.24 (dd, *J* = 8.4, 5.3 Hz, 1H), 2.22 (m, 1H), 0.95 (d, *J* = 5.9 Hz, 3H), 0.93 (d, *J* = 5.9 Hz, 3H); ^13^C NMR (126 MHz, DMSO) δ 182.1, 172.3, 163.7, 139.3, 135.2, 127.8, 126.2, 123.3, 115.4, 114.8, 111.8, 57.5, 29.8, 19.2, 18.1; HRMS (ESI-QTOF) *m*/*z* [M − H]^−^ Found 367.0115 (calcd. 367.0117 for C_15_H_14_^81^BrN_2_O_4_) (NMR spectra, Figures S118–S121).

*5-bromoindolyl-3-glyoxyl-D-valine* (**54**): brown amorphous solid, 18.1 mg; [α]^23^_D_ = −21.1 (c 0.06, MeOH); UV (MeOH) λ_max_ (log ε) 323 (4.1), 274 (4.1), 260 (4.1) and 215 (4.5) nm; IR (FTIR) ν_max_ 3655, 3269, 2980, 2889, 1725, 1679, 1634, 1498, 1429, 1392, 1231, 1154 and 967 cm^−1^; ^1^H NMR (500 MHz, DMSO) δ 12.44 (br s, 1H), 8.67 (s, 1H), 8.54 (d, *J* = 8.4 Hz, 1H), 8.34 (d, *J* = 1.9 Hz, 1H), 7.53 (d, *J* = 8.6 Hz, 1H), 7.42 (dd, *J* = 8.6, 1.9 Hz, 1H), 4.23 (dd, *J* = 8.4, 5.8 Hz, 1H), 2.22 (m, 1H), 0.95 (d, *J* = 6.8 Hz, 3H), 0.93 (d, *J* = 6.8 Hz, 3H); ^13^C NMR (126 MHz, DMSO) δ 182.1, 172.3, 163.7, 139.3, 135.2, 127.8, 126.1, 123.3, 115.4, 114.8, 111.7, 57.5, 29.8, 19.2, 18.1; HRMS (ESI-QTOF) *m*/*z* [M − H]^−^ Found 367.0116 (calcd. 367.0117 for C_15_H_14_^81^BrN_2_O_4_) (NMR spectra, Figures S122–S125).

*6-bromoindolyl-3-glyoxyl-L-valine* (**55**): brown amorphous solid, 13.9 mg; [α]^23^_D_ = +17.2 (c 0.06, MeOH); UV (MeOH) λ_max_ (log ε) 324 (3.9), 276 (4.1), 260 (4.1) and 211 (4.5) nm; IR (FTIR) ν_max_ 3657, 3269, 2980, 2888, 1631, 1499, 1440, 1392, 1250, and 1155 and 955 cm^−1^; ^1^H NMR (500 MHz, DMSO) δ 12.34 (d, *J* = 3.2 Hz, 1H), 8.64 (d, *J* = 3.2 Hz, 1H), 8.56 (d, *J* = 8.4 Hz, 1H), 8.14 (d, *J* = 8.4 Hz, 1H), 7.75 (d, *J* = 1.8 Hz, 1H), 7.41 (dd, *J* = 8.4, 1.8 Hz, 1H), 4.25 (dd, *J* = 8.4, 5.9 Hz, 1H), 2.21 (m, 1H), 0.95 (d, *J* = 6.9 Hz, 3H), 0.93 (d, *J* = 6.9 Hz, 3H); ^13^C NMR (126 MHz, DMSO) δ 182.2, 172.4, 163.9, 139.2, 137.4, 125.6, 125.1, 122.9, 116.1, 115.4, 112.3, 57.5, 29.7, 19.2, 18.1; HRMS (ESI-QTOF) *m*/*z* [M − H]^−^ Found 367.0118 (calcd. 367.0117 for C_15_H_14_^81^BrN_2_O_4_) (NMR spectra, Figures S126–S129).

*6-bromoindolyl-3-glyoxyl-D-valine* (**56**): brown amorphous solid, 16.8 mg; [α]^23^_D_ = −22.9 (c 0.06, MeOH); UV (MeOH) λ_max_ (log ε) 323 (4.0), 274 (4.2), 260 (4.2) and 213 (4.6) nm; IR (FTIR) ν_max_ 3656, 3229, 2980, 2888, 1632, 1499, 1440, 1392, 1250, and 1156 cm^−1^; ^1^H NMR (500 MHz, DMSO) δ 12.35 (d, *J* = 3.2 Hz, 1H), 8.66 (d, *J* = 3.2 Hz, 1H), 8.55 (d, *J* = 8.4 Hz, 1H), 8.15 (d, *J* = 8.5 Hz, 1H), 7.75 (d, *J* = 1.8 Hz, 1H), 7.41 (dd, *J* = 8.5, 1.8 Hz, 1H), 4.26 (dd, *J* = 8.4, 5.9 Hz, 1H), 2.22 (m, 1H), 0.95 (d, *J* = 6.8 Hz, 3H), 0.94 (d, *J* = 6.8 Hz, 3H); ^13^C NMR (126 MHz, DMSO) δ 182.2, 172.3, 163.9, 139.1, 137.3, 125.5, 125.0, 122.8, 116.0, 115.4, 112.2, 57.4, 29.7, 19.1, 18.1; HRMS (ESI-QTOF) *m*/*z* [M − H]^−^ Found 367.0114 (calcd. 367.0117 for C_15_H_14_^81^BrN_2_O_4_) (NMR spectra, Figures S130–S133).

### 4.3. Cheminformatics Analyses of Marine and Synthetic Indole Chemical Diversity

The structures for 2048 MIAs reported in the MarinLit database (up to the end of 2021) were imported into the freely available cheminformatics software Osiris DataWarrior (version 6.01.05) [8,14]. The reported biological activities were manually obtained from biological testing data reported in the primary literature for the 2048 compounds, which were scaled to disease and infection targets and potency of activity classifications in Table S2 [3]. The chemical diversity of MIAs, bioactive synthetic IGAs (*n* = 147), and the synthetic IGAs **25**–**56** (*n* = 32) was visualised using a 50 × 50 neuron self-organising map (SOM) and the SkeletonSpheres chemical descriptor (1024 bin resolution non-binary byte array/vector).

### 4.4. In Vitro α-Synuclein MS-Binding Assay

α-synuclein protein solutions (10 μM) were prepared in 10 mM ammonium acetate. Synthetic IGAs **25**, **26**, **34**, **41**, **45**, **46**, **51**, and **52** (100 μM) were dissolved in 100% MeOH and incubated with α-syn at room temperature for 3 h. Protein-ligand interactions were analysed using (+) HRESIMS calibrated with sodium trifluoroacetate (0.1 mg/mL) with the following parameters: nebuliser 2.0 Bar; dry gas 7 L/min; dry temp 150 °C; funnel I RF 400 Vpp; end plate offset 500 V; capillary 4500 V; ISCID 0.0 ev; ion energy 4.0 eV; transfer time 100 μs; multipole RF 800 Vpp; collision RF 1200 Vpp; prepulse storage 10 μs; spectra rate: 2 × 100 Hz. The resulting spectra were analysed for additional peaks representing complexes formed between α-syn and active compounds.

### 4.5. Antiplasmodial Image-Based Assay

An anti-plasmodial imaging assay reported by Duffy and Avery [45] was used to examine the antiplasmodial activities of synthetic indoles **25**, **26**, **36**, **37**, **38**, **40**, **41**, **43**, **45**, **46**, **47**, **48**, **50** and **51**. All compounds were diluted to 60 μM in DMSO and tested at three increasing concentrations from 0.4 nM–40 μM per log dose in a 16-point CRC (N = 2) in full dose-response against chloroquine-resistant (3D7) and sensitive (Dd2) parasite strains of *Plasmodium falciparum*. Dihydroartemisinin (DHA), chloroquine, pyrimethamine, and puromycin were used as controls in addition to 0.4% DMSO. Normalised percent inhibition data was obtained using in-plate positive (4 μM DHA) and negative (0.4% DMSO) controls, which were then used to calculate IC_50_ values. Compounds were incubated in CellCarrier-384 well imaging plates in the presence of 2% parasitemia (3D7 or Dd2) at 0.3% hematocrit in a total assay volume of 50 μL for 72 h at 37 °C, 5% O_2_, 5% CO_2_ and 90% N_2_. After incubation, plates were stained with 4′,6-diamidino-2-phenylindole (DAPI) and imaged using an Opera Phenix high-content imaging system (Revvity, Mulgrave, Victoria, Australia). Images were analysed using Harmony (Revvity) software (version 3), while percent parasite inhibition was calculated using the minimum (0.4% DMSO) and (DHA). IC_50_ values were determined by plotting % inhibition against log dose and analysed using nonlinear regression and sigmoidal dose-response using GraphPad Prism version 6.

### 4.6. SARS-CoV-2 3CL^pro^ Inhibition Assays

The assays for measuring inhibition of SARS-CoV-2 3CL^pro^ have been previously reported [31]. Reactions contained 0.01% Triton X100 to minimise compound and enzyme aggregation [46] and were performed with and without 4 mM DTT [32]. All protease assays were performed in 96-well plates in 50 mM Tris, 1 mM EDTA in 200 µL total at pH 7.5. Each well contained 25 nM of SARS-CoV-2 3CL^Pro^ and eCFP-Venus biosensor (500 nM). After the sequential addition of SARS-CoV-2 3CL^Pro^, buffer (100 µL) and compounds **25**–**56** to each well, the assay was initiated by adding eCFP-Venus biosensor (100 µL). The plates were incubated at 30 °C for 4 h, with fluorescence measured using an excitation wavelength of 434 nm, and emission wavelengths of 477 and 528 nm, respectively. HTS was performed in duplicate using 5 µM of each compound. Z’ score was calculated using the sample means and standard deviations for positive and negative controls. The dose–response for selected hits was performed using a concentration ranging from 0.001 µM to 20 µM and included a sample with no added compound. All data processing was performed in R (version 4.0.2).

### 4.7. Serine Protease Assays

Serine protease-inhibitory activities for **25**–**56** were determined in 96 well plates. Each inhibitor was initially tested in duplicate against elastase and chymotrypsin at 10 µM. Where inhibitory activity was observed at this concentration, further experiments with multiple dilutions of the inhibitor were undertaken to determine the IC_50_. Assays for the inhibition of elastase were undertaken using high-purity porcine pancreatic elastase (Sigma, Melbourne, Victoria, Australia, E0258) in an assay buffer of 50 mM Tris-HCl (pH 8.0) at room temperature. Dilutions of each inhibitor (originally resuspended in DMSO) were prepared in triplicate and then preincubated with 375 ng of elastase for 15 min at room temperature in a total reaction volume of 85 µL. Following this preincubation, 15 µL of 2 mM N-succinyl-Ala-Ala-Ala-p-nitroanilide (Sigma, S4760) in assay buffer was added to each well and kinetic measurement of absorbance at 405 nm (Tecan Spark Plate Reader, Zürich, Switzerland) was undertaken for 30 min. Assays for the inhibition of chymotrypsin were undertaken at 37 °C using α-chymotrypsin from bovine pancreas (Sigma, C4129) in an assay buffer of 50 mM Tris-HCl, 100 mM NaCl, and 1 mM CaCl_2_ (pH 7.8). Dilutions of each inhibitor were prepared in triplicate and preincubated with 5 µg of chymotrypsin for 10 min at 37 °C in a total reaction volume of 75 µL. After preincubation, 25 µL of 2 mM N-succinyl-Gly-Gly-Phe-p-nitroanilide (Sigma, S1899) was added to each well and kinetic measurement of absorbance at 405 nm was undertaken for 30 min. Inhibitory activity was determined by comparison of the slope of the reaction curve for multiple dilutions of the inhibitor to that of an equivalent solvent control and expressed as IC_50_ ± standard deviation.

### 4.8. Human Cancer Cell Cytotoxicity Assay

Synthetic **25**–**42** and **44**–**56** (prepared at 20 mM stock concentration in DMSO and a 14 pt CRC) and controls 0.4% DMSO/10 µM puromycin were added 24 h after cell seeding and incubated for 72 h. After 66 h, resazurin was added to a final concentration of 60 µM. Fluorescence was monitored (excitation and emission wavelengths 530 and 590 nm, respectively) using a Tecan Spark Plate Reader (Zürich, Switzerland) at 72 h. Data was normalised to DMSO and puromycin controls (10 µM). HCT-116 cells were cultured in McCoy’s 5A media supplemented with 10% heat-inactivated FBS. For this assay, 500 cells/well were seeded into Greiner black-wall, clear-bottom 384-well cell culture plates. MDA-MB-231 cells were cultured in DMEM media supplemented with 10% heat-inactivated FBS and 10 mM HEPES and 2000 cells/well seeded into Greiner black wall, clear bottom 384 well cell culture plates for compound evaluation. OVCAR8 cells were cultured in RPMI media supplemented with 10% heat-inactivated FBS, and 400 cells/well seeded into Greiner black wall, clear bottom 384 well cell culture plates used for the assay.

## 5. Conclusions

This work highlights the value of orthogonal approaches, including meta-analyses of NP bioactivities, cheminformatics tools, and NP synthesis, in expanding our knowledge of NP bioactive chemical space. Incorporating bioactivity data obtained from synthetic libraries of related structures has also been insightful in directing the choice of screening targets for marine NP-inspired IGAs. The new bioactivities obtained herein for **25**–**56**, amyloid protein binding, antiplasmodial, and protease inhibitory activities, support more directed NP investigations that aim to broaden the scope of NP biological testing. Additionally, we have outlined synthetic approaches that explore further structural modification of marine IGA scaffolds to improve their potential applications toward several disease and infection targets. By exploring large NP databases with cheminformatics tools alongside the inclusion of functional assays, we can enhance the impact of NPs and NP scaffolds for future drug discovery and development.

## Figures and Tables

**Table 1:** Synthetic 5 and 6-brominated indol-3-yl-glyoxylamides **25**–**56**.

Compound	R_1_	R_2_	R_3_	Compound	R_1_	R_2_	R_3_
**25**	Br	H	L-alanine	**41**	H	Br	L-glutamic acid
**26**	Br	H	D-alanine	**42**	H	Br	D-glutamic acid
**27**	H	Br	L-alanine	**43**	Br	H	L-serine
**28**	H	Br	D-alanine	**44**	H	Br	L-serine
**29**	Br	H	L-arginine	**45**	Br	H	L-tryptophan
**30**	Br	H	D-arginine	**46**	Br	H	D-tryptophan
**31**	H	Br	L-arginine	**47**	H	Br	L-tryptophan
**32**	H	Br	D-arginine	**48**	H	Br	D-tryptophan
**33**	Br	H	L-cysteine	**49**	Br	H	L-tyrosine
**34**	H	Br	L-cysteine	**50**	Br	H	D-tyrosine
**35**	Br	H	L-histidine	**51**	H	Br	L-tyrosine
**36**	H	Br	L-histidine	**52**	H	Br	D-tyrosine
**37**	Br	H	L-isoleucine	**53**	Br	H	L-valine
**38**	H	Br	L-isoleucine	**54**	Br	H	D-valine
**39**	Br	H	L-glutamic acid	**55**	H	Br	L-valine
**40**	Br	H	D-glutamic acid	**56**	H	Br	D-valine

**Table 2:** Antiplasmodial activities against *P. falciparum* parasite strains 3D7 and Dd2 and HEK293 cell cytotoxicity for selected indol-3-yl-glyoxylamide analogues.

	% Inhibition @ 60 µM (IC_50_)					
Compound	R_1_	R_2_	R_3_	3D7 ^a^	Dd2 ^b^	HEK293 ^c^
**25**	H	Br	L-Arg	1.0	6.5	18.5
**26**	H	Br	D-Arg	56.0	33.0	14.5
**36**	H	Br	L-His	7.0	15.0	14.5
**37**	Br	H	L-Ile	11.5	9.0	15.0
**38**	H	Br	L-Ile	23.5	4.5	14.5
**40**	Br	H	D-Glu	2.0	2.5	18.5
**41**	H	Br	L-Glu	6.0	1.0	22.0
**43**	Br	H	L-Ser	1.0	1.0	21.0
**45**	Br	H	L-Trp	73.0	56.0	24.0
**46**	Br	H	D-Trp	100.0 (7.4 µM)	97.5 (8.2 µM)	21.5 (IA @ 60 µM)
**47**	H	Br	L-Trp	14.0	20.0	14.0
**48**	H	Br	D-Trp	99.0	97.5	16.0
**50**	Br	H	D-Tyr	99.0	97.5	20.5
**51**	H	Br	L-Tyr	58.0	56.0	21.5
Dihydroartemisinin	-	-	-	100.0 (1.3 nM)	100.0 (1.7 nM)	IA @ 2 µM
Puromycin	-	-	-	(79.2 nM)	(86.5 nM)	(673 nM)
Chloroquine	-	-	-	(77.8 nM)	(695.5 nM)	IA @ 10 µM
Pyrimethamine	-	-	-	−10.0 (14.1 nM)	IA @ 10 µM	IA @ 10 µM

^a^ 3D7 = chloroquine-sensitive *P. falciparum* strain, ^b^ Dd2 = chloroquine-resistant *P. falciparum* strain, ^c^ Human embryonic kidney cell line.

## Data Availability

Supporting data are available in the Electronic Supporting Information (ESI) provided.

## Supplementary Materials

The following supporting information can be downloaded at: https://www.mdpi.com/article/10.3390/molecules29153648/s1, Table S1; Marine indol-3-yl-glyoxylamides **1**–**24**, Figure S1; Marine indol-3-yl-glyoxylamides **1**–**24** reported to end of 2021 (MarinLit database), Figure S2; Background topography of marine indole alkaloid chemical diversity visualised in a self-organising map, Figure S3; Chemical diversity of marine indole alkaloids by producing phylum visualised in a self-organising map, Figure S3; marine indol-3-yl-glyoxylamides **1**–**24**, Figures S4 and S5; synthetic library of indol-3-yl-glyoxylamides **25**–**56**, Table S2; Potency of bioactivity criteria for categorising potency of bioactivity relevant to drug discovery, Table S3; Inhibition of SARS-CoV-2 3CL^PRO^ at 20 µM for **25**–**56,** Table S4; Inhibition of mammalian serine proteases (elastase and chymotrypsin) for **25**–**56**, Figures S6–S133; NMR spectra for **25**–**56**, Figures S134–S140; α-synuclein MS binding assay results (**25**, **26**, **34**, **37**, **45**, **46**, **51**, and **52**), Figure S141; Dose-response curves for synthetic indoles **46**, **48**, **50**, and **51** against chloroquine-sensitive (3D7) and -resistant (Dd2) *Plasmodium falciparum* parasite strains and a human embryonic cell line (HEK293).
