# Supplementary material for: Cheminformatics-Guided Exploration of Synthetic Marine Natural Product-Inspired Brominated Indole-3-Glyoxylamides and Their Potentials for Drug Discovery
Source: Molecules. 2024 Aug 1;29(15):3648. doi: 10.3390/molecules29153648 (PMC11314621; doi:10.3390/molecules29153648)
Supplement: Supplementary file 1 [file molecules-29-03648-s001.zip › molecules-3098138-SI.pdf]

Supporting Information

Cheminformatics-Guided Exploration of Synthetic Marine Natural Product-Inspired Brominated Indole-3-Glyoxylamides and Their Potentials for Drug Discovery

1. Supporting Information Contents

**Table S1**	Marine indol-3-yl-glyoxylamides **1-24** (SMILES, Compound name, Phylum, Genus, Species, and year isolated) reported in MarinLit to the end of 2021.
**Figure S1**	Marine indol-3-yl-glyoxylamides **1-24** reported to end of 2021 (MarinLit database).
**Figure S2**	Background topography of marine indole alkaloid chemical diversity visualized in a self-organizing map (50 x 50 neuron, SkelSpheres chemical descriptor).
**Figure S3**	Chemical diversity of marine indole alkaloid chemical diversity (n = 2048) by producing phylum visualized in a self-organizing map (50 x 50 neuron, SkelSpheres chemical descriptor).
**Figure S4**	Synthetic library of indol-3-yl-glyoxylamides **25-46**.
**Figure S5**	Synthetic library of indol-3-yl-glyoxylamides **47-56**.
**Table S2**	Potency of bioactivity criteria used for categorising NP potency relevant to drug discovery.
**Table S3**	Inhibition of SARS-CoV-2 $3CL^{PRO}$ at 20 μM for **25-56**.
**Table S4**	Inhibition of mammalian serine proteases (elastase and chymotrypsin) for **25-56**.
**Figure S6**	^{1}H NMR spectrum for 5-bromoindolyl-3-glyoxyl-L-alanine (**25**) recorded in DMSO-d_6
**Figure S7**	^{13}C NMR spectrum for 5-bromoindolyl-3-glyoxyl-L-alanine (**25**) recorded in DMSO-d_6
**Figure S8**	HSQC NMR spectrum 5-bromoindolyl-3-glyoxyl-L-alanine (**25**) recorded in DMSO-d_6
**Figure S9**	COSY NMR spectrum 5-bromoindolyl-3-glyoxyl-L-alanine (**25**) recorded in DMSO-d_6
**Figure S10**	^{1}H NMR spectrum for 5-bromoindolyl-3-glyoxyl-D-alanine (**26**) recorded in DMSO-d_6
**Figure S11**	^{13}C NMR spectrum for 5-bromoindolyl-3-glyoxyl-D-alanine (**26**) recorded in DMSO-d_6
**Figure S12**	HSQC NMR spectrum 5-bromoindolyl-3-glyoxyl-D-alanine (**26**) recorded in DMSO-d_6
**Figure S13**	COSY NMR spectrum 5-bromoindolyl-3-glyoxyl-D-alanine (**26**) recorded in DMSO-d_6
**Figure S14**	^{1}H NMR spectrum for 6-bromoindolyl-3-glyoxyl-L-alanine (**27**) recorded in DMSO-d_6
**Figure S15**	^{13}C NMR spectrum for 6-bromoindolyl-3-glyoxyl-L-alanine (**27**) recorded in DMSO-d_6
**Figure S16**	HSQC NMR spectrum 6-bromoindolyl-3-glyoxyl-L-alanine (**27**) recorded in DMSO-d_6
**Figure S17**	COSY NMR spectrum 6-bromoindolyl-3-glyoxyl-L-alanine (**27**) recorded in DMSO-d_6
**Figure S18**	^{1}H NMR spectrum for 6-bromoindolyl-3-glyoxyl-D-alanine (**28**) recorded in DMSO-d_6
**Figure S19**	^{13}C NMR spectrum for 6-bromoindolyl-3-glyoxyl-D-alanine (**28**) recorded in DMSO-d_6
**Figure S20**	HSQC NMR spectrum 6-bromoindolyl-3-glyoxyl-D-alanine (**28**) recorded in DMSO-d_6
**Figure S21**	COSY NMR spectrum 6-bromoindolyl-3-glyoxyl-D-alanine (**28**) recorded in DMSO-d_6
**Figure S22**	^{1}H NMR spectrum for 5-bromoindolyl-3-glyoxyl-L-arginine (**29**) recorded in DMSO-d_6
**Figure S23**	^{13}C NMR spectrum for 5-bromoindolyl-3-glyoxyl-L-arginine (**29**) recorded in DMSO-d_6
**Figure S24**	HSQC NMR spectrum 5-bromoindolyl-3-glyoxyl-L-arginine (**29**) recorded in DMSO-d_6
**Figure S25**	COSY NMR spectrum 5-bromoindolyl-3-glyoxyl-L-arginine (**29**) recorded in DMSO-d_6
**Figure S26**	^{1}H NMR spectrum for 5-bromoindolyl-3-glyoxyl-D-arginine (**30**) recorded in DMSO-d_6
**Figure S27**	^{13}C NMR spectrum for 5-bromoindolyl-3-glyoxyl-D-arginine (**30**) recorded in DMSO-d_6
**Figure S28**	HSQC NMR spectrum 5-bromoindolyl-3-glyoxyl-D-arginine (**30**) recorded in DMSO-d_6

**Figure S29**	COSY NMR spectrum 5-bromoindolyl-3-glyoxyl-D-arginine (**30**) recorded in DMSO-d_6
**Figure S30**	^{1}H NMR spectrum for 6-bromoindolyl-3-glyoxyl-L-arginine (**31**) recorded in DMSO-d_6
**Figure S31**	^{13}C NMR spectrum for 6-bromoindolyl-3-glyoxyl-L-arginine (**31**) recorded in DMSO-d_6
**Figure S32**	HSQC NMR spectrum 6-bromoindolyl-3-glyoxyl-L-arginine (**31**) recorded in DMSO-d_6
**Figure S33**	COSY NMR spectrum 6-bromoindolyl-3-glyoxyl-L-arginine (**31**) recorded in DMSO-d_6
**Figure S34**	^{1}H NMR spectrum for 6-bromoindolyl-3-glyoxyl-D-arginine (**32**) recorded in DMSO-d_6
**Figure S35**	^{13}C NMR spectrum for 6-bromoindolyl-3-glyoxyl-D-arginine (**32**) recorded in DMSO-d_6
**Figure S36**	HSQC NMR spectrum 6-bromoindolyl-3-glyoxyl-D-arginine (**32)** recorded in DMSO-d_6
**Figure S37**	COSY NMR spectrum 6-bromoindolyl-3-glyoxyl-D-arginine (**32**) recorded in DMSO-d_6
**Figure S38**	^{1}H NMR spectrum for 5-bromoindolyl-3-glyoxyl-L-cystine dimer (**33**) recorded in DMSO-d_6
**Figure S39**	^{13}C NMR spectrum for 5-bromoindolyl-3-glyoxyl-L-cystine dimer (**33**) recorded in DMSO-d_6
**Figure S40**	HSQC NMR spectrum 5-bromoindolyl-3-glyoxyl-L-cystine dimer (**33**)recorded in DMSO-d_6
**Figure S41**	COSY NMR spectrum 5-bromoindolyl-3-glyoxyl-L-cystine dimer (**33**)recorded in DMSO-d_6
**Figure S42**	^{1}H NMR spectrum for 6-bromoindolyl-3-glyoxyl-L-cystine dimer (**34**) recorded in DMSO-d_6
**Figure S43**	^{13}C NMR spectrum for 6-bromoindolyl-3-glyoxyl-L-cystine dimer (**34**) recorded in DMSO-d_6
**Figure S44**	HSQC NMR spectrum 6-bromoindolyl-3-glyoxyl-L-cystine dimer (**34**) recorded in DMSO-d_6
**Figure S45**	COSY NMR spectrum 6-bromoindolyl-3-glyoxyl-L-cystine dimer (**34**) recorded in DMSO-d_6
**Figure S46**	^{1}H NMR spectrum for 5-bromoindolyl-3-glyoxyl-L-glutamic acid (**35**) recorded in DMSO-d_6
**Figure S47**	^{13}C NMR spectrum for 5-bromoindolyl-3-glyoxyl-L-glutamic acid (**35**) recorded in DMSO-d_6
**Figure S48**	HSQC NMR spectrum 5-bromoindolyl-3-glyoxyl-L-glutamic acid (**35**) recorded in DMSO-d_6
**Figure S49**	COSY NMR spectrum 5-bromoindolyl-3-glyoxyl-L-glutamic acid (**35**) recorded in DMSO-d_6
**Figure S50**	^{1}H NMR spectrum for 5-bromoindolyl-3-glyoxyl-D-glutamic acid (**36**) recorded in DMSO-d_6
**Figure S51**	^{13}C NMR spectrum for 5-bromoindolyl-3-glyoxyl-D-glutamic acid (**36**) recorded in DMSO-d_6
**Figure S52**	HSQC NMR spectrum 5-bromoindolyl-3-glyoxyl-D-glutamic acid (**36**) recorded in DMSO-d_6
**Figure S53**	COSY NMR spectrum 5-bromoindolyl-3-glyoxyl-D-glutamic acid (**36**) recorded in DMSO-d_6
**Figure S54**	^{1}H NMR spectrum for 6-bromoindolyl-3-glyoxyl-L-glutamic acid (**37**) recorded in DMSO-d_6
**Figure S55**	^{13}C NMR spectrum for 6-bromoindolyl-3-glyoxyl-L-glutamic acid (**37**) recorded in DMSO-d_6
**Figure S56**	HSQC NMR spectrum 6-bromoindolyl-3-glyoxyl-L-glutamic acid (**37**) recorded in DMSO-d_6
**Figure S57**	COSY NMR spectrum 6-bromoindolyl-3-glyoxyl-L-glutamic acid (**37**) recorded in DMSO-d_6
**Figure S58**	^{1}H NMR spectrum for 6-bromoindolyl-3-glyoxyl-D-glutamic acid (**38**) recorded in DMSO-d_6
**Figure S59**	^{13}C NMR spectrum for 6-bromoindolyl-3-glyoxyl-D-glutamic acid (**38**) recorded in DMSO-d_6
**Figure S60**	HSQC NMR spectrum 6-bromoindolyl-3-glyoxyl-D-glutamic acid (**38**) recorded in DMSO-d_6
**Figure S61**	COSY NMR spectrum 6-bromoindolyl-3-glyoxyl-D-glutamic acid (**38**) recorded in DMSO-d_6
**Figure S62**	^{1}H NMR spectrum for 6-bromoindolyl-3-glyoxyl-L-histidine (**39**) recorded in DMSO-d_6
**Figure S63**	^{13}C NMR spectrum for 6-bromoindolyl-3-glyoxyl-L-histidine (**39**) recorded in DMSO-d_6
**Figure S64**	HSQC NMR spectrum 6-bromoindolyl-3-glyoxyl-L-histidine (**39**) recorded in DMSO-d_6
**Figure S65**	COSY NMR spectrum 6-bromoindolyl-3-glyoxyl-L-histidine (**39**) recorded in DMSO-d_6
**Figure S66**	^{1}H NMR spectrum for 6-bromoindolyl-3-glyoxyl-L-histidine (**40**) recorded in DMSO-d_6
**Figure S67**	^{13}C NMR spectrum for 6-bromoindolyl-3-glyoxyl-L-histidine (**40**) recorded in DMSO-d_6
**Figure S68**	HSQC NMR spectrum 6-bromoindolyl-3-glyoxyl-L-histidine (**40**) recorded in DMSO-d_6
**Figure S69**	COSY NMR spectrum 6-bromoindolyl-3-glyoxyl-L-histidine (**40**) recorded in DMSO-d_6
**Figure S70**	^{1}H NMR spectrum for 5-bromoindolyl-3-glyoxyl-L-isoleucine (**41**) recorded in DMSO-d_6
**Figure S71**	^{13}C NMR spectrum for 5-bromoindolyl-3-glyoxyl-L-isoleucine (**41**) recorded in DMSO-d_6
**Figure S72**	HSQC NMR spectrum 5-bromoindolyl-3-glyoxyl-L-isoleucine (**41**) recorded in DMSO-d_6
**Figure S73**	COSY NMR spectrum 5-bromoindolyl-3-glyoxyl-L-isoleucine (**41**) recorded in DMSO-d_6
**Figure S74**	^{1}H NMR spectrum for 6-bromoindolyl-3-glyoxyl-L-isoleucine (**42**) recorded in DMSO-d_6
**Figure S75**	^{13}C NMR spectrum for 6-bromoindolyl-3-glyoxyl-L-isoleucine (**42**) recorded in DMSO-d_6
**Figure S76**	HSQC NMR spectrum 6-bromoindolyl-3-glyoxyl-L-isoleucine (**42**) recorded in DMSO-d_6
**Figure S77**	COSY NMR spectrum 6-bromoindolyl-3-glyoxyl-L-isoleucine (**42**) recorded in DMSO-d_6
**Figure S78**	^{1}H NMR spectrum for 5-bromoindolyl-3-glyoxyl-L-serine (**43**) recorded in DMSO-d_6
**Figure S79**	^{13}C NMR spectrum for 5-bromoindolyl-3-glyoxyl-L-serine (**43**) recorded in DMSO-d_6
**Figure S80**	HSQC NMR spectrum 5-bromoindolyl-3-glyoxyl-L-serine (**43**) recorded in DMSO-d_6
**Figure S81**	COSY NMR spectrum 5-bromoindolyl-3-glyoxyl-L-serine (**43**) recorded in DMSO-d_6

**Figure S82**	^{1}H NMR spectrum for 6-bromoindolyl-3-glyoxyl-L-serine (**44**) recorded in DMSO-d_6
**Figure S83**	^{13}C NMR spectrum for 6-bromoindolyl-3-glyoxyl-L-serine (**44**) recorded in DMSO-d_6
**Figure S84**	HSQC NMR spectrum 6-bromoindolyl-3-glyoxyl-L-serine (**44**) recorded in DMSO-d_6
**Figure S85**	COSY NMR spectrum 6-bromoindolyl-3-glyoxyl-L-serine (**44**) recorded in DMSO-d_6
**Figure S86**	^{1}H NMR spectrum for 5-bromoindolyl-3-glyoxyl-L-tryptophan (**45**) recorded in DMSO-d_6
**Figure S87**	^{13}C NMR spectrum for 5-bromoindolyl-3-glyoxyl-L-tryptophan (**45**) recorded in DMSO-d_6
**Figure S88**	HSQC NMR spectrum 5-bromoindolyl-3-glyoxyl--tryptophan (**45**) recorded in DMSO-d_6
**Figure S89**	COSY NMR spectrum 5-bromoindolyl-3-glyoxyl-L-tryptophan (**45**) recorded in DMSO-d_6
**Figure S90**	^{1}H NMR spectrum for 5-bromoindolyl-3-glyoxyl-D-tryptophan (**46**) recorded in DMSO-d_6
**Figure S91**	^{13}C NMR spectrum for 5-bromoindolyl-3-glyoxyl-D-tryptophan (**46**) recorded in DMSO-d_6
**Figure S92**	HSQC NMR spectrum 5-bromoindolyl-3-glyoxyl-D-tryptophan (**46**) recorded in DMSO-d_6
**Figure S93**	COSY NMR spectrum 5-bromoindolyl-3-glyoxyl-D-tryptophan (**46**) recorded in DMSO-d_6
**Figure S94**	^{1}H NMR spectrum for 6-bromoindolyl-3-glyoxyl-L-tryptophan (**47**) recorded in DMSO-d_6
**Figure S95**	^{13}C NMR spectrum for 6-bromoindolyl-3-glyoxyl-L-tryptophan (**47**) recorded in DMSO-d_6
**Figure S96**	HSQC NMR spectrum 6-bromoindolyl-3-glyoxyl-L-tryptophan (**47**) recorded in DMSO-d_6
**Figure S97**	COSY NMR spectrum 6-bromoindolyl-3-glyoxyl-L-tryptophan (**47**) recorded in DMSO-d_6
**Figure S98**	^{1}H NMR spectrum for 6-bromoindolyl-3-glyoxyl-D-tryptophan (**48**) recorded in DMSO-d_6
**Figure S99**	^{13}C NMR spectrum for 6-bromoindolyl-3-glyoxyl-D-tryptophan (**48**) recorded in DMSO-d_6
**Figure S100**	HSQC NMR spectrum 6-bromoindolyl-3-glyoxyl-D-tryptophan (**48**) recorded in DMSO-d_6
**Figure S101**	COSY NMR spectrum 6-bromoindolyl-3-glyoxyl-D-tryptophan (**48**) recorded in DMSO-d_6
**Figure S102**	^{1}H NMR spectrum for 5-bromoindolyl-3-glyoxyl-L-tyrosine (**49**) recorded in DMSO-d_6
**Figure S103**	^{13}C NMR spectrum for 5-bromoindolyl-3-glyoxyl-L-tyrosine (**49**) recorded in DMSO-d_6
**Figure S104**	HSQC NMR spectrum 5-bromoindolyl-3-glyoxyl-L-tyrosine (**49**) recorded in DMSO-d_6
**Figure S105**	COSY NMR spectrum 5-bromoindolyl-3-glyoxyl-L-tyrosine (**49**) recorded in DMSO-d_6
**Figure S106**	^{1}H NMR spectrum for 5-bromoindolyl-3-glyoxyl-D-tyrosine (**50**) recorded in DMSO-d_6
**Figure S107**	^{13}C NMR spectrum for 5-bromoindolyl-3-glyoxyl-D-tyrosine (**50**) recorded in DMSO-d_6
**Figure S108**	HSQC NMR spectrum 5-bromoindolyl-3-glyoxyl-D-tyrosine (**50**) recorded in DMSO-d_6
**Figure S109**	COSY NMR spectrum 5-bromoindolyl-3-glyoxyl-D-tyrosine (**50**) recorded in DMSO-d_6
**Figure S110**	^{1}H NMR spectrum for 6-bromoindolyl-3-glyoxyl-L-tyrosine (**51**) recorded in DMSO-d_6
**Figure S111**	^{13}C NMR spectrum for 6-bromoindolyl-3-glyoxyl-L-tyrosine (**51**) recorded in DMSO-d_6
**Figure S112**	HSQC NMR spectrum 6-bromoindolyl-3-glyoxyl-L-tyrosine (**51**) recorded in DMSO-d_6
**Figure S113**	COSY NMR spectrum 6-bromoindolyl-3-glyoxyl-L-tyrosine (**51**) recorded in DMSO-d_6
**Figure S114**	^{1}H NMR spectrum for 6-bromoindolyl-3-glyoxyl-D-tyrosine (**52**) recorded in DMSO-d_6
**Figure S115**	^{13}C NMR spectrum for 6-bromoindolyl-3-glyoxyl-D-tyrosine (**52**) recorded in DMSO-d_6
**Figure S116**	HSQC NMR spectrum 6-bromoindolyl-3-glyoxyl-D-tyrosine (**52**) recorded in DMSO-d_6
**Figure S117**	COSY NMR spectrum 6-bromoindolyl-3-glyoxyl-D-tyrosine (**52**) recorded in DMSO-d_6
**Figure S118**	^{1}H NMR spectrum for 5-bromoindolyl-3-glyoxyl-L-valine (**53**) recorded in DMSO-d_6
**Figure S119**	^{13}C NMR spectrum for 5-bromoindolyl-3-glyoxyl-L-valine (**53**) recorded in DMSO-d_6
**Figure S120**	HSQC NMR spectrum 5-bromoindolyl-3-glyoxyl-L-valine (**53**) recorded in DMSO-d_6
**Figure S121**	COSY NMR spectrum 5-bromoindolyl-3-glyoxyl-L-valine (**53**) recorded in DMSO-d_6
**Figure S122**	^{1}H NMR spectrum for 5-bromoindolyl-3-glyoxyl-D-valine (**54**) recorded in DMSO-d_6
**Figure S123**	^{13}C NMR spectrum for 5-bromoindolyl-3-glyoxyl-D-valine (**54**) recorded in DMSO-d_6
**Figure S124**	HSQC NMR spectrum 5-bromoindolyl-3-glyoxyl-D-valine (**54**) recorded in DMSO-d_6
**Figure S125**	COSY NMR spectrum 5-bromoindolyl-3-glyoxyl-D-valine (**54**) recorded in DMSO-d_6
**Figure S126**	^{1}H NMR spectrum for 6-bromoindolyl-3-glyoxyl-L-valine (**55**) recorded in DMSO-d_6
**Figure S127**	^{13}C NMR spectrum for 6-bromoindolyl-3-glyoxyl-L-valine (**55**) recorded in DMSO-d_6
**Figure S128**	HSQC NMR spectrum 6-bromoindolyl-3-glyoxyl-L-valine (**55**) recorded in DMSO-d_6
**Figure S129**	COSY NMR spectrum 6-bromoindolyl-3-glyoxyl-L-valine (**55**) recorded in DMSO-d_6
**Figure S130**	^{1}H NMR spectrum for 6-bromoindolyl-3-glyoxyl-D-valine (**56**) recorded in DMSO-d_6
**Figure S131**	^{13}C NMR spectrum for 6-bromoindolyl-3-glyoxyl-D-valine (**56**) recorded in DMSO-d_6
**Figure S132**	HSQC NMR spectrum 6-bromoindolyl-3-glyoxyl-D-valine (**56**) recorded in DMSO-d_6
**Figure S133**	COSY NMR spectrum 6-bromoindolyl-3-glyoxyl-D-valine (**56**) recorded in DMSO-d_6
**Figure S134-140**	α-synuclein MS binding assay results (**25**, **26**, **34**, **37**, **45**, **46**, **51**, **and 52**)

**Figure S141**	Dose-response curves for synthetic indoles **46**, **48**, **50**, and **51** against chloroquine-sensitive (3D7) and -resistant (Dd2) *Plasmodium falciparum* parasite strains and a human embryonic cell line (HEK293).

2. References

Table S1. Marine indol-3-yl-glyoxylamides **1-24** (SMILES, Compound name, Phylum, Genus, Species and year isolated) reported to the end of 2021 (MarinLit database).[1]

Manuscript No.	SMILES Code	Compound	Phylum	Genus	Species	Year
1	O=C(C(C1=CNC2=C1C=CC=C2)=O)N[C@@H](CCC[NH+]=C(N)N)C(O)=O	leptoclinidamine A	Chordata	Leptoclinides	durus	2009
2	O=C(C(C1=CNC2=C1C=CC(O)=C2)=O)N[C@@H](CCC[NH+]=C(N)N)C(O)=O	leptoclinidamine B	Chordata	Leptoclinides	durus	2009
3	O=C(C(C1=CNC2=C1C=CC(O)=C2)=O)N[C@@H](CCCN=C(N)N)C(O)=O	(-)-leptoclinidamine B	Chordata	Leptoclinides	dubius	2012
4	O=C(C(C1=CNC2=C1C=CC(O)=C2)=O)NCCCC[C@@H](C(O)=O)N	herdmanine I	Chordata	Herdmania	momus	1990
5	O=C(C(C1=CNC2=C1C=CC(O)=C2)=O)N[C@@H](CC3=CNC=N3)C(O)=O	herdmanine K	Chordata	Herdmania	momus	2012
6	BrC(C=C1CCNC(C(C2=CNC3=C2C=CC=C3)=O)=O)=C(C=C1)O	polyandrocarpamide A	Chordata	Polyandrocarpa	sp	1990
7	IC(C=C1CCNC(C(C2=CNC3=C2C=CC=C3)=O)=O)=C(C=C1)O	polyandrocarpamide B	Chordata	Polyandrocarpa	sp	2012
8	O=C(C(C1=CNC2=C1C=CC=C2)=O)NCCC(C=C3)=CC=C3O	polyandrocarpamide C	Chordata	Polyandrocarpa	sp	1990
9	O=C(C(C1=CNC2=C1C=CC=C2)=O)NCCC[NH2+]CCCC[NH3+]	didemnidine A	Chordata	Didemnum	sp	2011
10	BrC(C=C1)=CC2=C1C(C(C(NCCC[NH2+]CCCC[NH3+])=O)=O)=CN2	didemnidine B	Chordata	Didemnum	sp	2011
11	BrC(C=C1)=CC2=C1C(/C=C/NC(C(C3=CNC4=C3C=CC=C4O)=O)=O)=CN2	coscinamide C	Porifera	Coscinoderma	sp	2000
12	O=C(C(C1=CNC2=C1C=CC(Br)=C2)=O)N/C=C\C3=CNC4=C3C=CC=C4	(Z)-coscinamide D	Porifera	Lamellomorpha	strongylata	2019
13	O=C(NC1)C2=C1C(C(C=CC=C3)=C3N45)=C4C6=C2C7=C(C=CC=C7)N6[C@@H]8O[C@@]5(C)[C@H](OC)[C@H](NC(C(C9=CNC%10=C9C=CC=C%10)=O)=O)C8	13G-29I	Actinobacteria	Streptomyces	sp	2020
14	BrC(C=C1)=CC2=C1C(/C=C/NC(C(C3=CNC4=C3C=CC=C4)=O)=O)=CN2	coscinamide A	Porifera	Coscinoderma	sp	2000
15	O=C(C(C1=CNC2=C1C=CC=C2)=O)N/C=C/C3=CNC4=C3C=CC=C4	coscinamide B	Porifera	Coscinoderma	sp	2000
16	O=C(C(C1=CNC2=C1C=CC=C2)=O)N[C@H](CO)[C@@H](C(C=C3)=CC=C3OCCC#N)O	preoxazinin-7	Mollusca	Mytilus	galloprovincialis	2007
17	O=C(C1=O)N[C@@H](C(C2=CNC(C=C3)=C2C=C3O)=O)C4=C5C1=CNC5=CC=C4O	hyrtimomine F	Porifera	Hyrtios	sp	2014
18	O=C(C(NCCS(=O)(O)=O)=O)C1=CNC2=CC=CC=C21	stolonine A	Chordata	Cnemidocarpa	stolonifera	2015
19	O=C(C(C1=CNC2=C1C=CC(Br)=C2)=O)N/C=C/C3=CNC4=C3C=CC=C4	(E)-coscinamide D	Porifera	Lamellomorpha	strongylata	2019
20	[NH3+]C[C@@H](NC(C(C1=CNC2=C1C=CC=C2)=O)=O)C3=CNC4=CC(Br)=CC=C43	calcicamide A	Porifera	Spongosorites	calcicola	2019
21	O=C(C(C1=CNC2=C1C=CC=C2)=O)NCCCCCC#CCCCCCCCCCC3=CN=CC=C3	pyrinodemin F	Porifera	Amphimedon	sp	2011
22	O=C(C(C1=CNC2=C1C=CC=C2)=O)N	empty	Porifera	Spongosorites	sp	2007
23	BrC(C=C1)=CC2=C1C(C(C(N)=O)=O)=CN2	empty	Porifera	Spongosorites	sp	2007
24	[NH3+][C@H](CNC(C(C1=CNC2=C1C=CC=C2)=O)=O)C3=CNC4=CC(Br)=CC=C43	calcicamide B	Porifera	Spongosorites	calcicola	2019

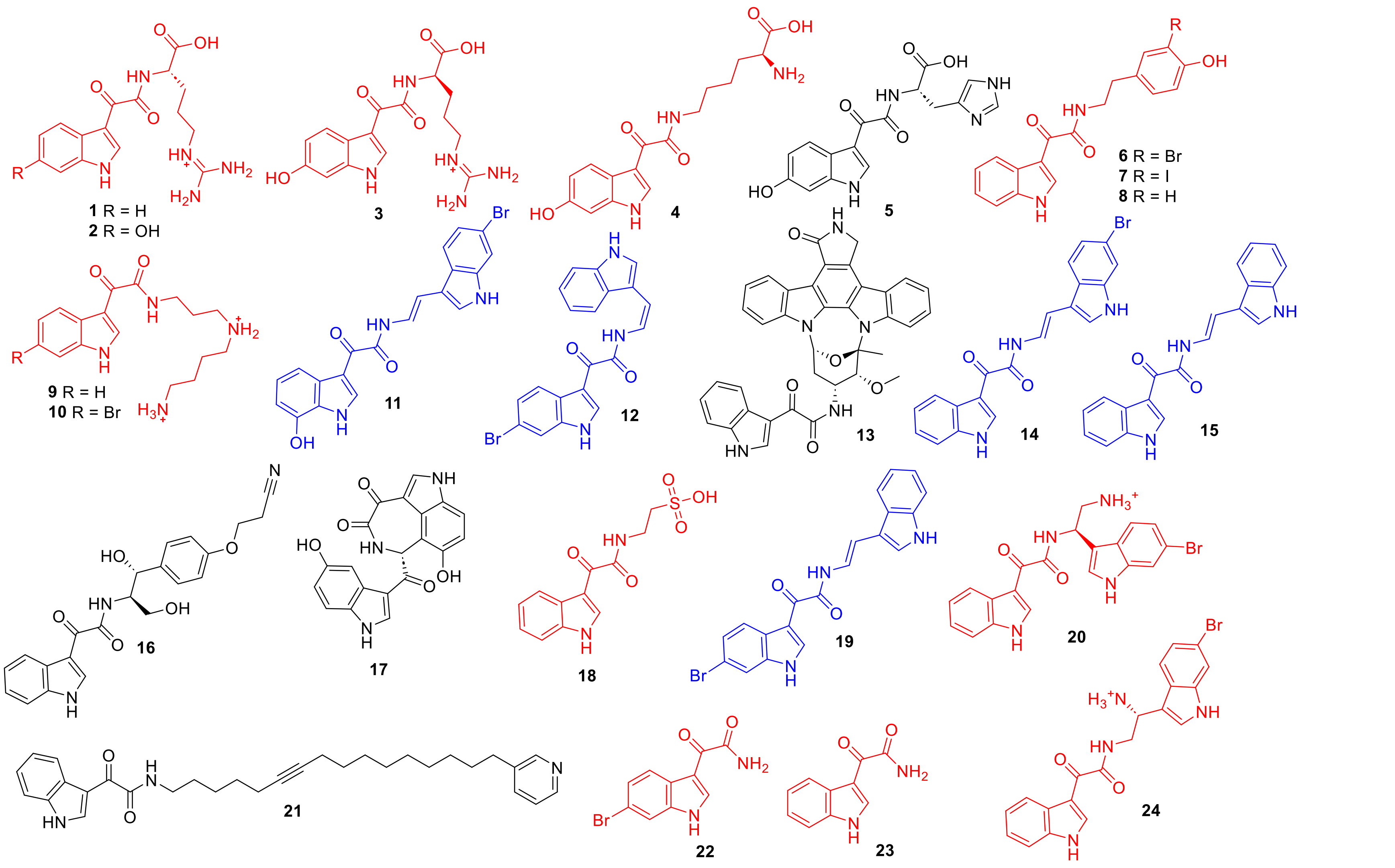

Figure S1. Marine indol-3-yl-glyoxyl-amides **1-24** reported in MarinLit to the end of 2021 (red = red cluster, blue = blue cluster, black = singleton IGAs) reported in MarinLit (end of 2021).

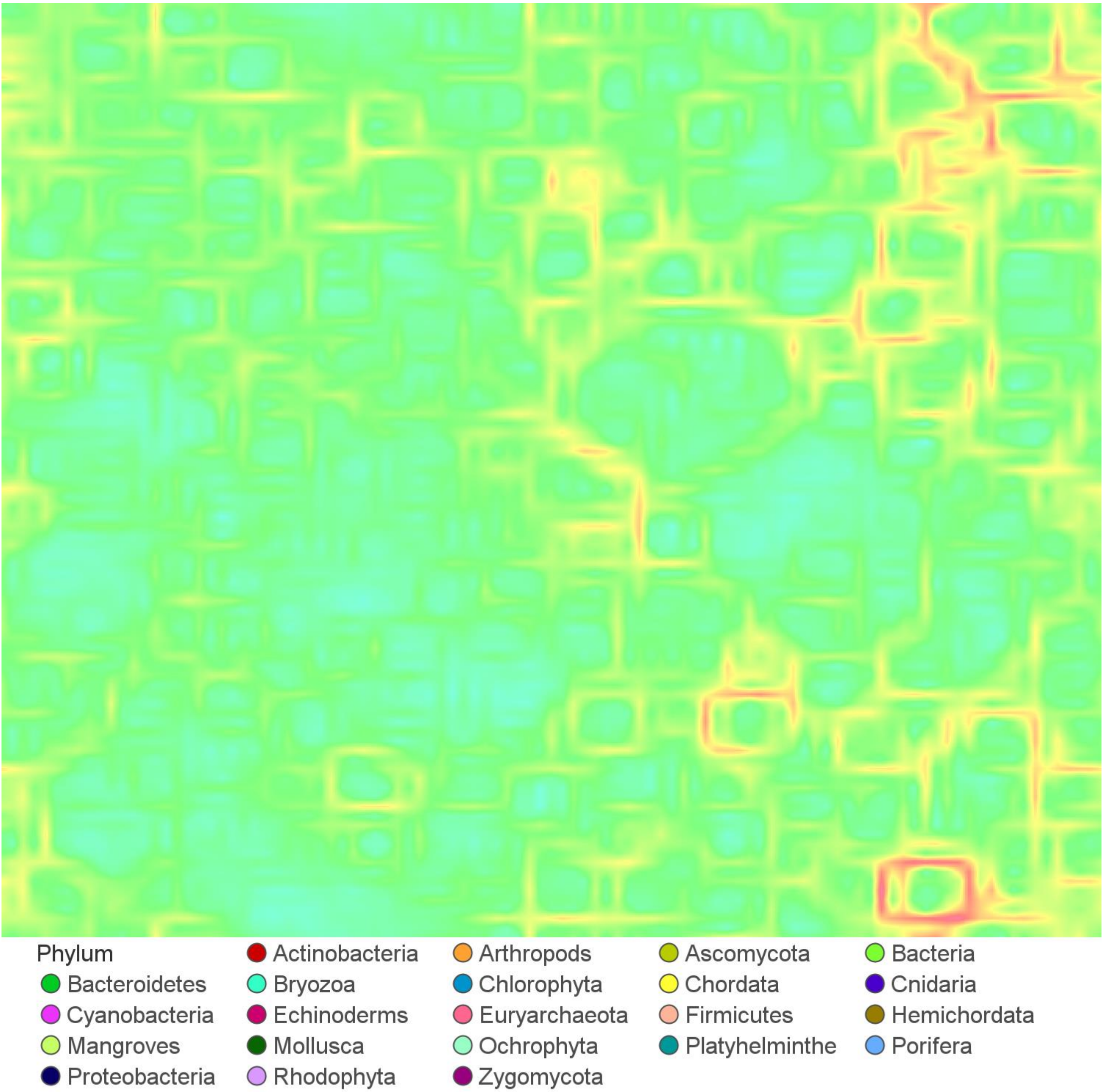

Figure S2. Background topography of marine indole alkaloid chemical diversity visualized in a self-organizing map (50 x 50 neuron, SkelSpheres chemical descriptor).

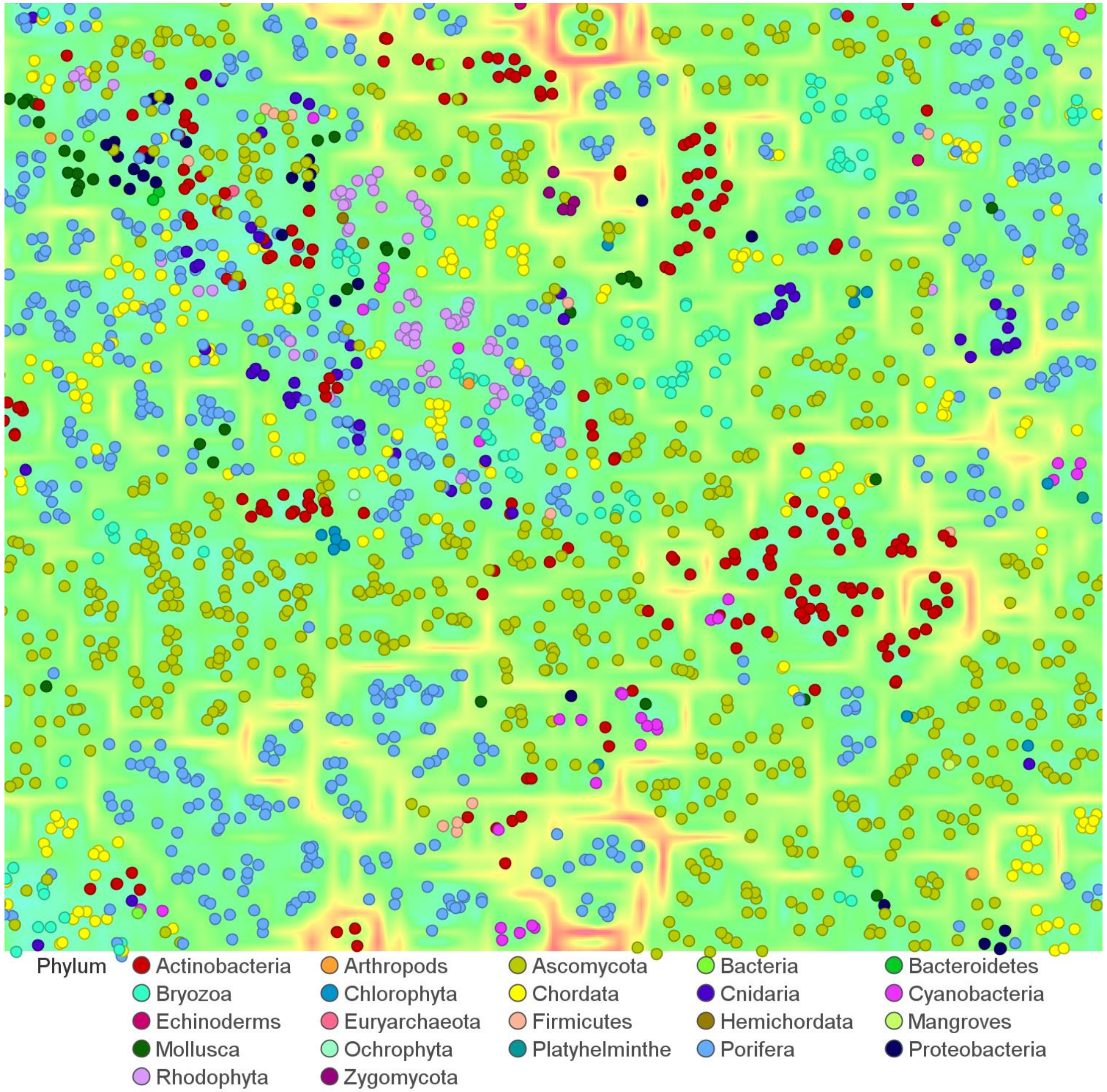

Figure S3. Chemical diversity of marine indole alkaloid chemical diversity (*n* = 2048) by producing phylum visualized in a self-organizing map (50 x 50 neuron, SkelSpheres chemical descriptor).

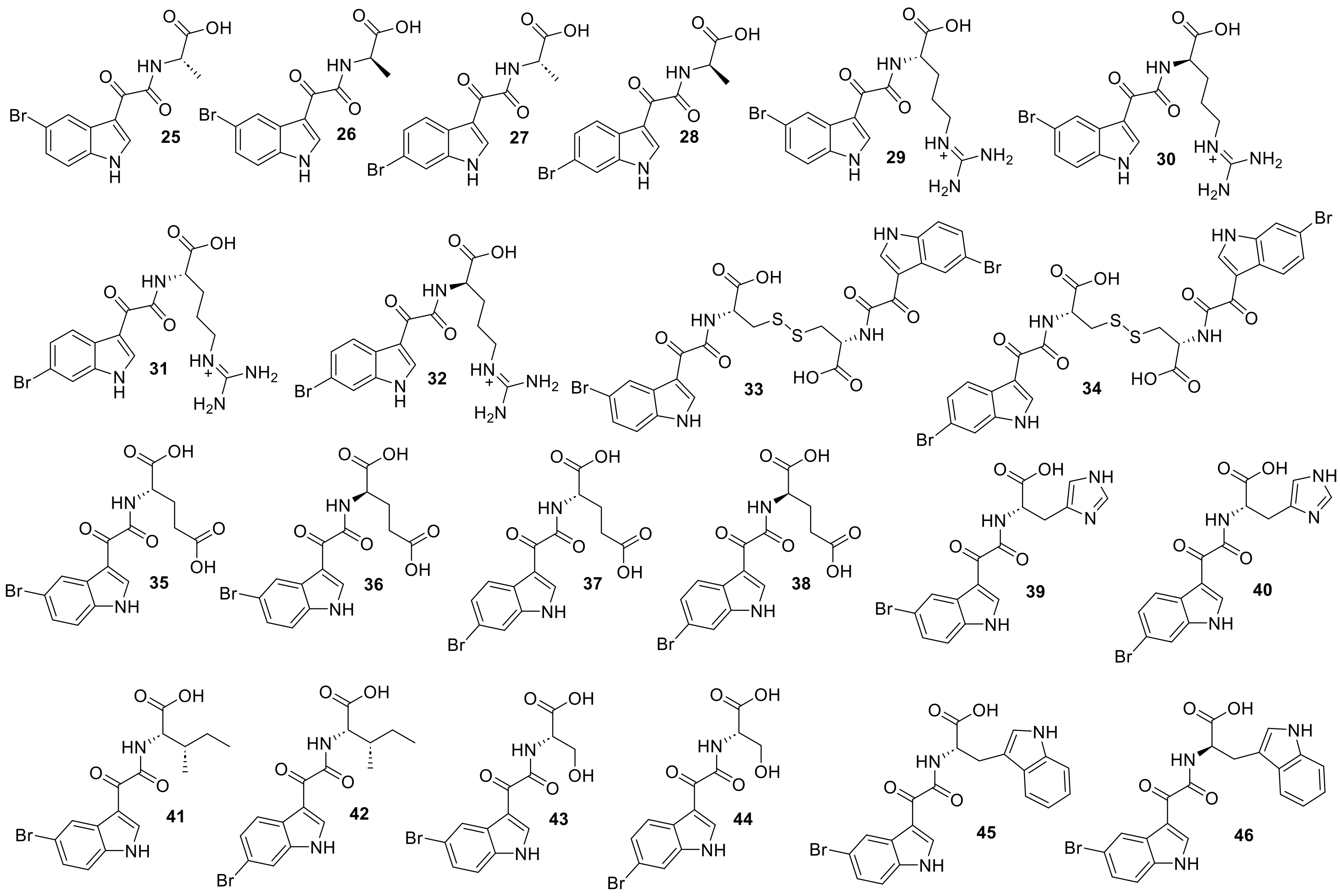

Figure S4. Synthetic indol-3-yl-glyoxylamides **25-46**.

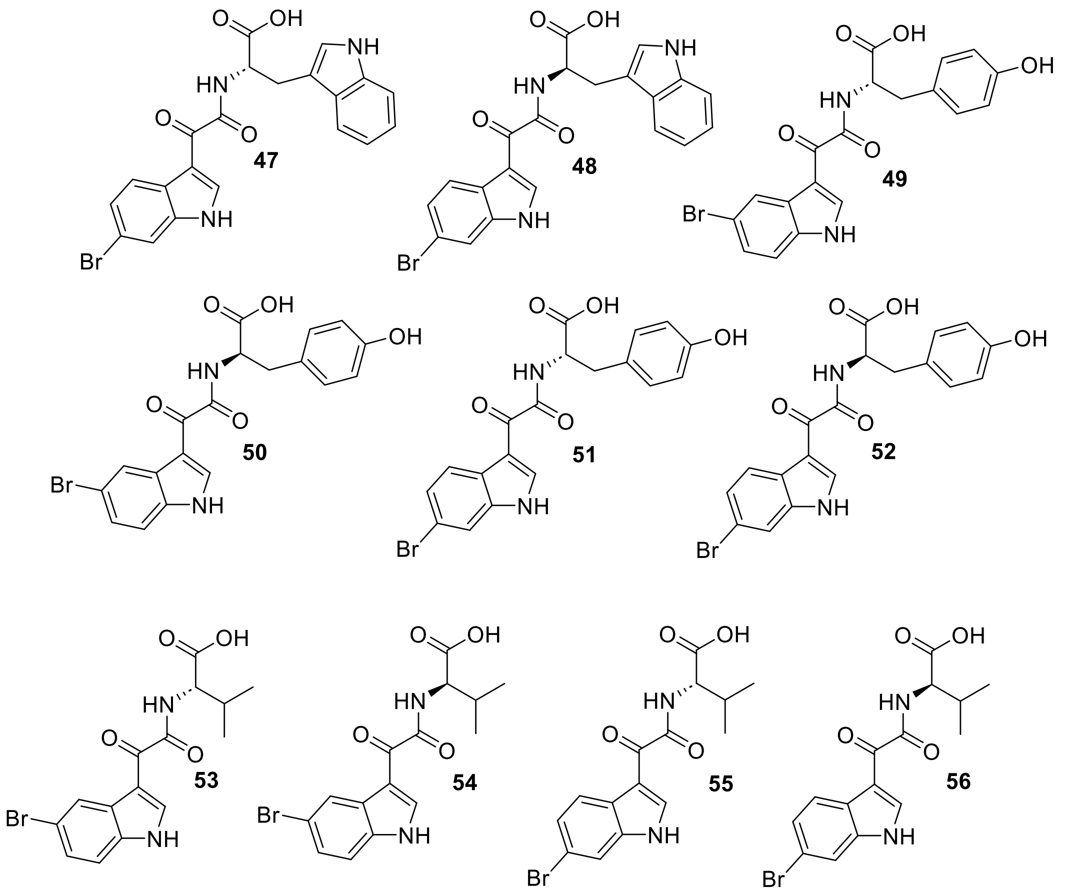

Figure S5. Synthetic indol-3-yl-glyoxylamides **47-56**.

Table S2. Potency of bioactivity criteria used for categorising NP potency of activity relevant to drug discovery.[1,2]

Bioactivity criteria	IC_{50}
**cytotoxic/antiparasitic/antioxidant/anti-inflammatory/antiviral/enzyme inhibitory***	
**Potent**	<0.1 µM
**Moderate**	0.1–1.0 µM
**Weak**	1.0–10 µM
**Inactive**	>10 µM
**Antibacterial/Antifungal Activity**	
	**MIC**
**Potent**	<1.0 µg/mL
**Moderate**	1.0–8.0 µg/mL
**Weak**	8.0–32.0 µg/mL
**Inactive**	>32.0 µg/mL

Bioassays reported as IC_{50}, EC_{50}, or GI_{50} were classified as inactive (>10 µM), weak (<10 µM), moderate (<1 µM), potent (<0.1 µM). For bioactivities reported as MIC the classifications were inactive (>32 µg/mL), weak (<32 µg/mL), moderate (<4 µg/mL) and potent (<1 µg/mL). Biological activities quoting disk diffusion/zone of inhibition, LC_{50}, LD_{50}, and ED_{50} were not considered.

Table S3. Inhibition of SARS-CoV-2 3CL^{PRO} viral protease at 20 µM for **25-56**.

Compound	R_1	R_2	R_3	% inhibition @ 20 µM	
				% inhibition No DTT (IC_{50} µM)	% inhibition DTT (IC_{50} µM)
**25**	Br	H	L-Ala	2.9	1.1
**26**	Br	H	D-Ala	0.8	1.0
**27**	H	Br	L-Ala	1.9	1.3
**28**	H	Br	D-Ala	1.7	0.8
**29**	Br	H	L-Arg	13.8	2.4
**30**	Br	H	D-Arg	10.1	1.0
**31**	H	Br	L-Arg	4.3	2.4
**32**	H	Br	D-Arg	3.0	0.8
**33**	Br	H	L-Cys	64.1 (12.5)	2.0
**34**	H	Br	L-Cys	86.1 (6.4)	4.6
**35**	Br	H	L-His	5.8	1.1
**36**	H	Br	L-His	5.2	1.4
**37**	Br	H	L-Ile	6.1	1.7
**38**	H	Br	L-Ile	5.1	0.6
**39**	Br	H	L-Glu	12.8	2.3
**40**	Br	H	D-Glu	9.1	3.0
**41**	H	Br	L-Glu	13.7	1.3
**42**	H	Br	D-Glu	19.5	4.2
**43**	Br	H	L-Ser	105.5 (1.2)	2.0
**44**	H	Br	L-Ser	7.3	2.9
**45**	Br	H	L-Trp	21.8	3.7
**46**	Br	H	D-Trp	27.4	2.5
**47**	H	Br	L-Trp	24.4	4.1
**48**	H	Br	D-Trp	20.8	2.0
**49**	Br	H	L-Tyr	15.0	1.9
**50**	Br	H	D-Tyr	6.1	0.9
**51**	H	Br	L-Tyr	23.3	6.9
**52**	H	Br	D-Tyr	14.0	4.9
**53**	Br	H	L-Val	1.9	1.3
**54**	Br	H	D-Val	1.3	1.0
**55**	H	Br	L-Val	6.8	3.0
**56**	H	Br	D-Val	6.8	2.7
**GC376**	-	-	-	99.4 (0.10)	100.2 (0.10)
**Ebselen**	-	-	-	114.2 (0.01)	0.2

Table S4. Inhibition of mammalian serine proteases (elastase and chymotrypsin) for **25-56**.

				% enzyme activity @ 10 μM (IC_{50} μM)	
**Compound**	**R_1**	**R_2**	**R_3**	**elastase**	**chymotrypsin**
**25**	Br	H	L-Ala	108.4	86.3
**26**	Br	H	D-Ala	104.4	92.7
**27**	H	Br	L-Ala	109.6	94.4
**28**	H	Br	D-Ala	107.4	92.1
**29**	Br	H	L-Arg	100.8	88.8
**30**	Br	H	D-Arg	96.2	86.3
**31**	H	Br	L-Arg	99.3	99.5
**32**	H	Br	D-Arg	102.3	99.2
**33**	Br	H	L-Cys	107.0	94.7
**34**	H	Br	L-Cys	93.5	93.1
**35**	Br	H	L-His	100.0	97.2
**36**	H	Br	L-His	104.0	95.4
**37**	Br	H	L-Ile	88.6	89.5
**38**	H	Br	L-Ile	91.1	97.2
**39**	Br	H	L-Glu	97.6	100.3
**40**	Br	H	D-Glu	97.1	91.9
**41**	H	Br	L-Glu	102.9	96.2
**42**	H	Br	D-Glu	105.4	93.9
**43**	Br	H	L-Ser	101.4	96.1
**44**	H	Br	L-Ser	99.0	97.1
**45**	Br	H	L-Trp	94.0	69.2 (16.3)
**46**	Br	H	D-Trp	90.9	47.7 (6.3)
**47**	H	Br	L-Trp	89.5	99.6
**48**	H	Br	D-Trp	91.1	83.6
**49**	Br	H	L-Tyr	99.5	82.9
**50**	Br	H	D-Tyr	95.3	89.2
**51**	H	Br	L-Tyr	100.3	91.8
**52**	H	Br	D-Tyr	97.1	97.2
**53**	Br	H	L-Val	101.3	91.6
**54**	Br	H	D-Val	105.4	97.3
**55**	H	Br	L-Val	88.9	105.0
**56**	H	Br	D-Val	104.4	94.0
**PMSF**	-	-	-	(138.1, ± 12.01)	(0.42, ± 0.2)

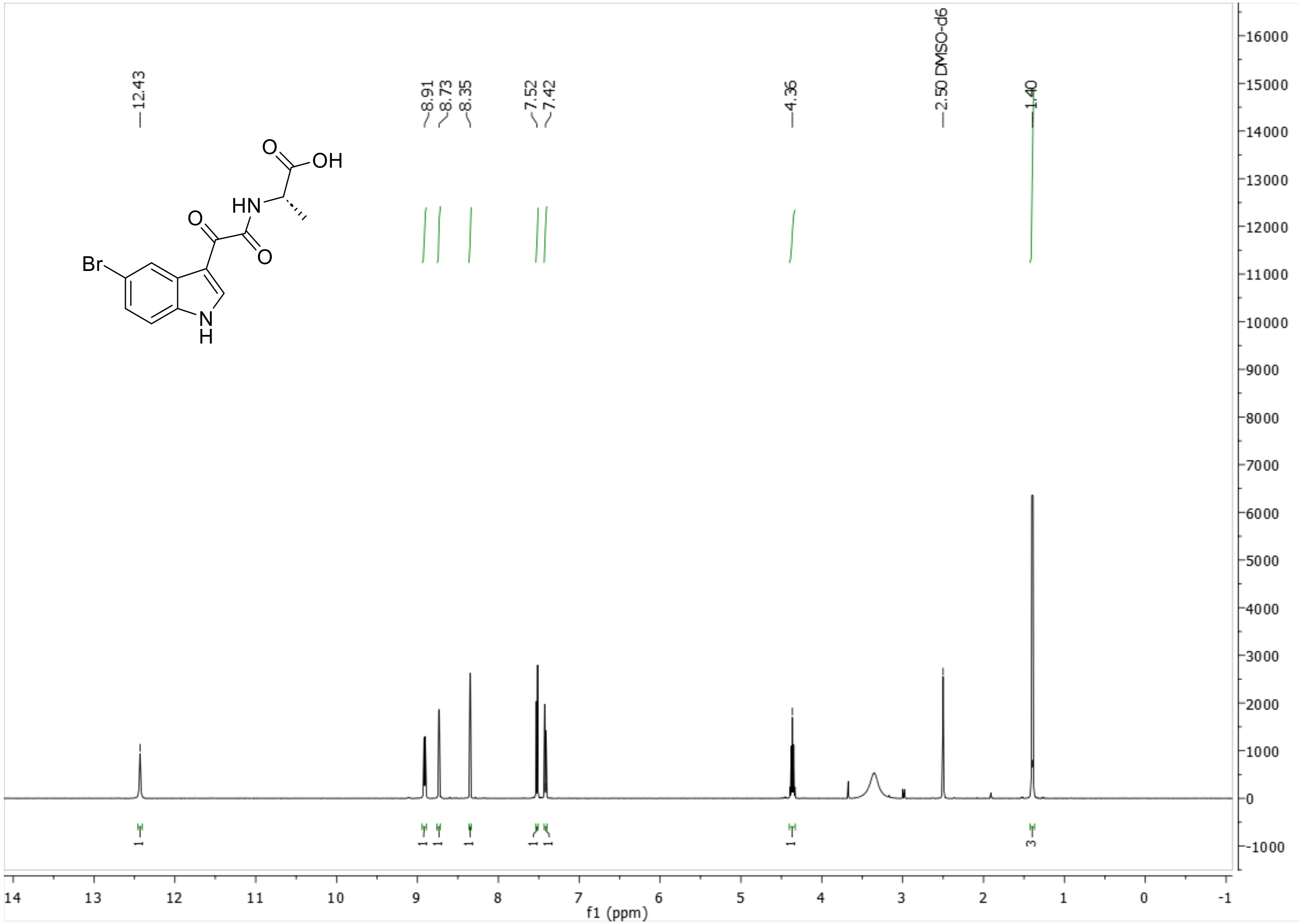

Figure S6. ^{1}H NMR spectrum for 5-bromoindolyl-3-glyoxyl-L-alanine (**25**) recorded in DMSO-d_6

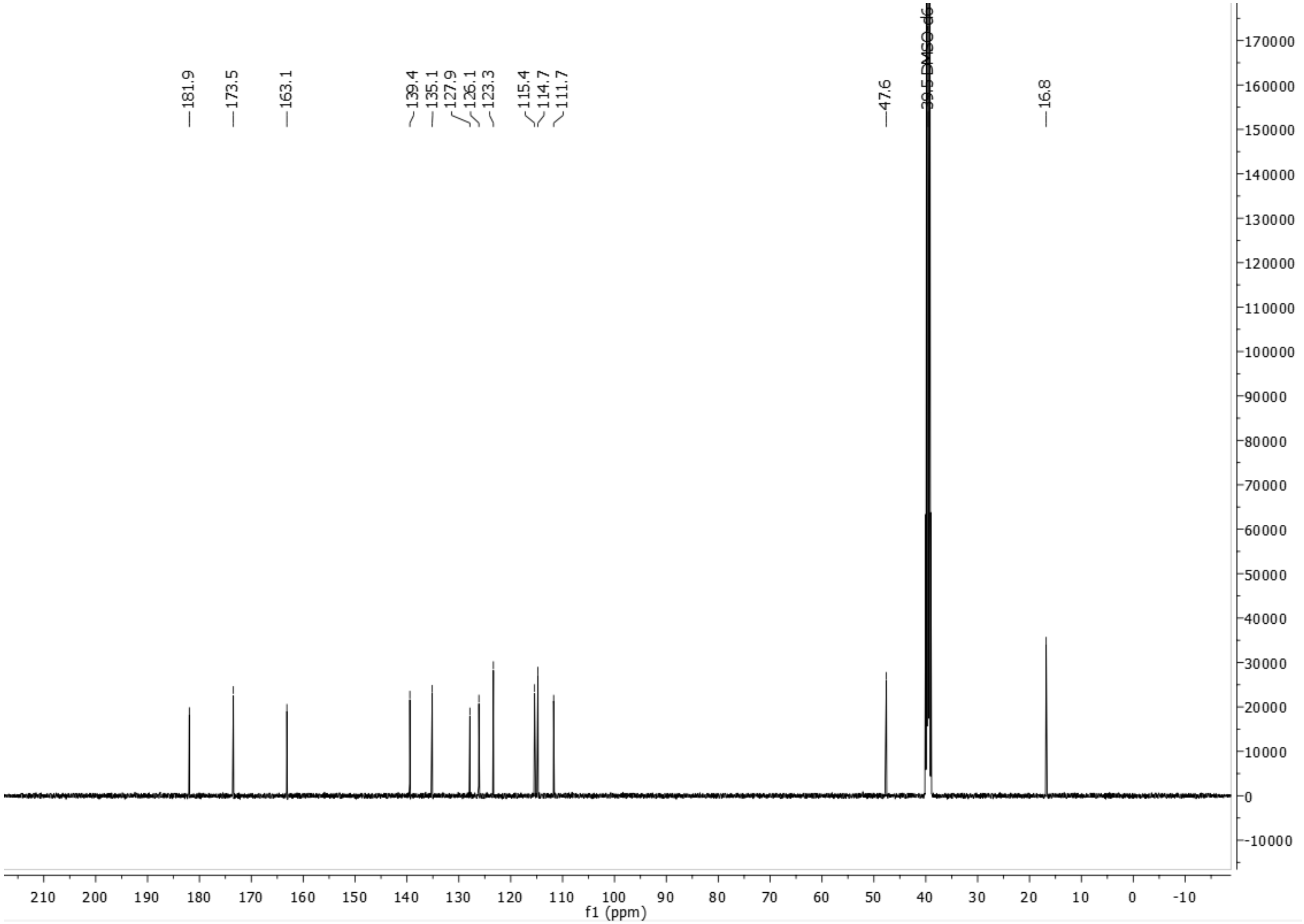

Figure S7. ^{13}C NMR spectrum for 5-bromoindolyl-3-glyoxyl-L-alanine (**25**) recorded in DMSO-d_6

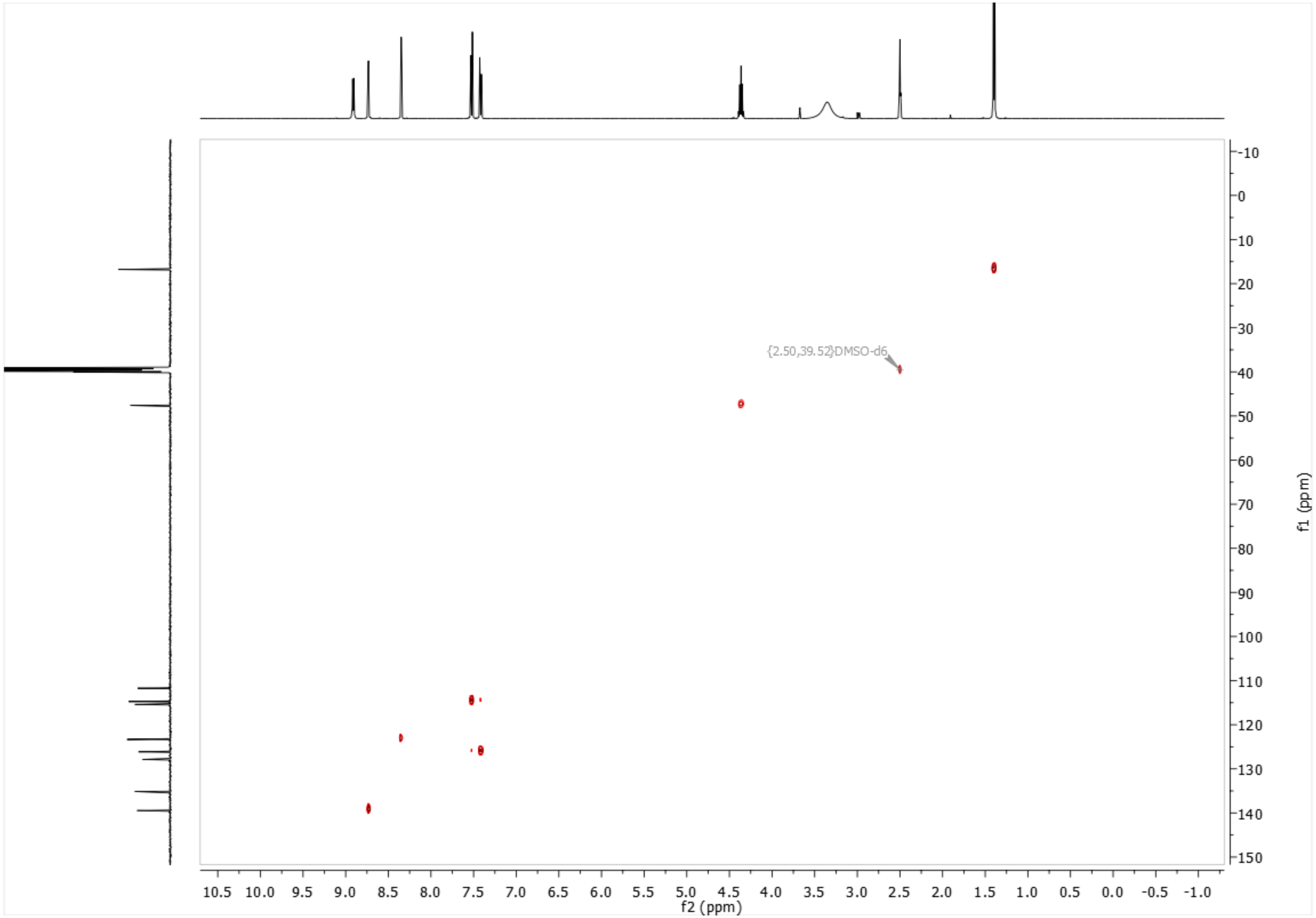

Figure S8. HSQC NMR spectrum 5-bromoindolyl-3-glyoxyl-L-alanine (**25**) recorded in DMSO-d_6

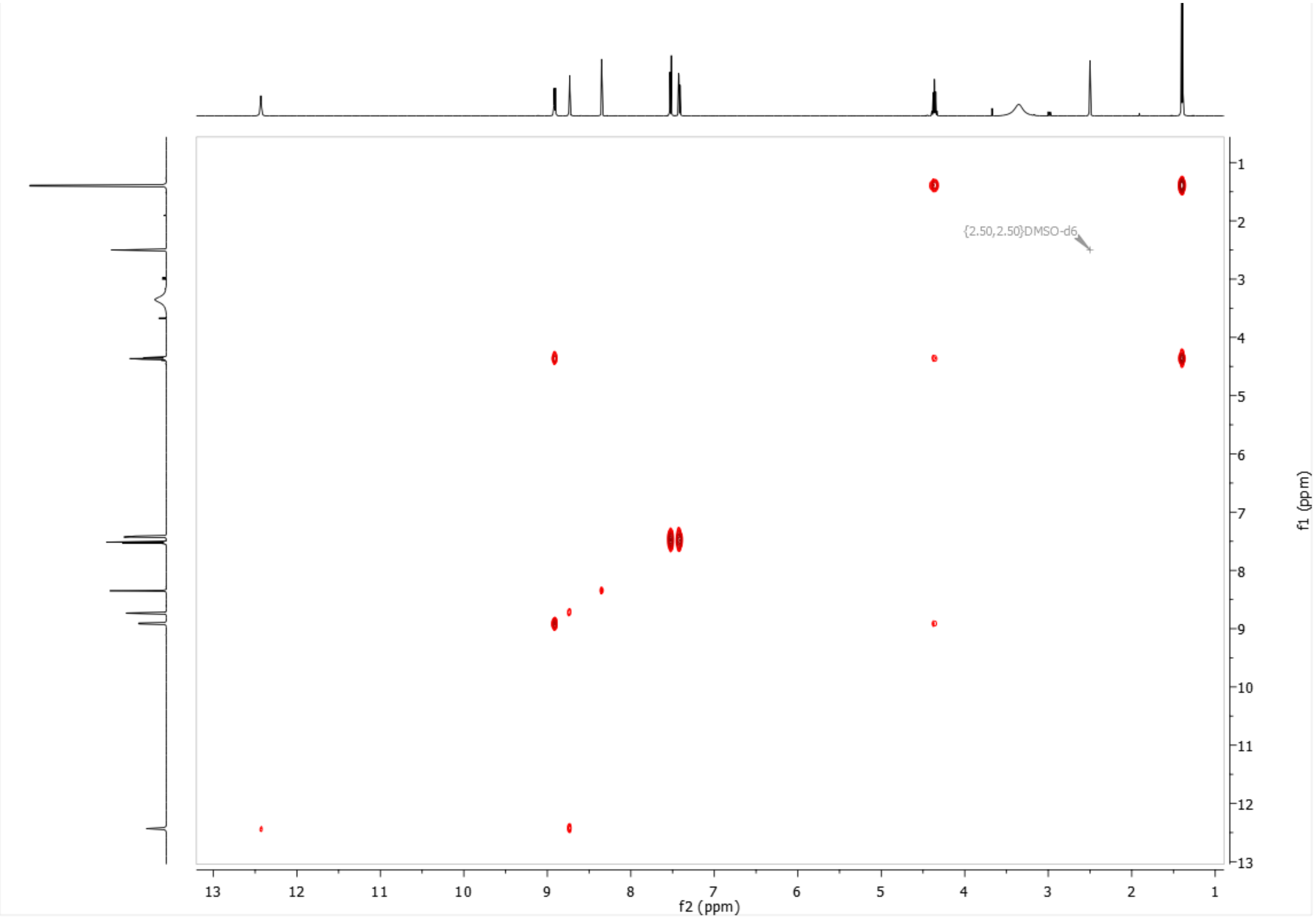

Figure S9. COSY NMR spectrum 5-bromoindolyl-3-glyoxyl-L-alanine (**25**) recorded in DMSO-d_6

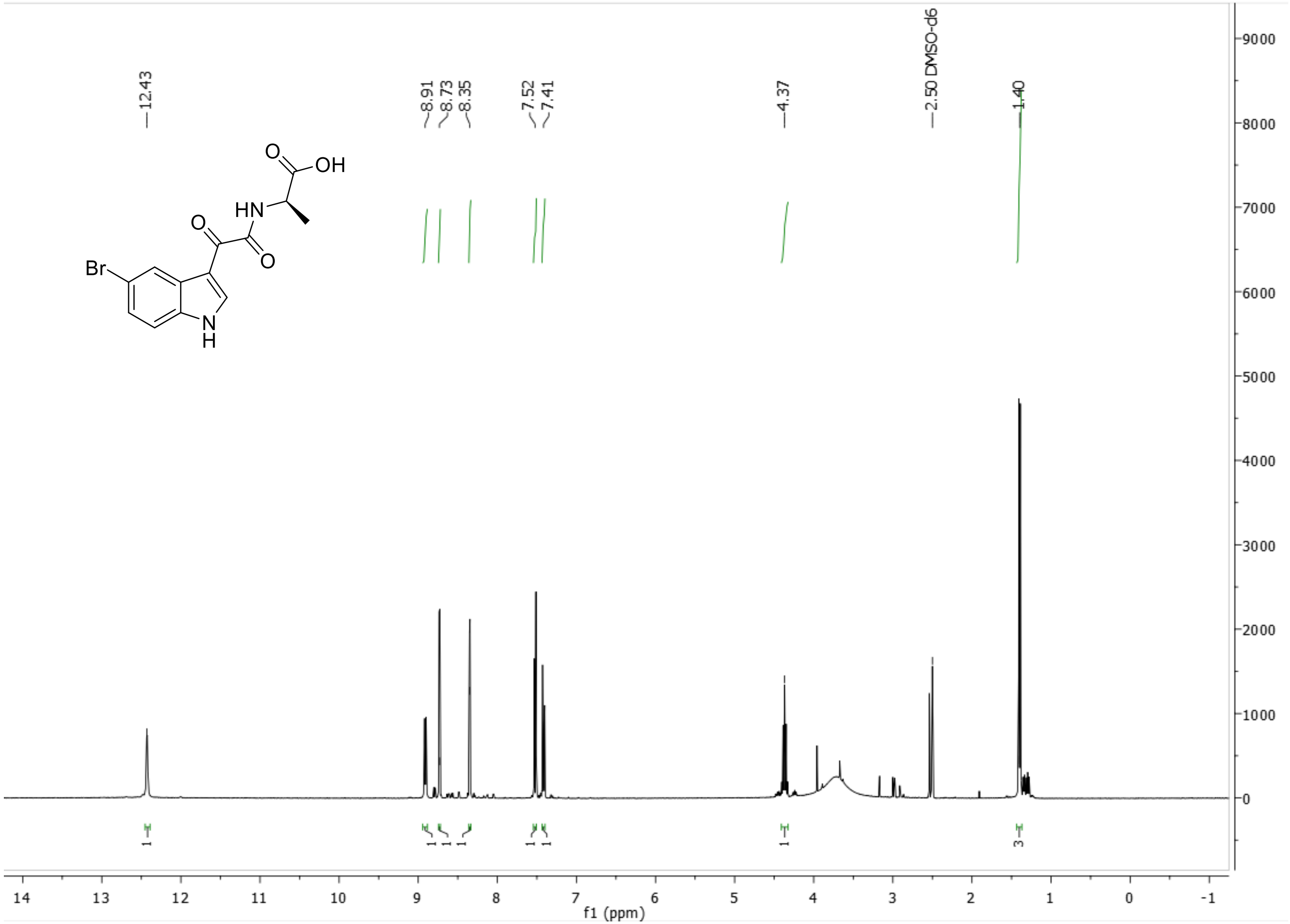

Figure S10. ^{1}H NMR spectrum for 5-bromoindolyl-3-glyoxyl-D-alanine (**26**) recorded in DMSO-d_6

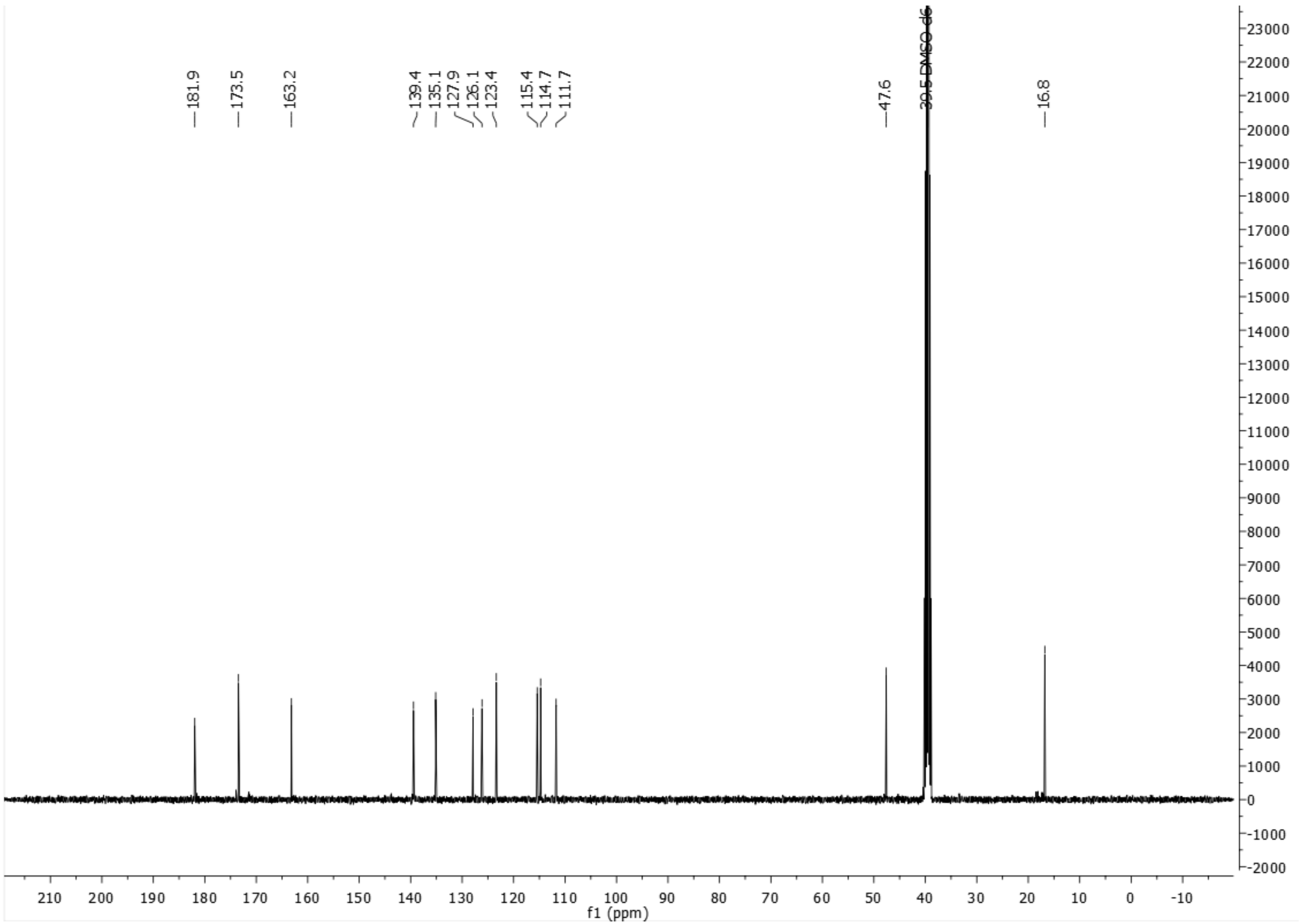

Figure S11. ^{13}C NMR spectrum for 5-bromoindolyl-3-glyoxyl-D-alanine (**26**) recorded in DMSO-d_6

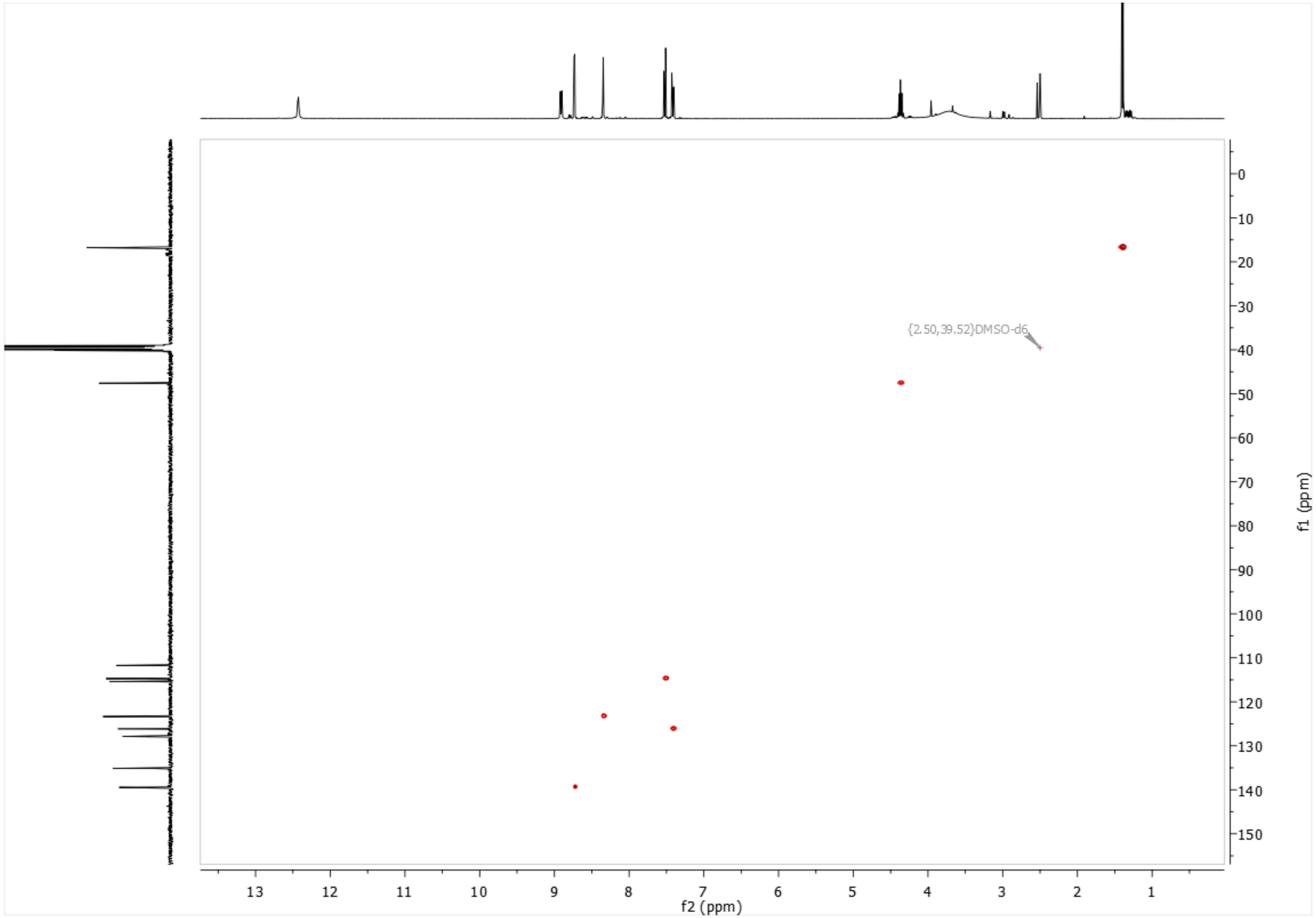

Figure S12. HSQC NMR spectrum for 5-bromoindolyl-3-glyoxyl-D-alanine (**26**) recorded in DMSO-d_6

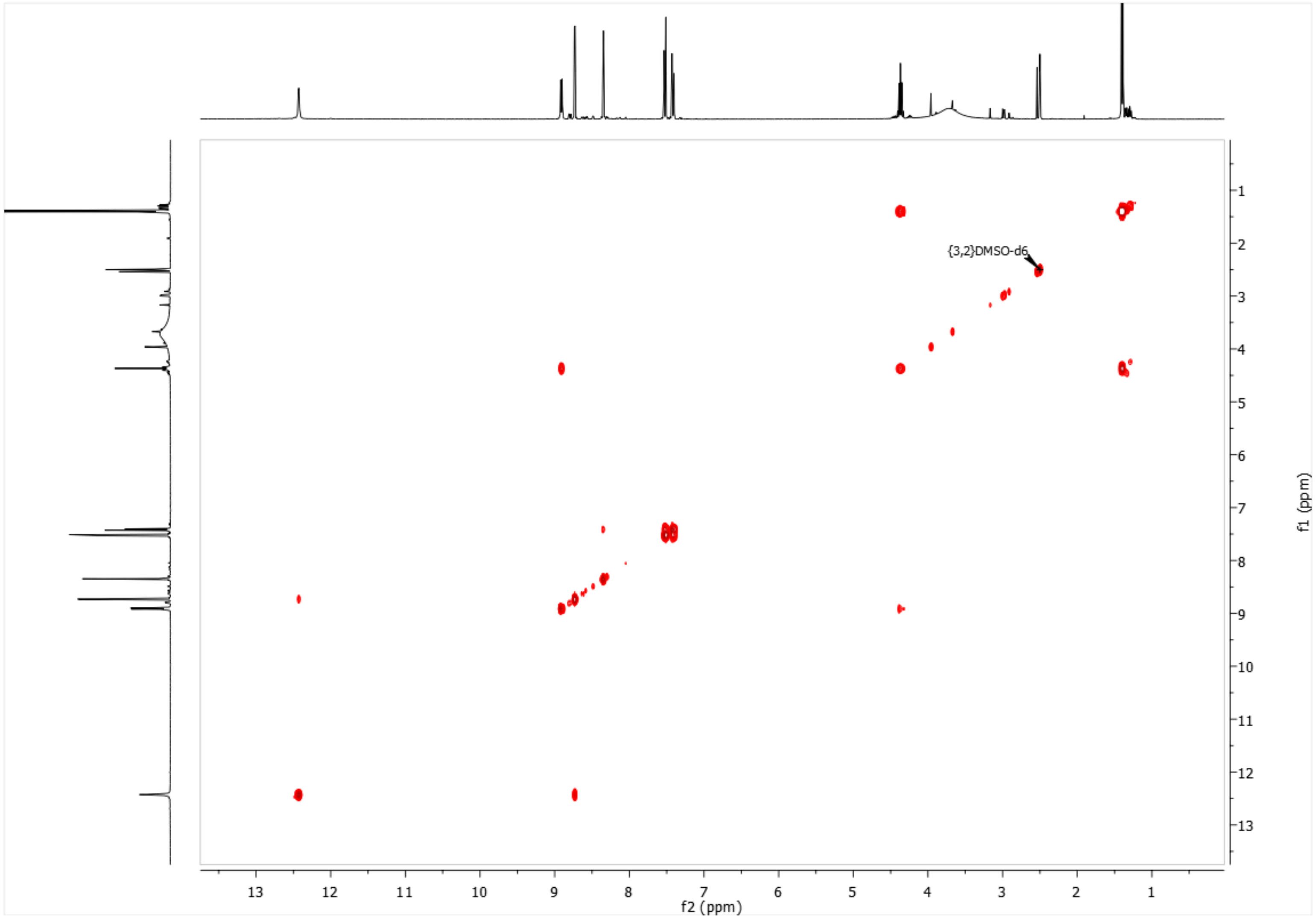

Figure S13. COSY NMR spectrum for 5-bromoindolyl-3-glyoxyl-D-alanine (**26**) recorded in DMSO-d_6

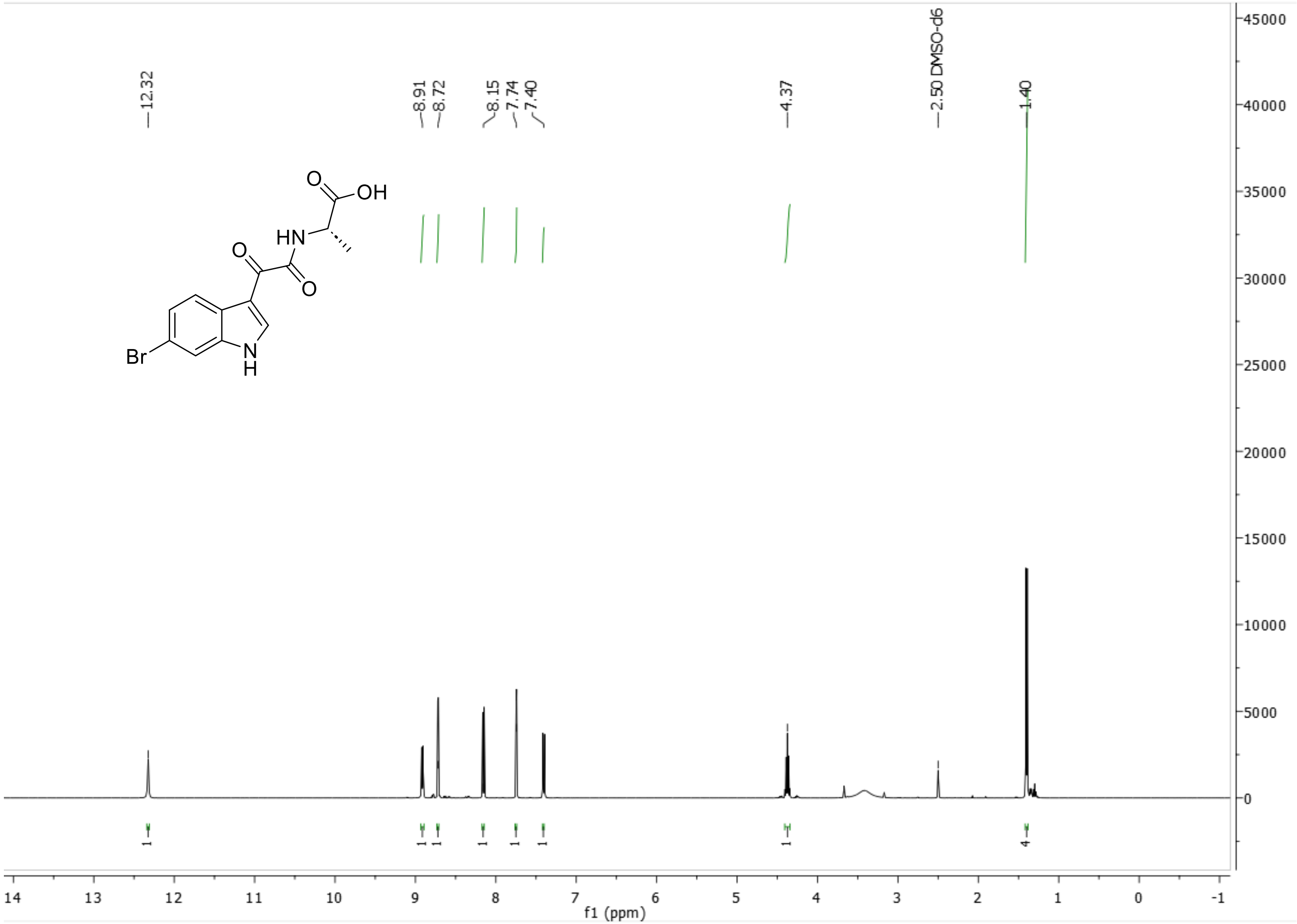

Figure S14. ^{1}H NMR spectrum for 6-bromoindolyl-3-glyoxyl-L-alanine (**27**) recorded in DMSO-d_6

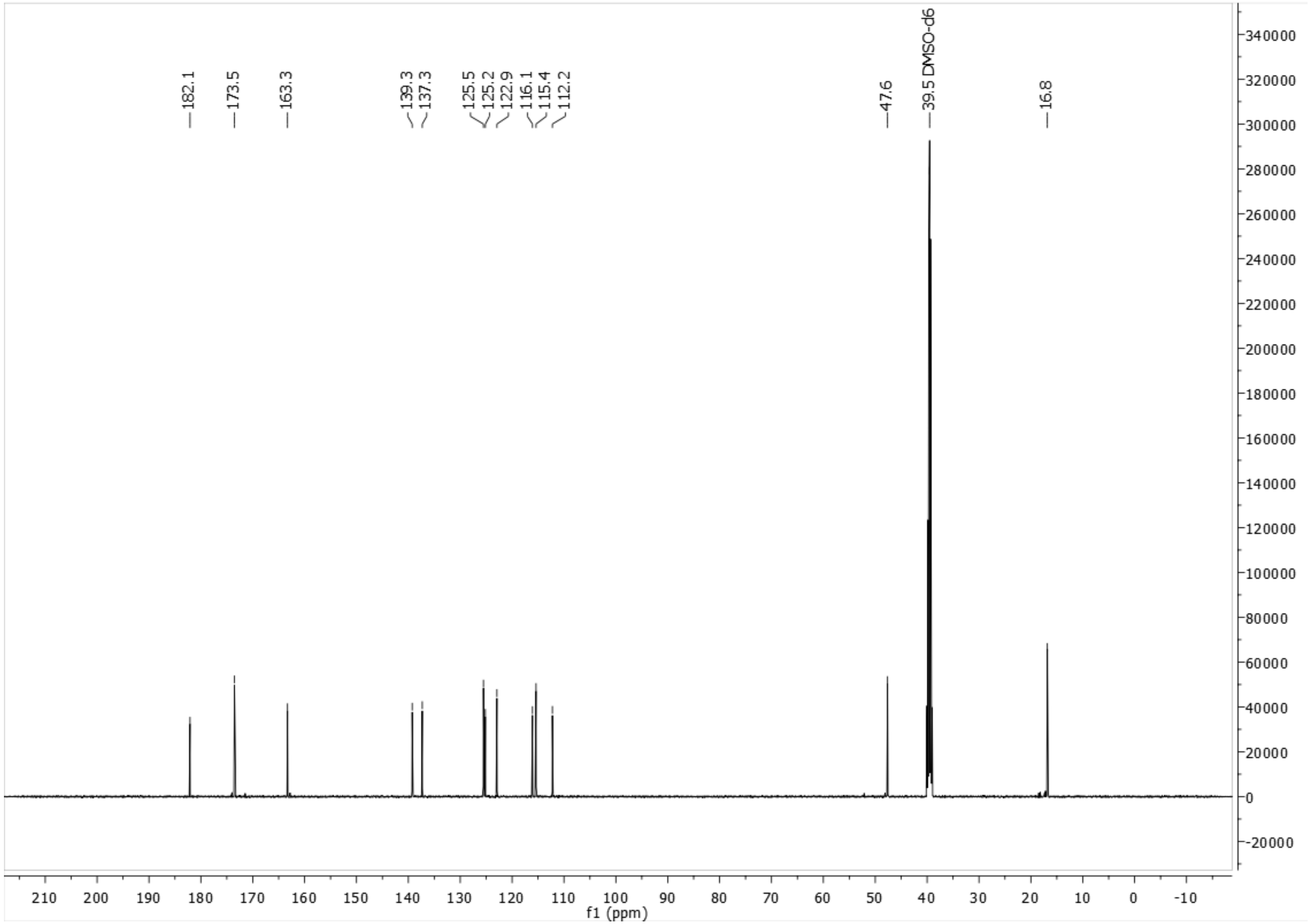

Figure S15. ^{13}C NMR spectrum for 6-bromoindolyl-3-glyoxyl-L-alanine (**27**) recorded in DMSO-d_6

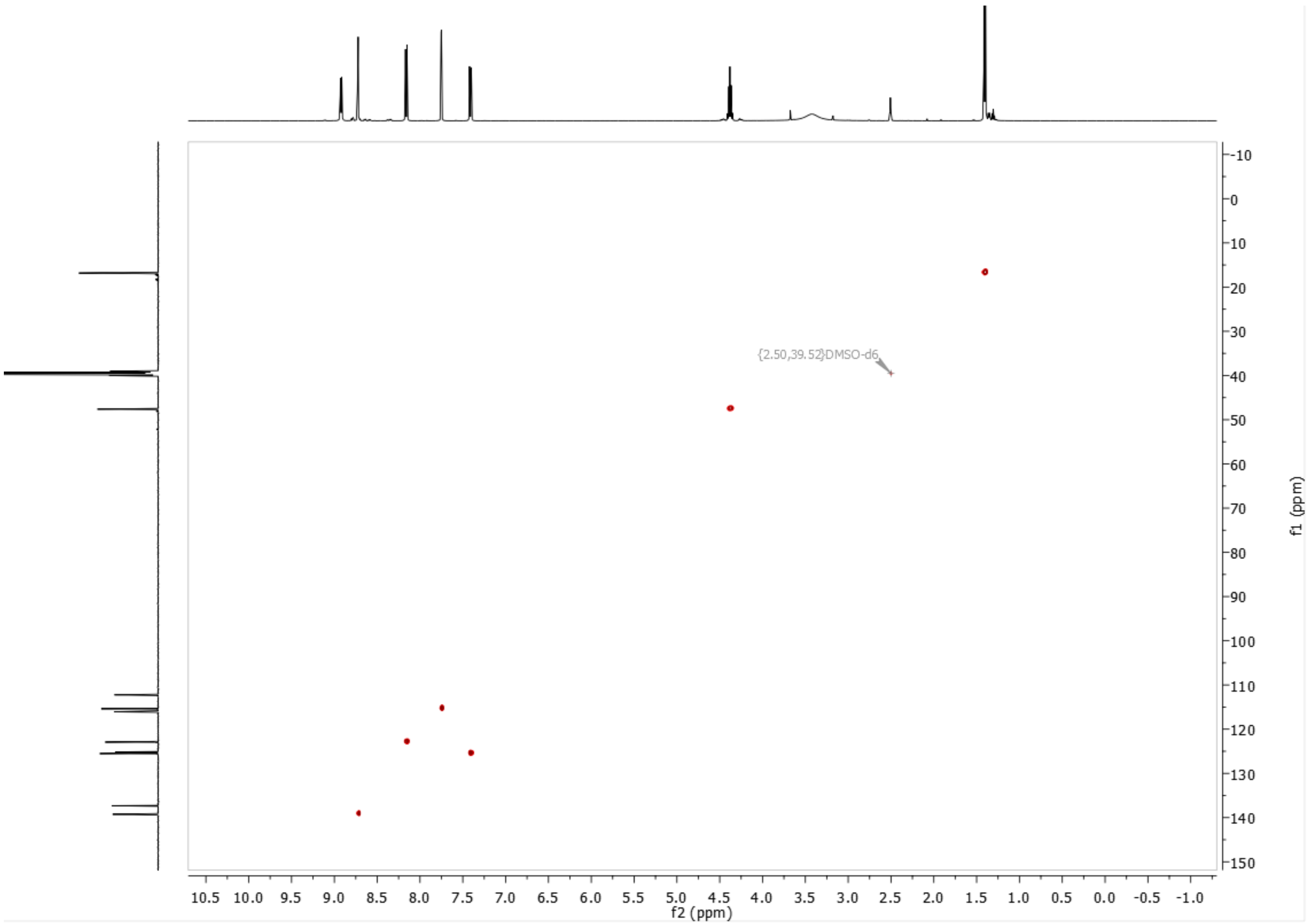

Figure S16. HSQC NMR spectrum for 6-bromoindolyl-3-glyoxyl-L-alanine (**27**) recorded in DMSO-d_6

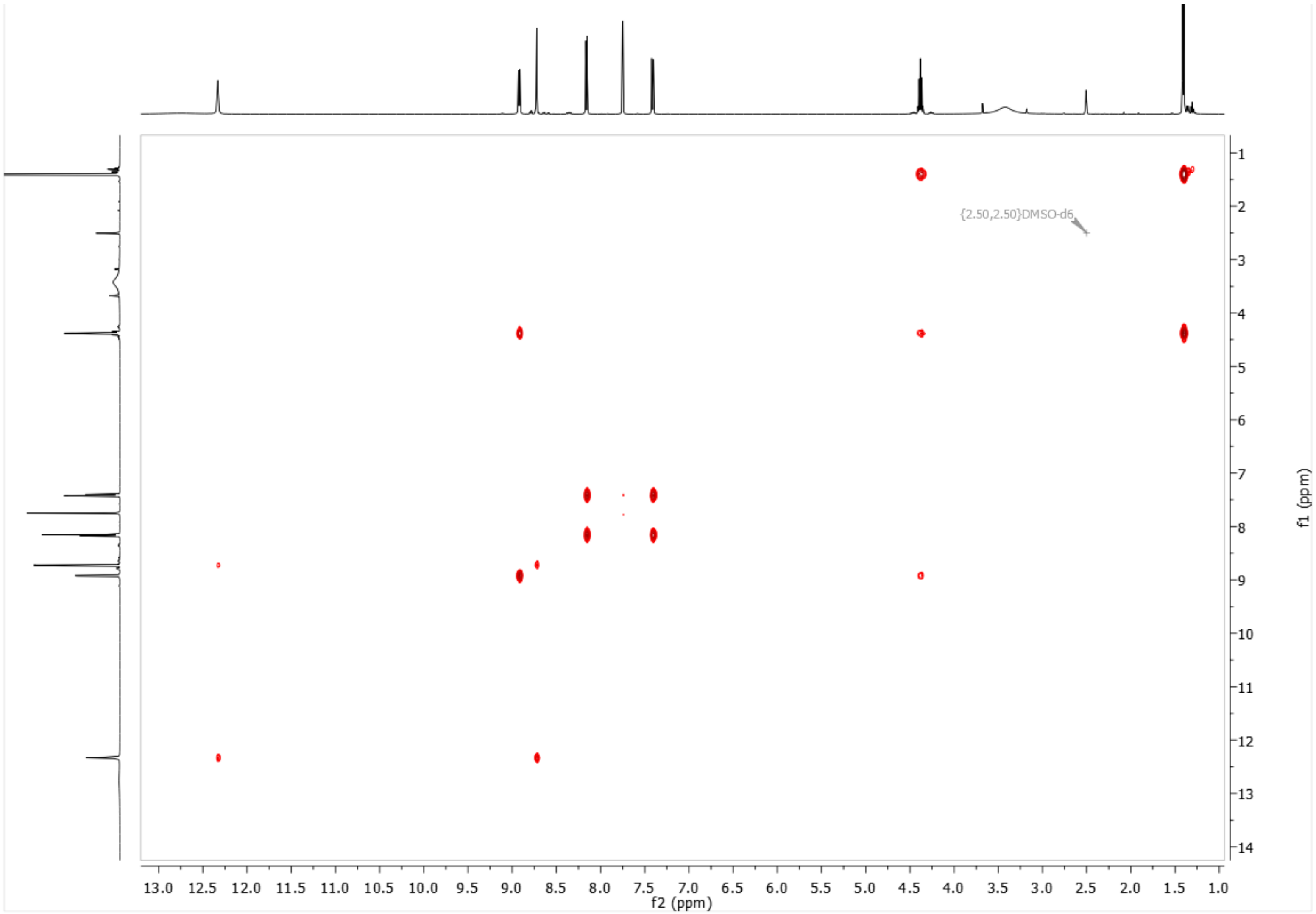

Figure S17. COSY NMR spectrum for 6-bromoindolyl-3-glyoxyl-L-alanine (**27**) recorded in DMSO-d_6

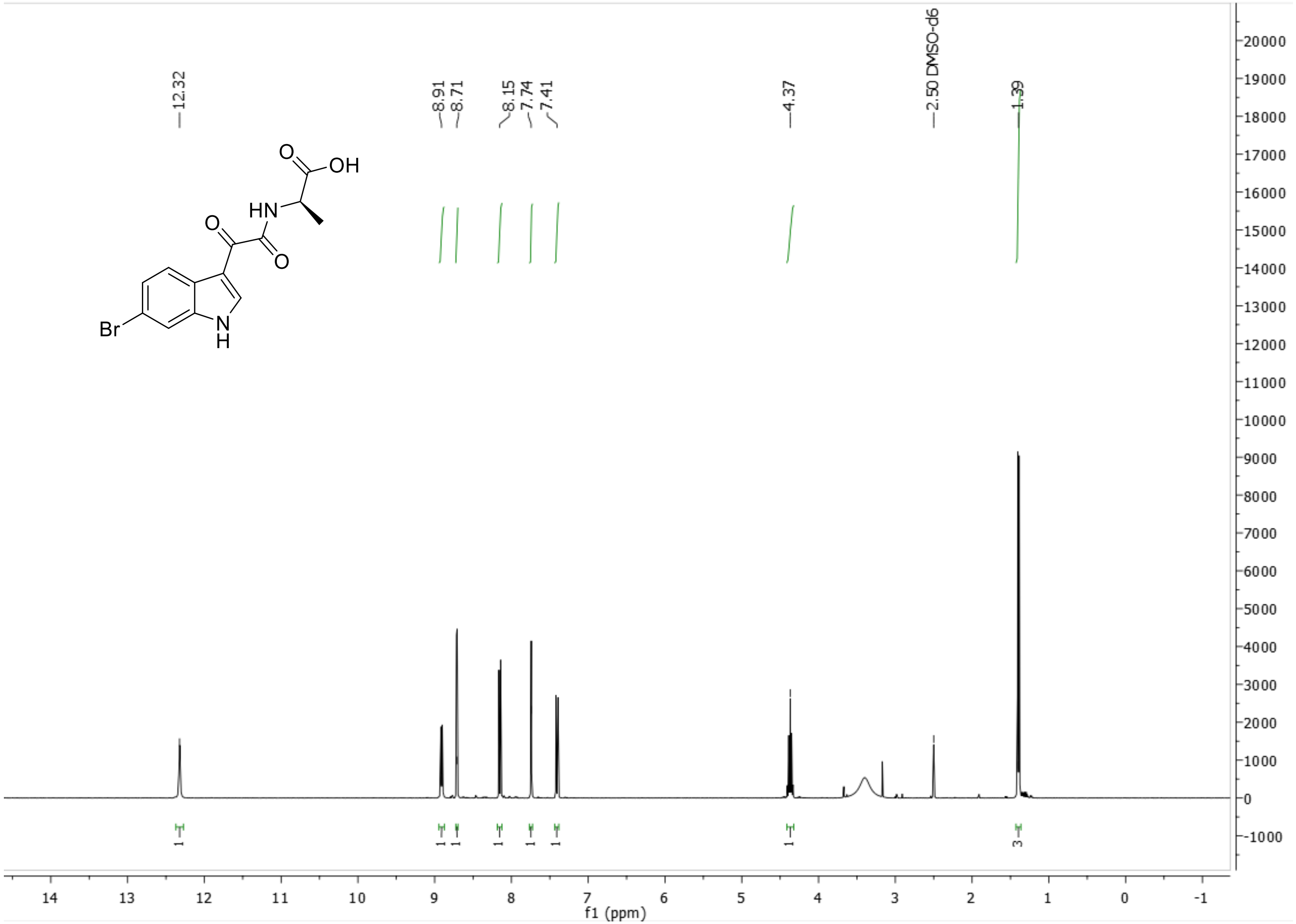

Figure S18. ^{1}H NMR spectrum for 6-bromoindolyl-3-glyoxyl-D-alanine (**28**) recorded in DMSO-d_6

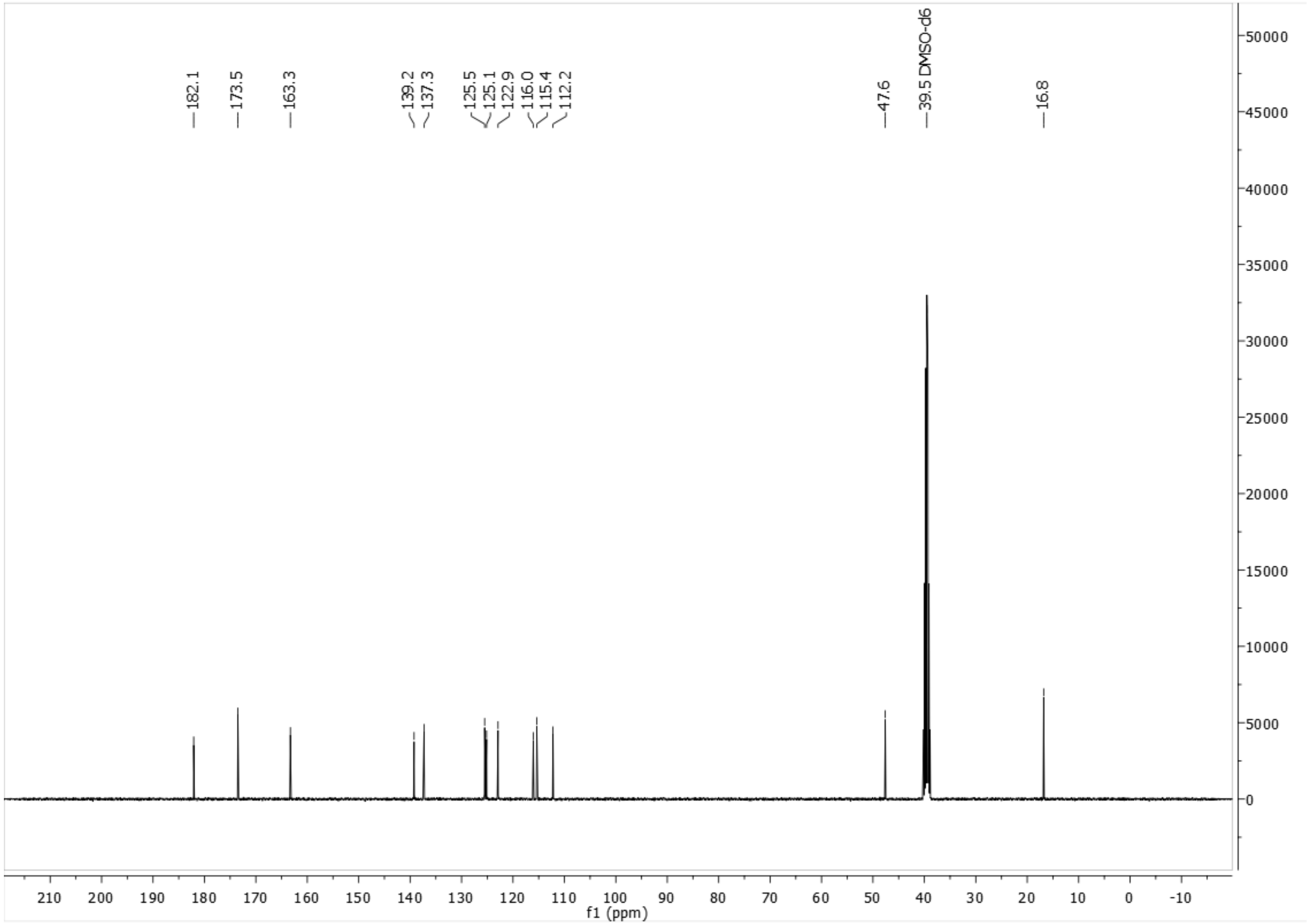

Figure S19. ^{13}C NMR spectrum for 6-bromoindolyl-3-glyoxyl-D-alanine (**28**) recorded in DMSO-d_6

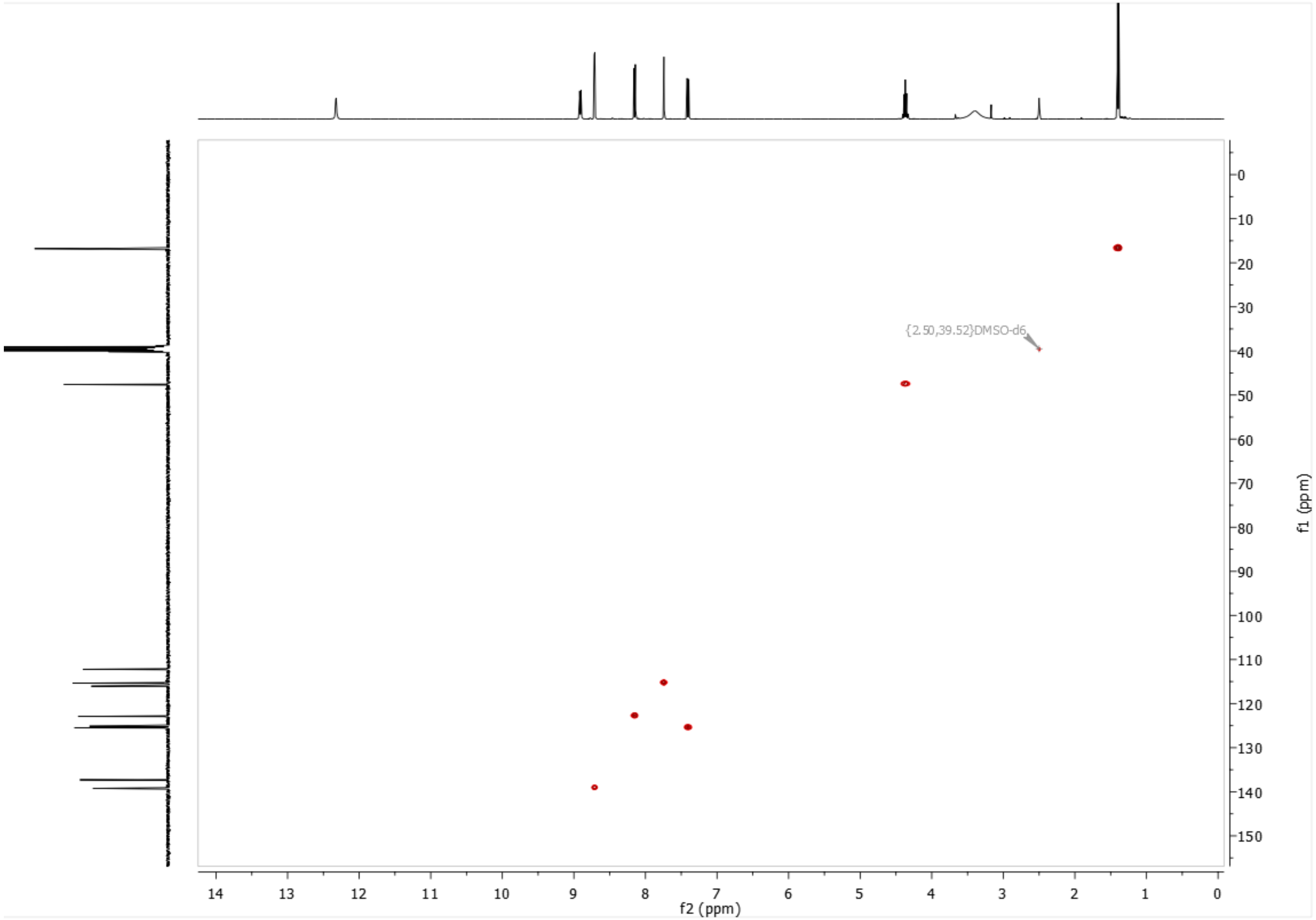

Figure S20. HSQC NMR spectrum for 6-bromoindolyl-3-glyoxyl-D-alanine (**28**) recorded in DMSO-d_6

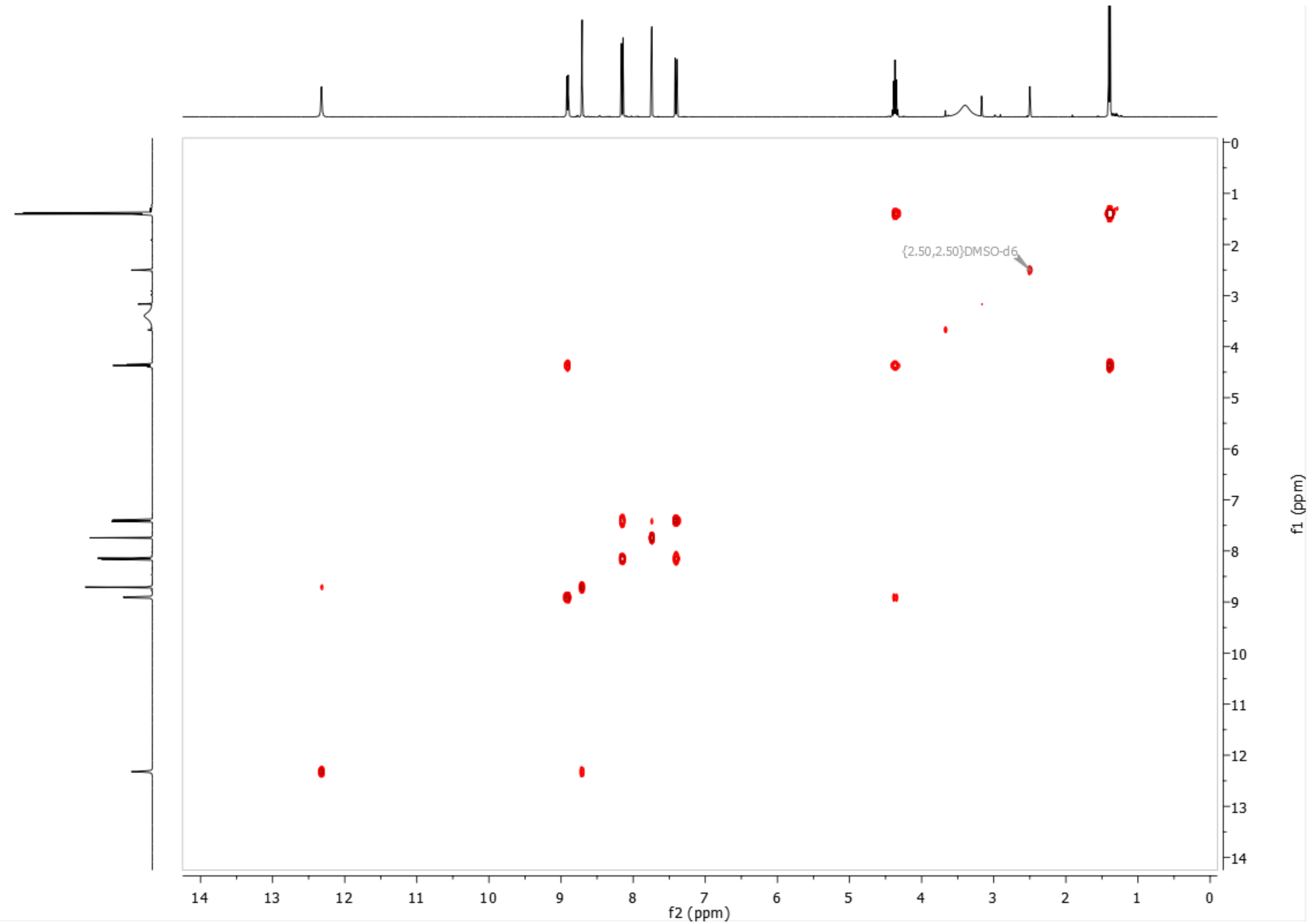

Figure S21. COSY NMR spectrum for 6-bromoindolyl-3-glyoxyl-D-alanine (**28**) recorded in DMSO-d_6

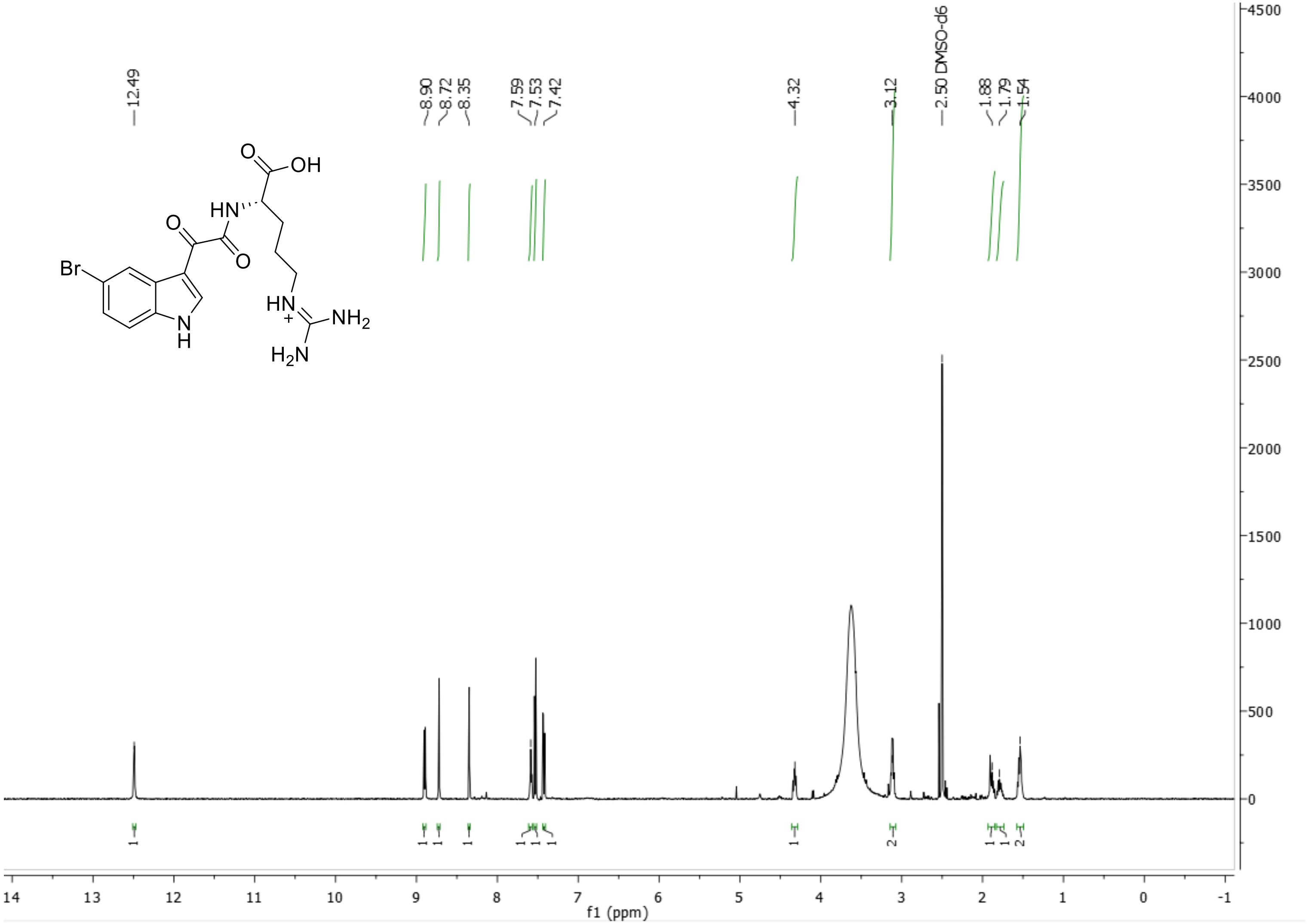

Figure S22. ^{1}H NMR spectrum for 5-bromoindolyl-3-glyoxyl-L-arginine (**29**) recorded in DMSO-d_6

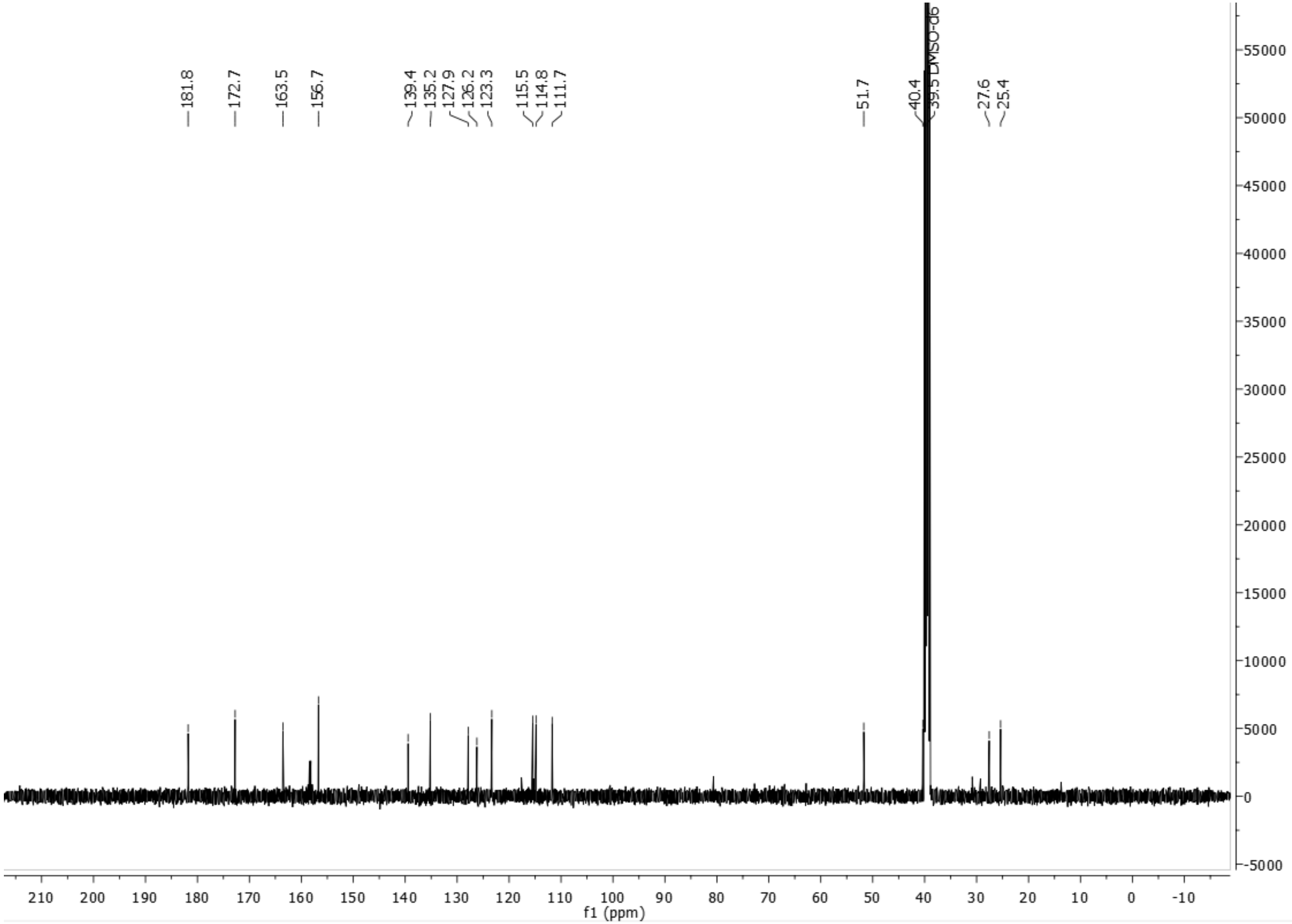

Figure S23. ^{13}C NMR spectrum for 5-bromoindolyl-3-glyoxyl-L-arginine (**29**) recorded in DMSO-d_6

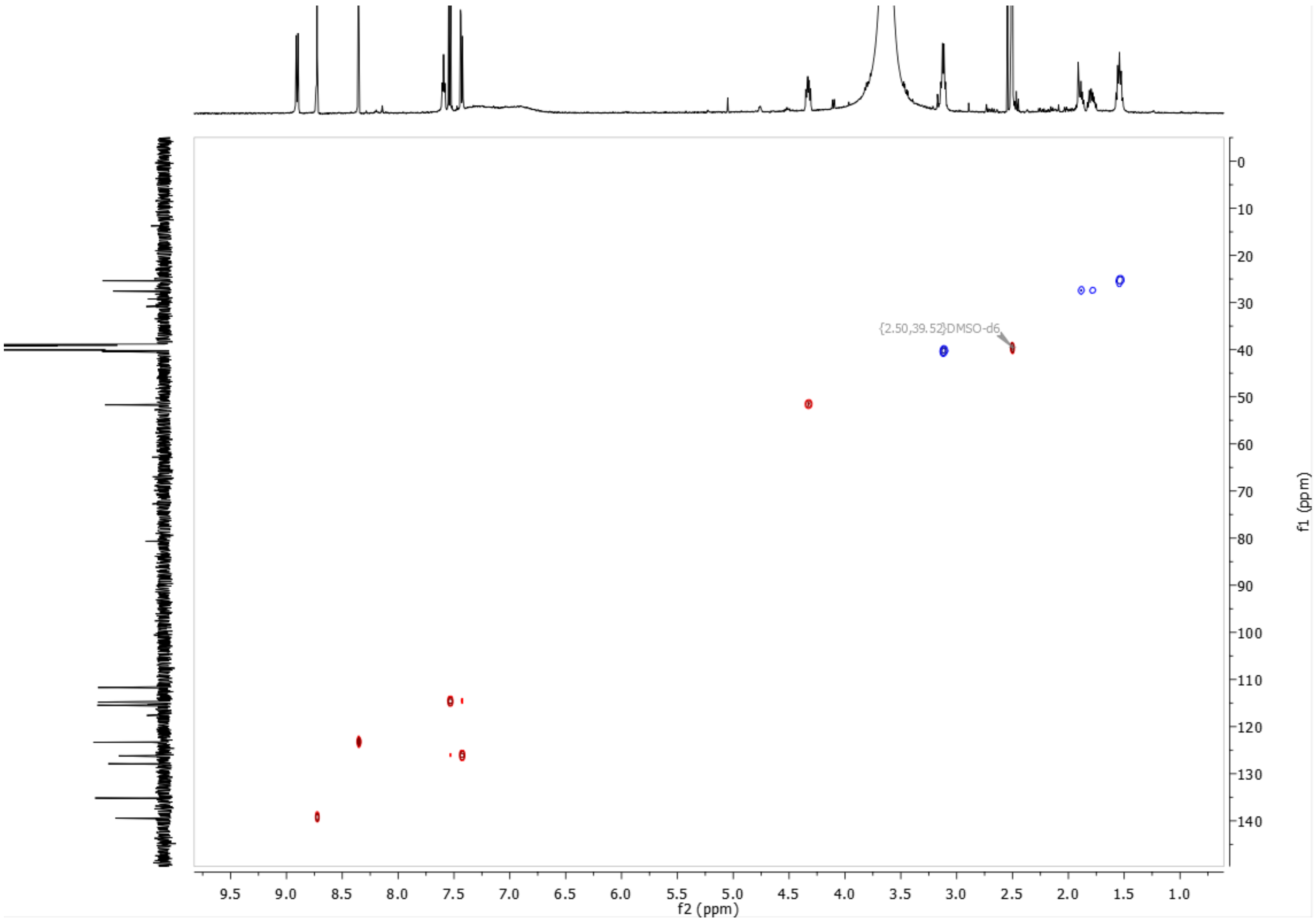

Figure S24. HSQC NMR spectrum for 5-bromoindolyl-3-glyoxyl-L-arginine (**29**) recorded in DMSO-d_6

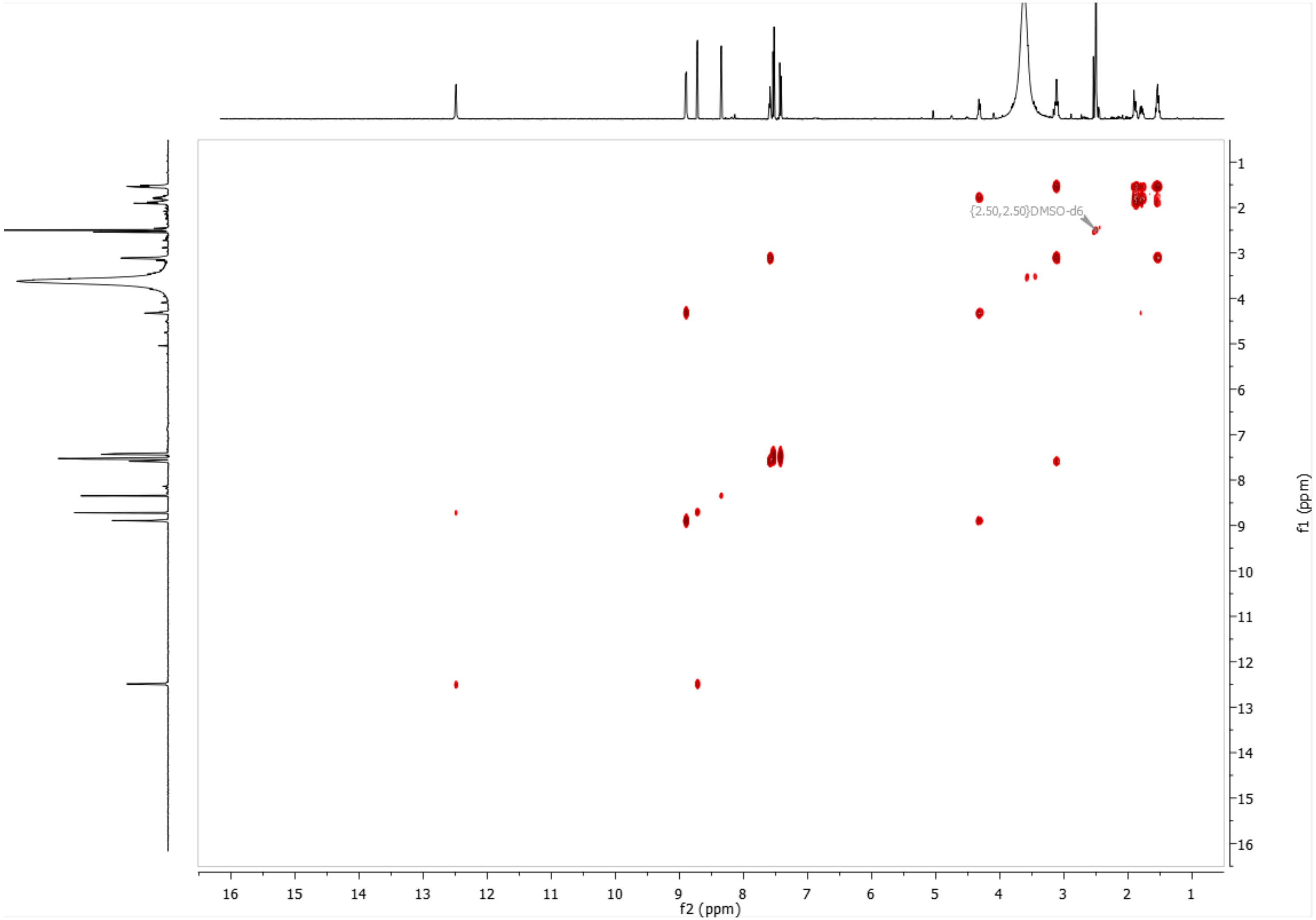

Figure S25. COSY NMR spectrum for 5-bromoindolyl-3-glyoxyl-L-arginine (**29**) recorded in DMSO-d_6

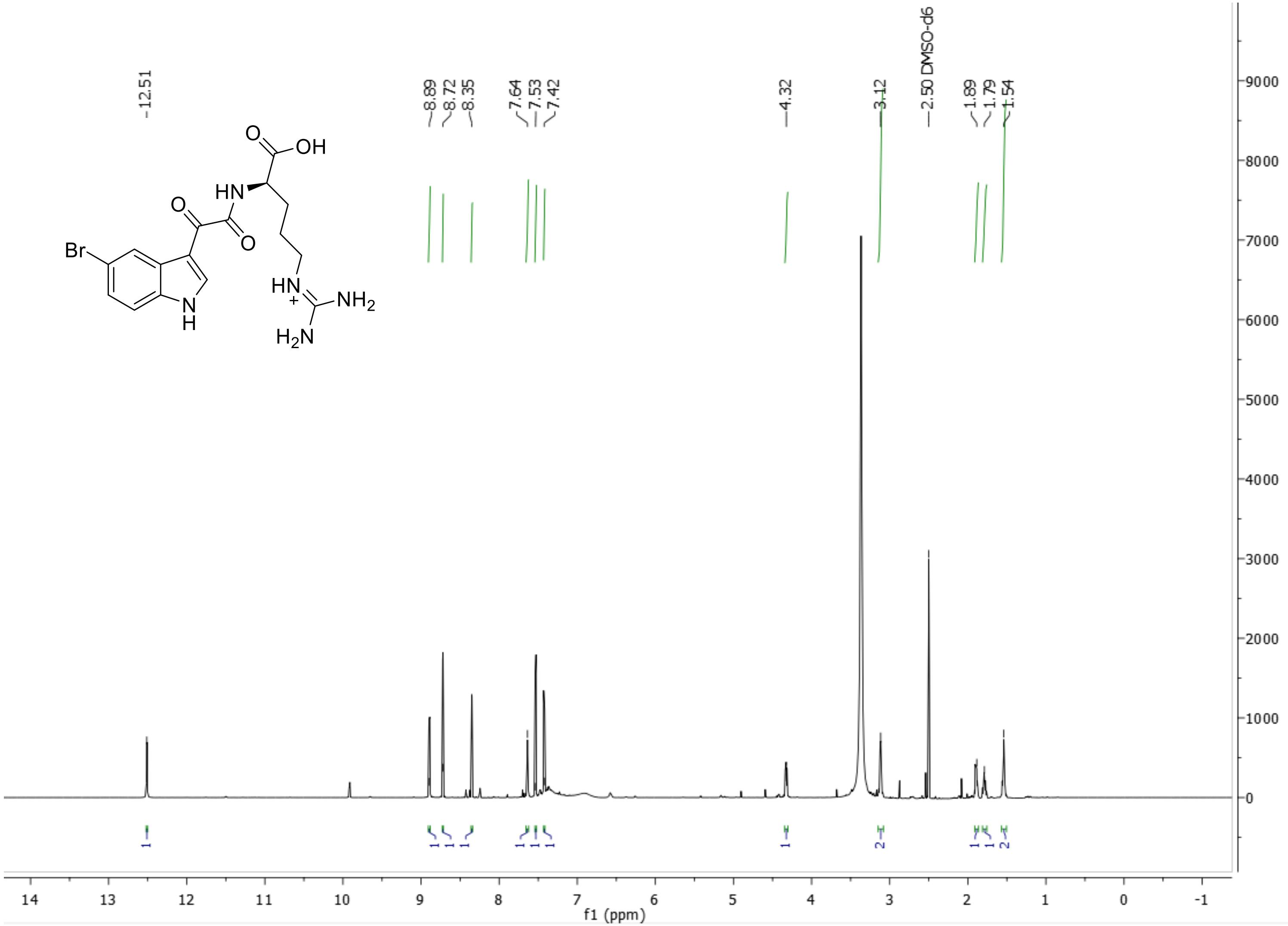

Figure S26. ^{1}H NMR spectrum for 5-bromoindolyl-3-glyoxyl-D-arginine (**30**) recorded in DMSO-d_6

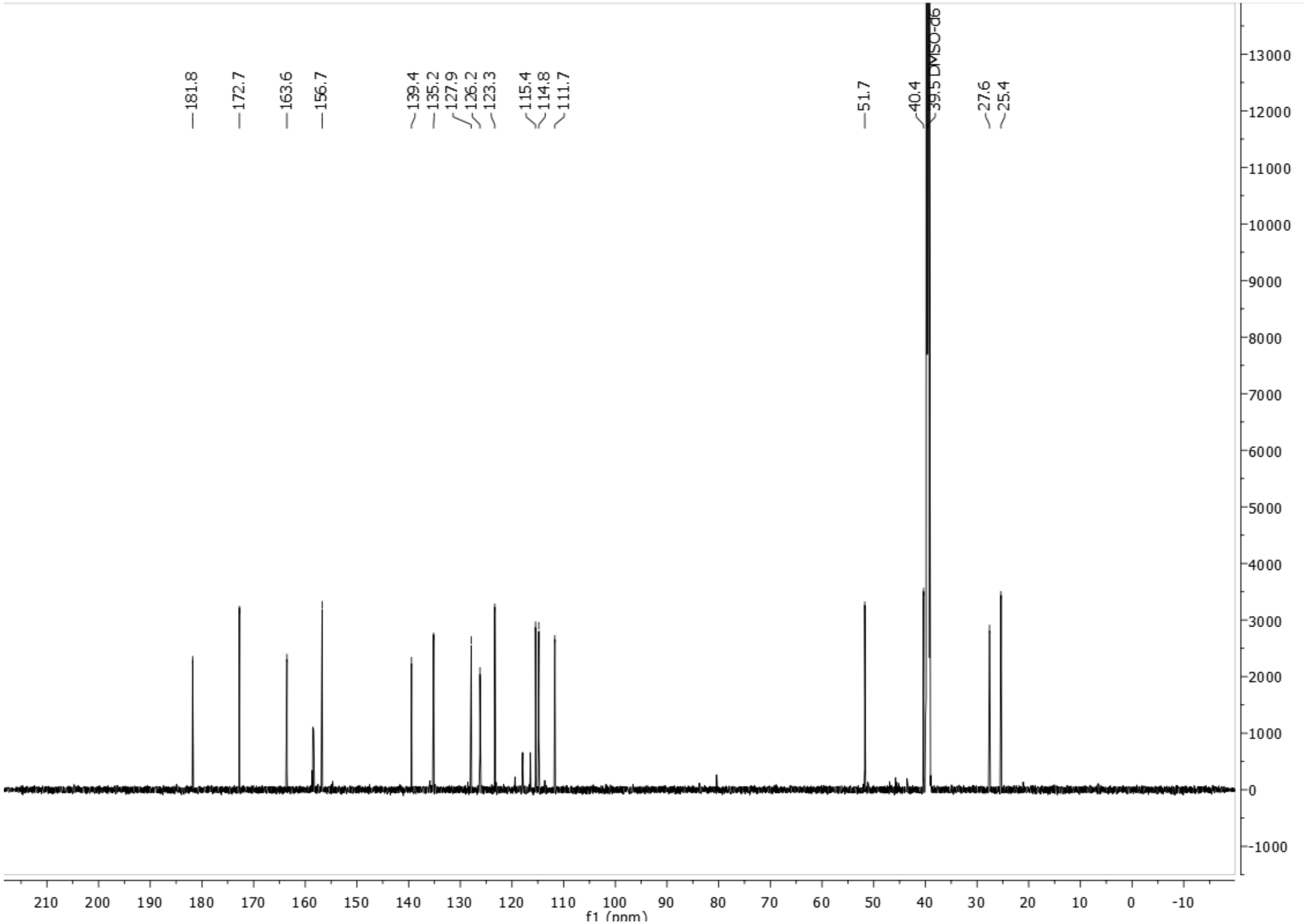

Figure S27. ^{13}C NMR spectrum for 5-bromoindolyl-3-glyoxyl-D-arginine (**30**) recorded in DMSO-d_6

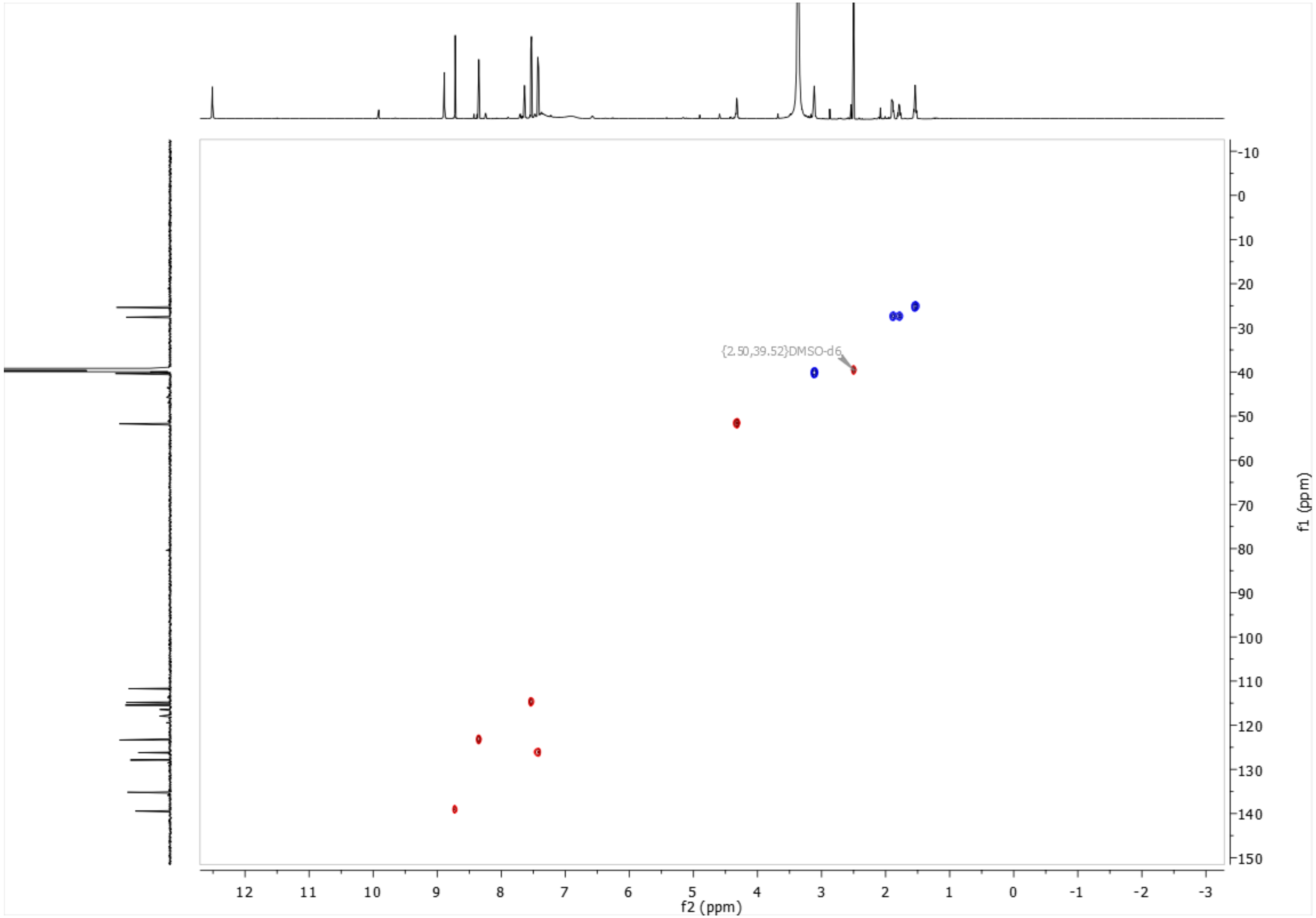

Figure S28. HSQC NMR spectrum for 5-bromoindolyl-3-glyoxyl-D-arginine (**30**) recorded in DMSO-d_6

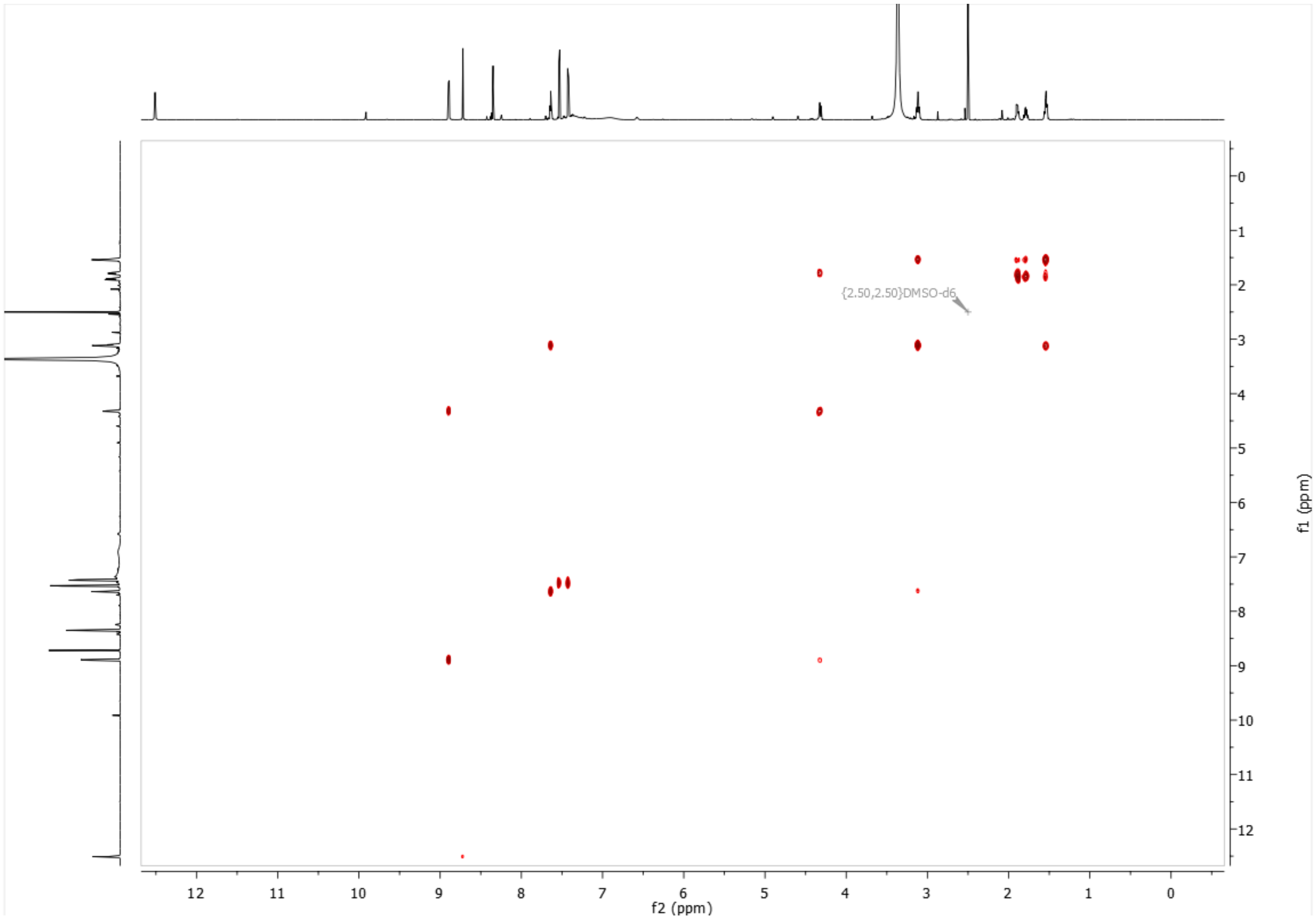

Figure S29. COSY NMR spectrum for 5-bromoindolyl-3-glyoxyl-D-arginine (**30**) recorded in DMSO-*d*$_6$

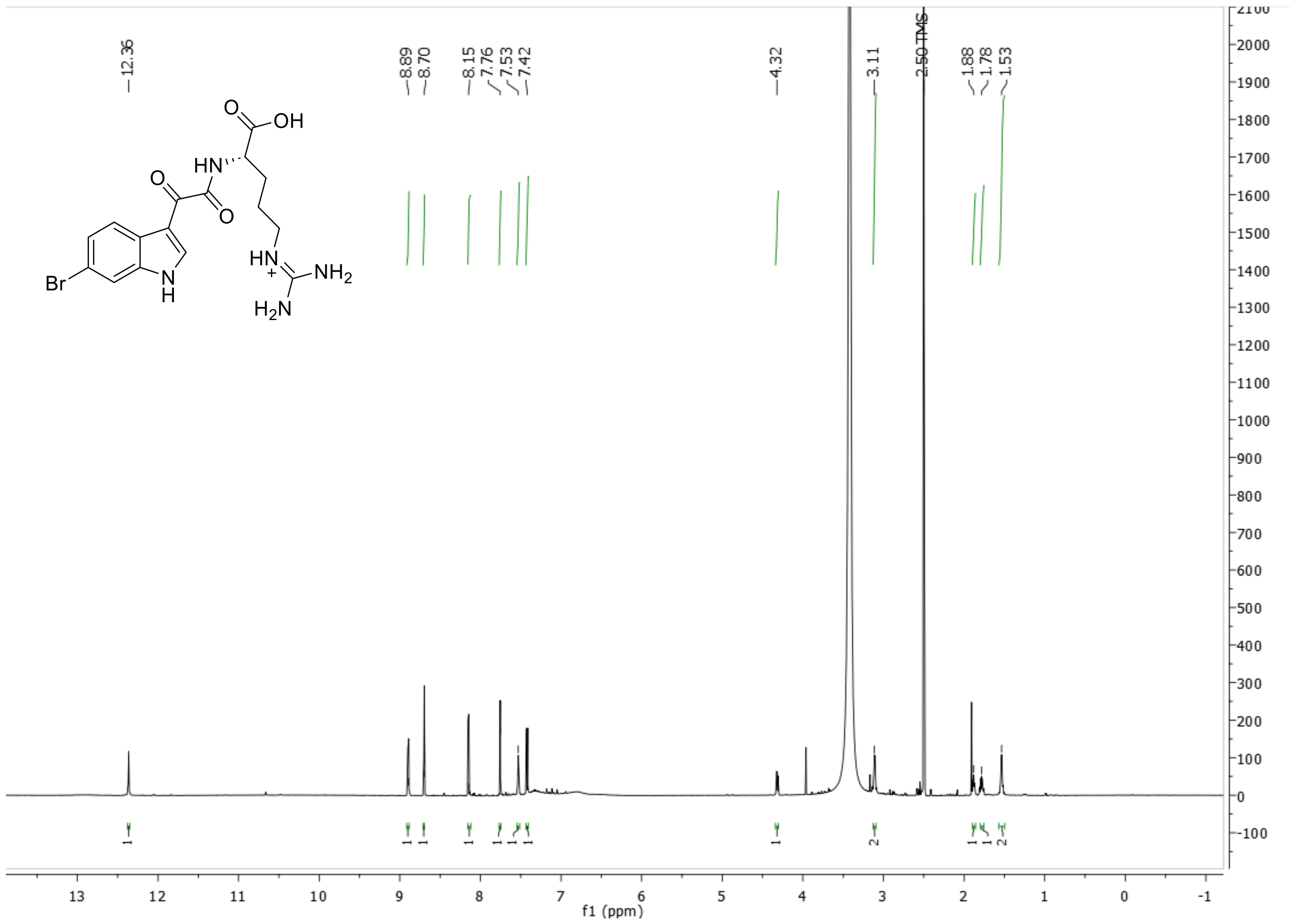

Figure S30. ^{1}H NMR spectrum for 6-bromoindolyl-3-glyoxyl-L-arginine (**31**) recorded in DMSO-d_6

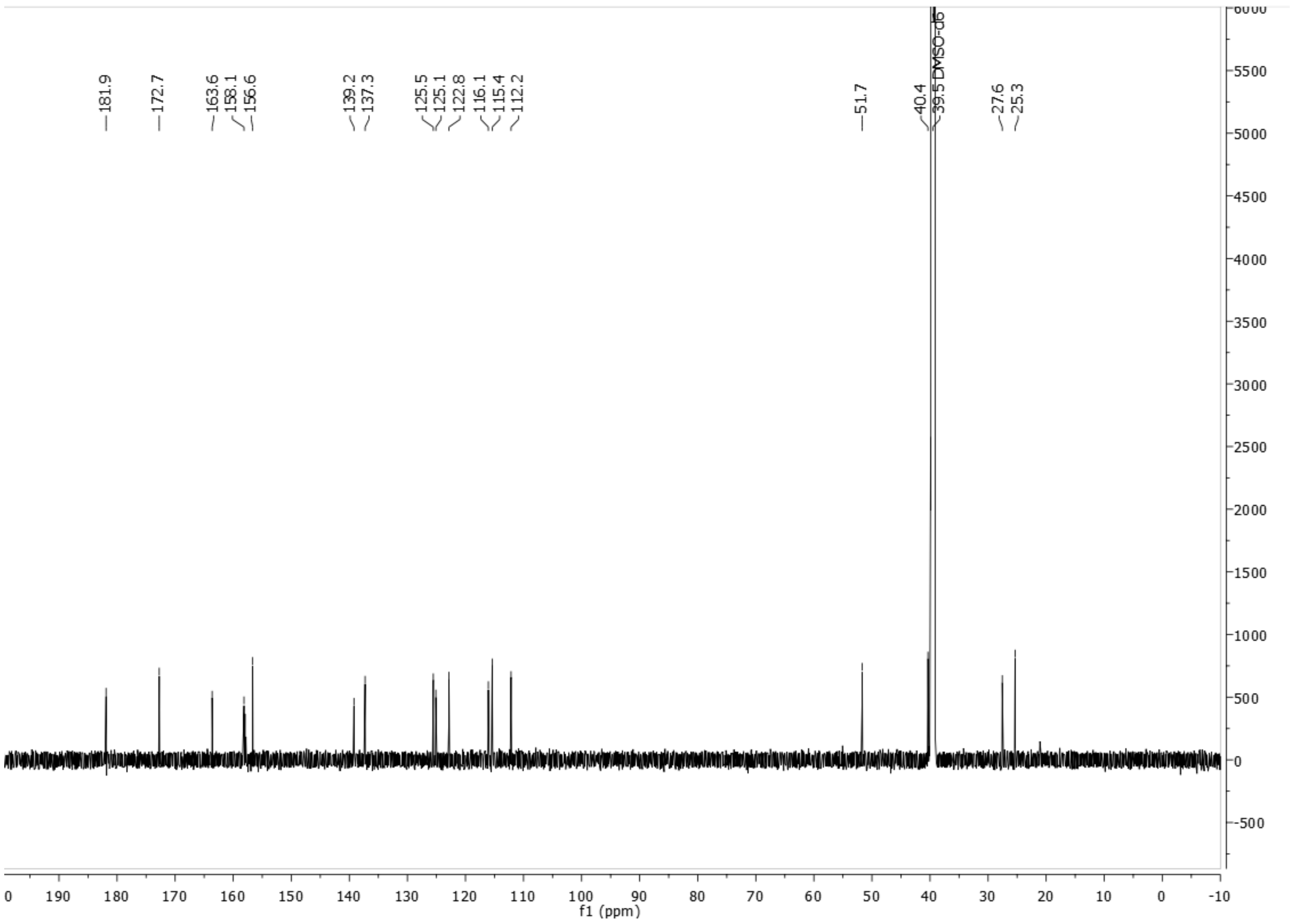

Figure S31. ^{13}C NMR spectrum for 6-bromoindolyl-3-glyoxyl-L-arginine (**31**) recorded in DMSO-d_6

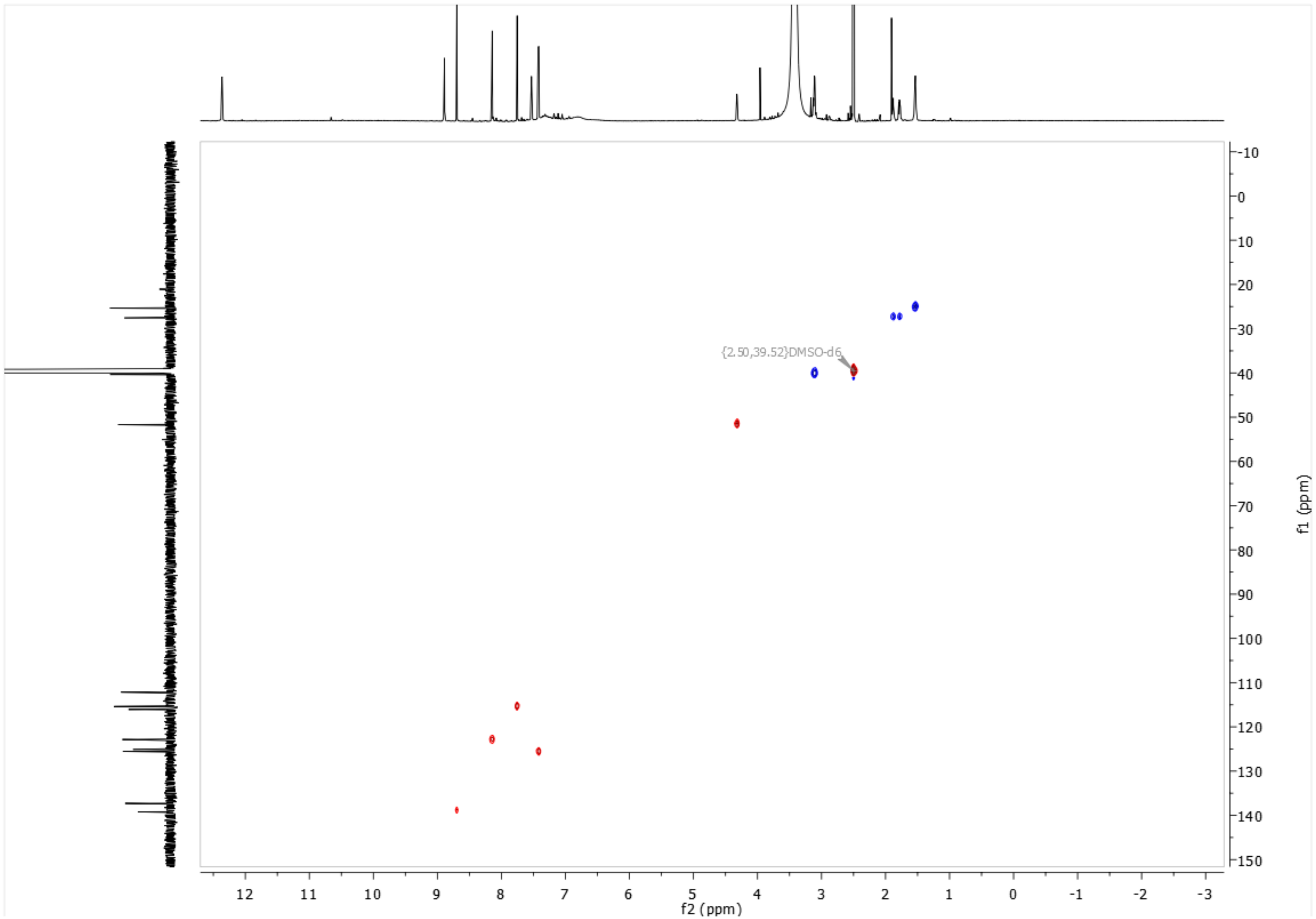

Figure S32. HSQC NMR spectrum for 6-bromoindolyl-3-glyoxyl-L-arginine (**31**) recorded in DMSO-d_6

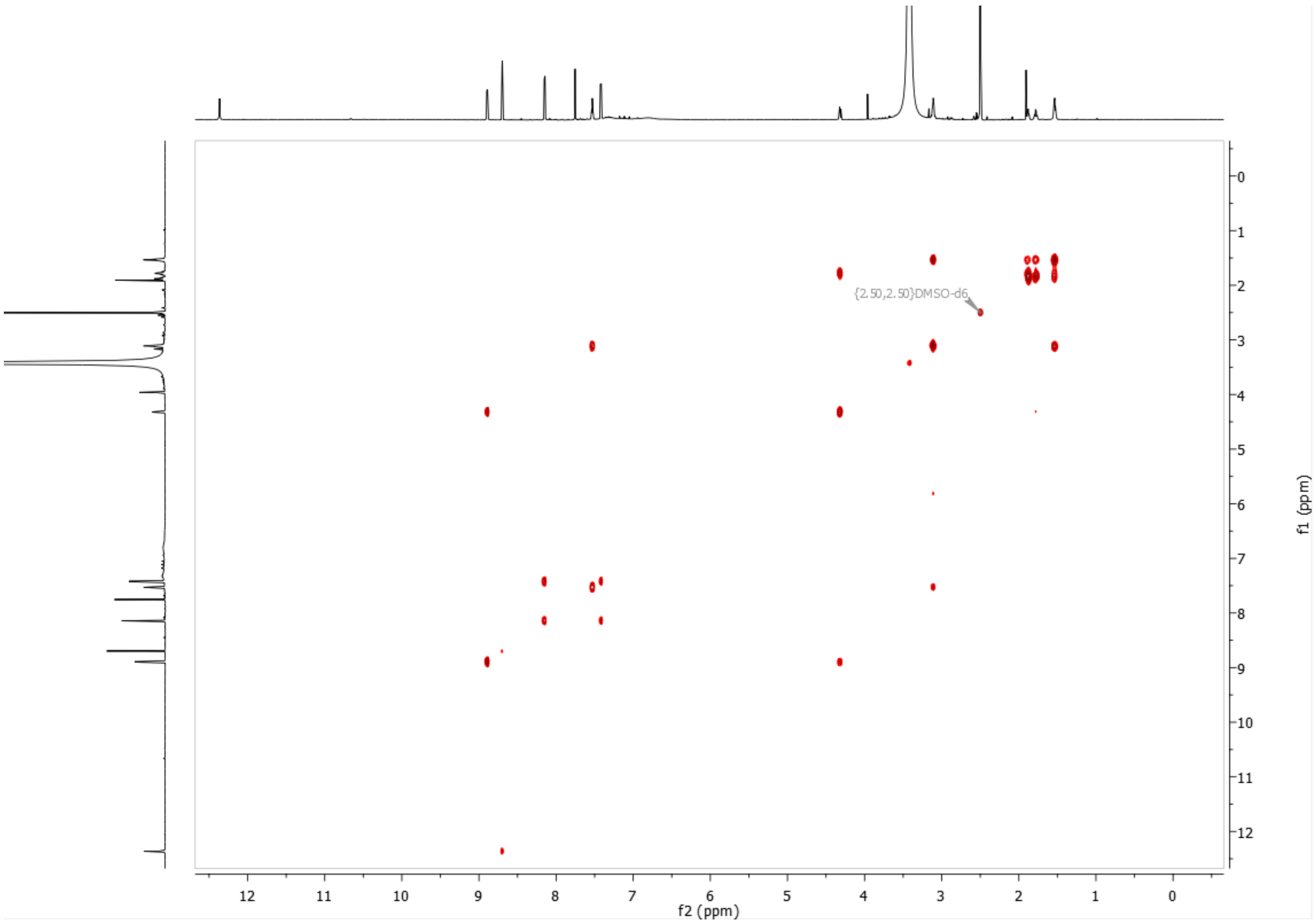

Figure S33. COSY NMR spectrum for 6-bromoindolyl-3-glyoxyl-L-arginine (**31**) recorded in DMSO-d_6

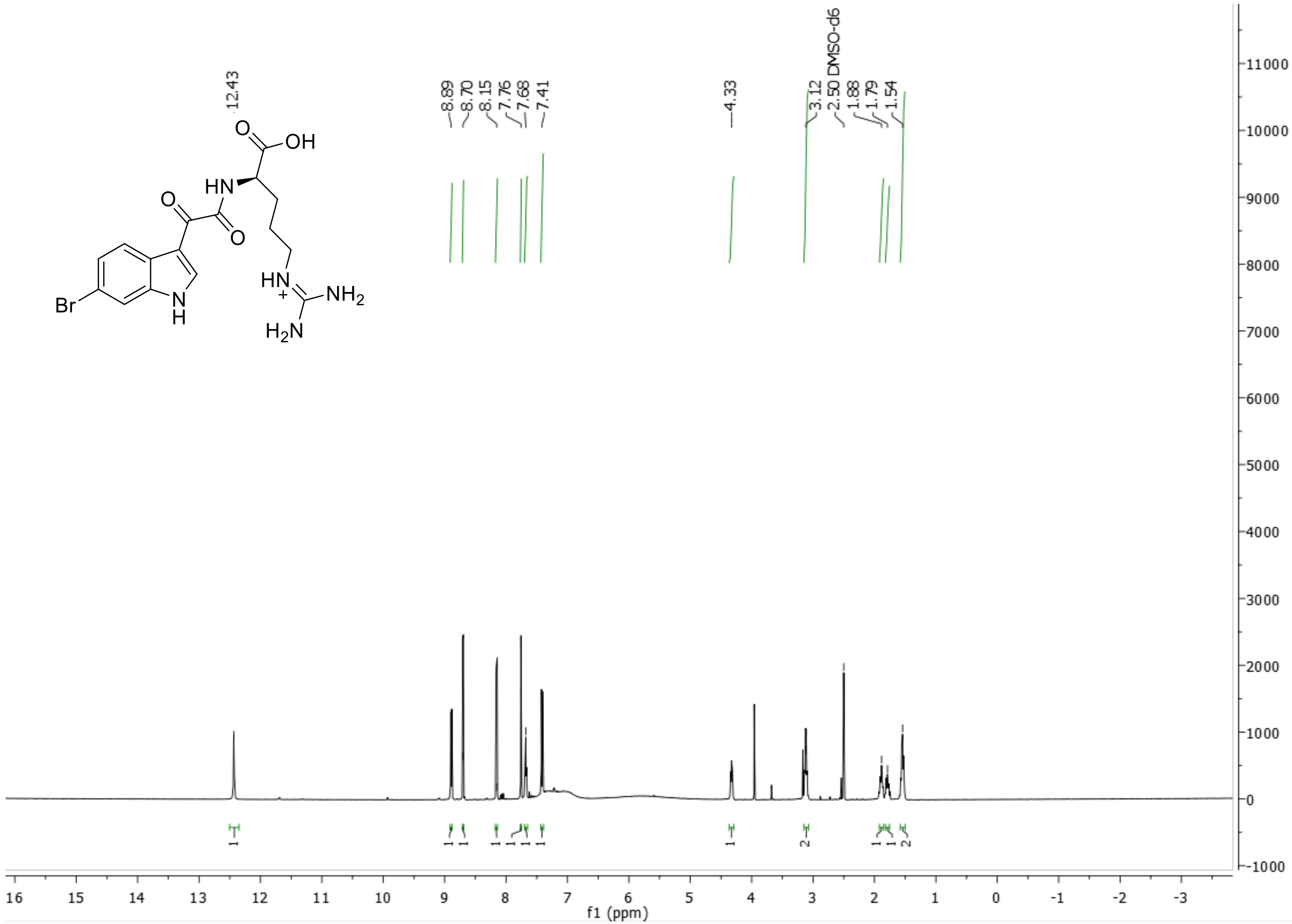

Figure S34. ^{1}H NMR spectrum for 6-bromoindolyl-3-glyoxyl-D-arginine (**32**) recorded in DMSO-d_6

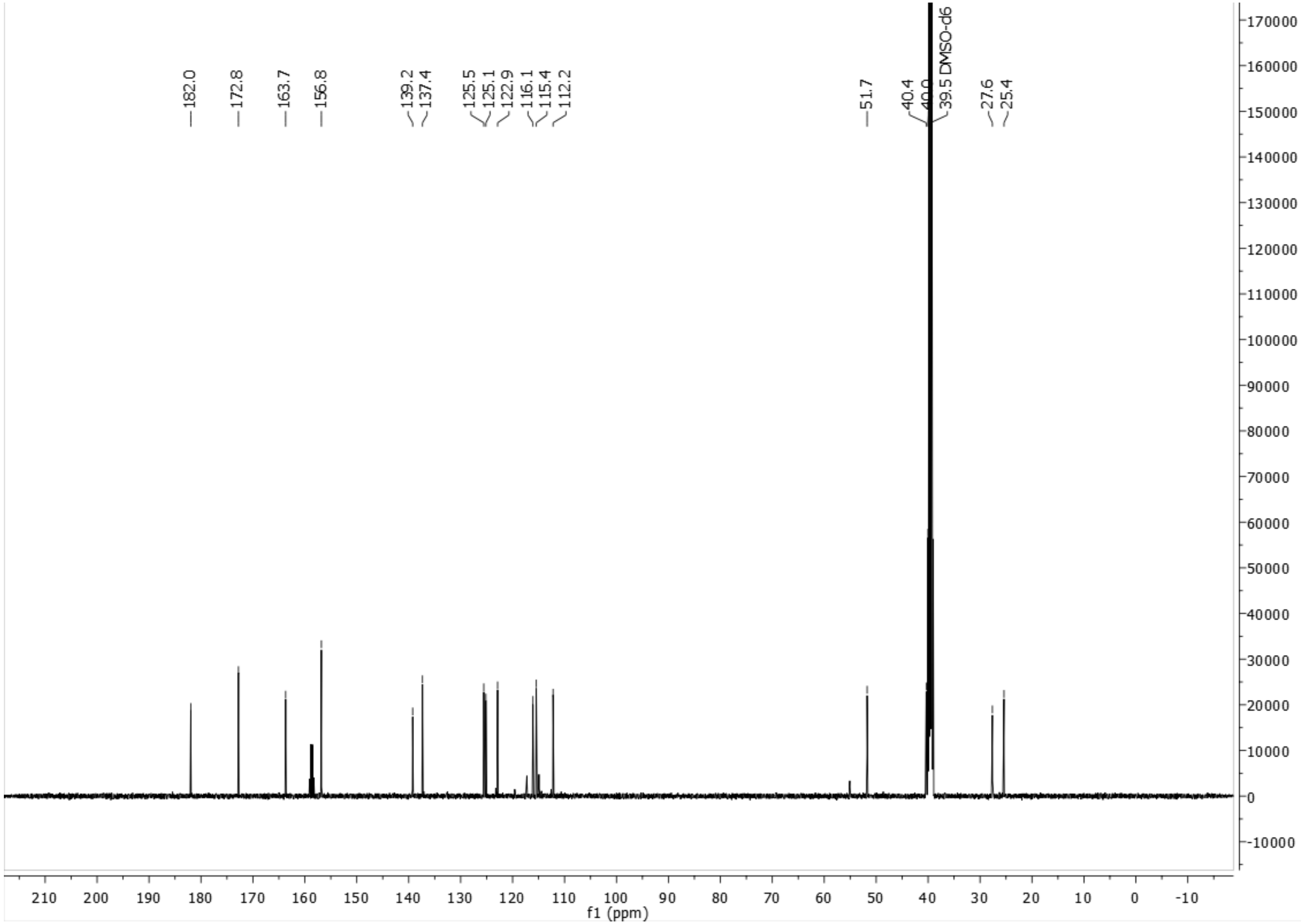

Figure S35. ^{13}C NMR spectrum for 6-bromoindolyl-3-glyoxyl-D-arginine (**32**) recorded in DMSO-d_6

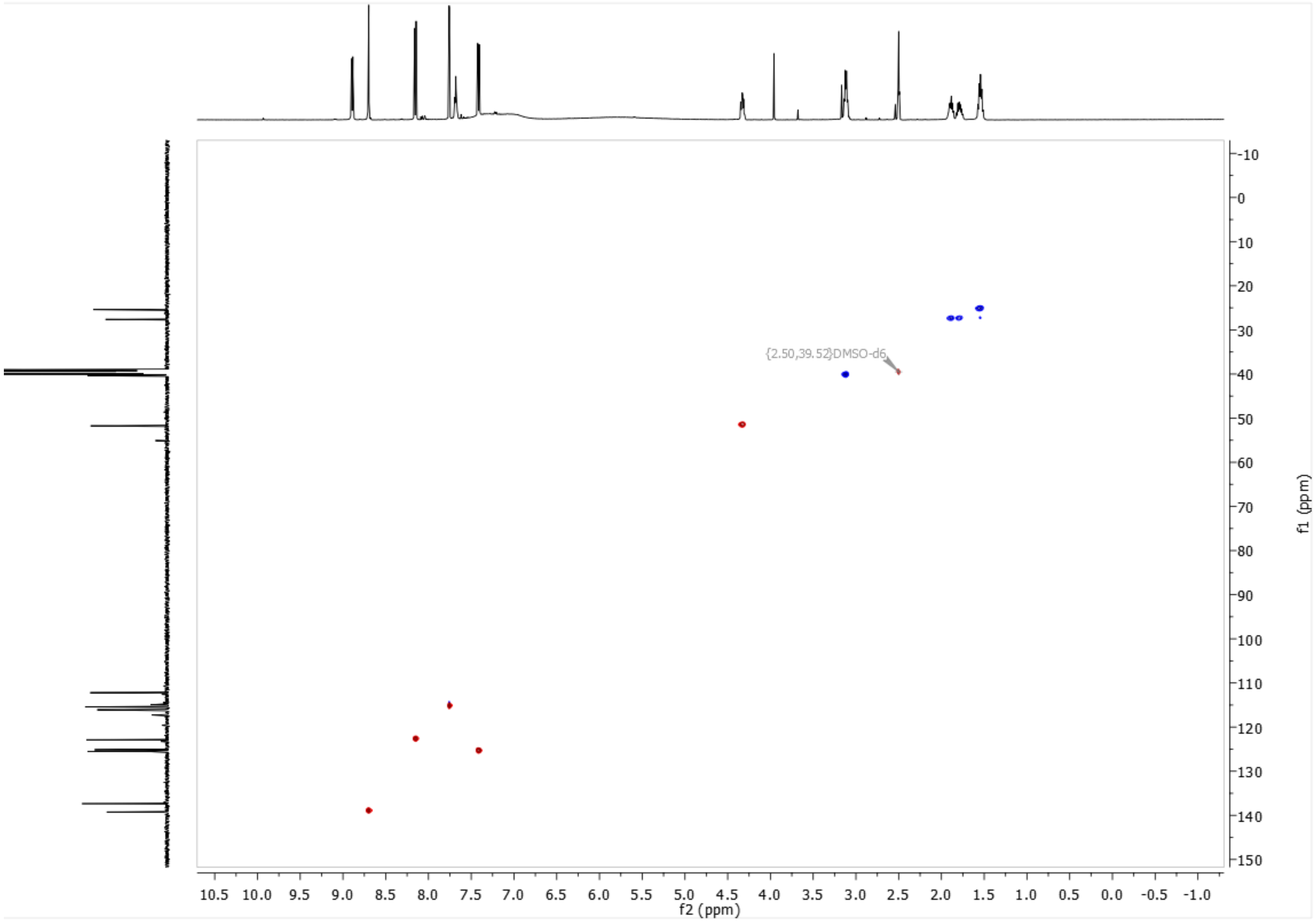

Figure S36. HSQC NMR spectrum for 6-bromoindolyl-3-glyoxyl-D-arginine (**32**) recorded in DMSO-d_6

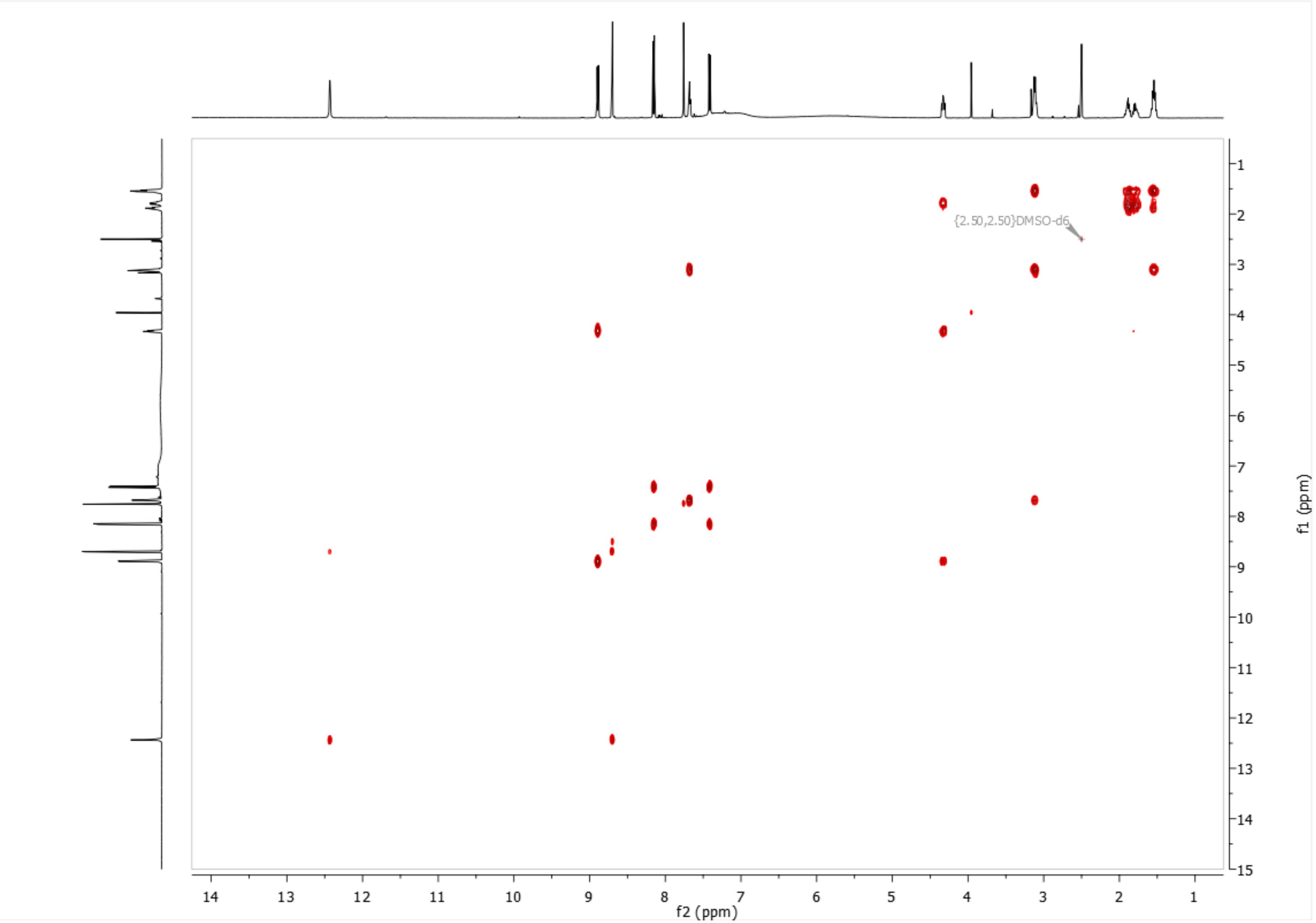

Figure S37. COSY NMR spectrum for 6-bromoindolyl-3-glyoxyl-D-arginine (**32**) recorded in DMSO-d_6

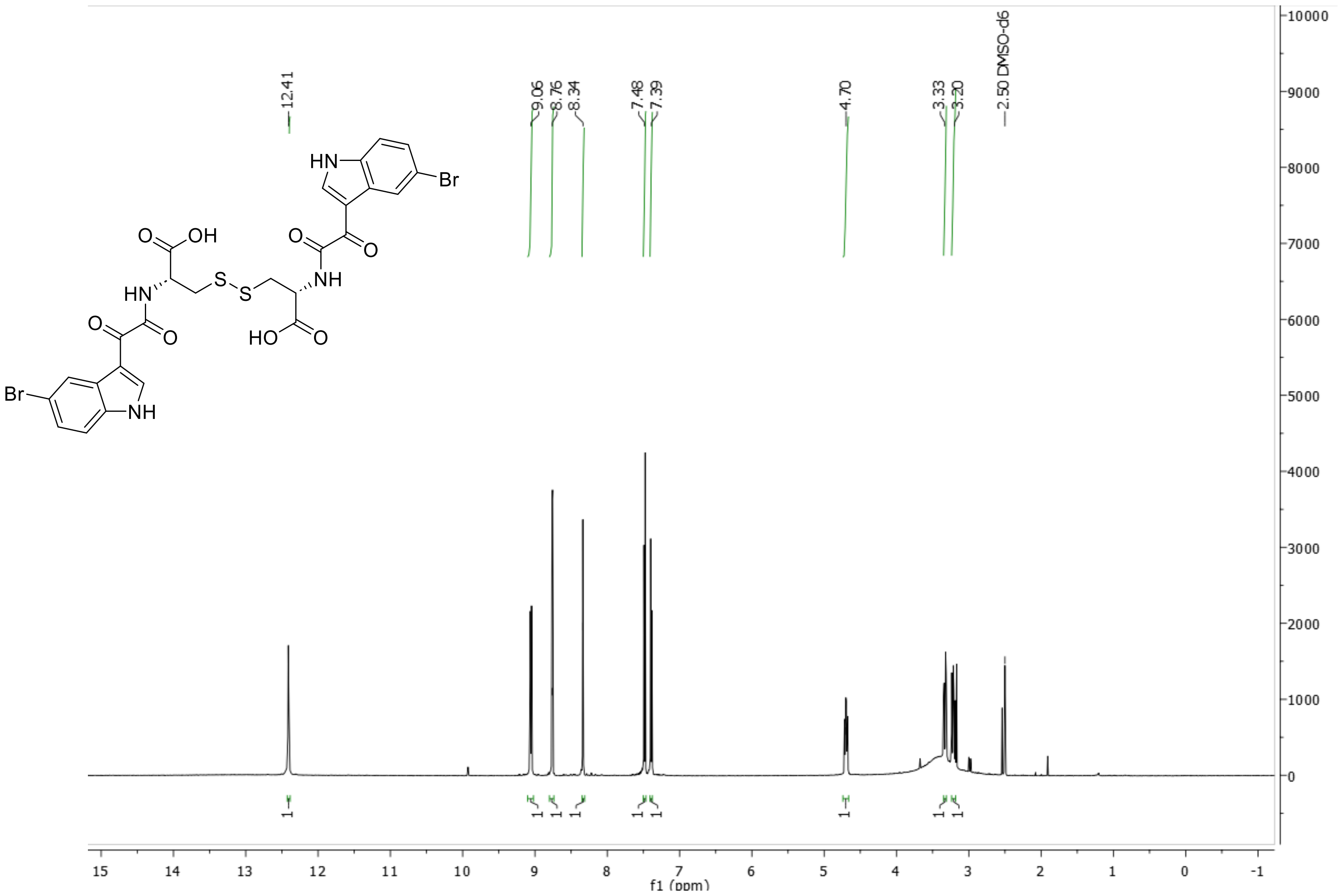

Figure S38. ^{1}H NMR spectrum for 5-bromoindolyl-3-glyoxyl-L-cystine dimer (**33**) recorded in DMSO-d_6

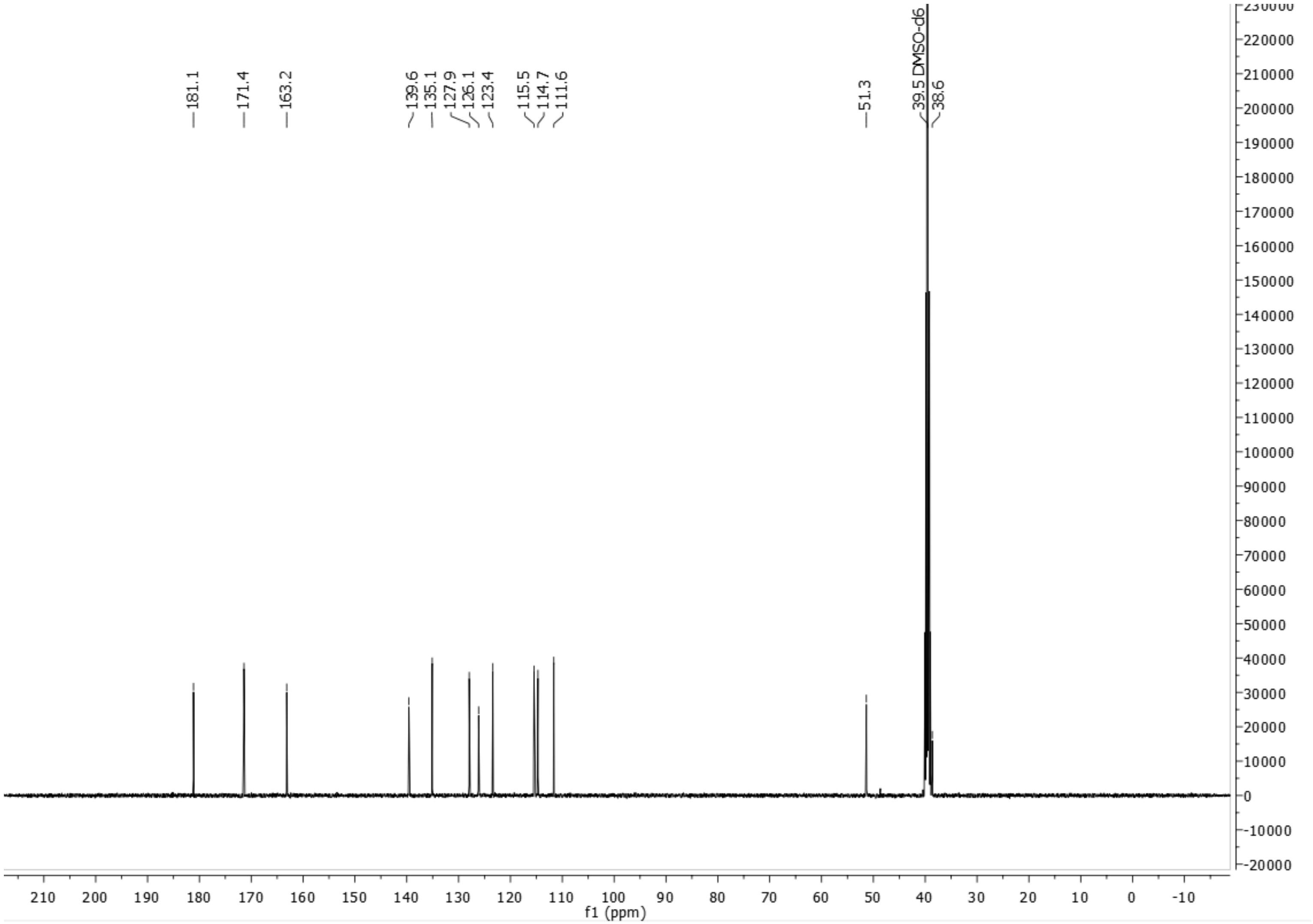

Figure S39. ^{13}C NMR spectrum for 5-bromoindolyl-3-glyoxyl-L-cystine dimer (**33**) recorded in DMSO-d_6

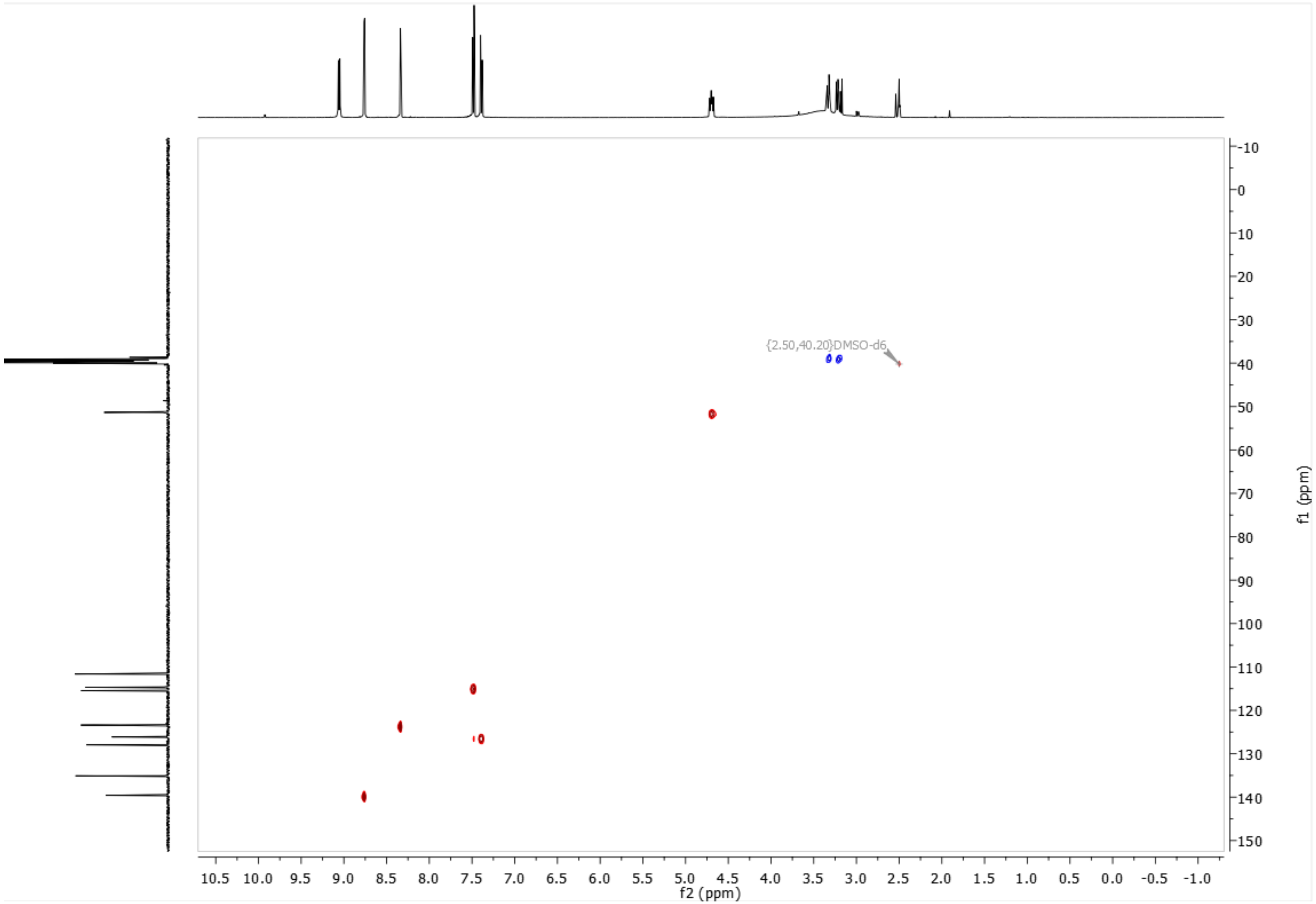

Figure S40. HSQC NMR spectrum for 5-bromoindolyl-3-glyoxyl-L-cystine dimer (**33**) recorded in DMSO-d_6

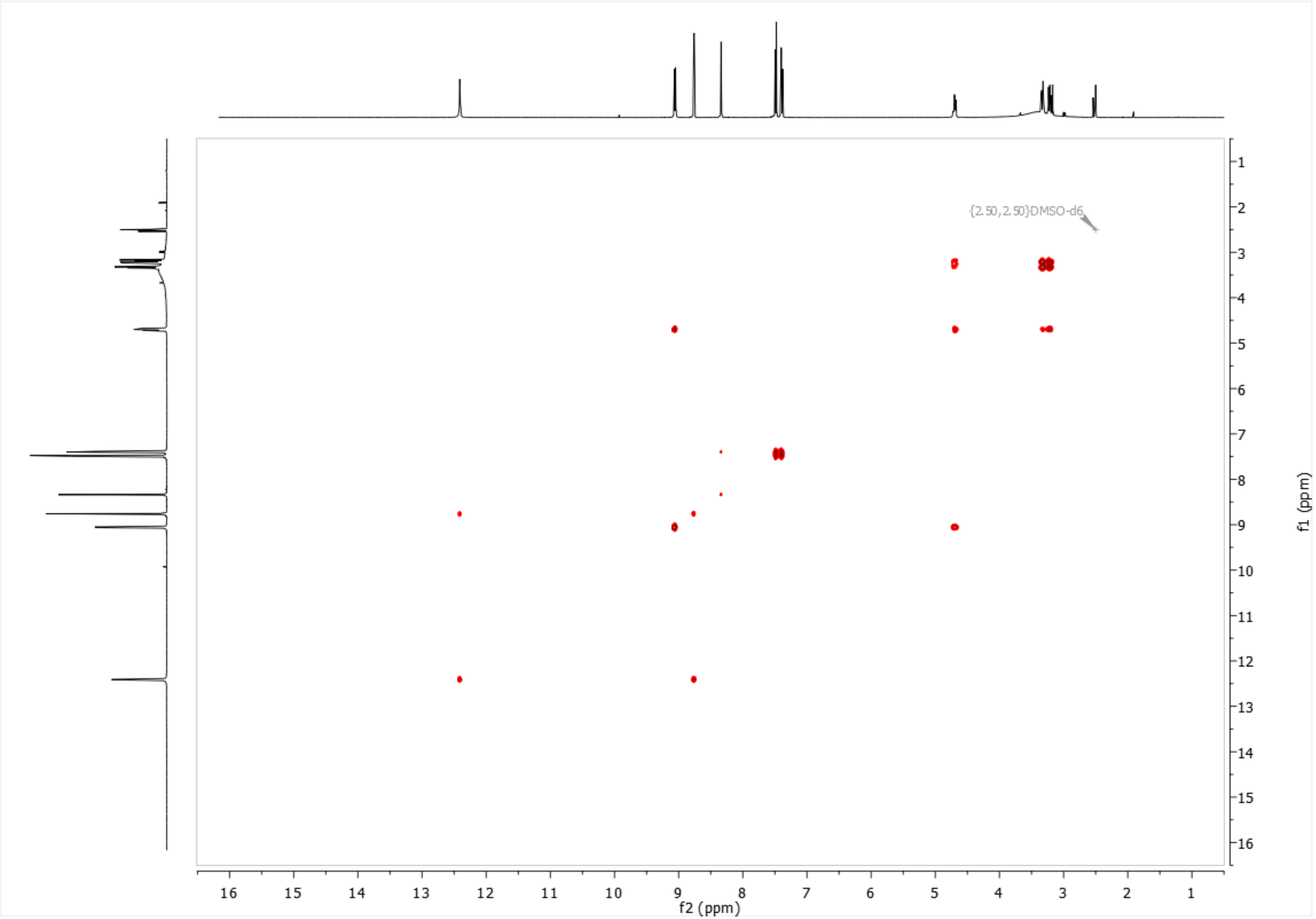

Figure S41. COSY NMR spectrum for 5-bromoindolyl-3-glyoxyl-L-cystine dimer (**33**) recorded in DMSO-d_6

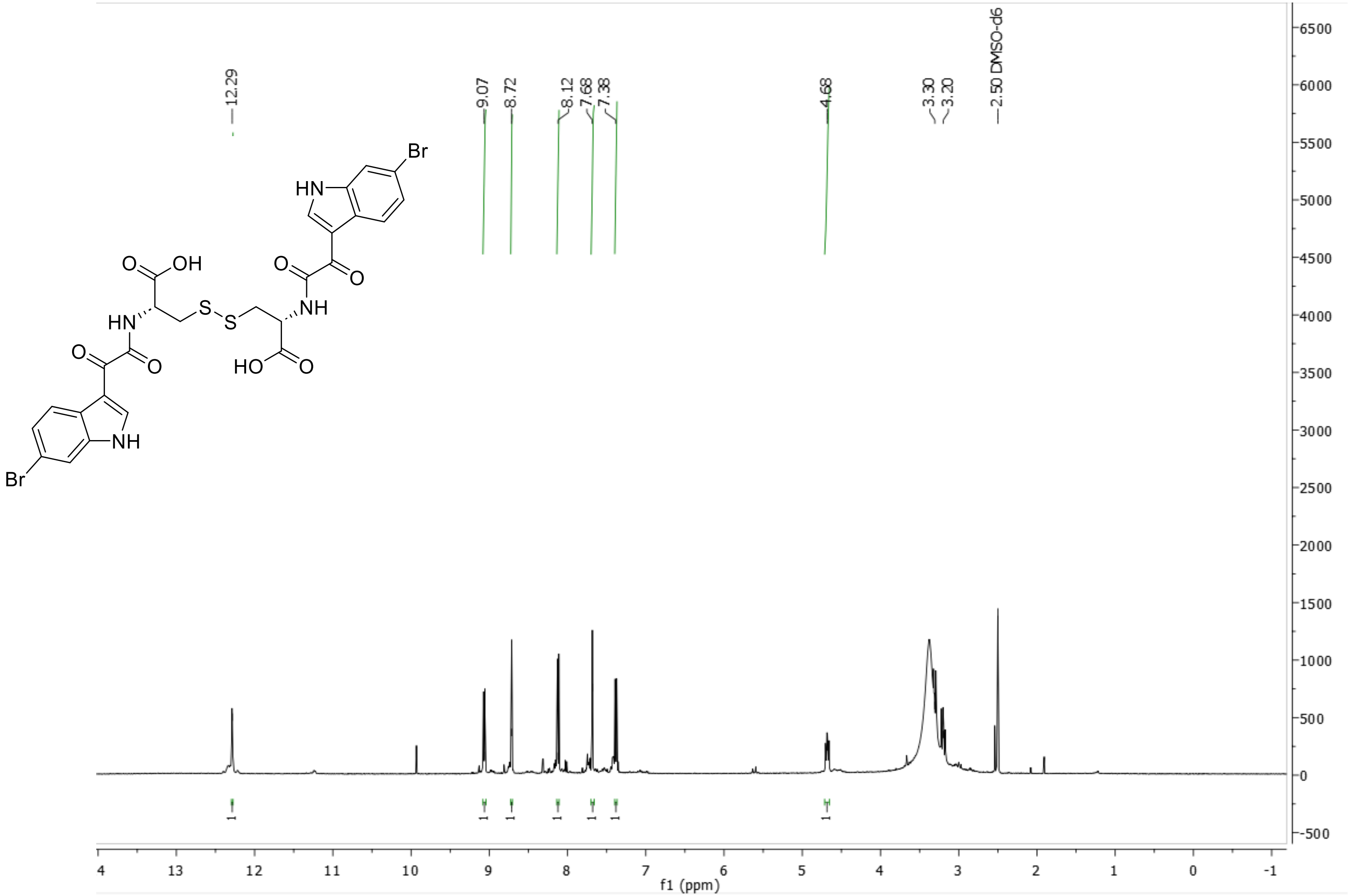

Figure S42. ^{1}H NMR spectrum for 6-bromoindolyl-3-glyoxyl-L-cystine dimer (**34**) recorded in DMSO-d_6

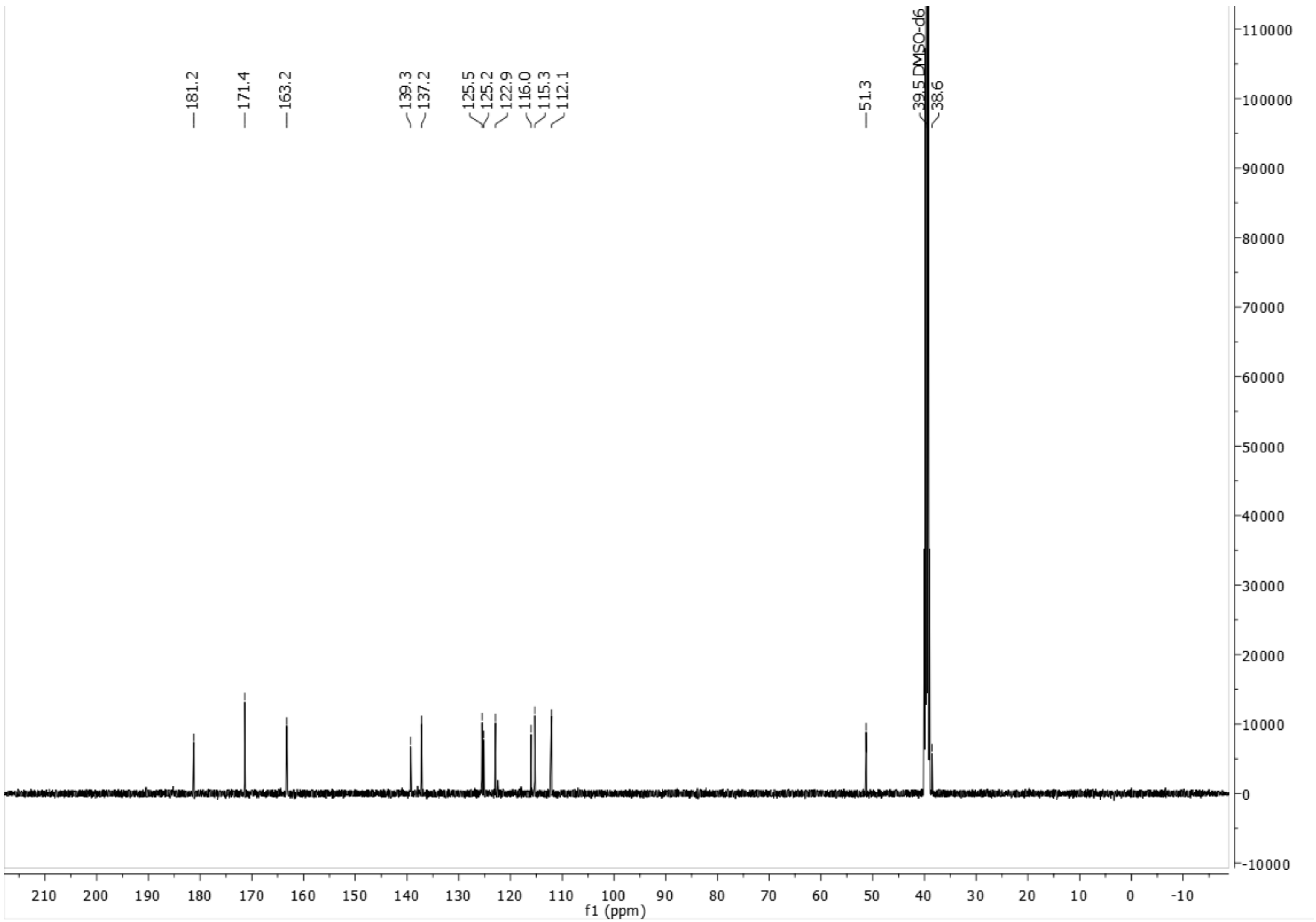

Figure S43. ^{13}C NMR spectrum for 6-bromoindolyl-3-glyoxyl-L-cystine dimer (**34**) recorded in DMSO-d_6

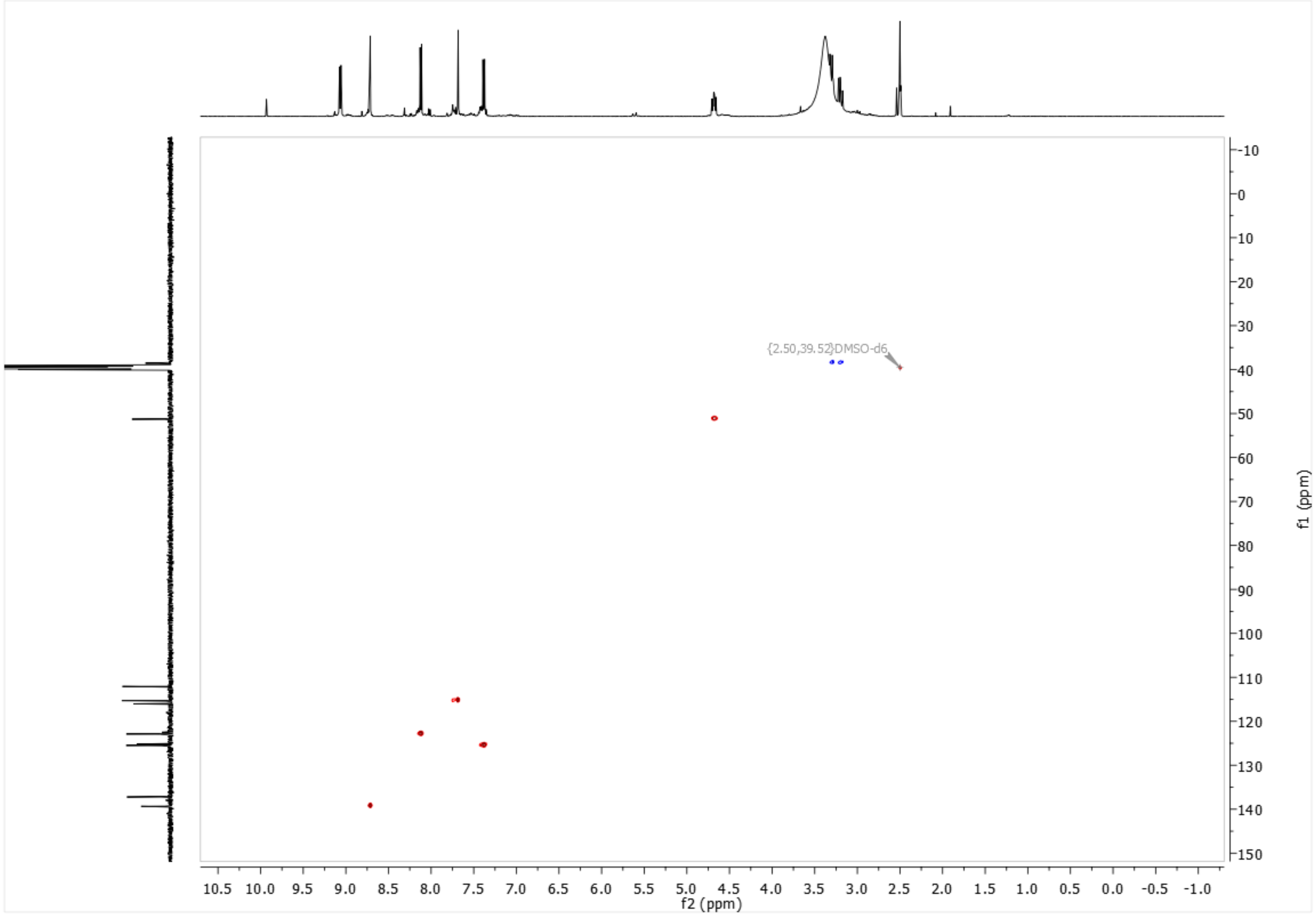

Figure S44. HSQC NMR spectrum for 6-bromoindolyl-3-glyoxyl-L-cystine dimer (**34**) recorded in DMSO-d_6

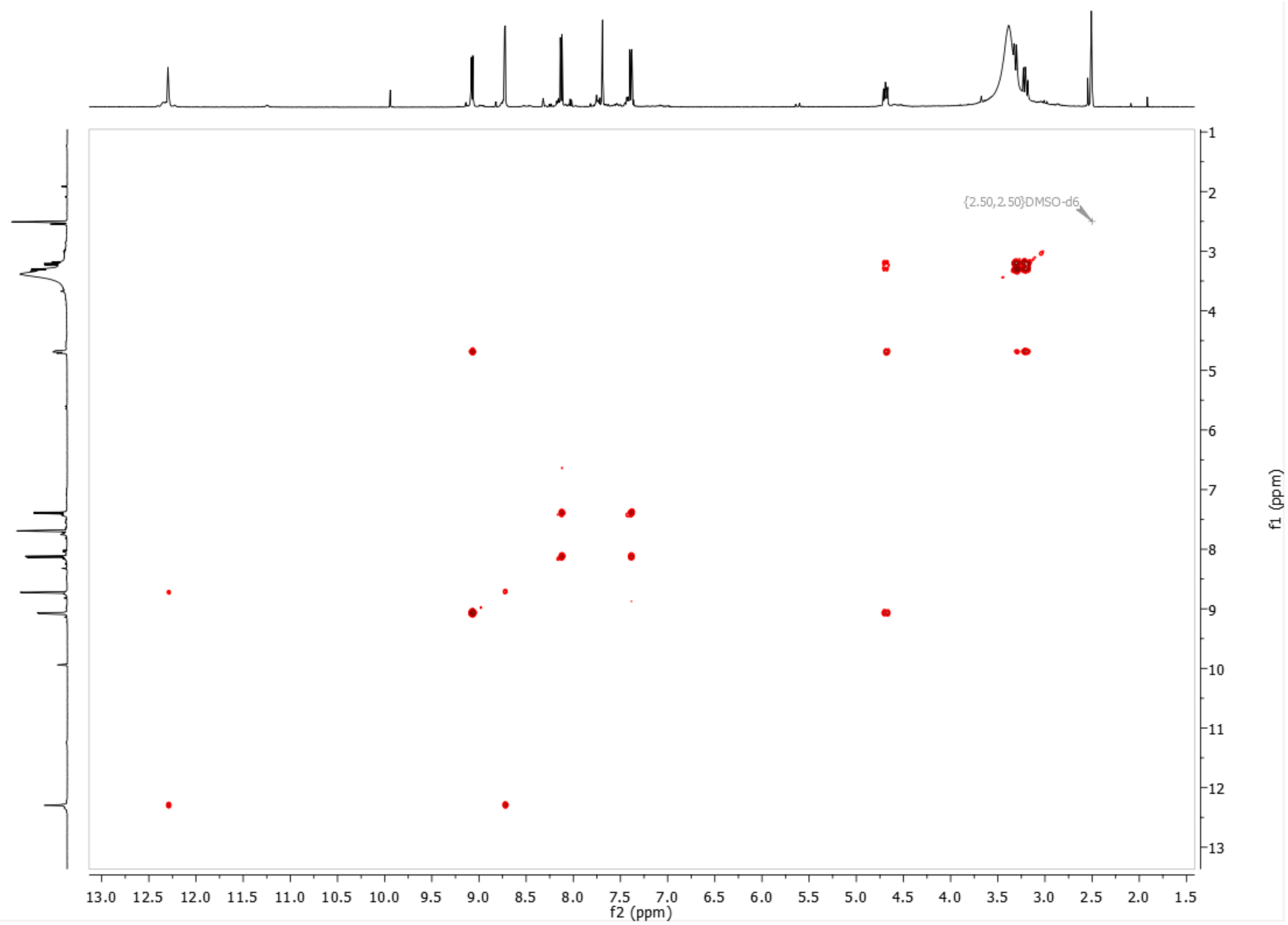

Figure S45. COSY NMR spectrum for 6-bromoindolyl-3-glyoxyl-L-cystine dimer (**34**) recorded in DMSO-d_6

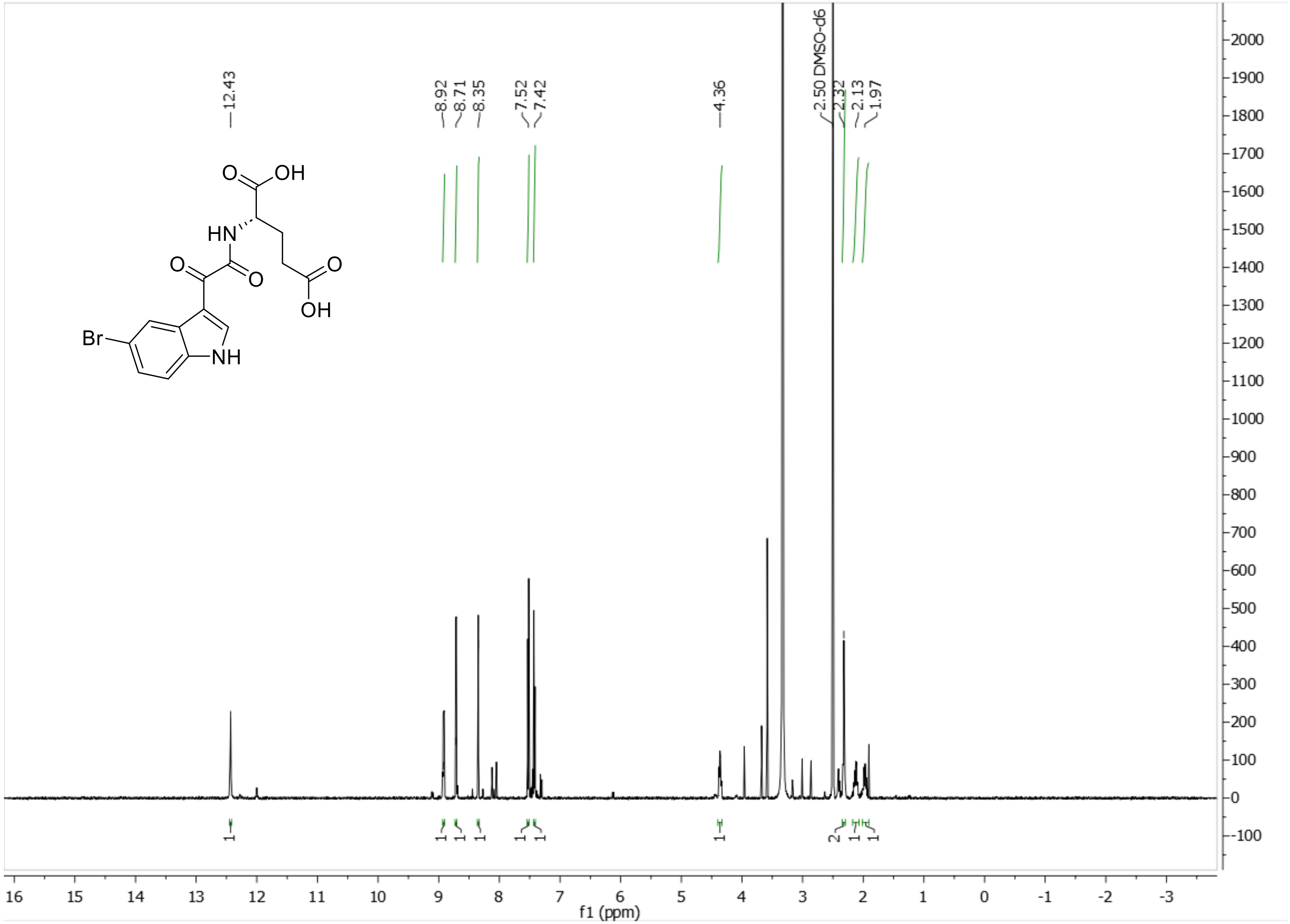

Figure S46. ^{1}H NMR spectrum for 5-bromoindolyl-3-glyoxyl-L-glutamic acid (**35**) recorded in DMSO-d_6

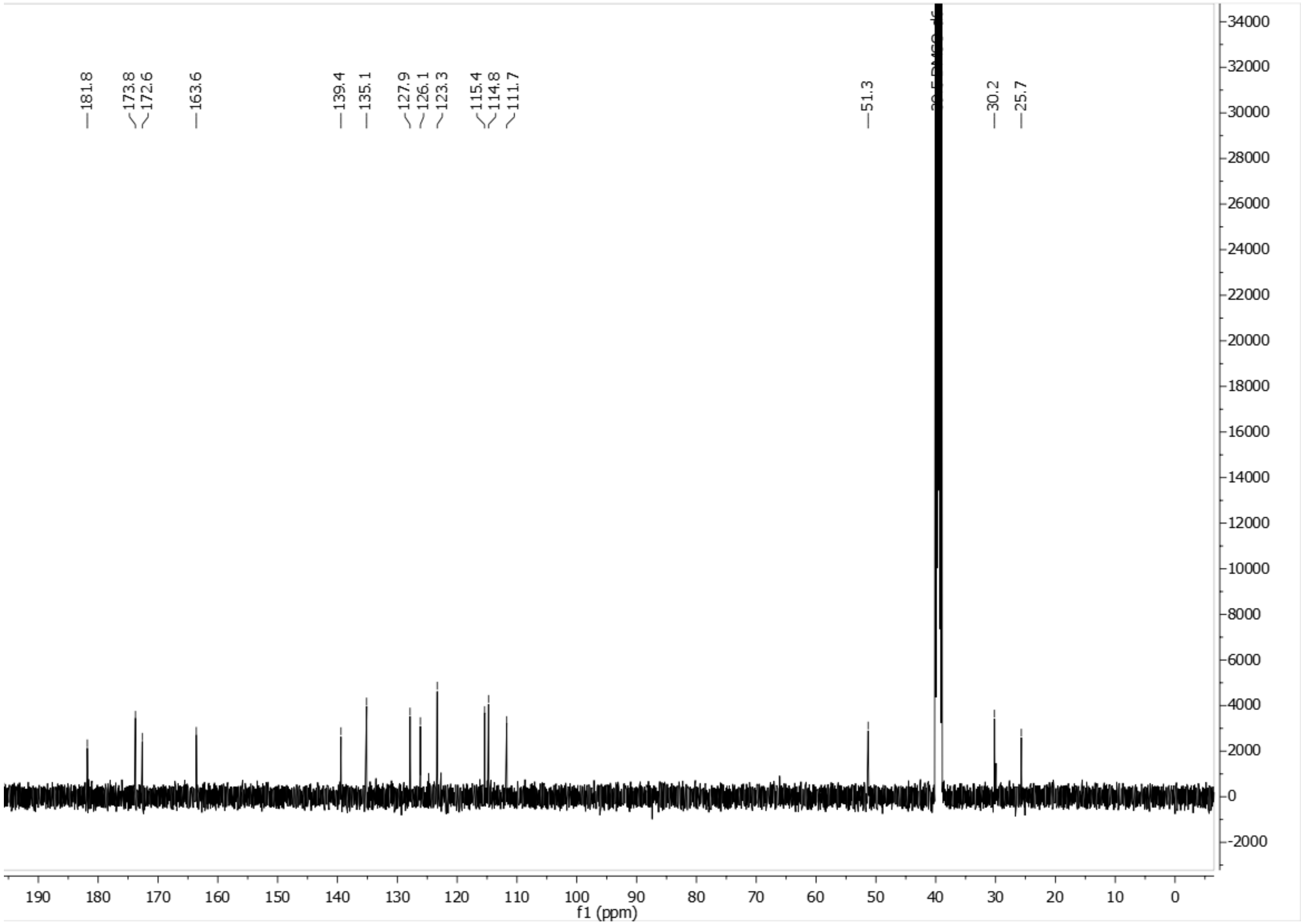

Figure S47. ^{13}C NMR spectrum for 5-bromoindolyl-3-glyoxyl-L-glutamic acid (**35**) recorded in DMSO-d_6

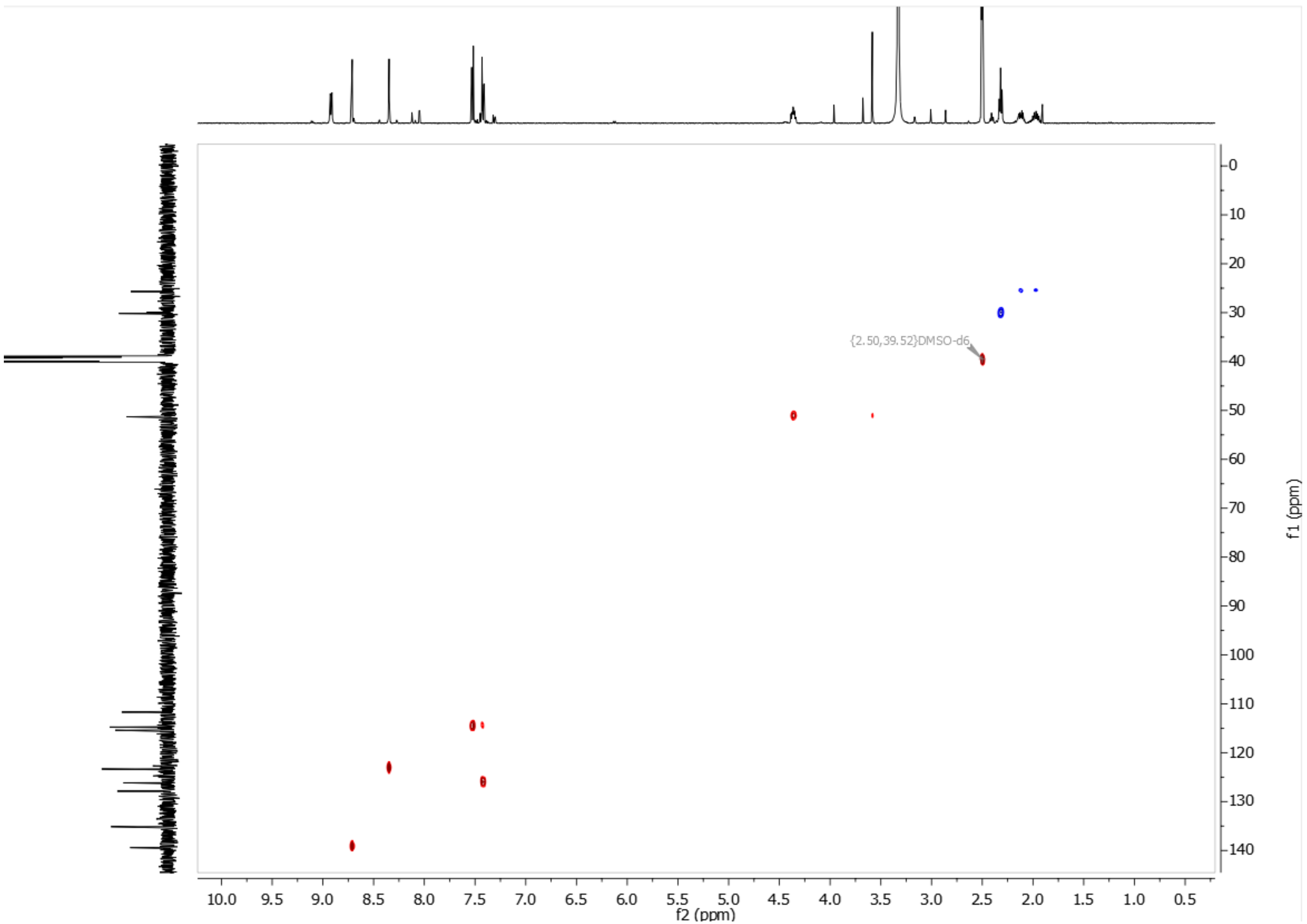

Figure S48. HSQC NMR spectrum for 5-bromoindolyl-3-glyoxyl-L-glutamic acid (**35**) recorded in DMSO-d_6

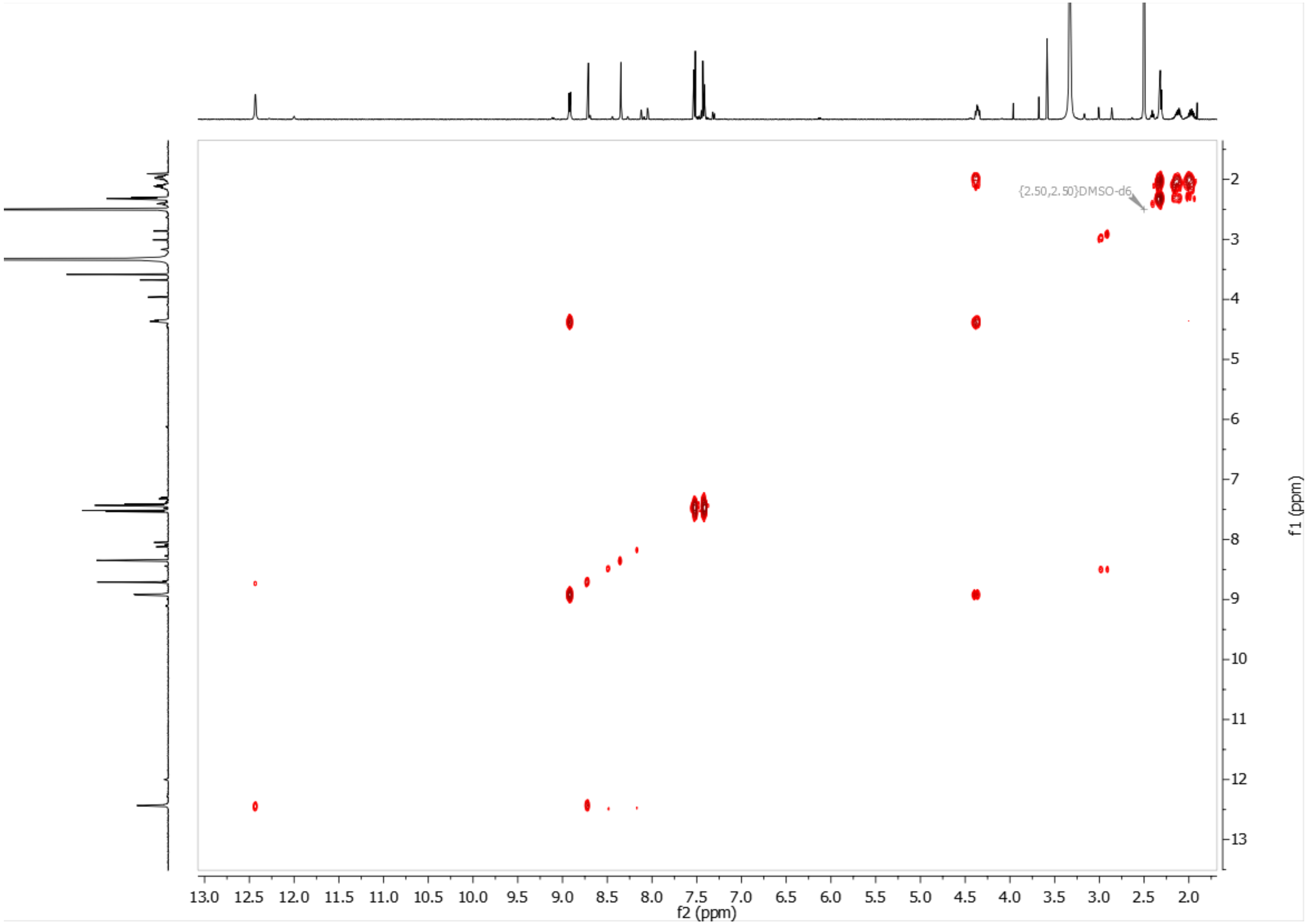

Figure S49. COSY NMR spectrum for 5-bromoindolyl-3-glyoxyl-L-glutamic acid (**35**) recorded in DMSO-d_6

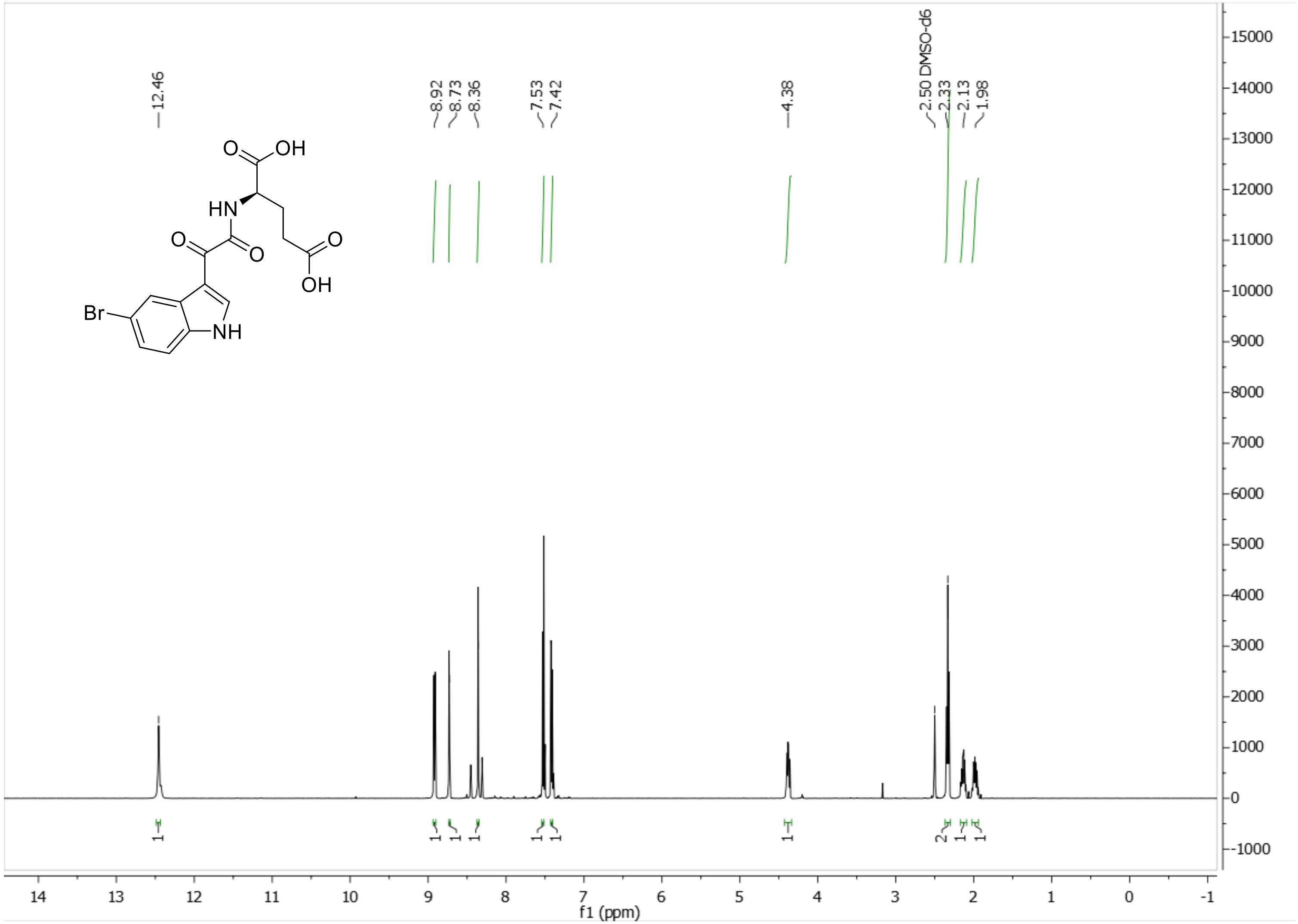

Figure S50. ^{1}H NMR spectrum for 5-bromoindolyl-3-glyoxyl-D-glutamic acid (**36**) recorded in DMSO-d_6

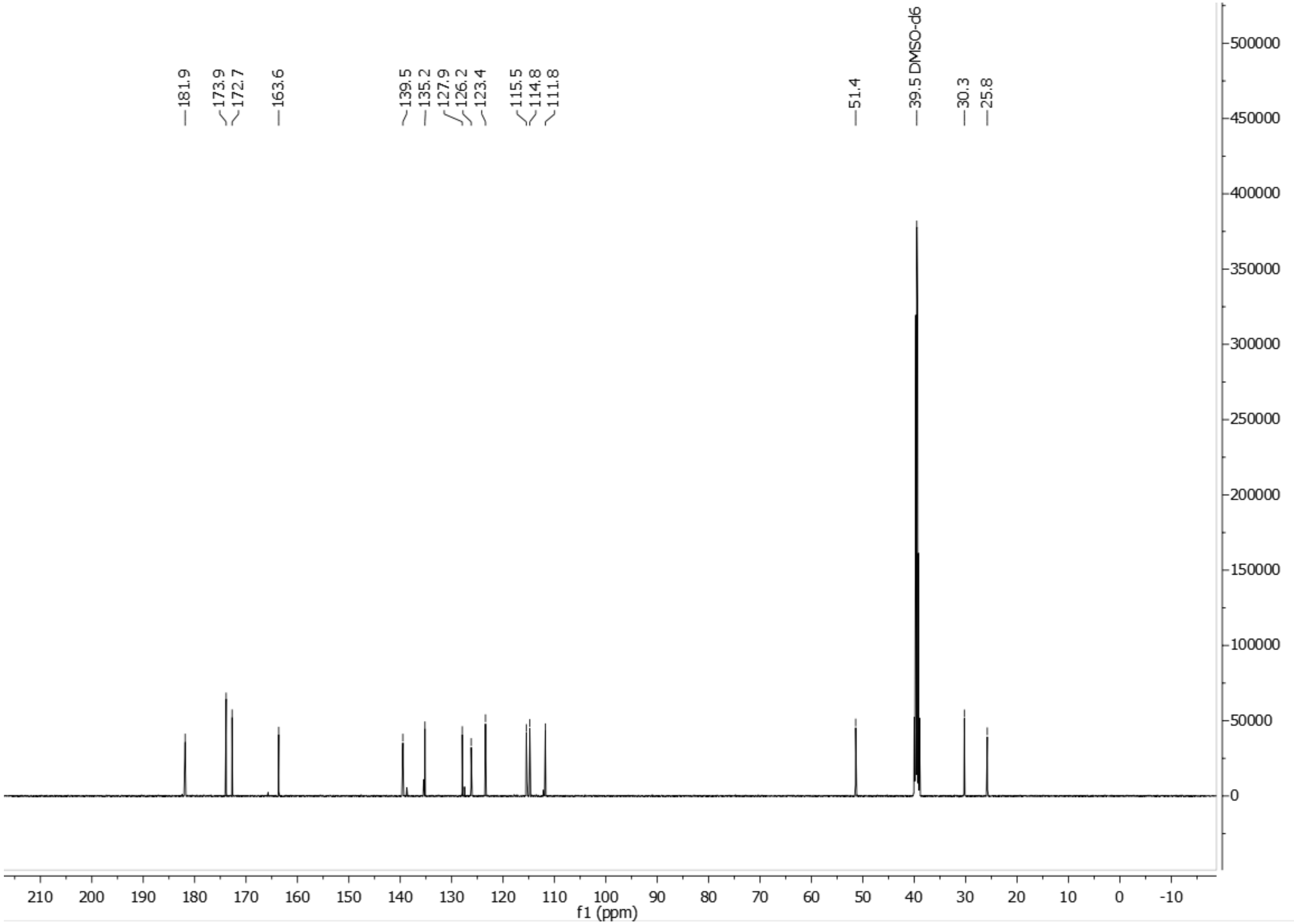

Figure S51. ^{13}C NMR spectrum for 5-bromoindolyl-3-glyoxyl-D-glutamic acid (**36**) recorded in DMSO-d_6

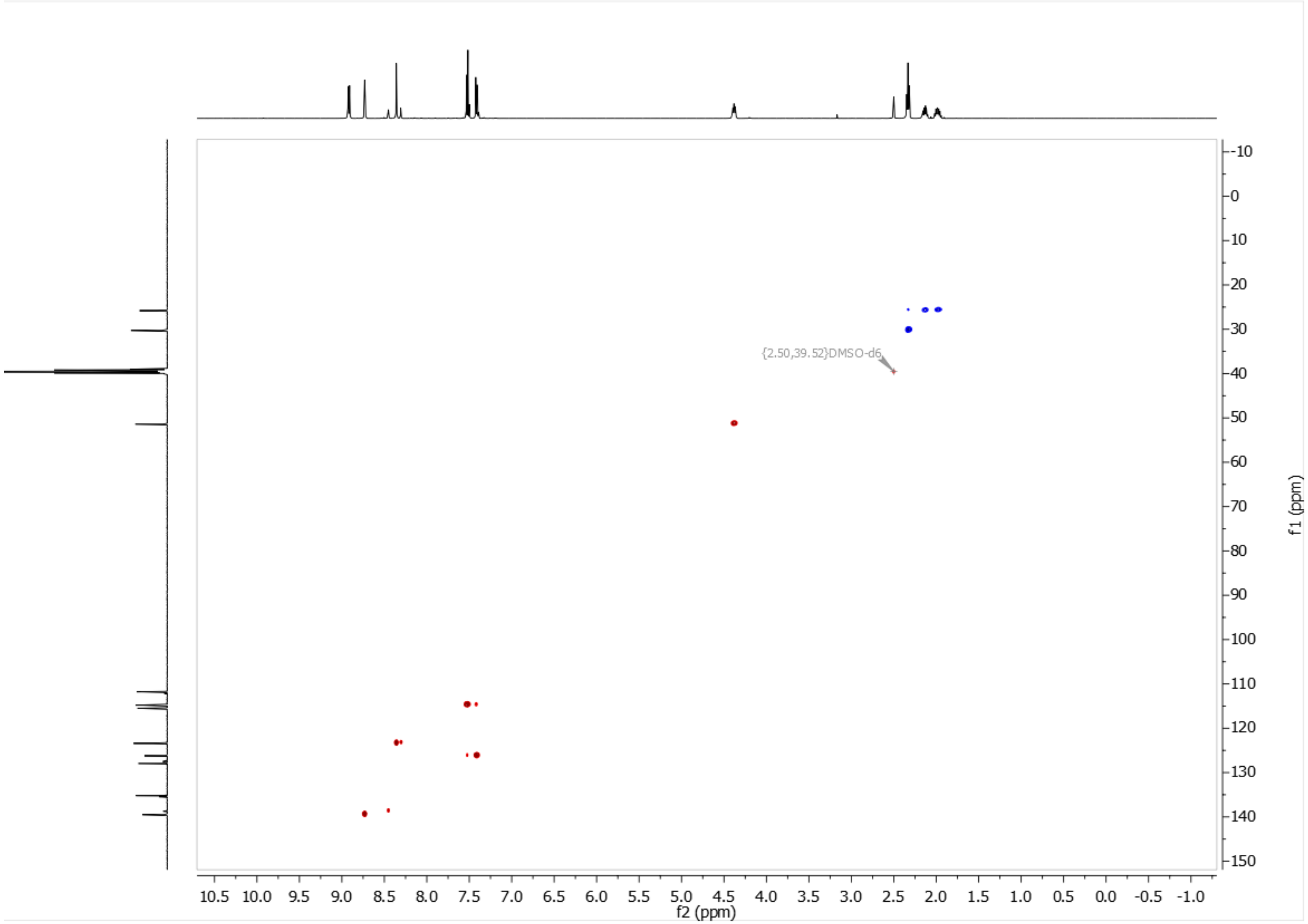

Figure S52. HSQC NMR spectrum for 5-bromoindolyl-3-glyoxyl-D-glutamic acid (**36**) recorded in DMSO-d_6

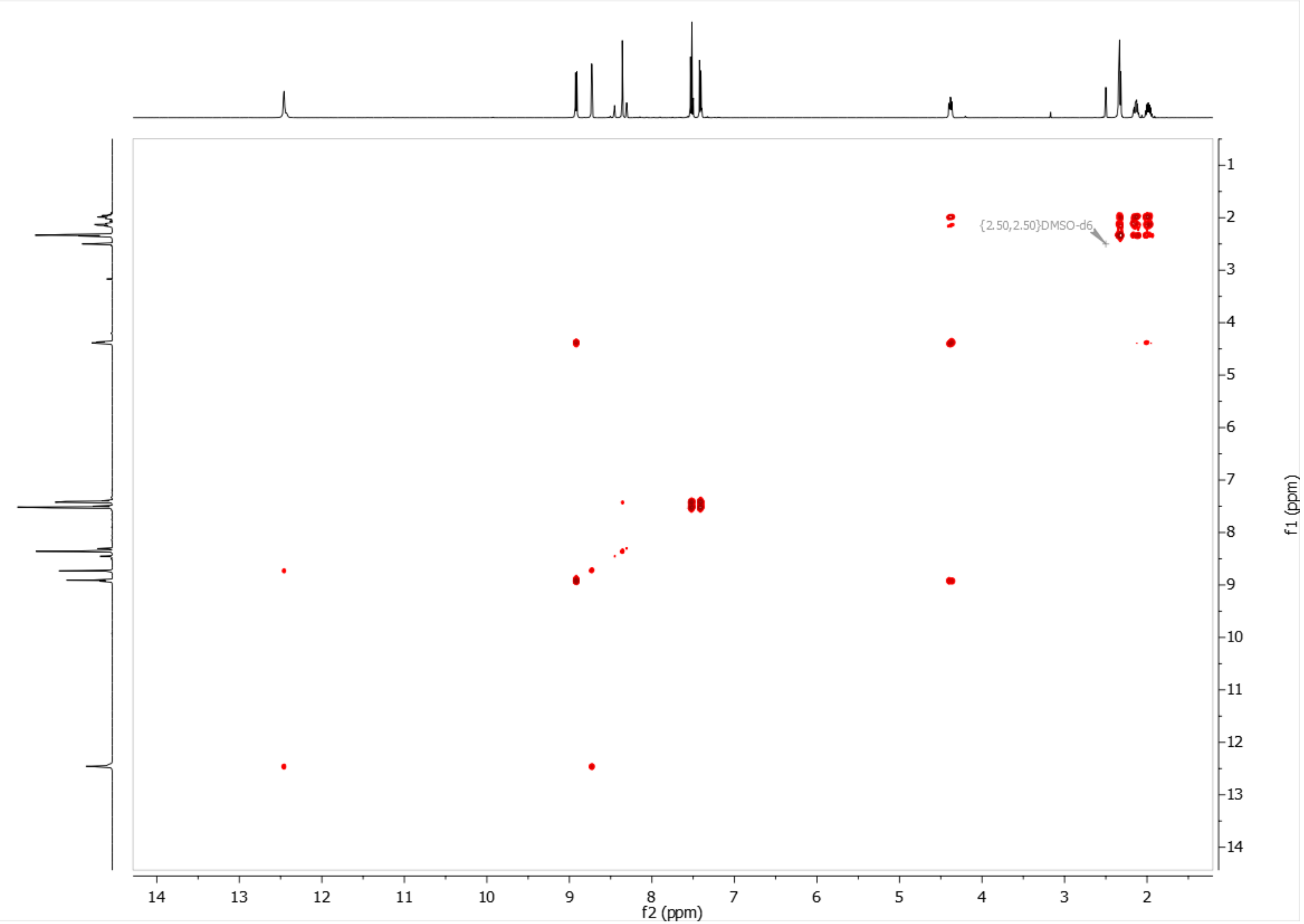

Figure S53. COSY NMR spectrum for 5-bromoindolyl-3-glyoxyl-D-glutamic acid (**36**) recorded in DMSO-d_6

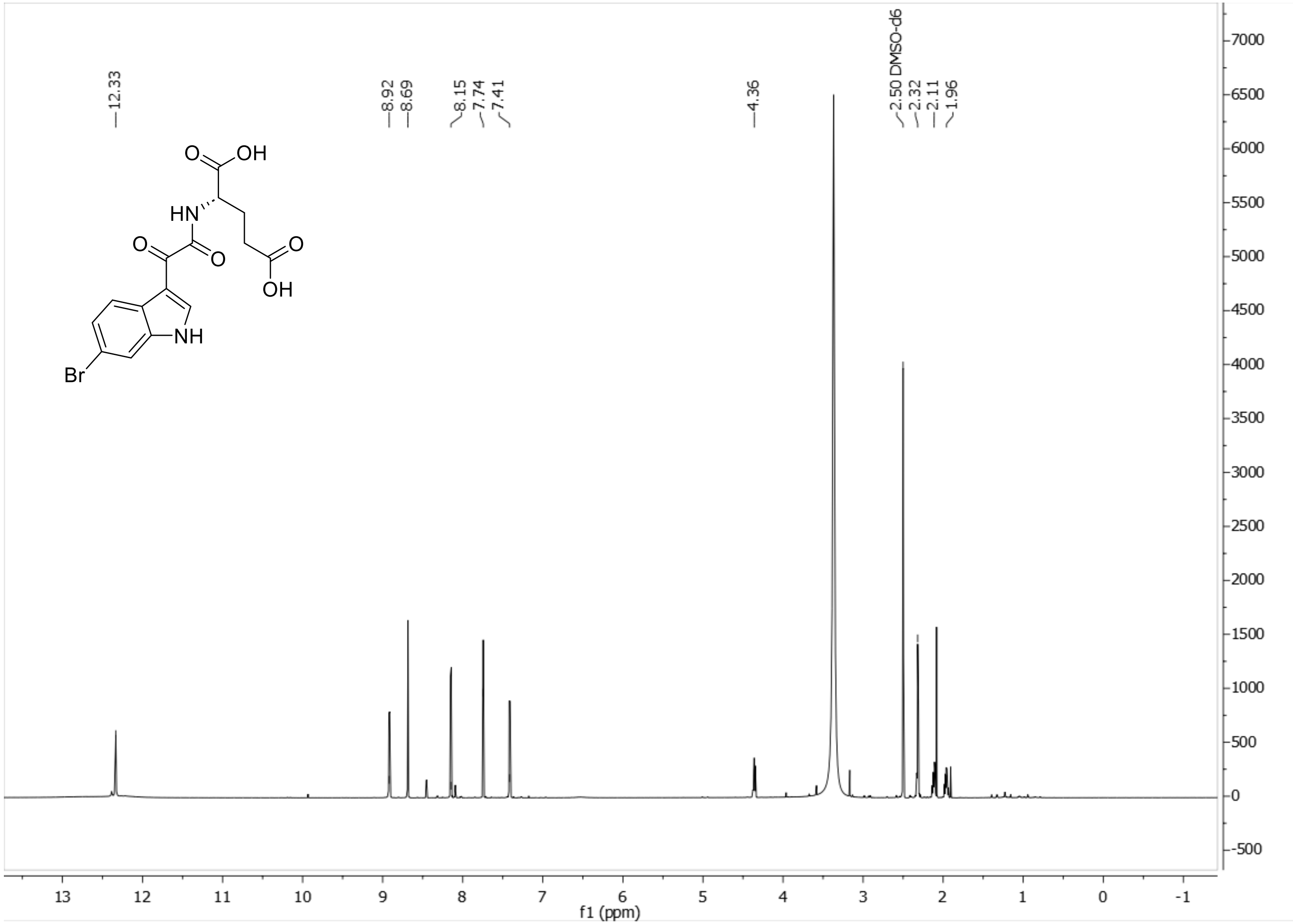

Figure S54. ^{1}H NMR spectrum for 6-bromoindolyl-3-glyoxyl-L-glutamic acid (**37**) recorded in DMSO-d_6

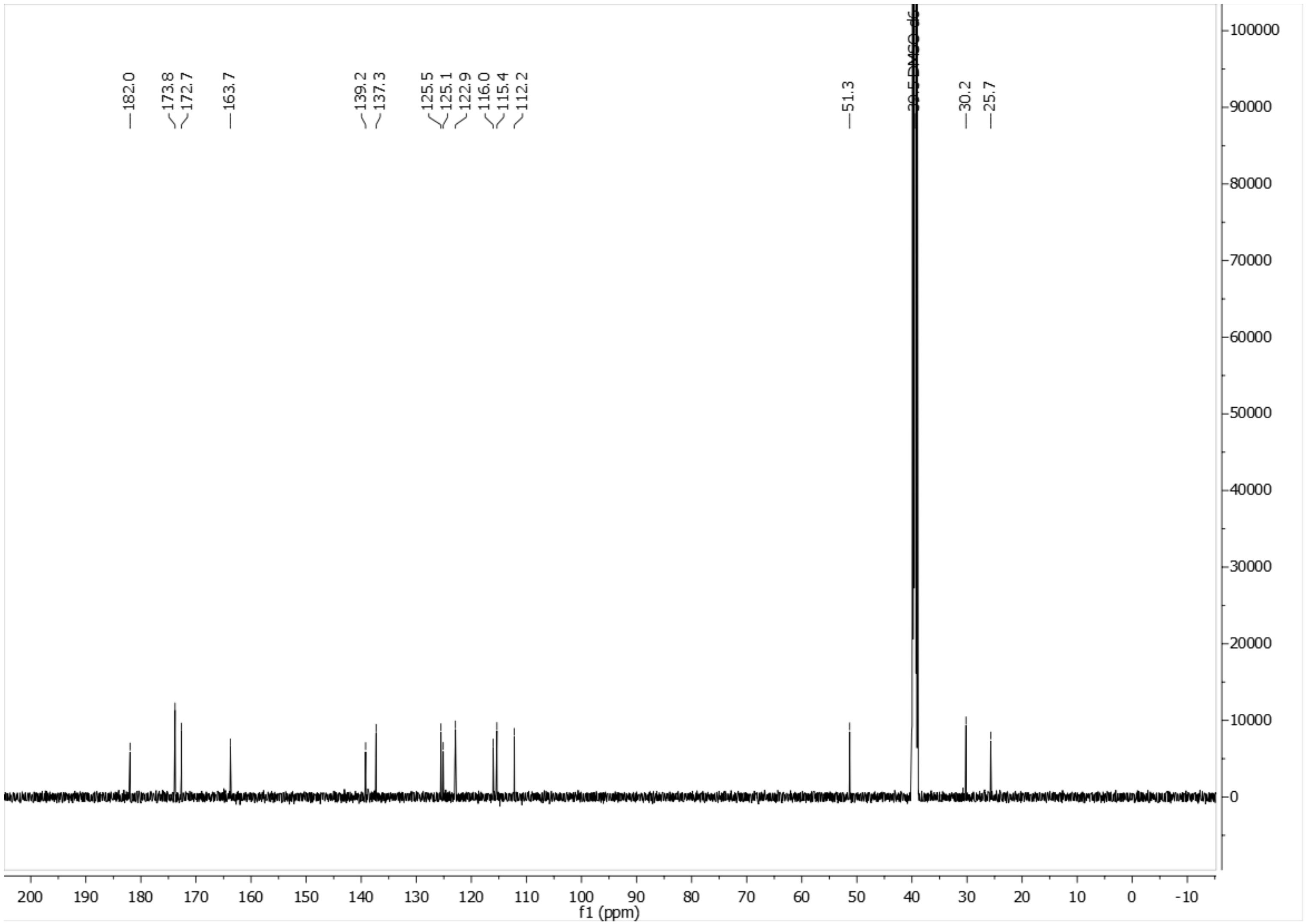

Figure S55. ^{13}C NMR spectrum for 6-bromoindolyl-3-glyoxyl-L-glutamic acid (**37**) recorded in DMSO-d_6

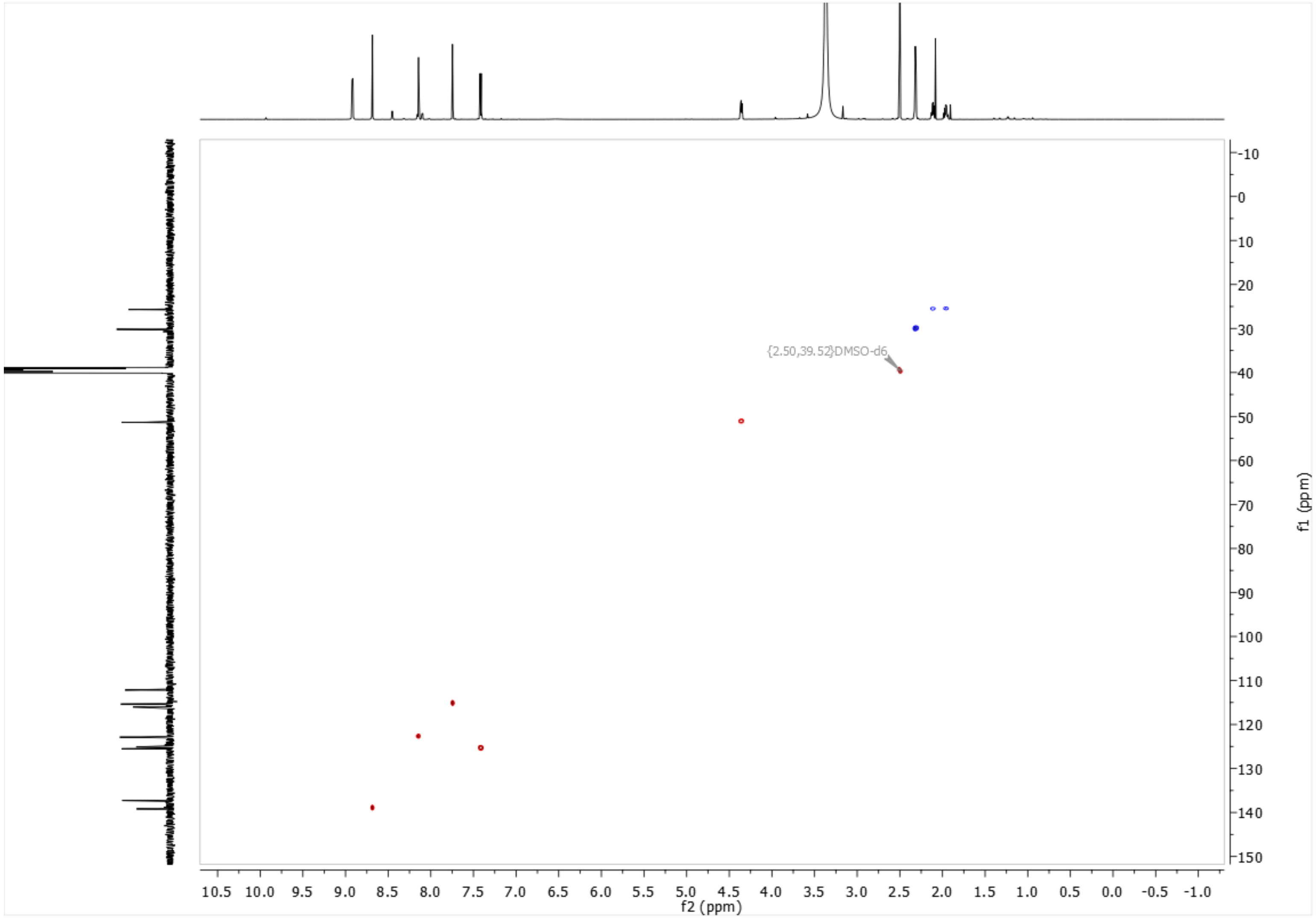

Figure S56. HSQC NMR spectrum for 6-bromoindolyl-3-glyoxyl-L-glutamic acid (**37**) recorded in DMSO-d_6

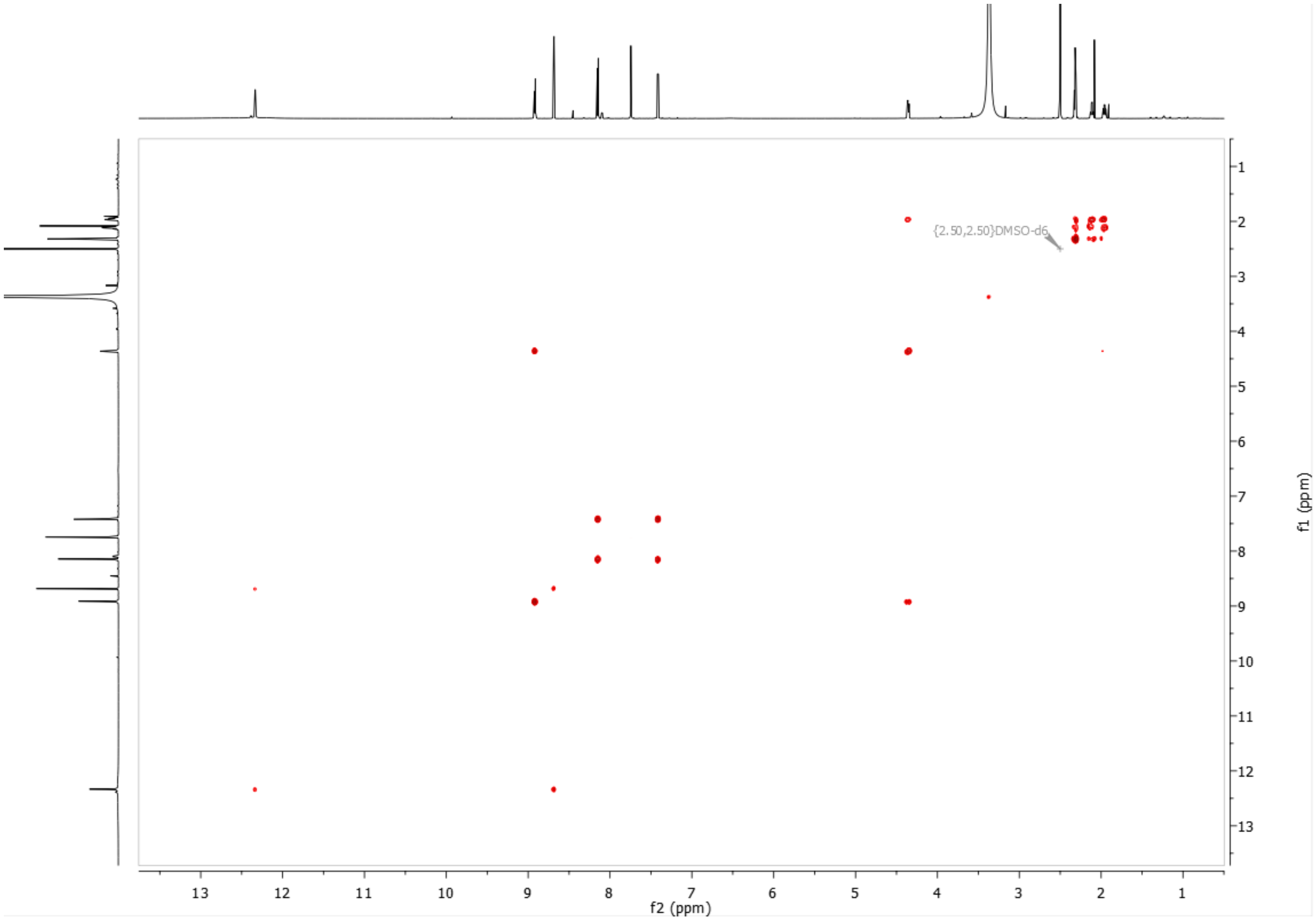

Figure S57. COSY NMR spectrum for 6-bromoindolyl-3-glyoxyl-L-glutamic acid (**37**) recorded in DMSO-d_6

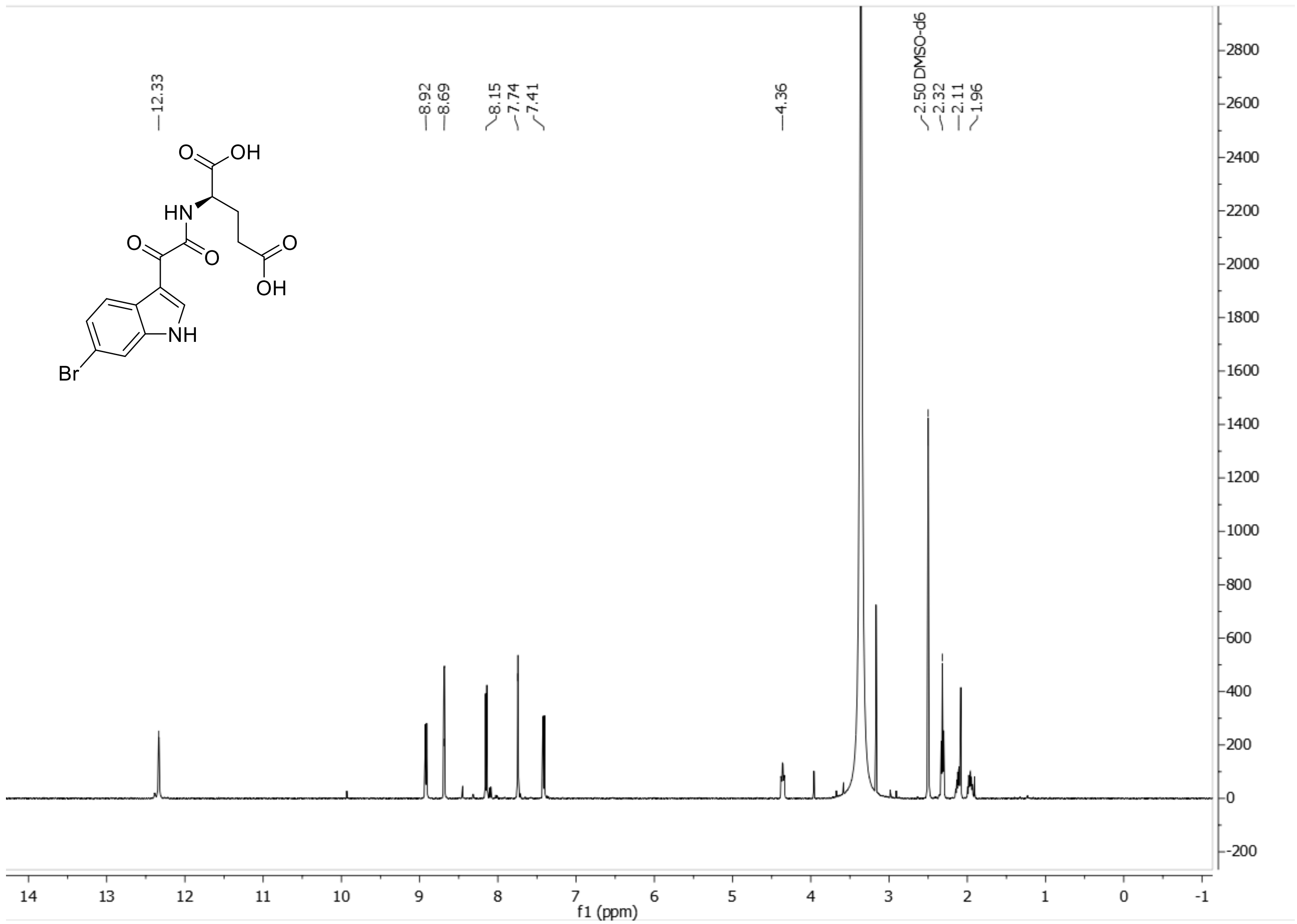

Figure S58. ^{1}H NMR spectrum for 6-bromoindolyl-3-glyoxyl-D-glutamic acid (**38**) recorded in DMSO-d_6

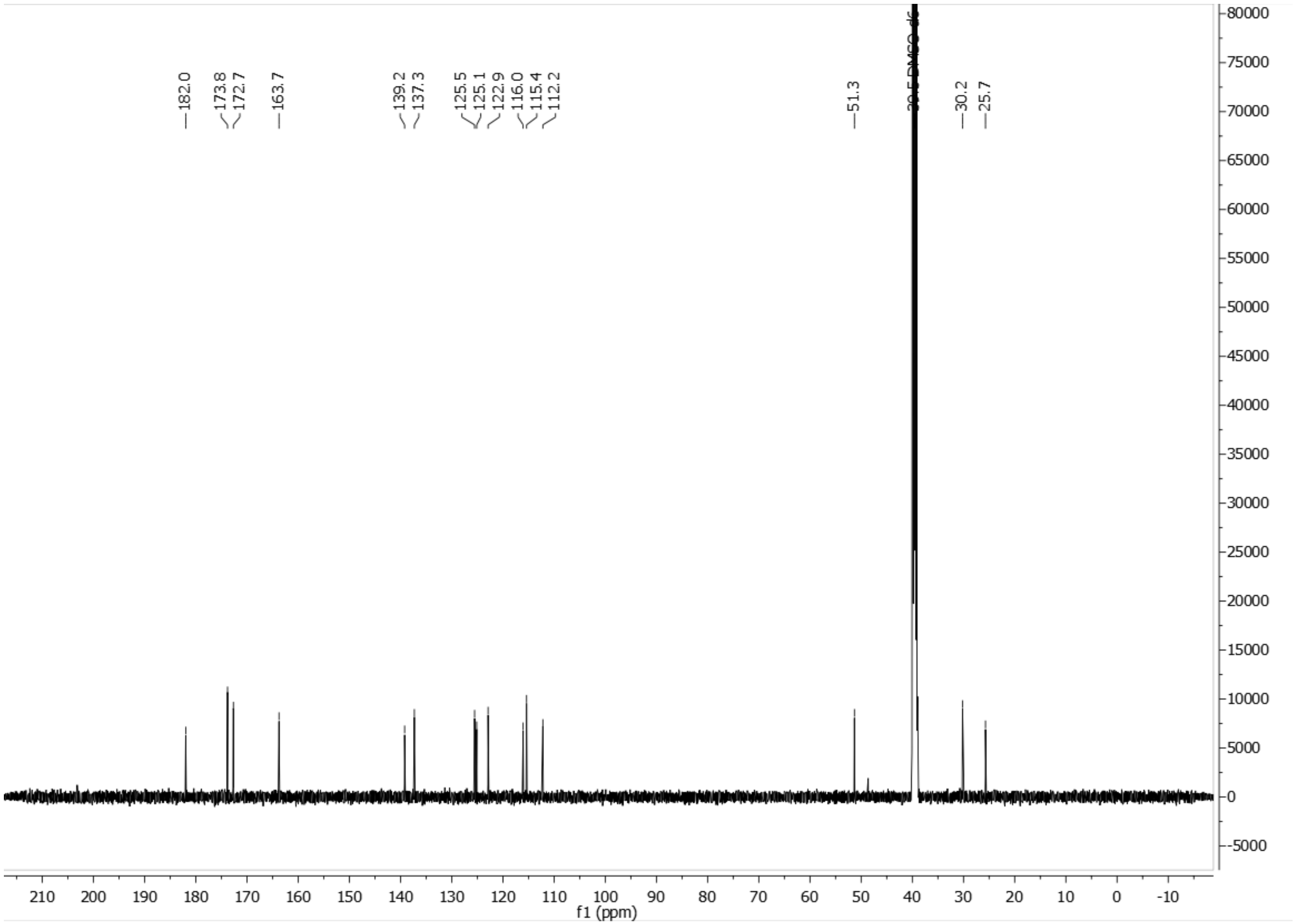

Figure S59. ^{13}C NMR spectrum for 6-bromoindolyl-3-glyoxyl-D-glutamic acid (**38**) recorded in DMSO-d_6

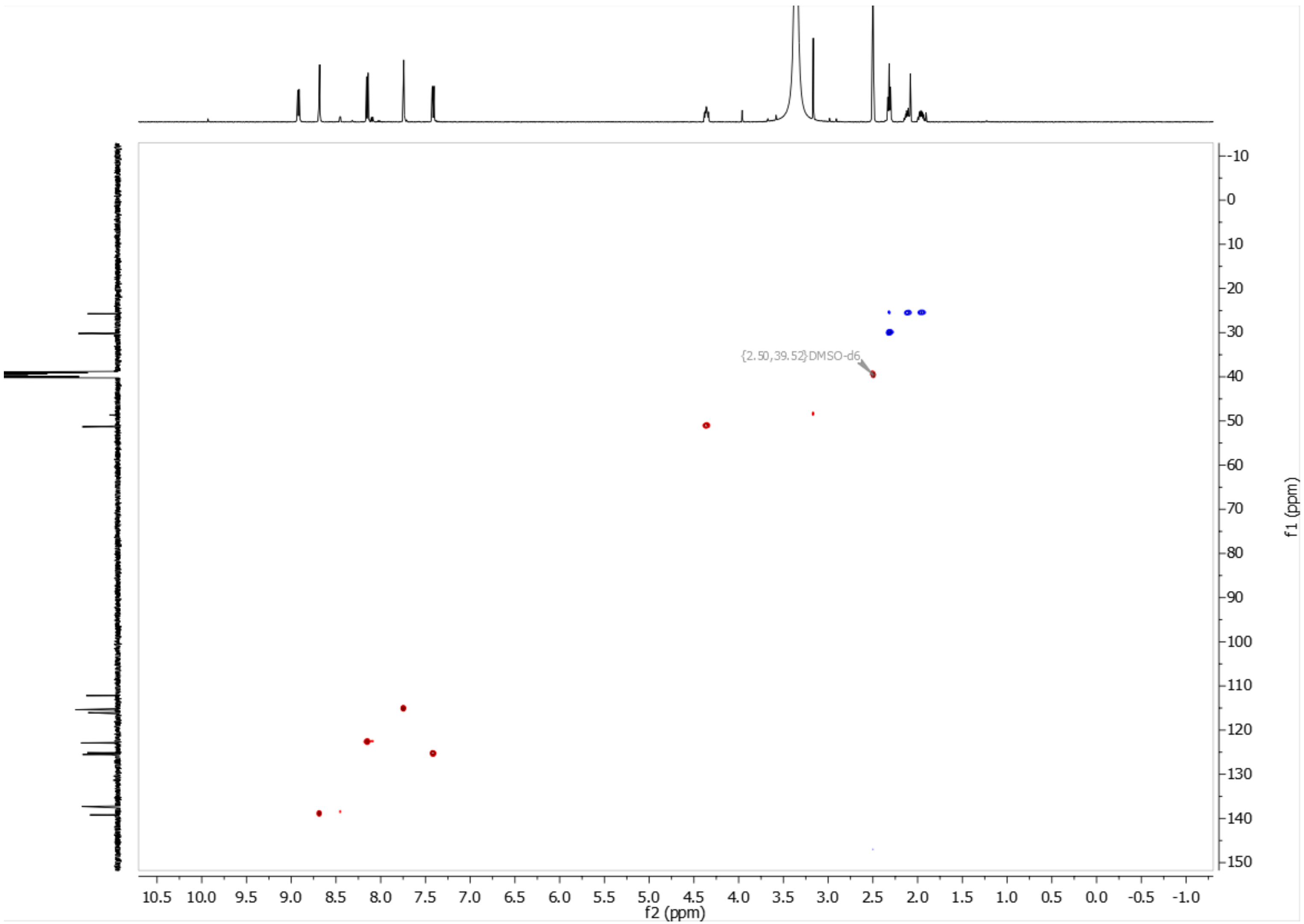

Figure S60. HSQC NMR spectrum for 6-bromoindolyl-3-glyoxyl-D-glutamic acid (**38**) recorded in DMSO-d_6

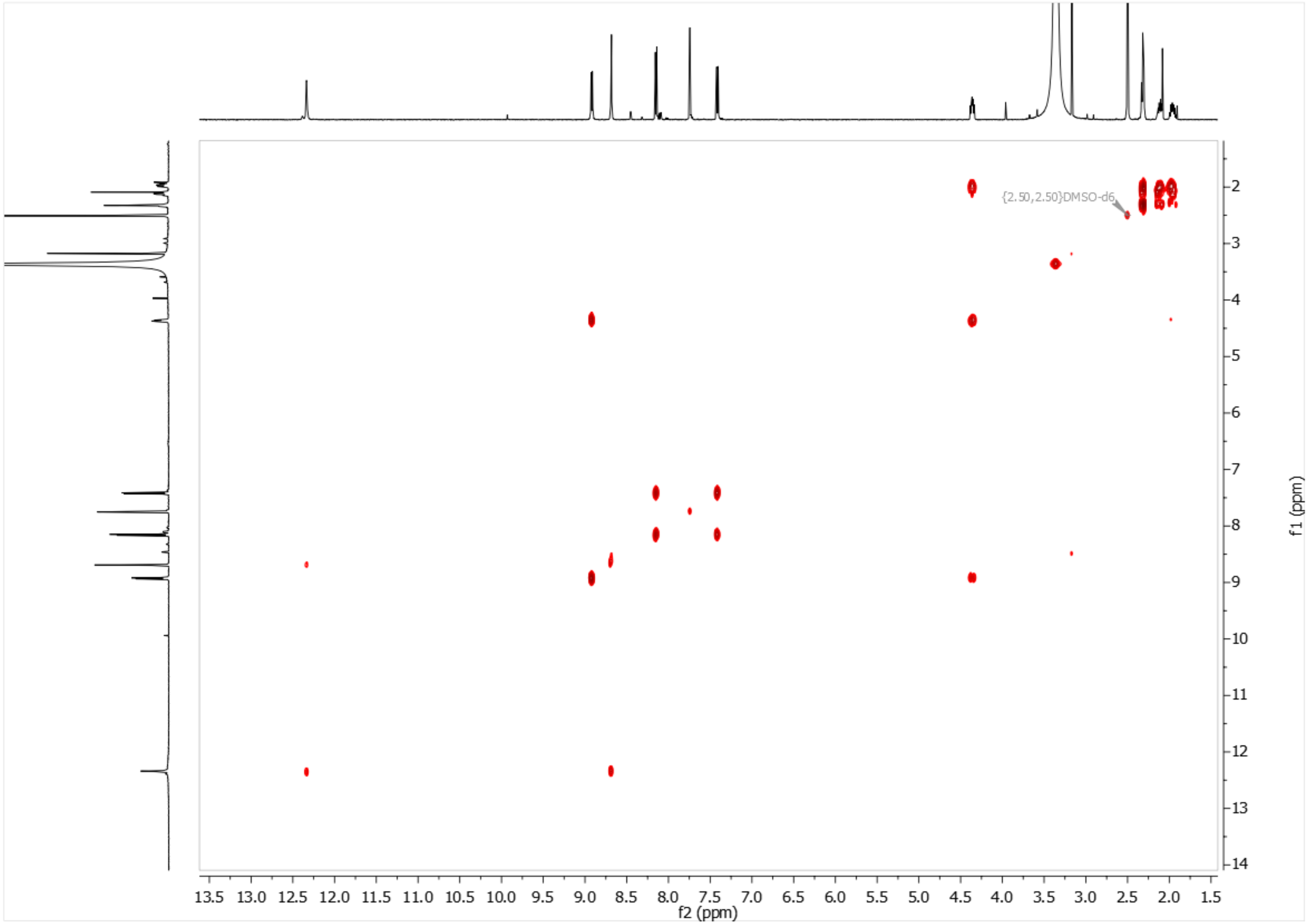

Figure S61. COSY NMR spectrum for 6-bromoindolyl-3-glyoxyl-D-glutamic acid (**38**) recorded in DMSO-d_6

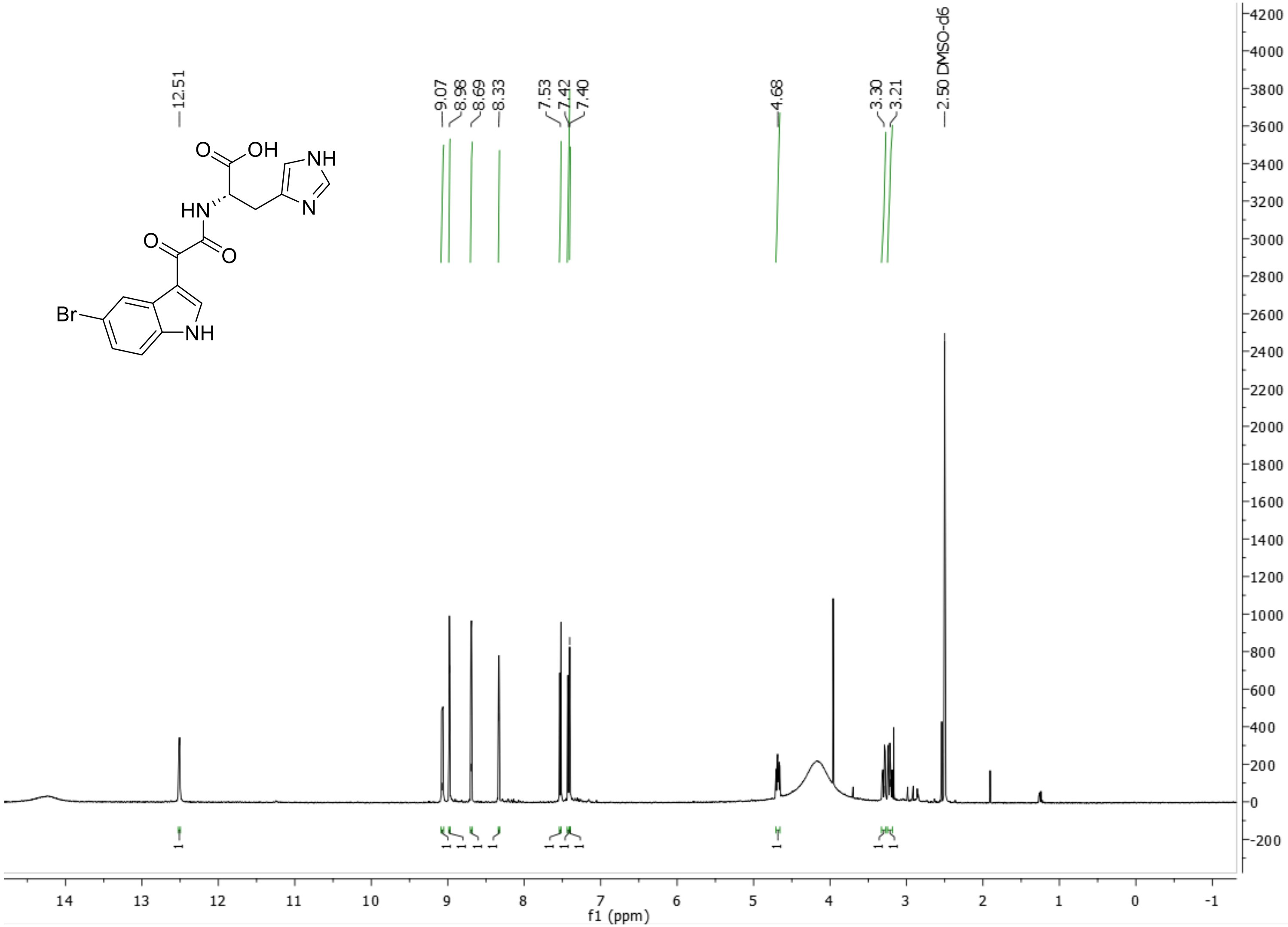

Figure S62. ^{1}H NMR spectrum for 5-bromoindolyl-3-glyoxyl-L-histidine (**39**) recorded in DMSO-d_6

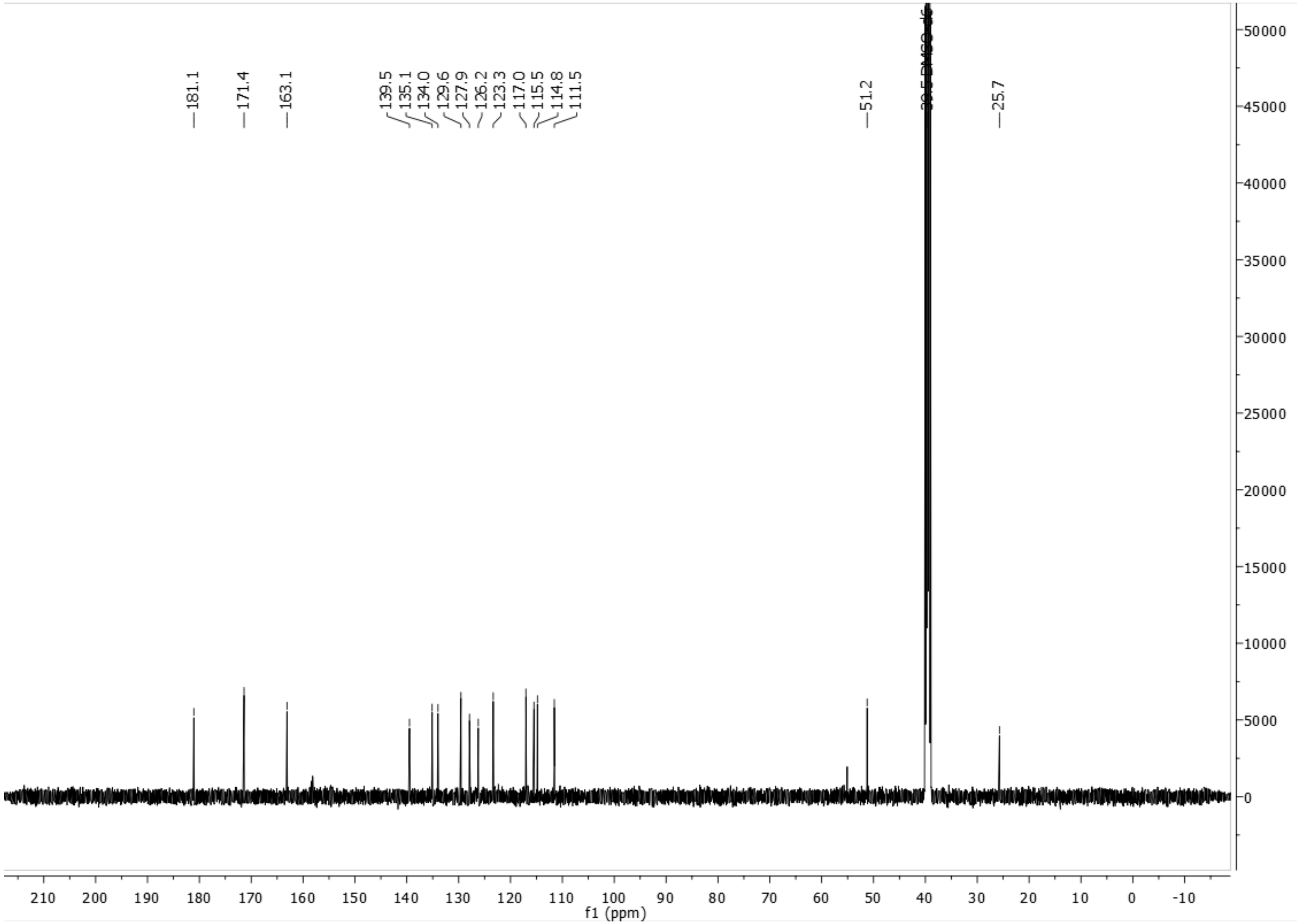

Figure S63. ^{13}C NMR spectrum for 5-bromoindolyl-3-glyoxyl-L-histidine (**39**) recorded in DMSO-d_6

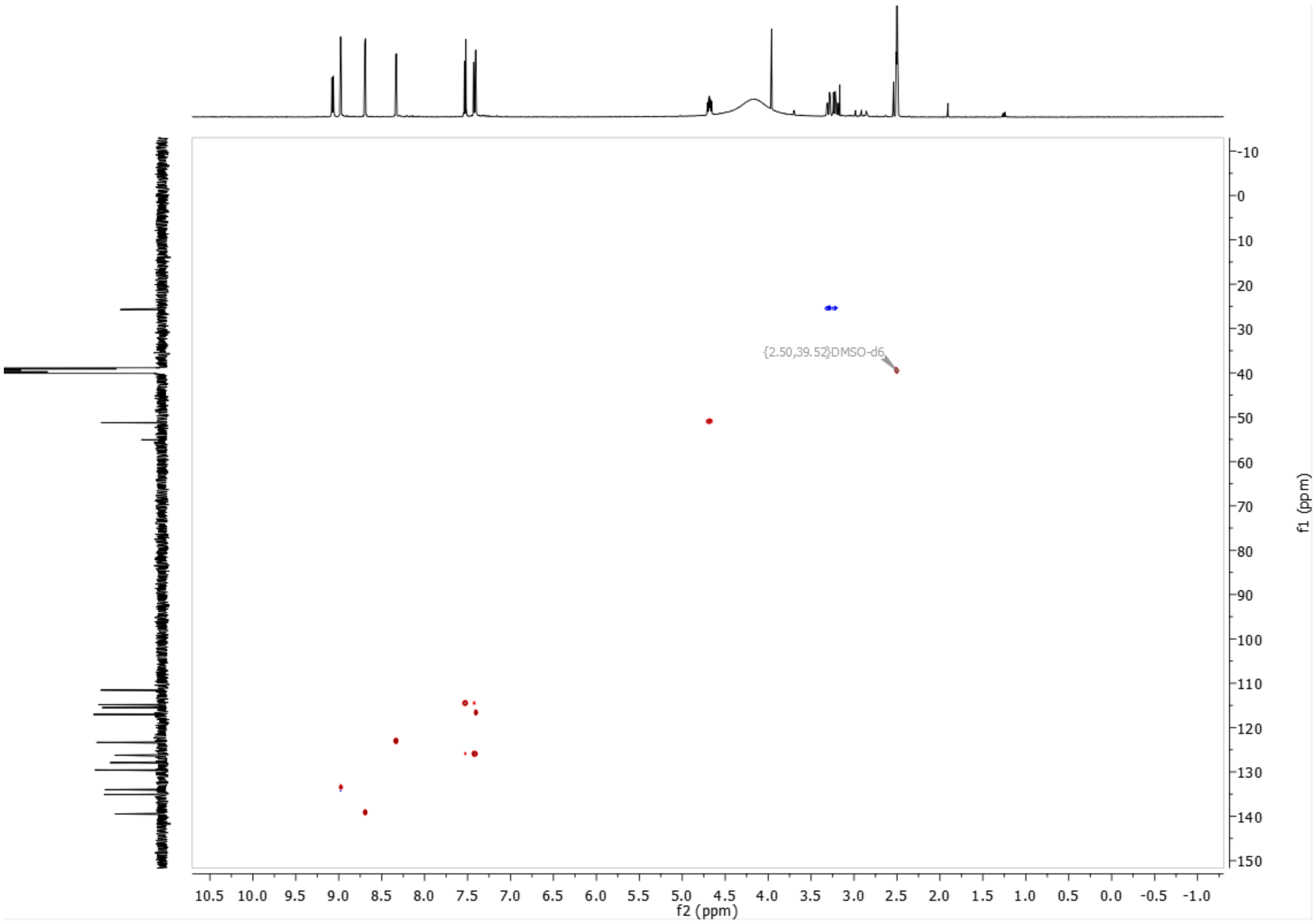

Figure S64. HSQC NMR spectrum for 5-bromoindolyl-3-glyoxyl-L-histidine (**39**) recorded in DMSO-d_6

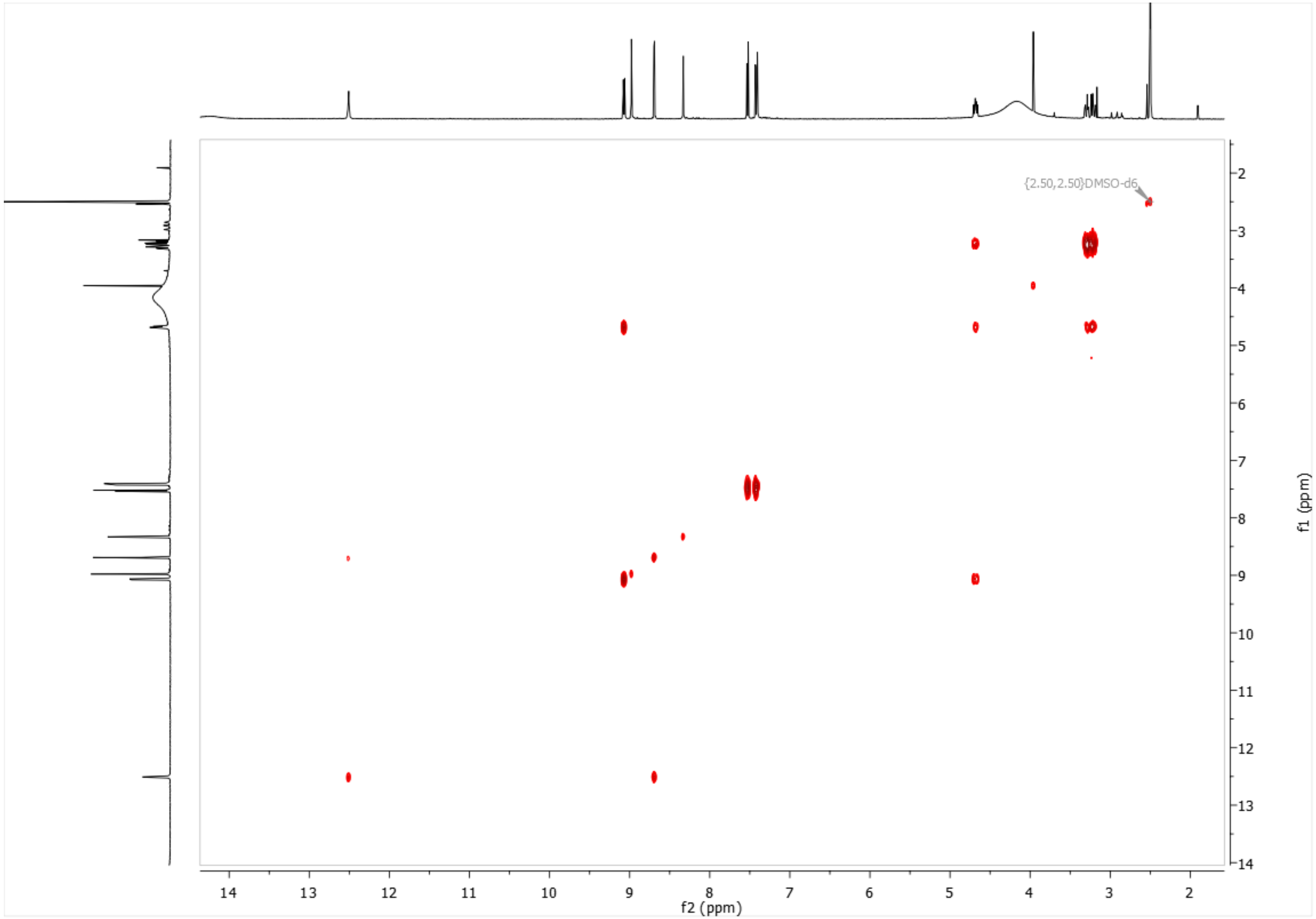

Figure S65. COSY NMR spectrum for 5-bromoindolyl-3-glyoxyl-L-histidine (**39**) recorded in DMSO-d_6

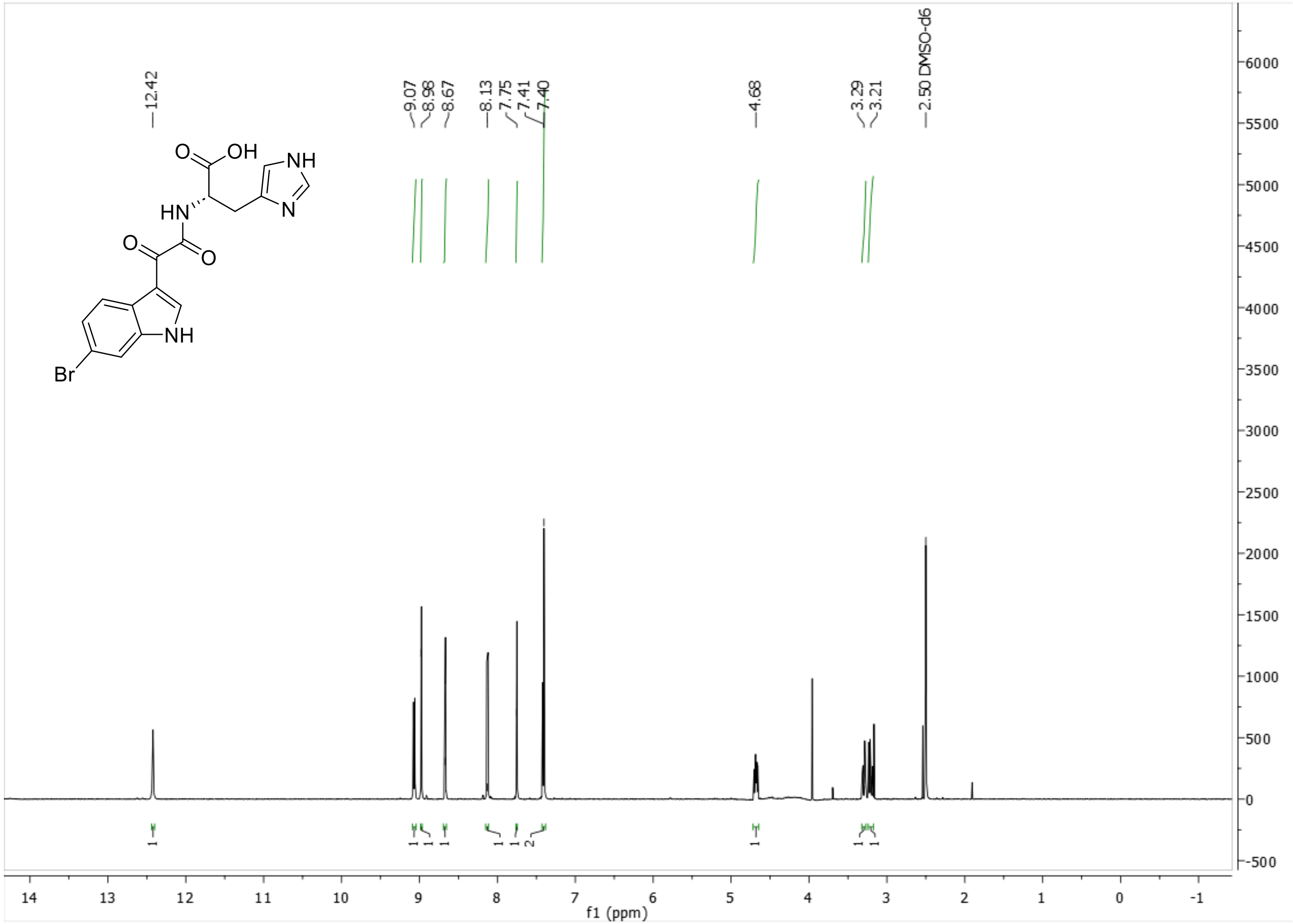

Figure S66. ^{1}H NMR spectrum for 6-bromoindolyl-3-glyoxyl-L-histidine (**40**) recorded in DMSO-d_6

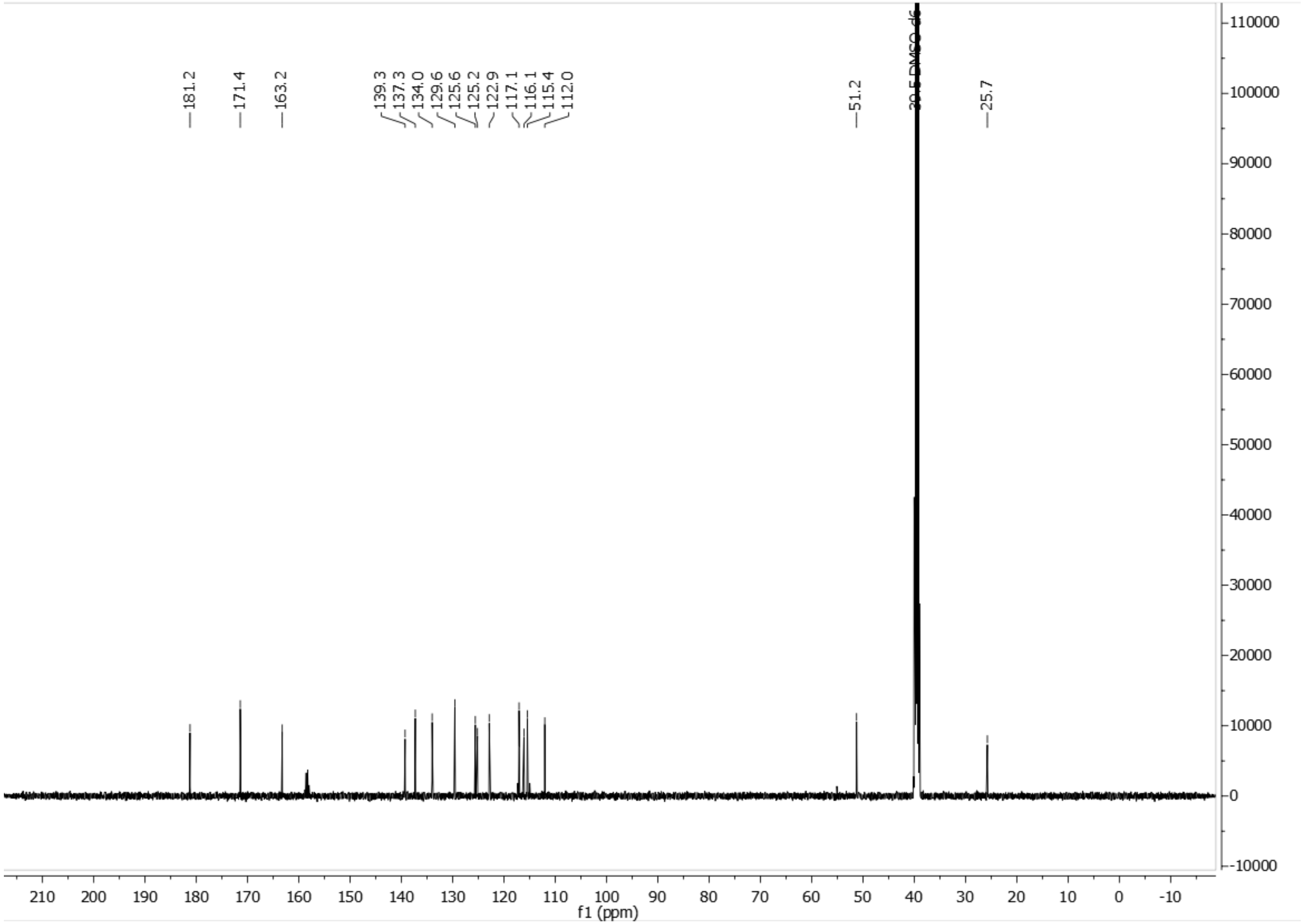

Figure S67. ^{13}C NMR spectrum for 6-bromoindolyl-3-glyoxyl-L-histidine (**40**) recorded in DMSO-d_6

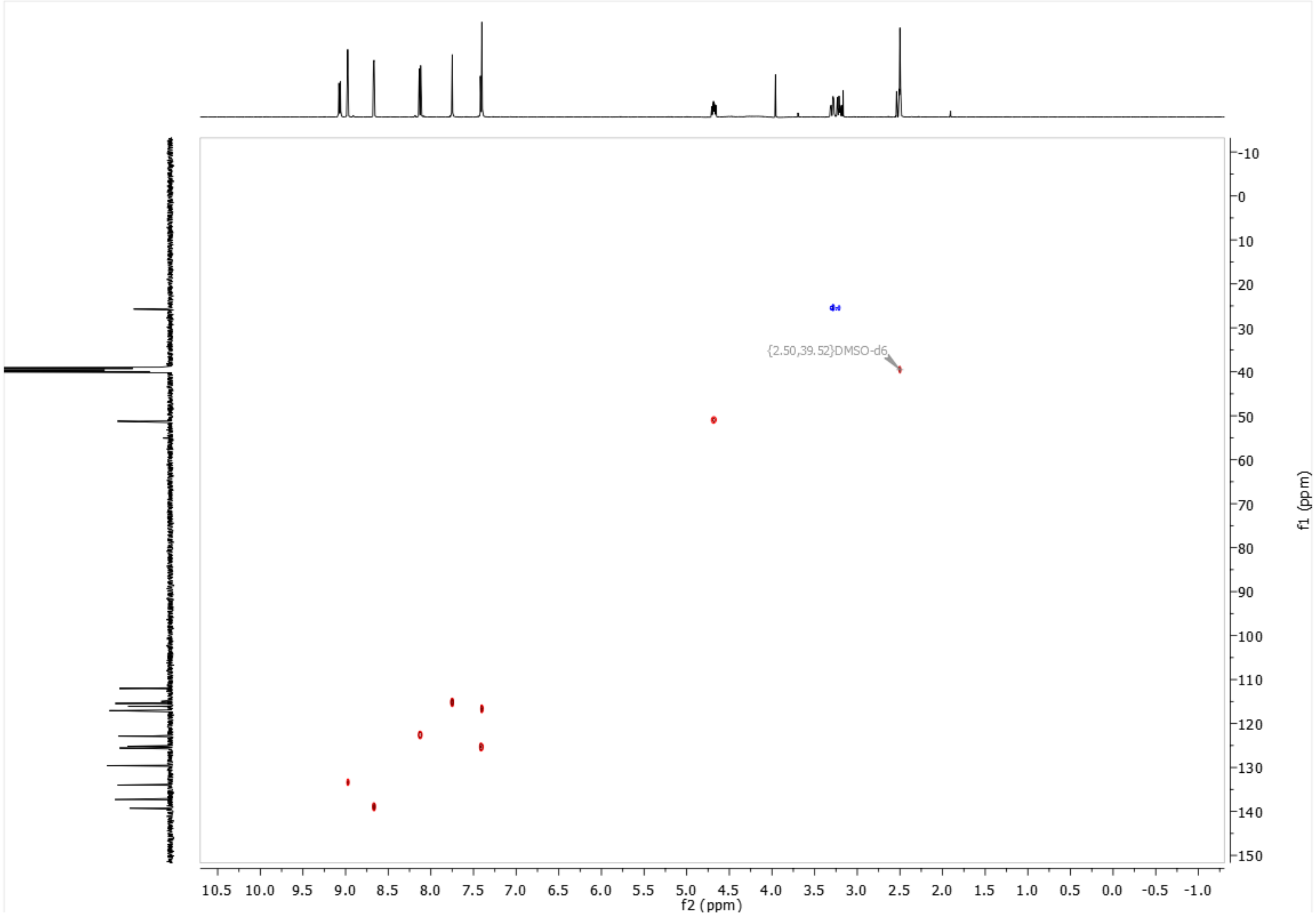

Figure S68. HSQC NMR spectrum for 6-bromoindolyl-3-glyoxyl-L-histidine (**40**) recorded in DMSO-d_6

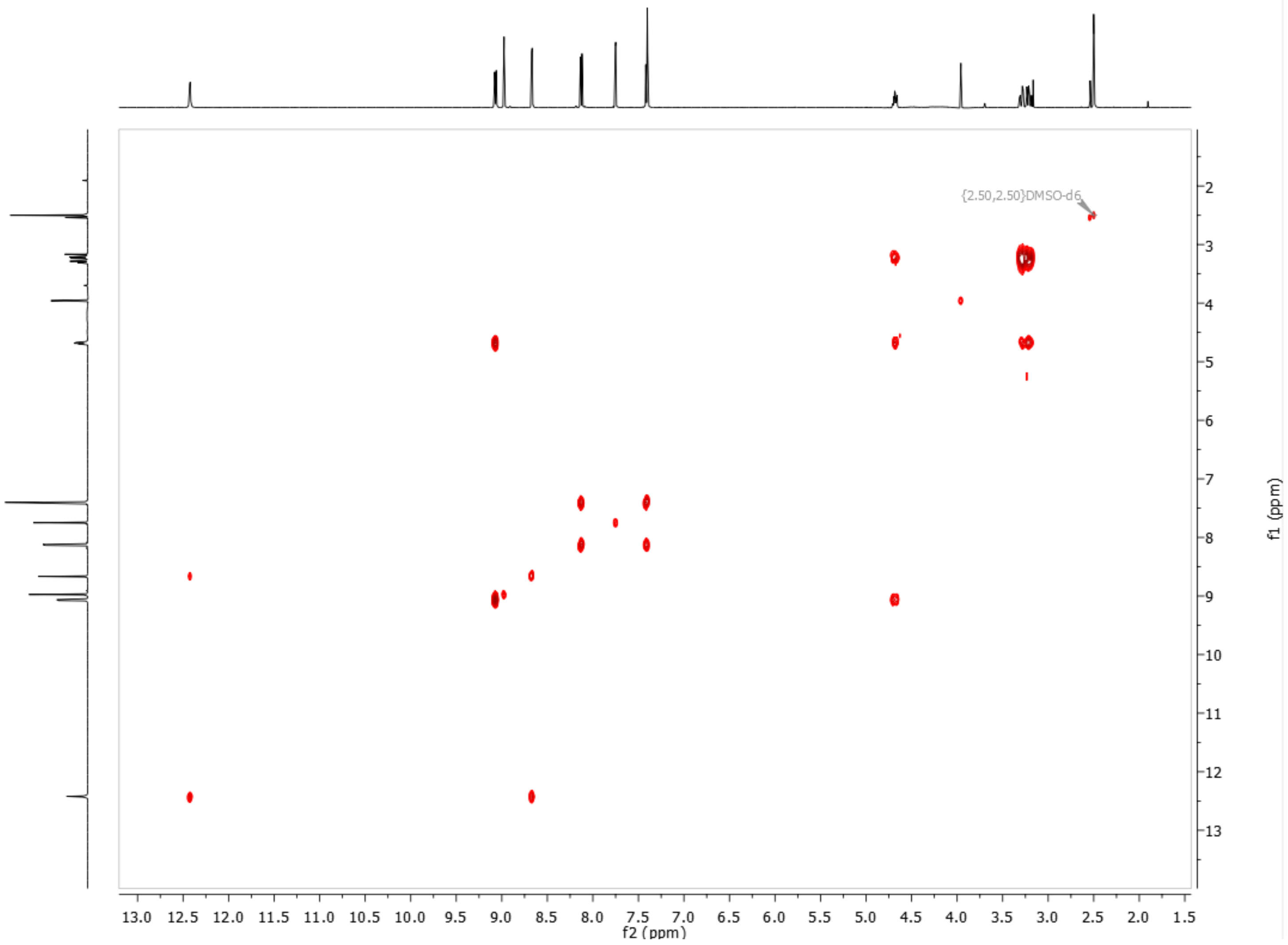

Figure S69. COSY NMR spectrum for 6-bromoindolyl-3-glyoxyl-L-histidine (**40**) recorded in DMSO-d_6

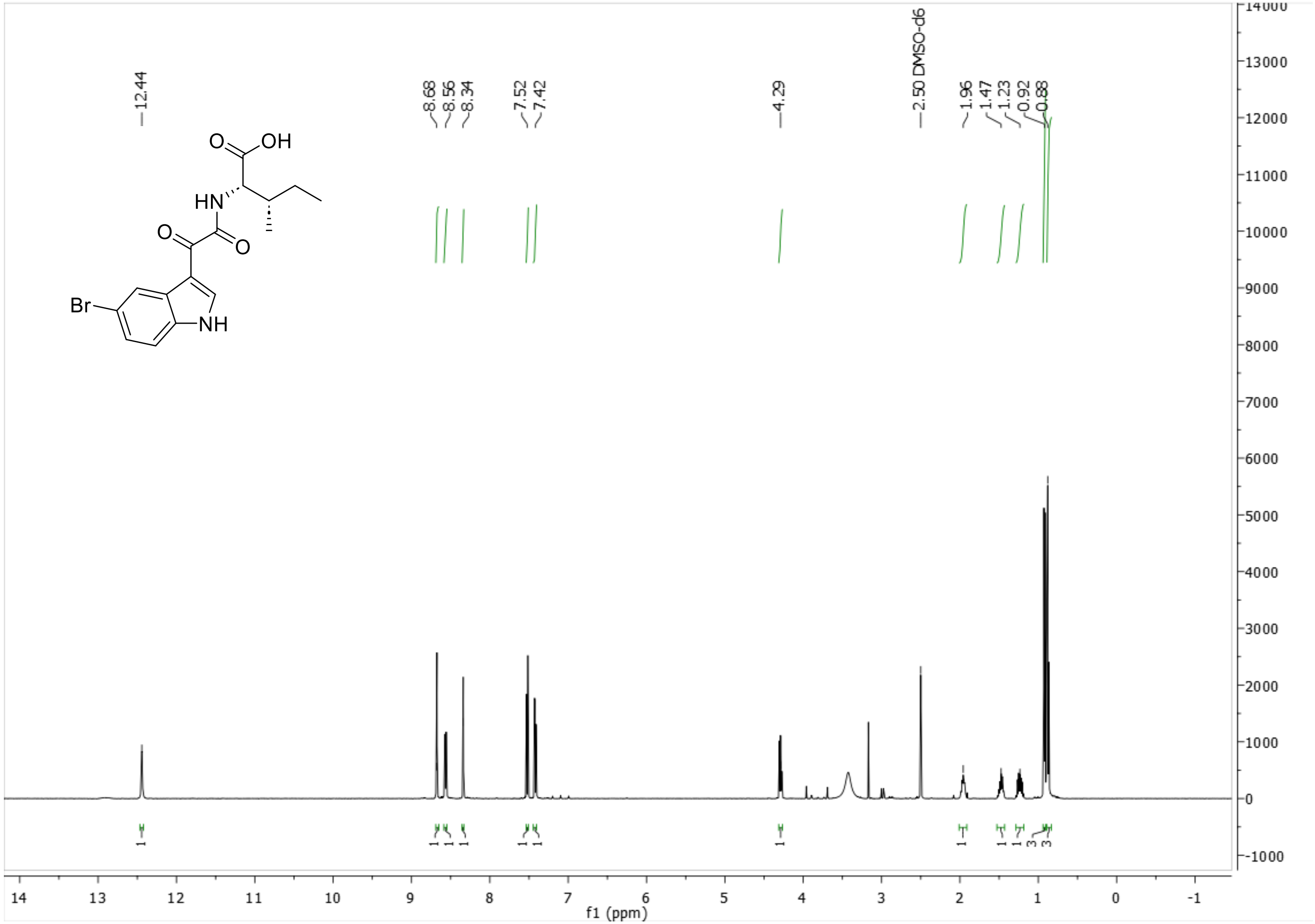

Figure S70. ^{1}H NMR spectrum for 5-bromoindolyl-3-glyoxyl-L-isoleucine (**41**) recorded in DMSO-d_6

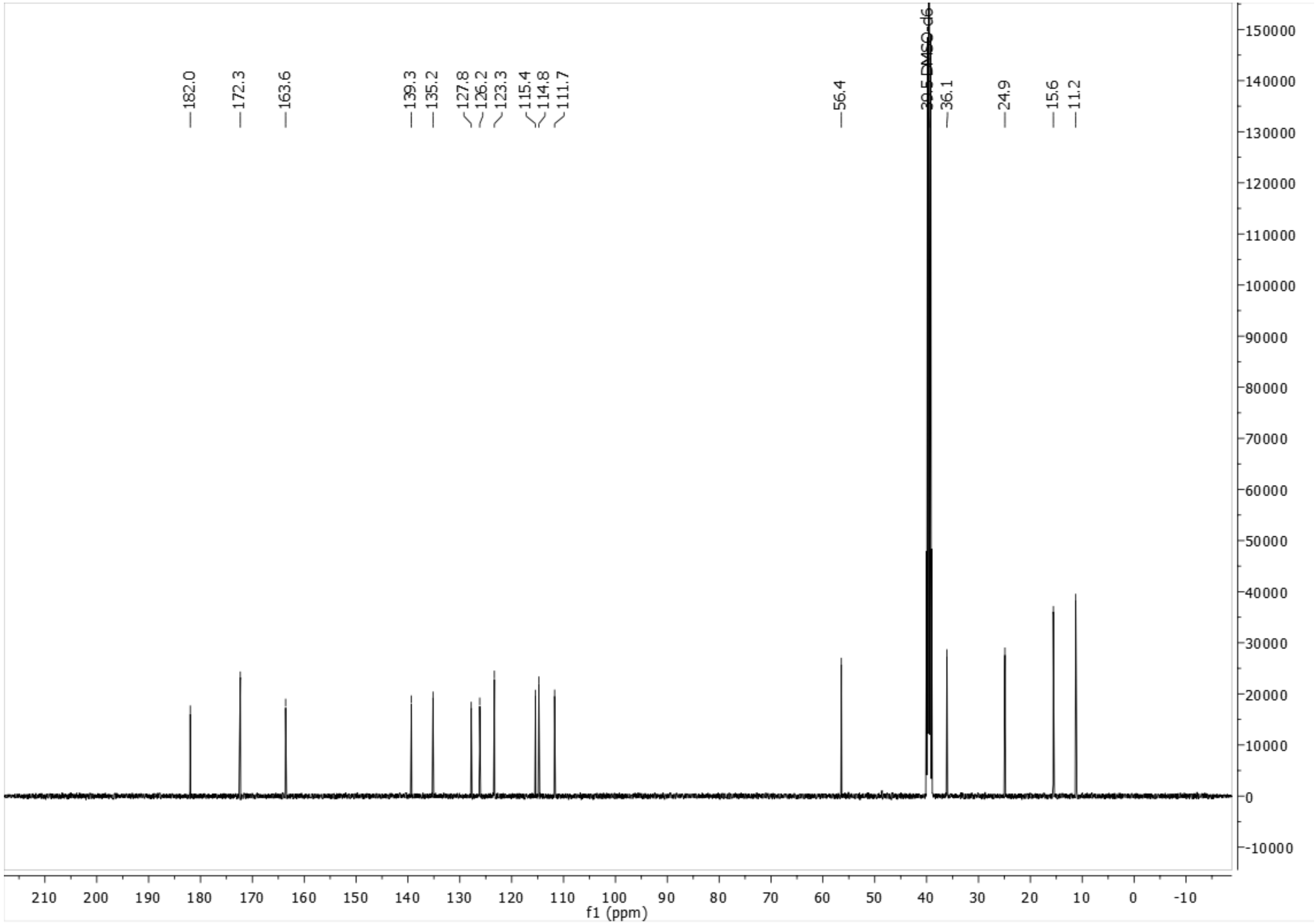

Figure S71. ^{13}C NMR spectrum for 5-bromoindolyl-3-glyoxyl-L-isoleucine (**41**) recorded in DMSO-d_6

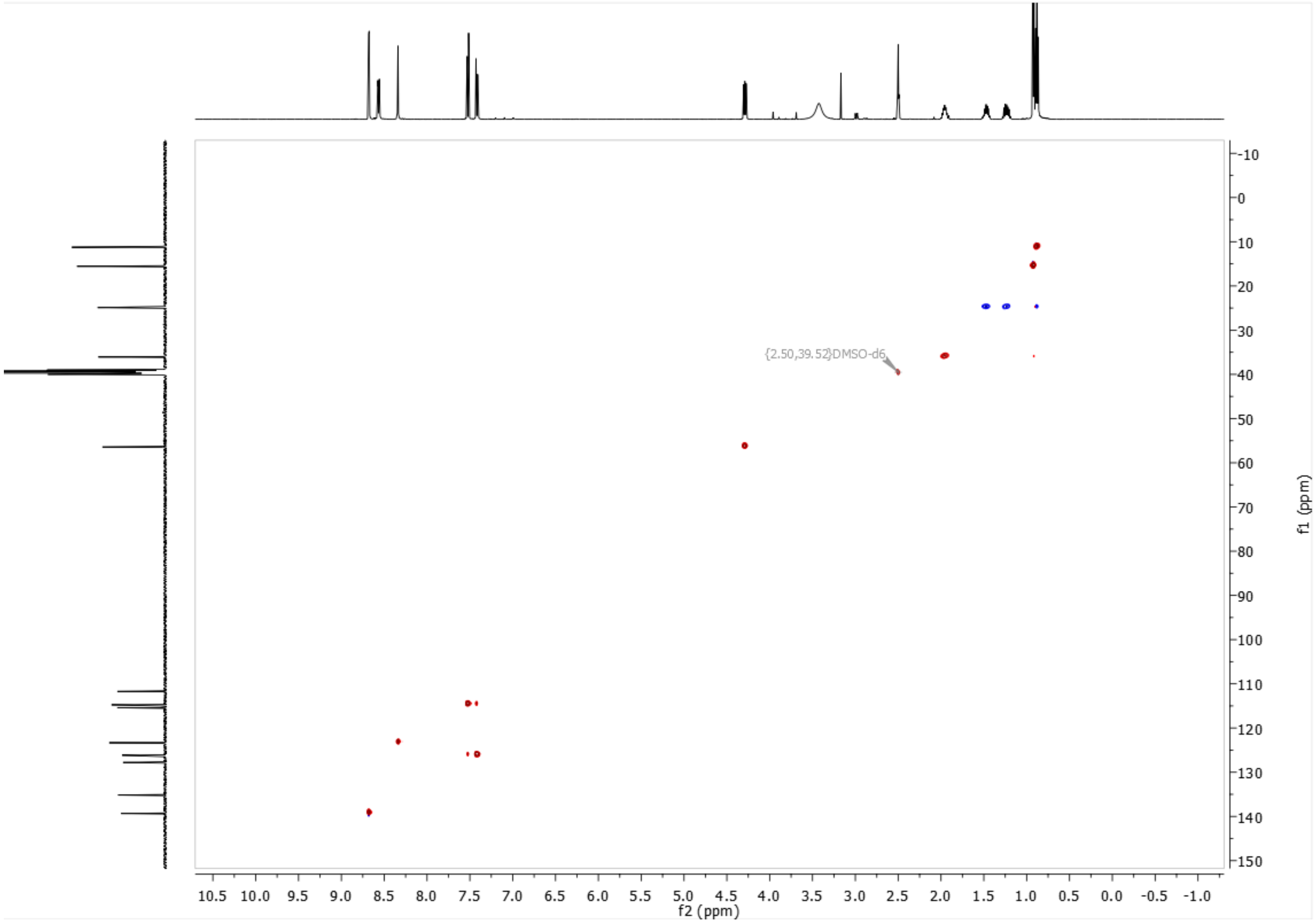

Figure S72. HSQC NMR spectrum for 5-bromoindolyl-3-glyoxyl-L-isoleucine (**41**) recorded in DMSO-d_6

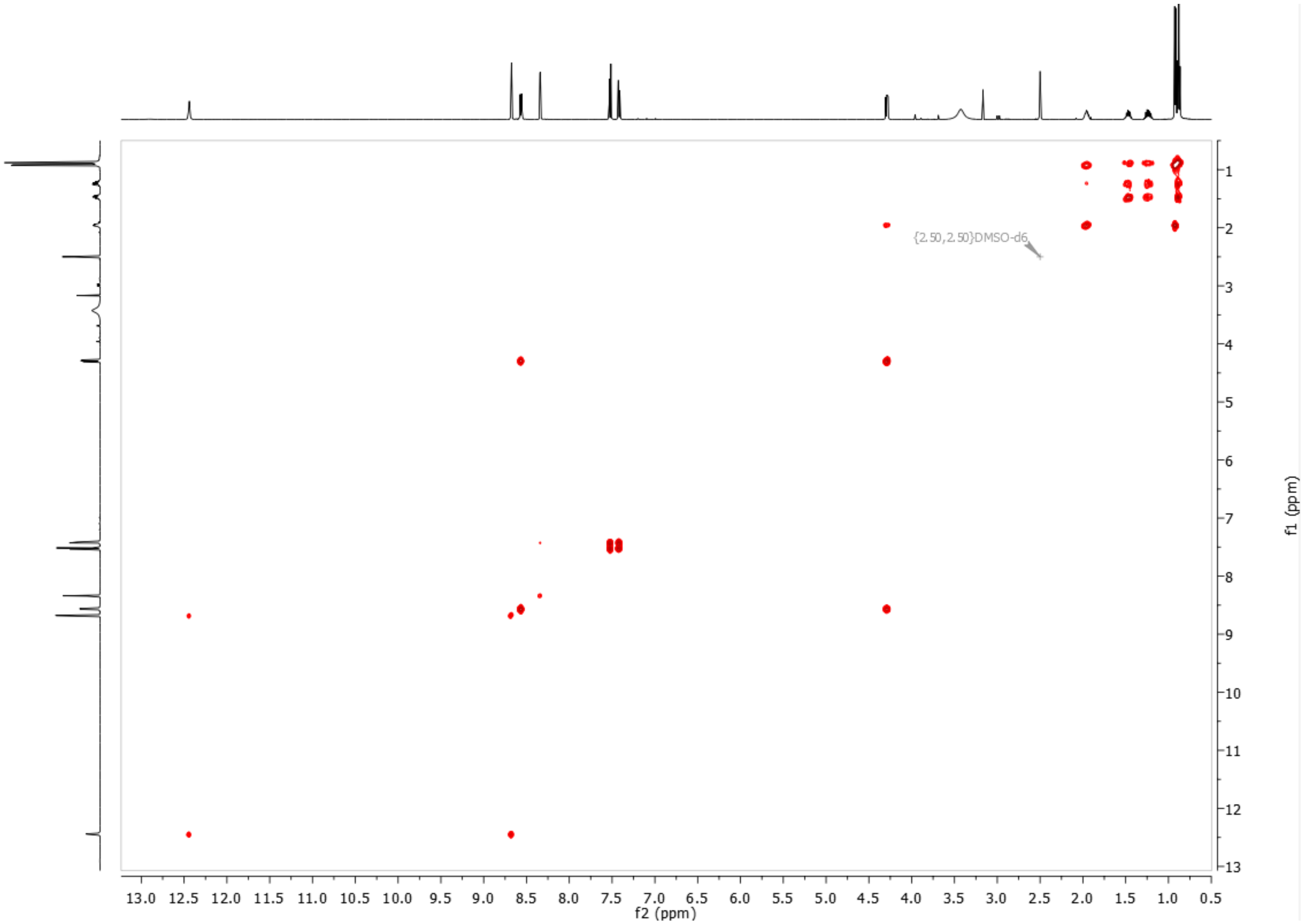

Figure S73. COSY NMR spectrum for 5-bromoindolyl-3-glyoxyl-L-isoleucine (**41**) recorded in DMSO-d_6

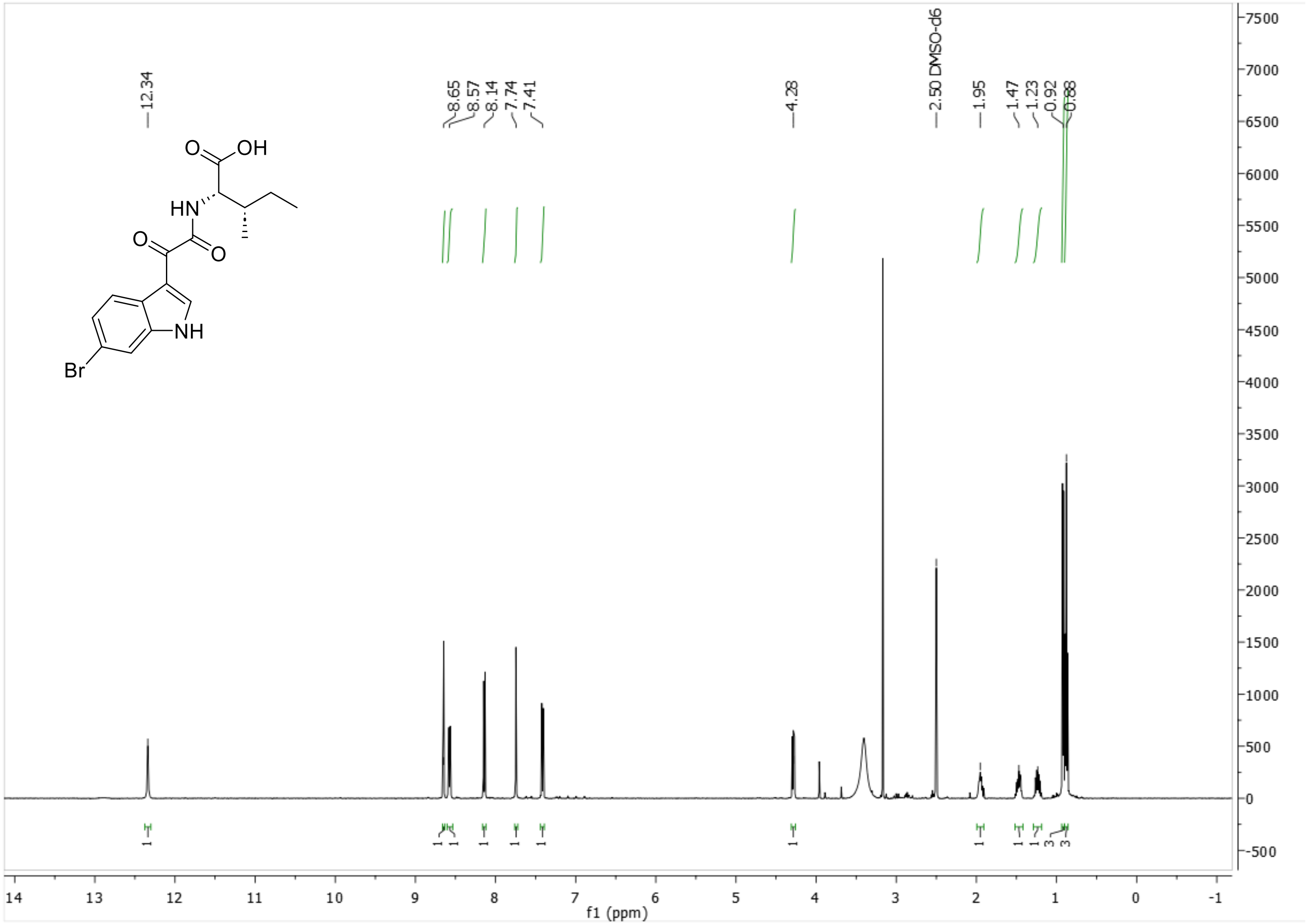

Figure S74. ^{1}H NMR spectrum for 6-bromoindolyl-3-glyoxyl-L-isoleucine (**42**) recorded in DMSO-d_6

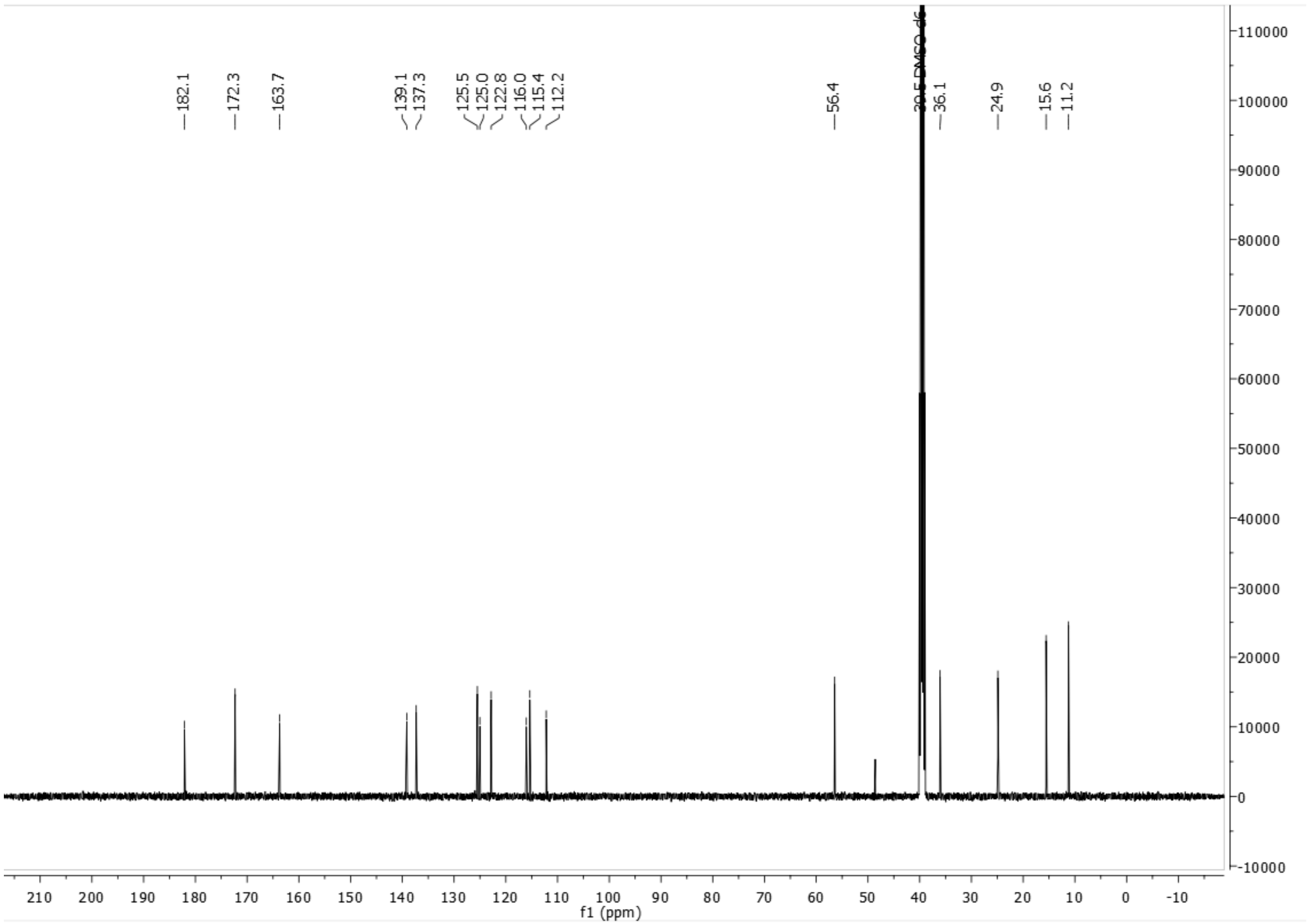

Figure S75. ^{13}C NMR spectrum for 6-bromoindolyl-3-glyoxyl-L-isoleucine (**42**) recorded in DMSO-d_6

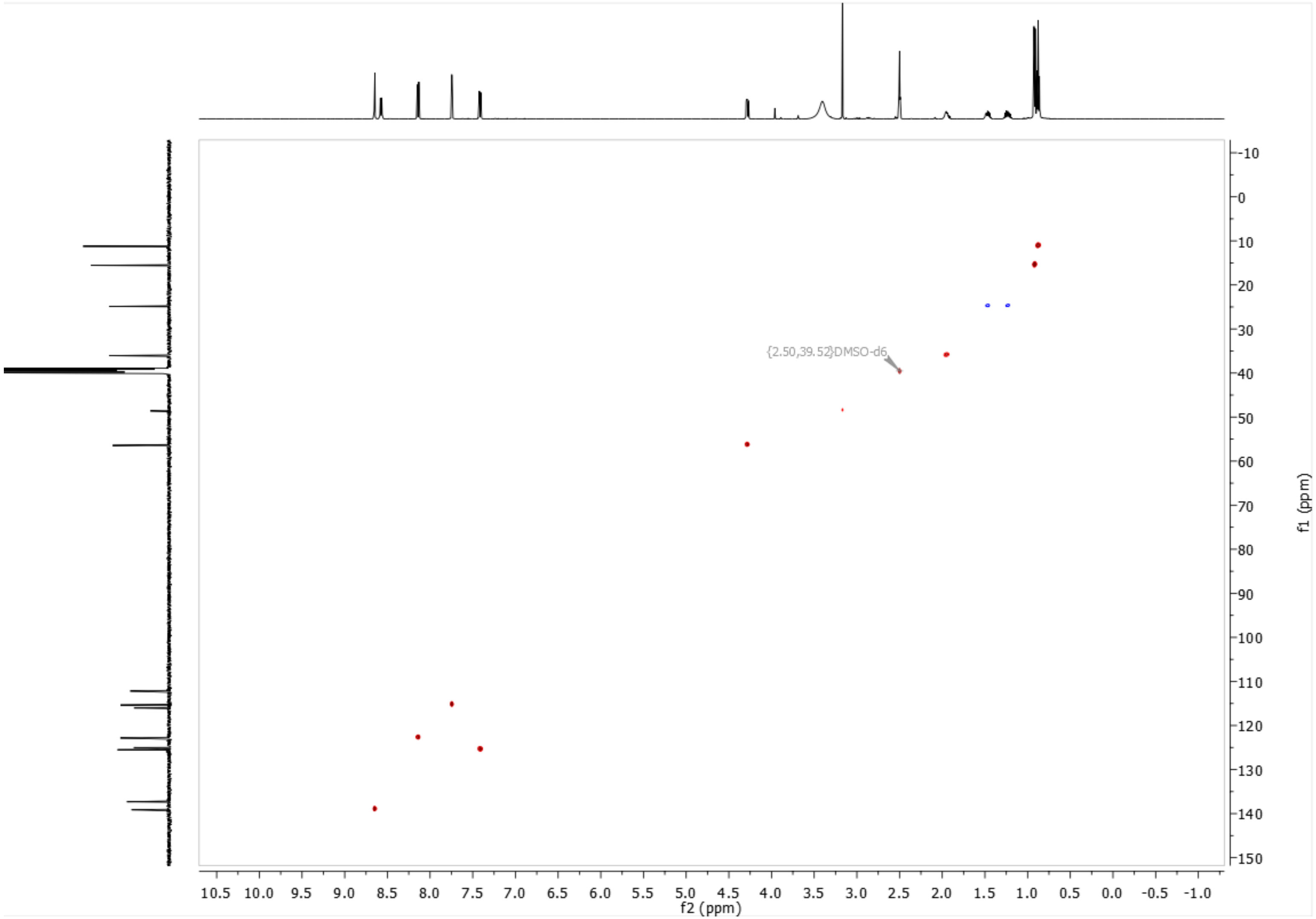

Figure S76. HSQC NMR spectrum for 6-bromoindolyl-3-glyoxyl-L-isoleucine (**42**) recorded in DMSO-d_6

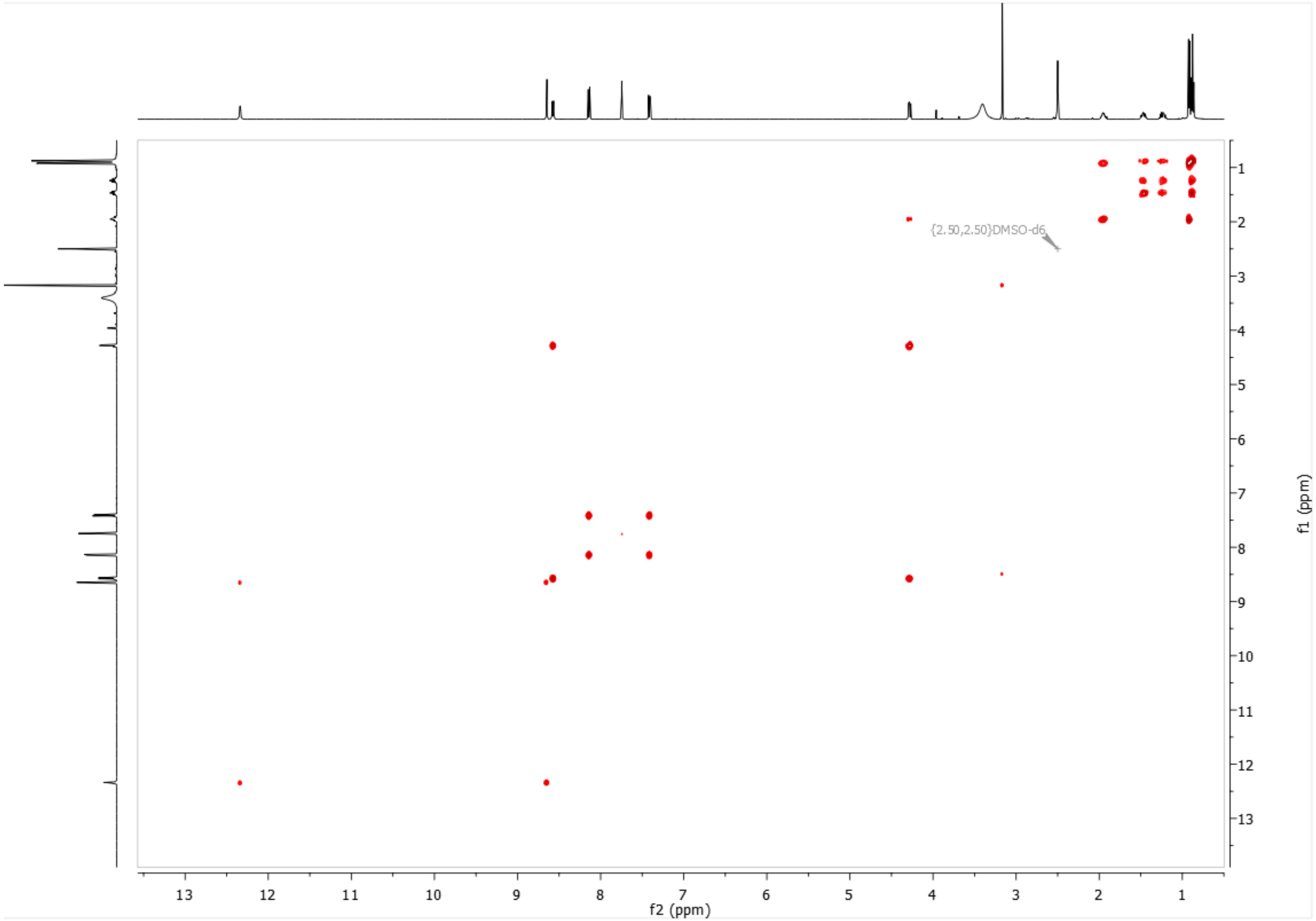

Figure S77. COSY NMR spectrum for 6-bromoindolyl-3-glyoxyl-L-isoleucine (**42**) recorded in DMSO-d_6

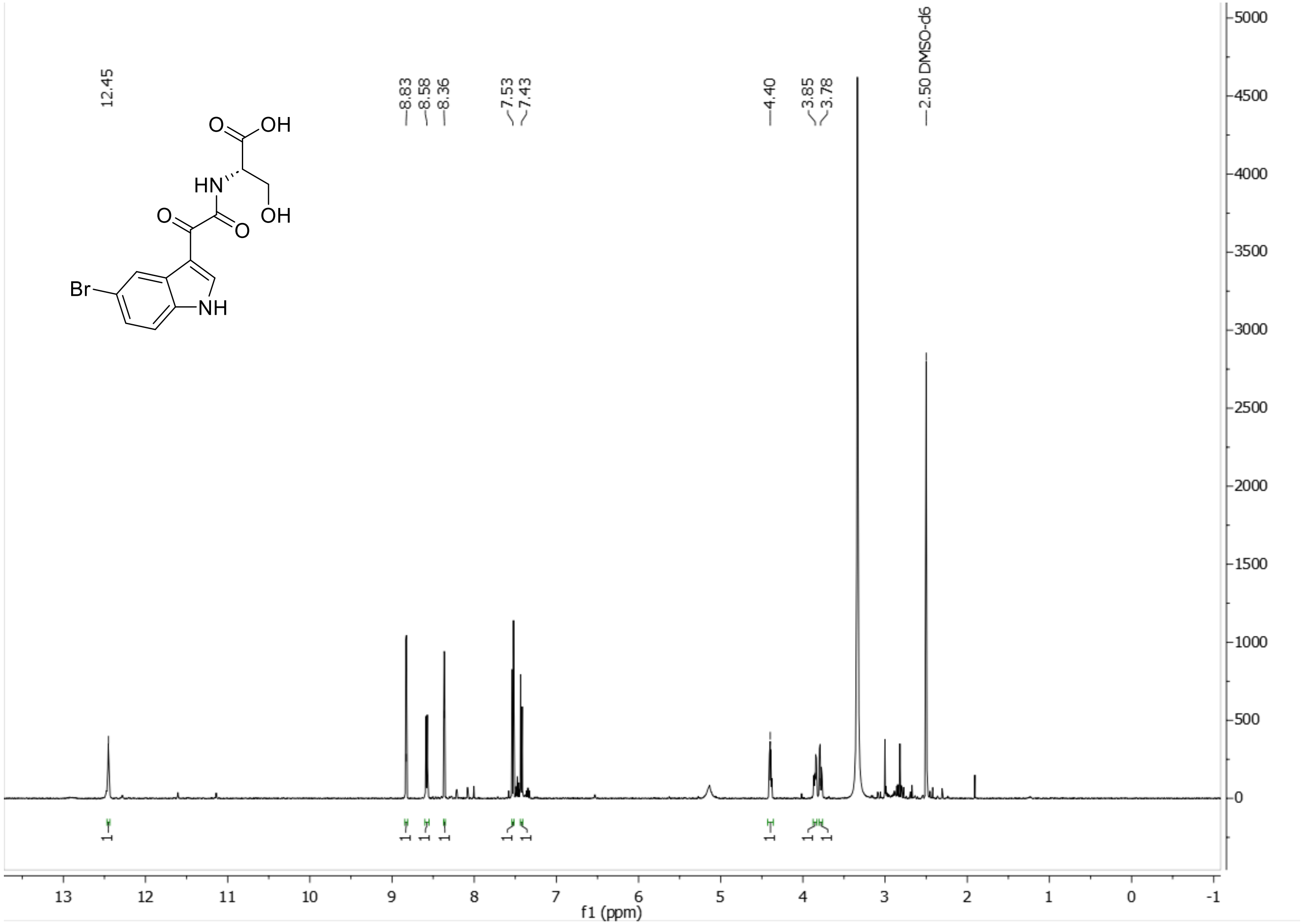

Figure S78. ^{1}H NMR spectrum for 5-bromoindolyl-3-glyoxyl-L-serine (**43**) recorded in DMSO-d_6

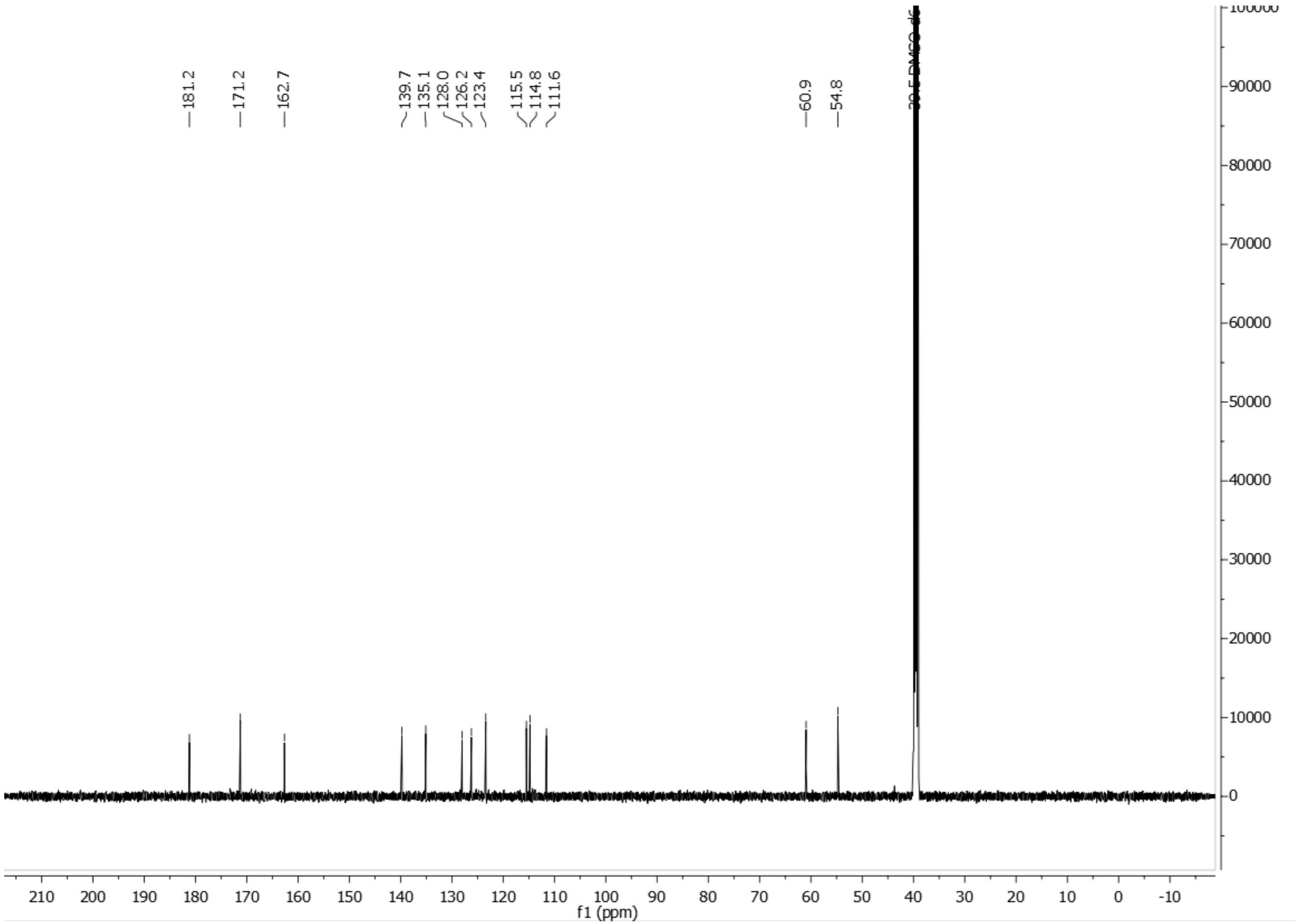

Figure S79. ^{13}C NMR spectrum for 5-bromoindolyl-3-glyoxyl-L-serine (**43**) recorded in DMSO-*d*$_6$

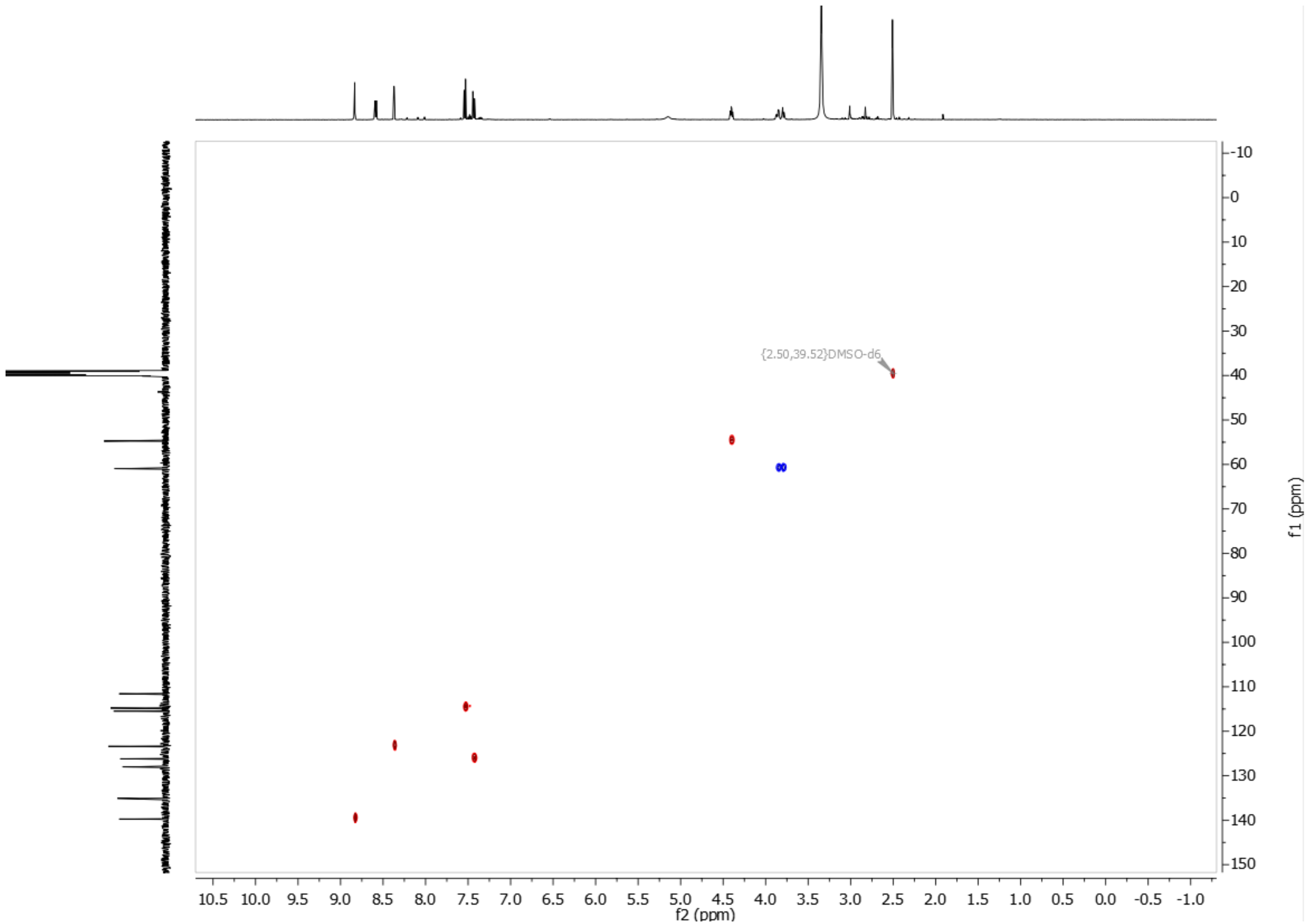

Figure S80. HSQC NMR spectrum for 5-bromoindolyl-3-glyoxyl-L-serine (**43**) recorded in DMSO-d_6

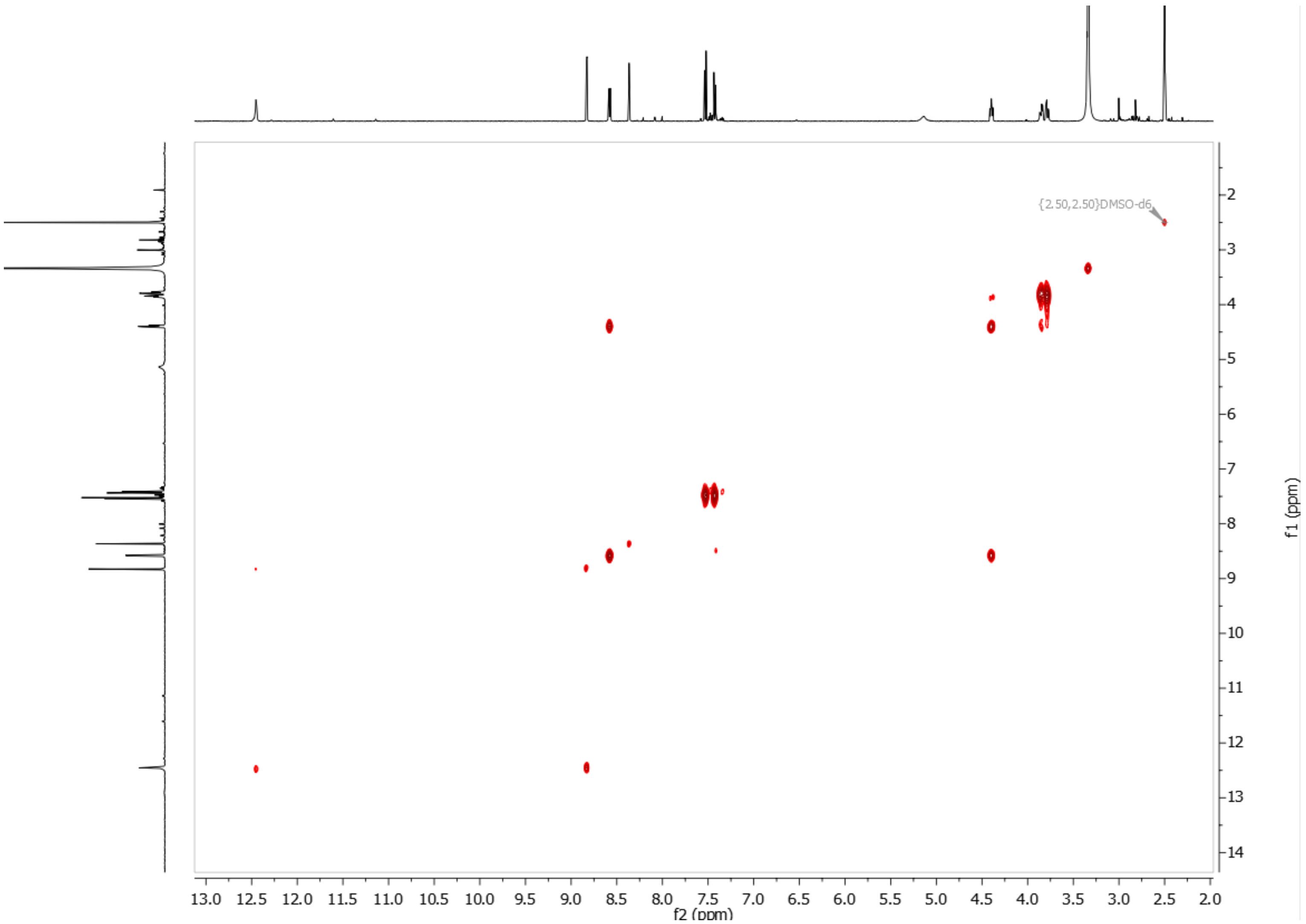

Figure S81. COSY NMR spectrum for 5-bromoindolyl-3-glyoxyl-L-serine (**43**) recorded in DMSO-d_6

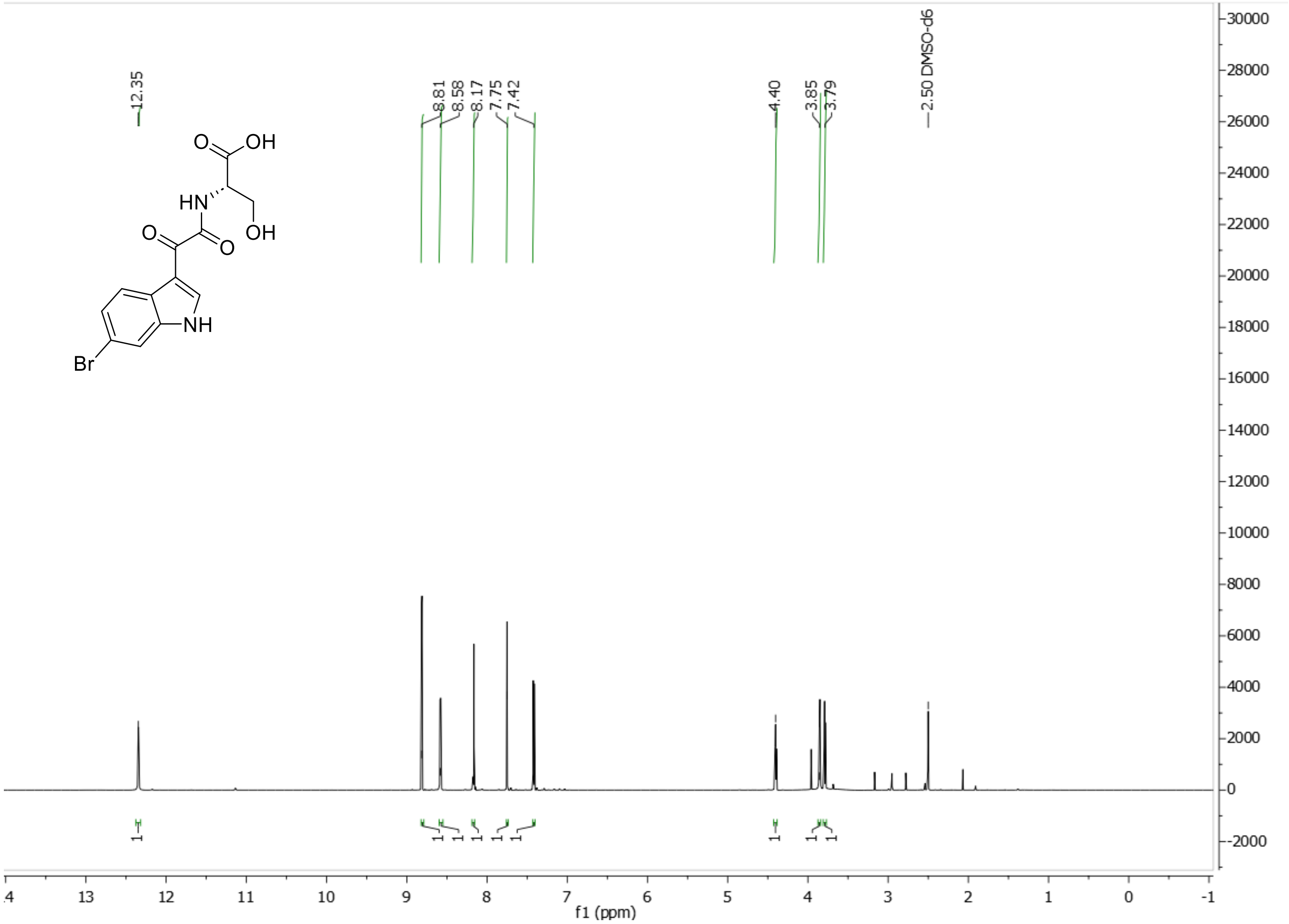

Figure S82. ^{1}H NMR spectrum for 6-bromoindolyl-3-glyoxyl-L-serine (**44**) recorded in DMSO-d_6

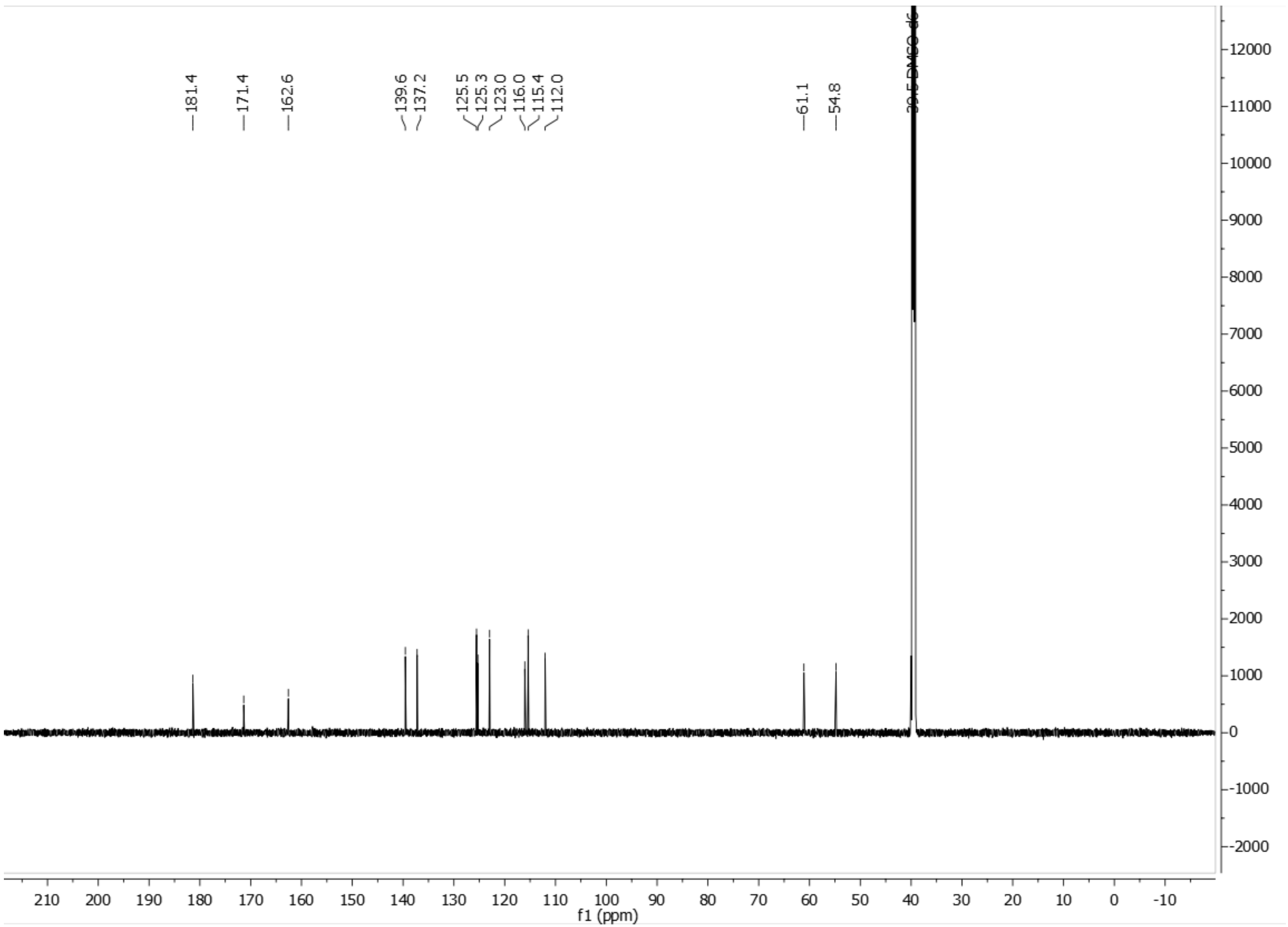

Figure S83. ^{13}C NMR spectrum for 6-bromoindolyl-3-glyoxyl-L-serine (**44**) recorded in DMSO-d_6

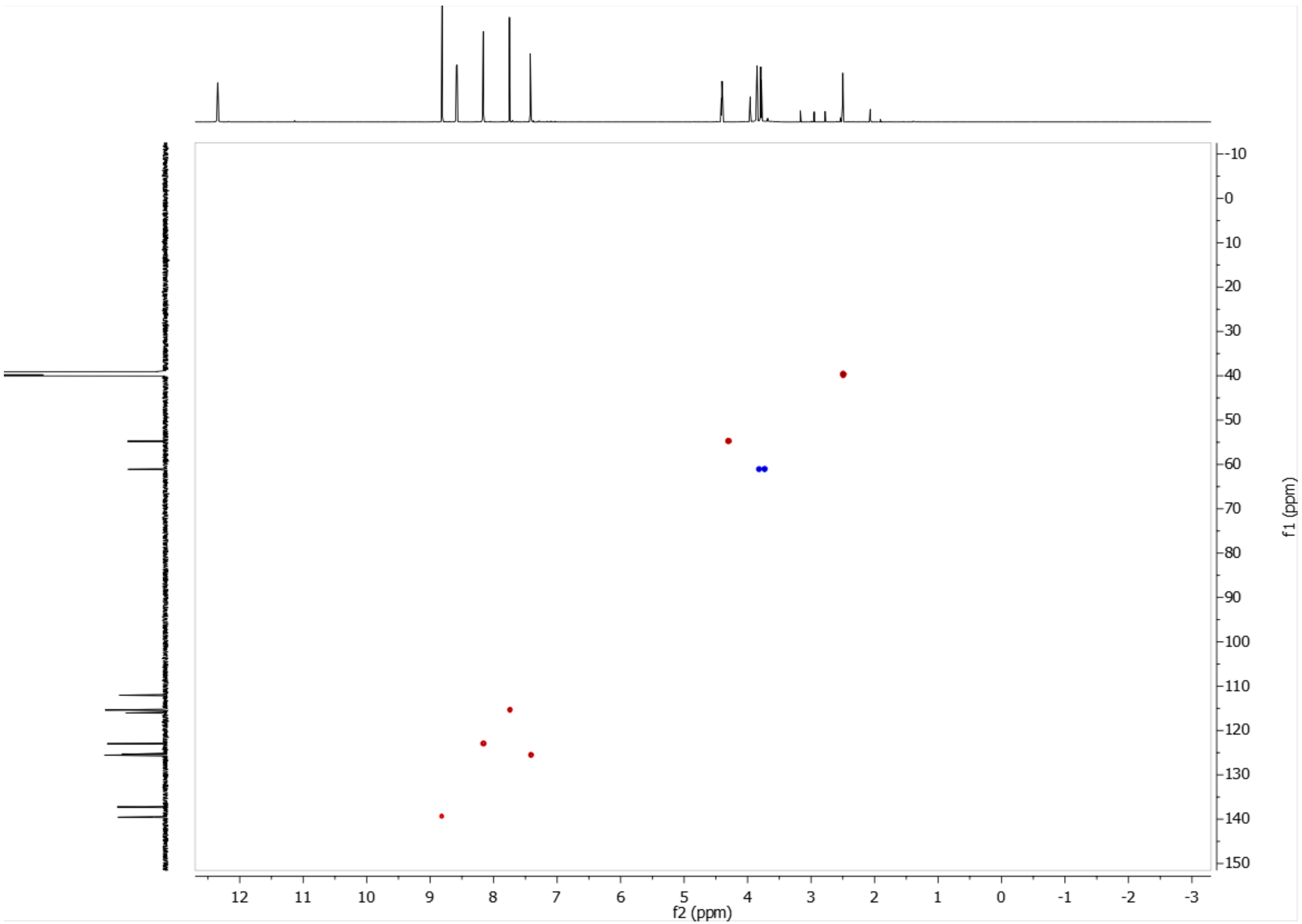

Figure S84. HSQC NMR spectrum for 6-bromoindolyl-3-glyoxyl-L-serine (**44**) recorded in DMSO-d_6

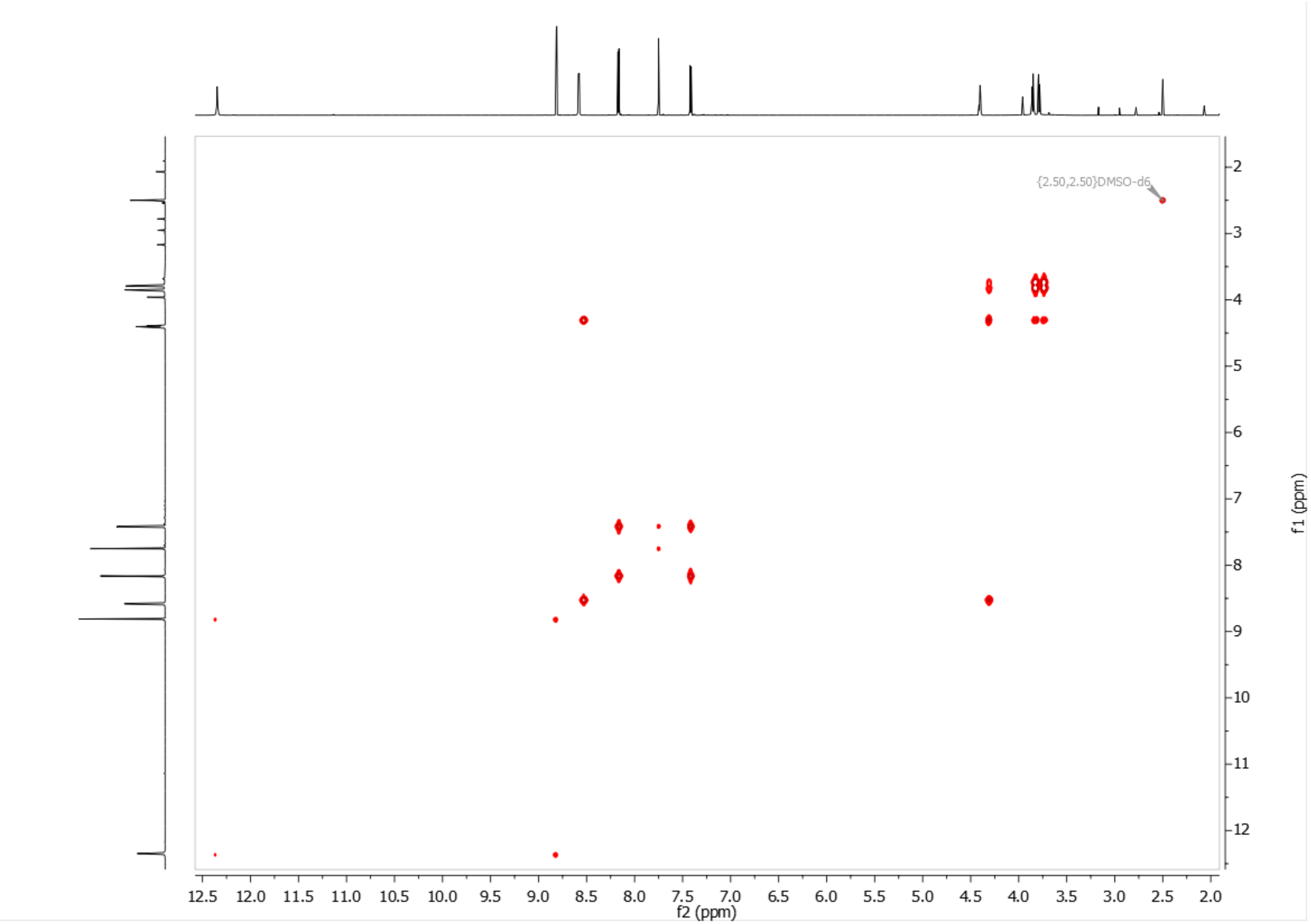

Figure S85. COSY NMR spectrum for 6-bromoindolyl-3-glyoxyl-L-serine (**44**) recorded in DMSO-d_6

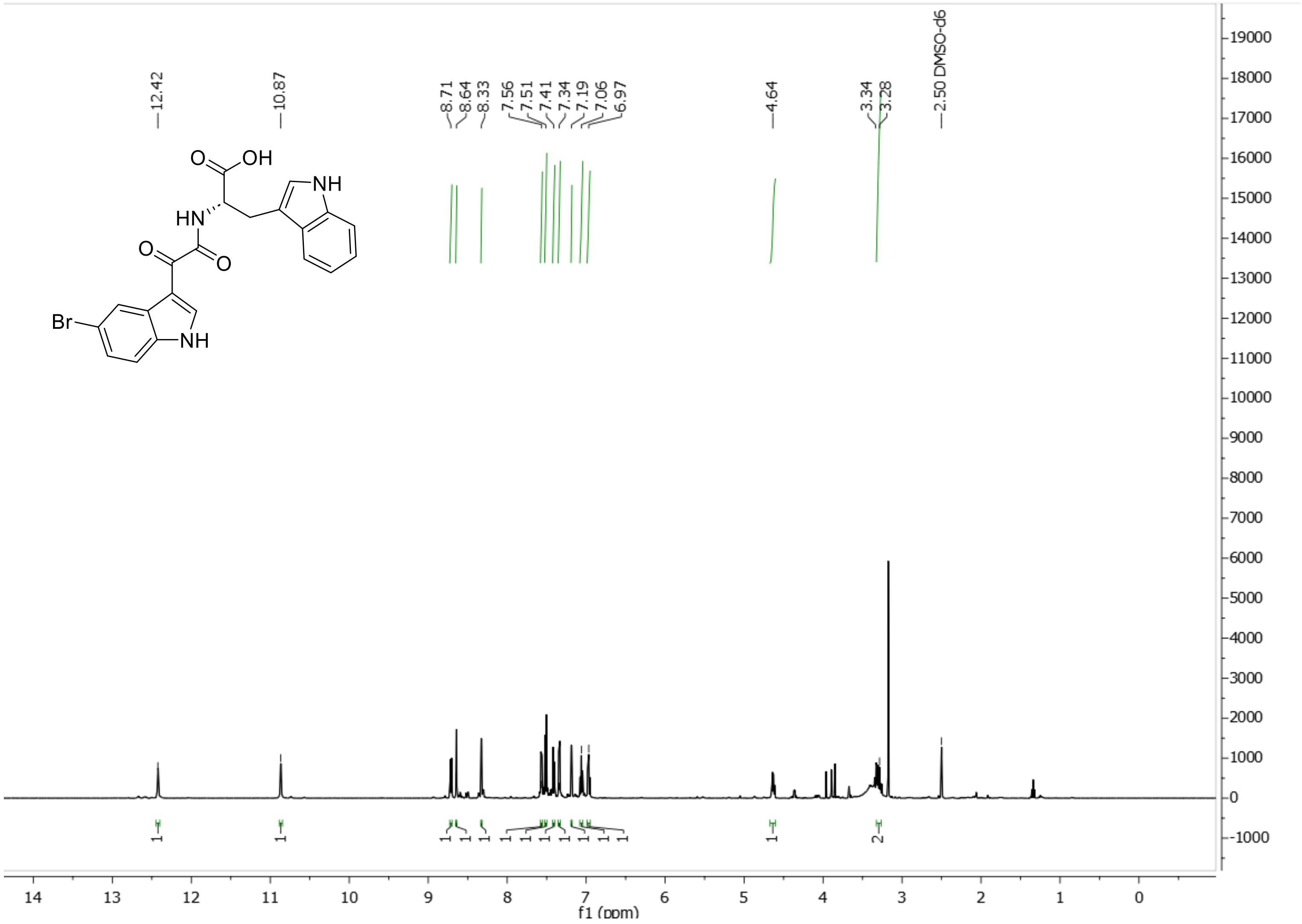

Figure S86. ^{1}H NMR spectrum for 5-bromoindolyl-3-glyoxyl-L-tryptophan (**45**) recorded in DMSO-d_6

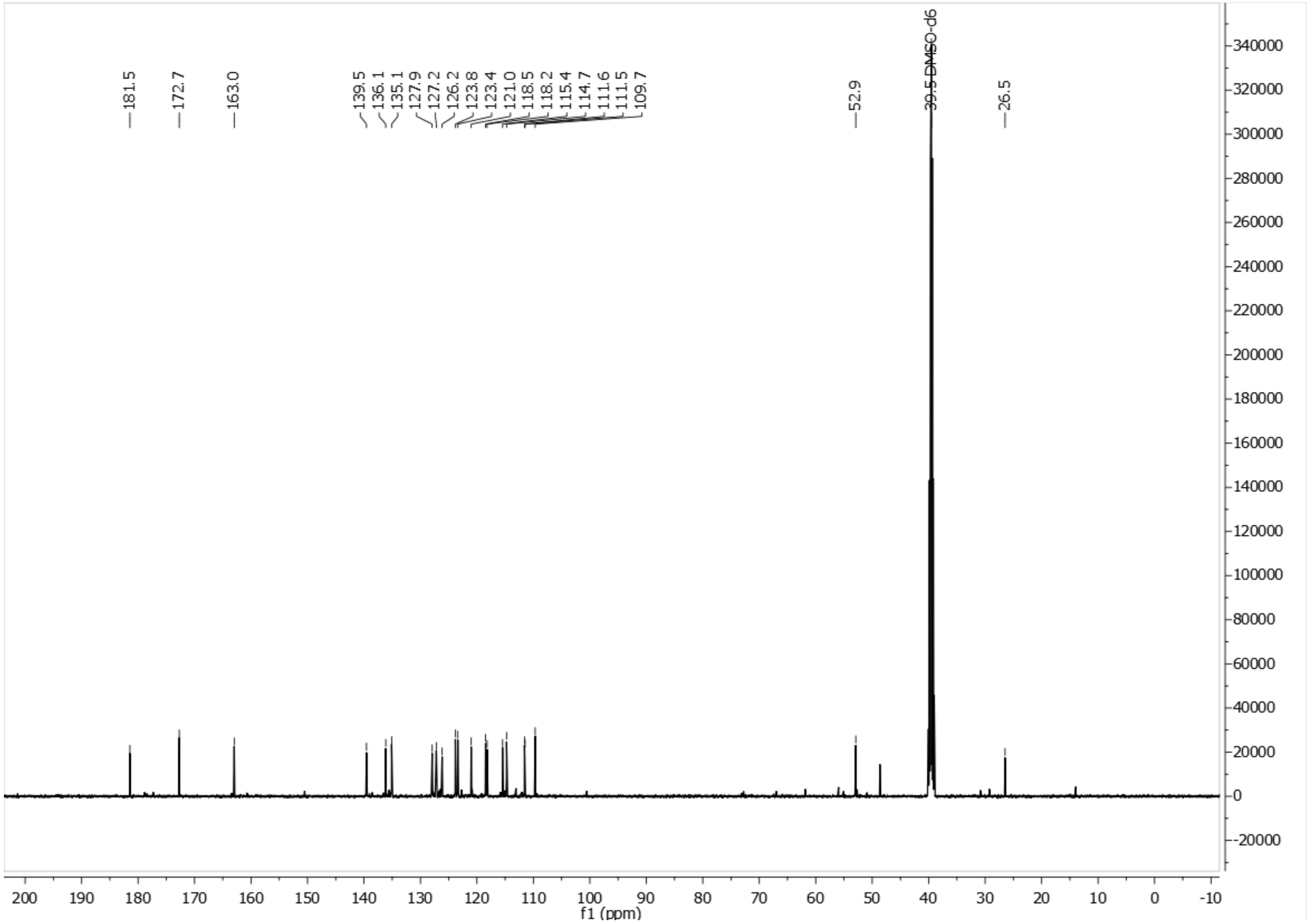

Figure S87. ^{13}C NMR spectrum for 5-bromoindolyl-3-glyoxyl-L-tryptophan (**45**) recorded in DMSO-d_6

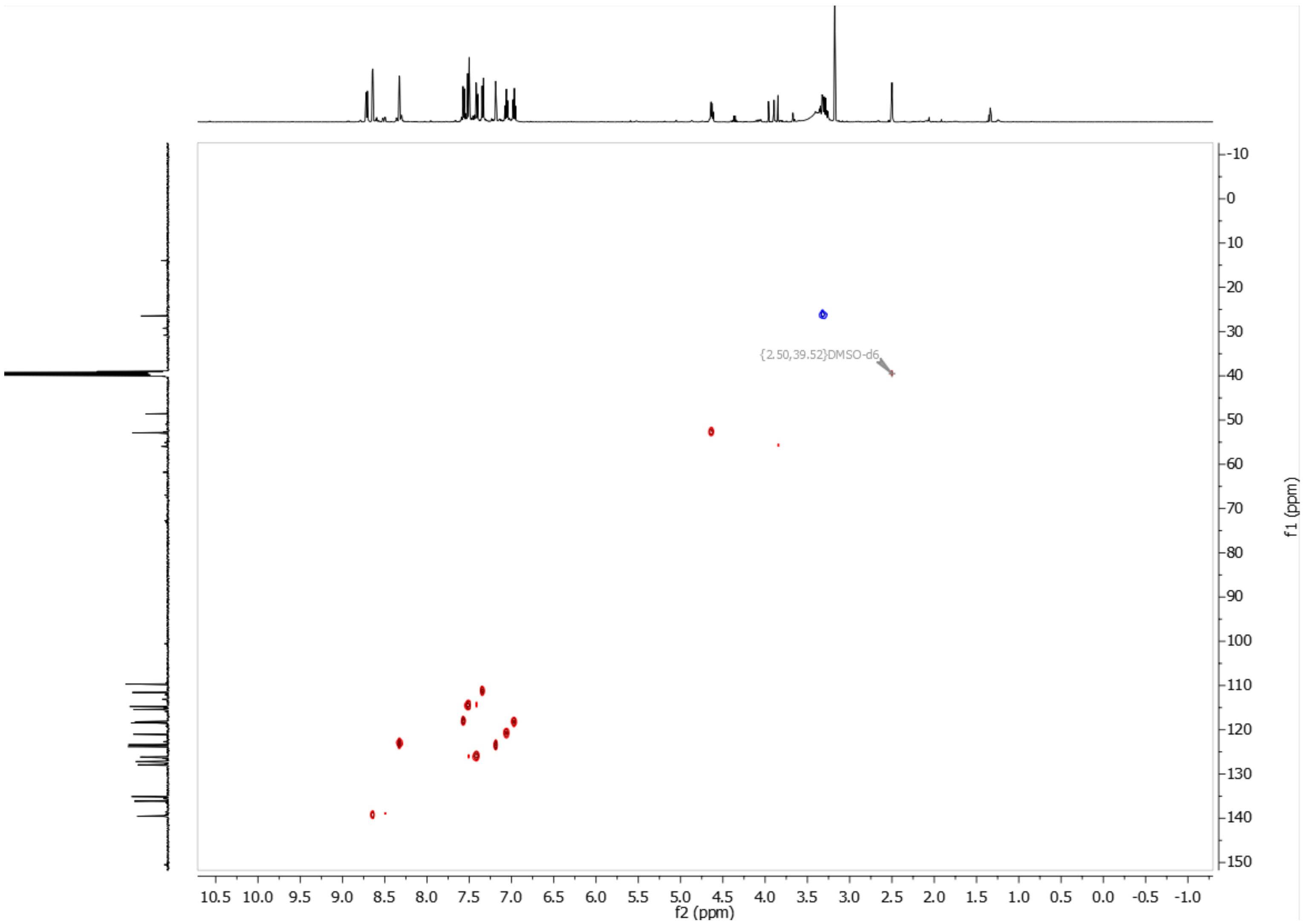

Figure S88. HSQC NMR spectrum for 5-bromoindolyl-3-glyoxyl-L-tryptophan (**45**) recorded in DMSO-d_6

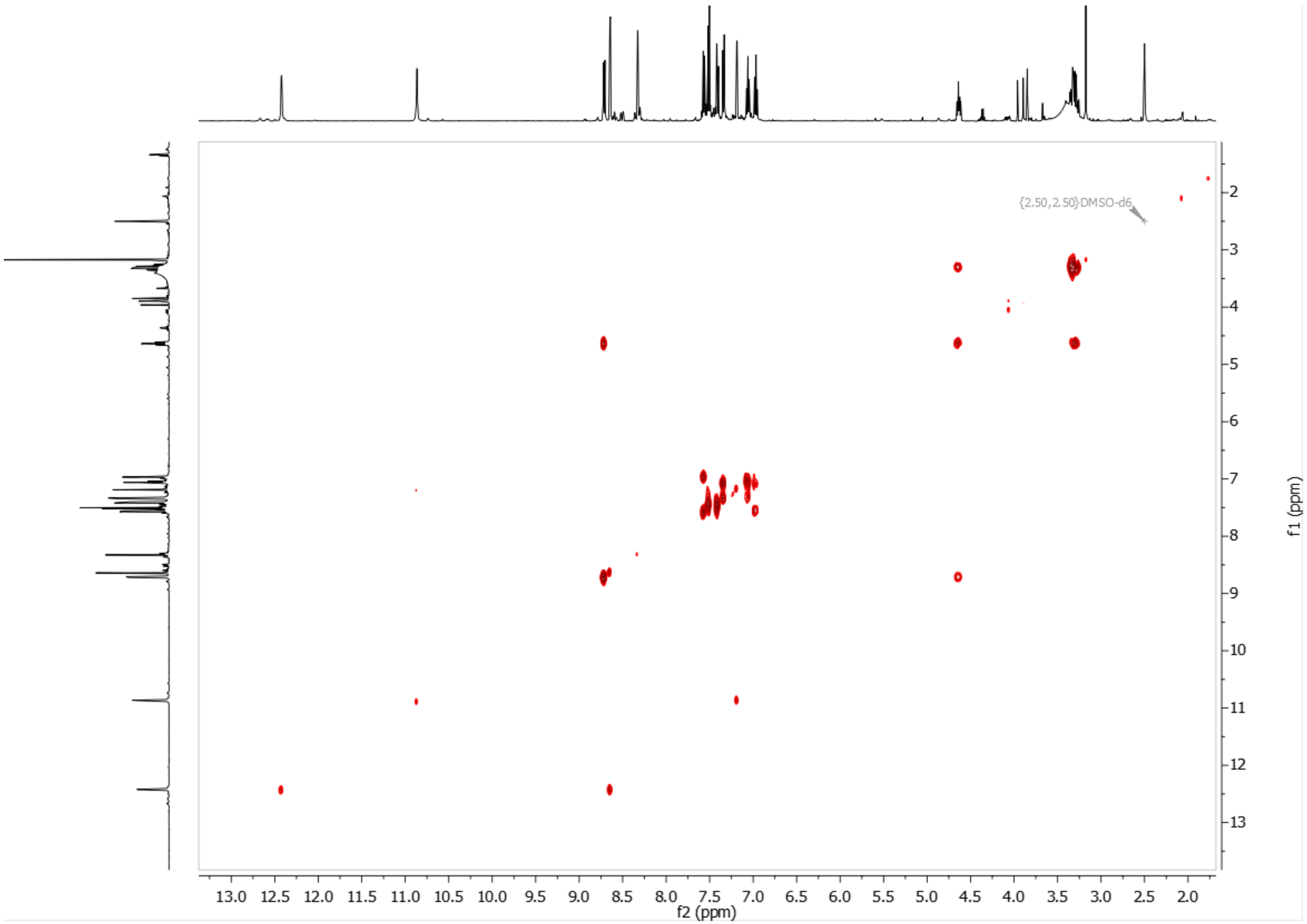

Figure S89. COSY NMR spectrum for 5-bromoindolyl-3-glyoxyl-L-tryptophan (**45**) recorded in DMSO-d_6

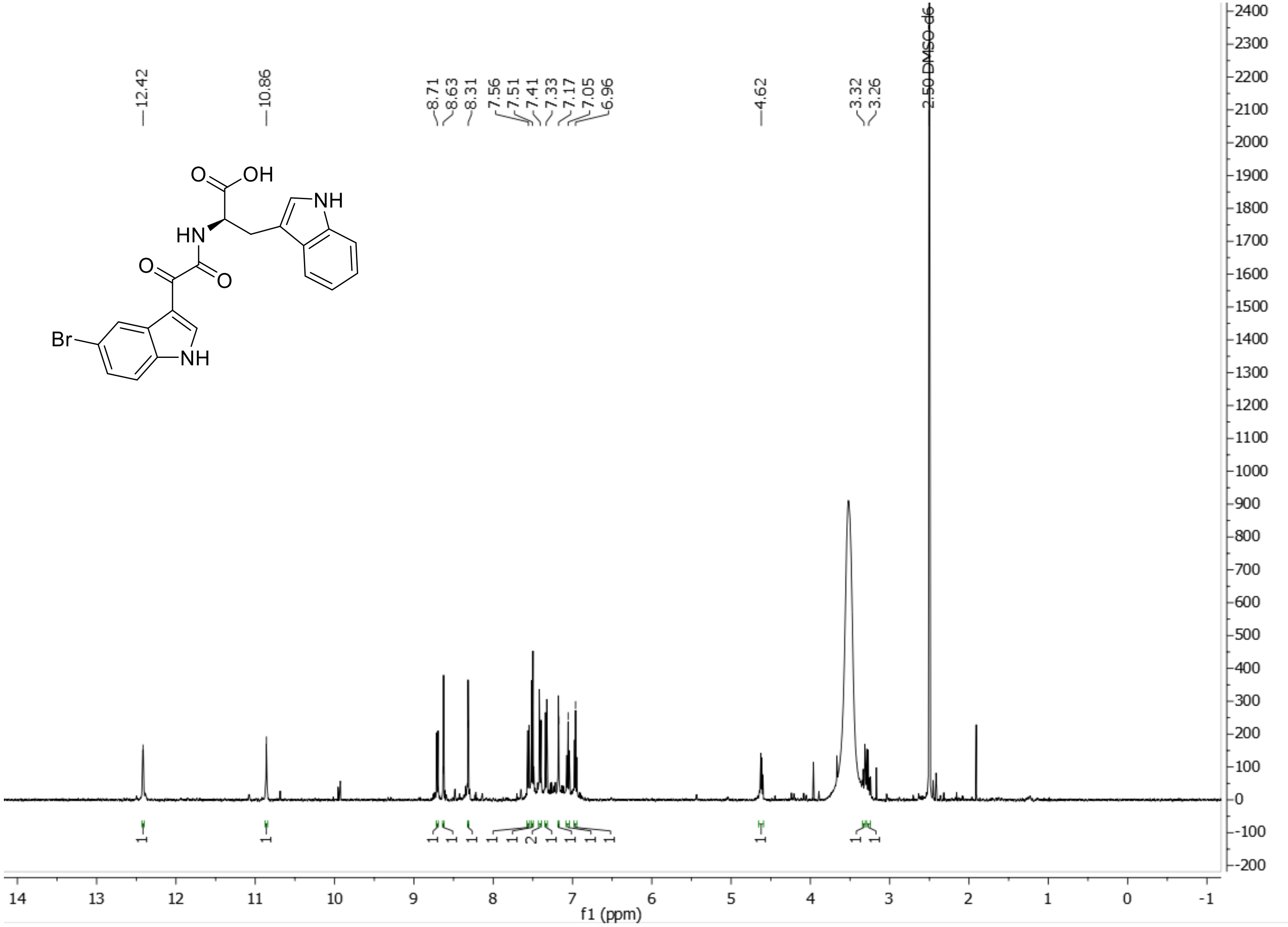

Figure S90. ^{1}H NMR spectrum for 5-bromoindolyl-3-glyoxyl-D-tryptophan (**46**) recorded in DMSO-d_6

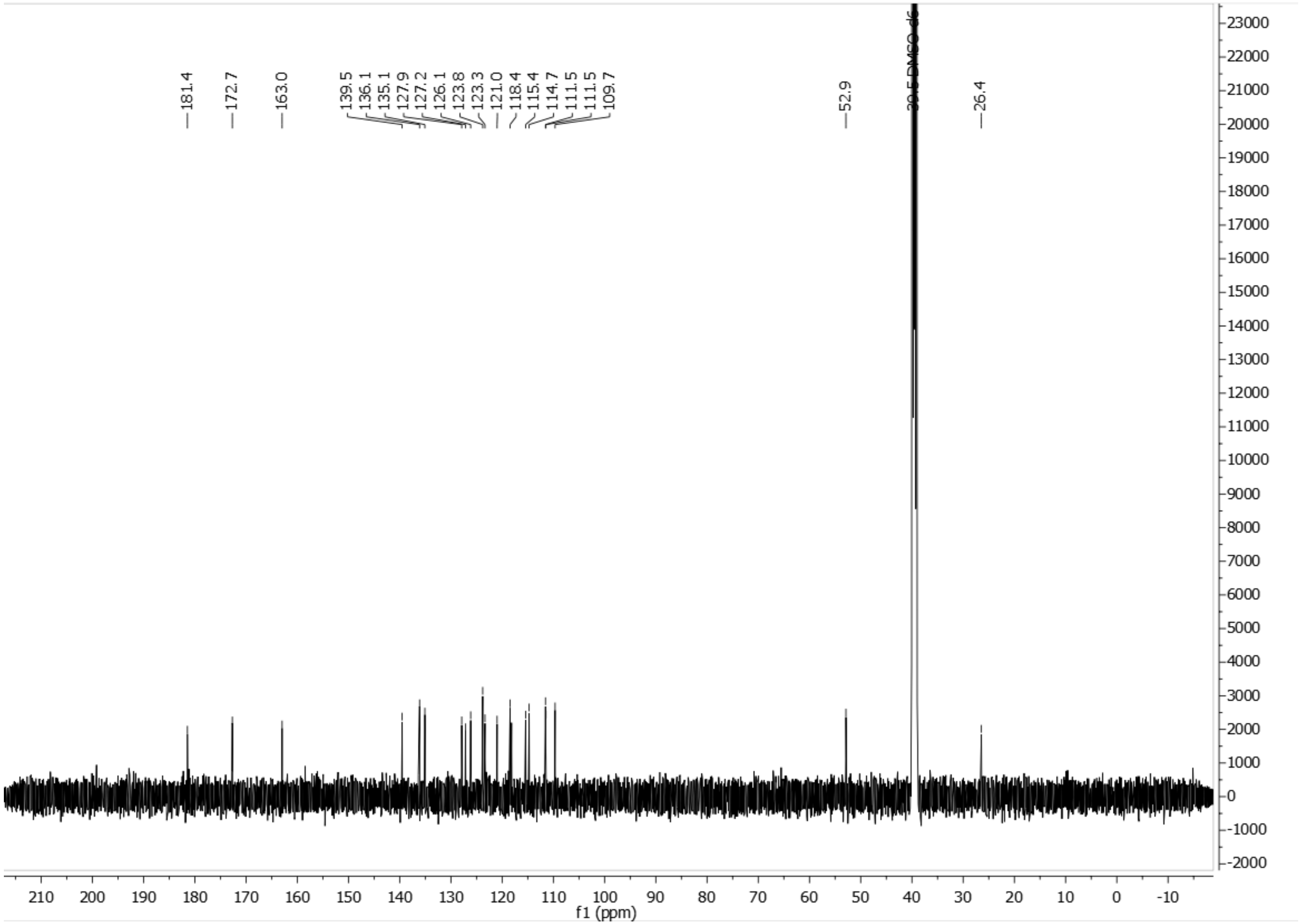

Figure S91. ^{13}C NMR spectrum for 5-bromoindolyl-3-glyoxyl-D-tryptophan (**46**) recorded in DMSO-d_6

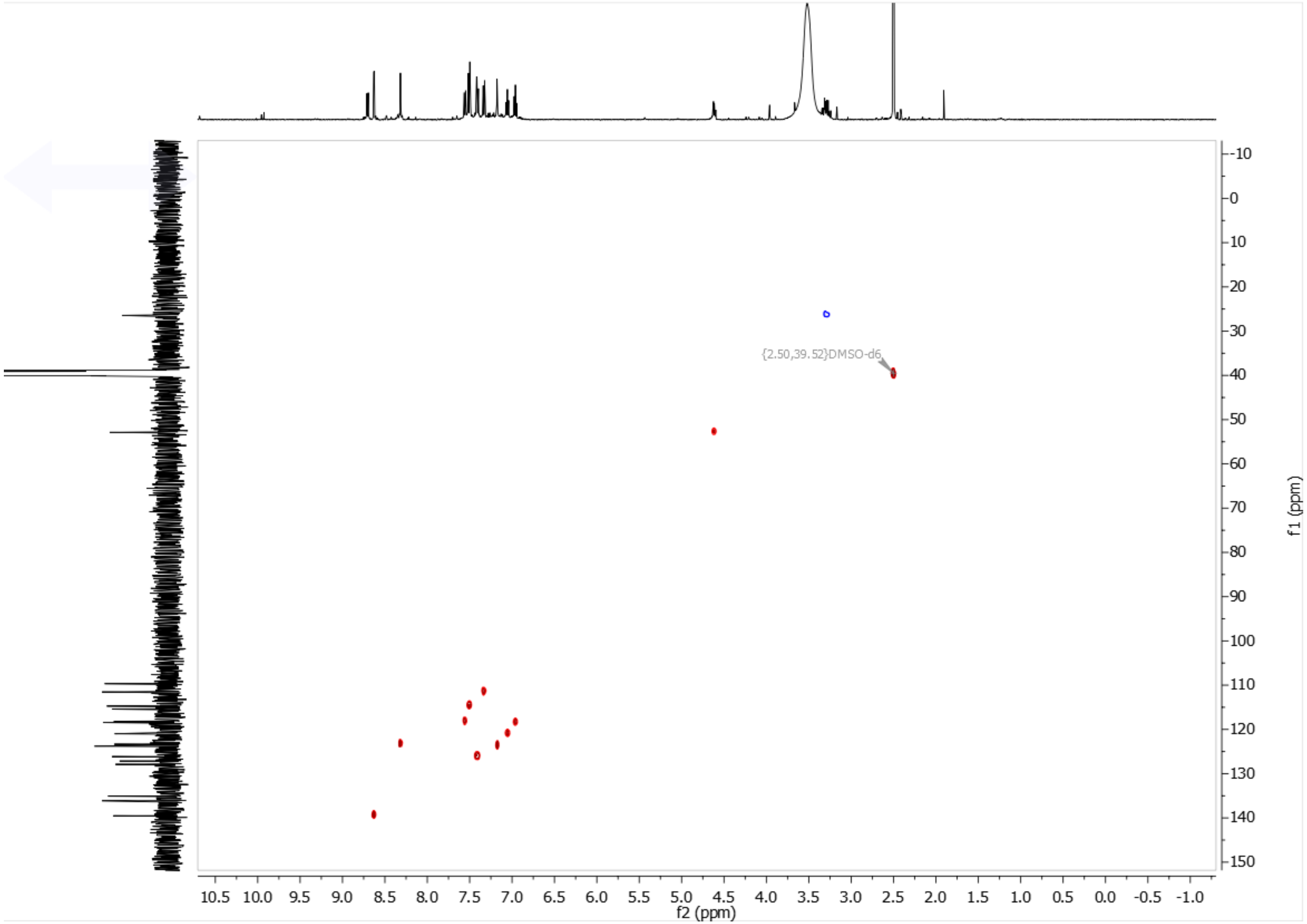

Figure S92. HSQC NMR spectrum for 5-bromoindolyl-3-glyoxyl-D-tryptophan (**46**) recorded in DMSO-d_6

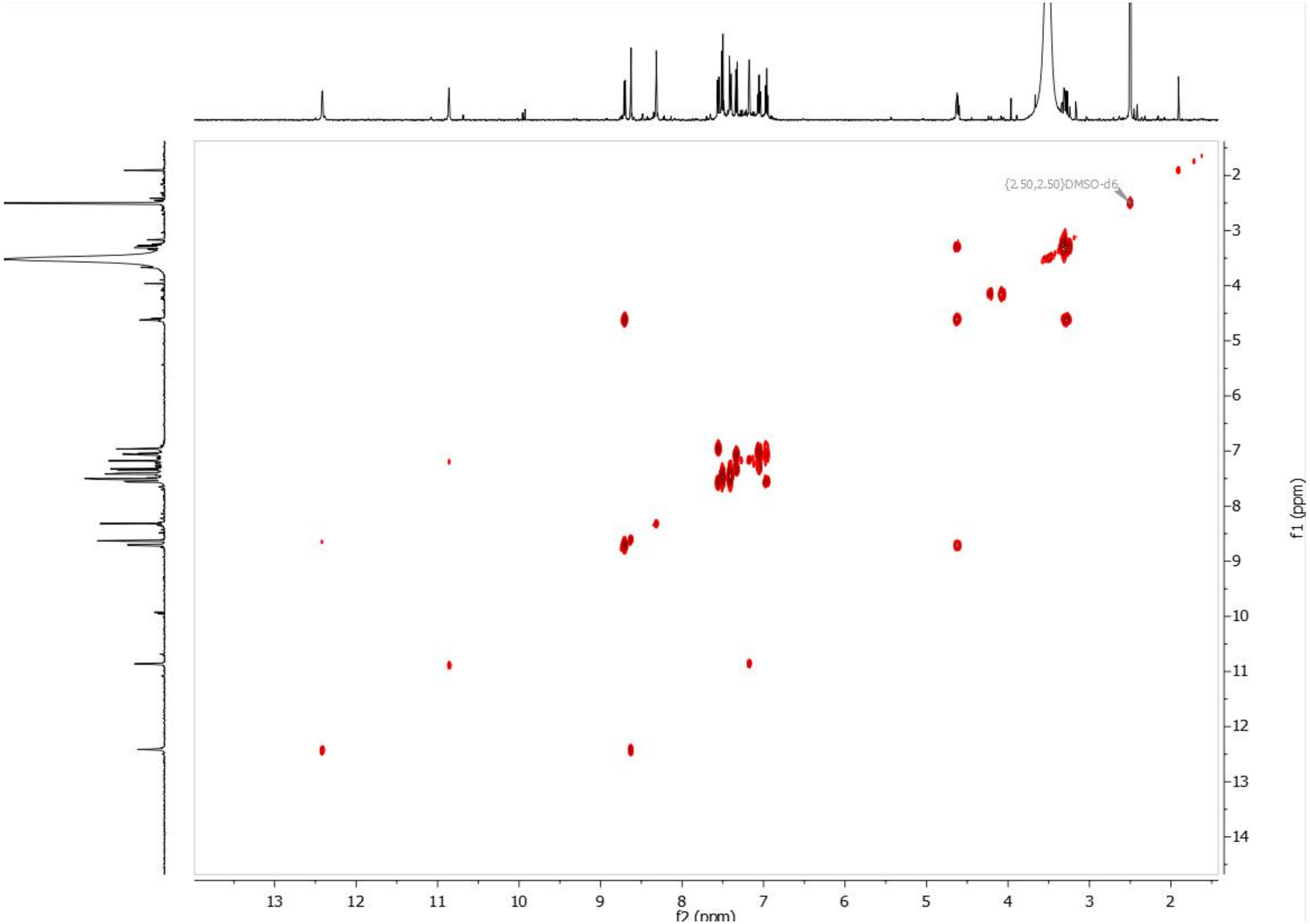

Figure S93. COSY NMR spectrum for 5-bromoindolyl-3-glyoxyl-D-tryptophan (**46**) recorded in DMSO-d_6

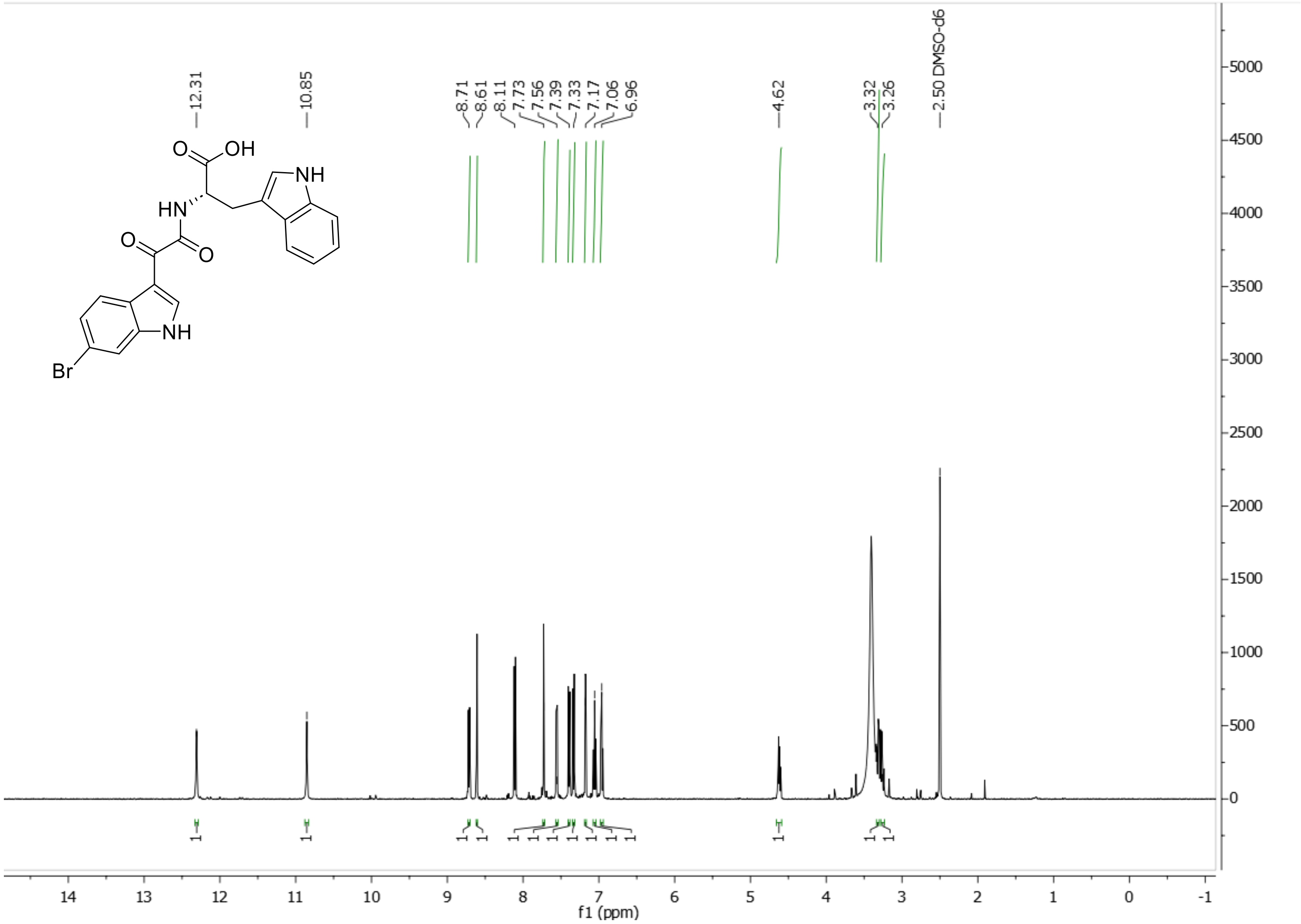

Figure S94. ^{1}H NMR spectrum for 6-bromoindolyl-3-glyoxyl-L-tryptophan (**47**) recorded in DMSO-d_6

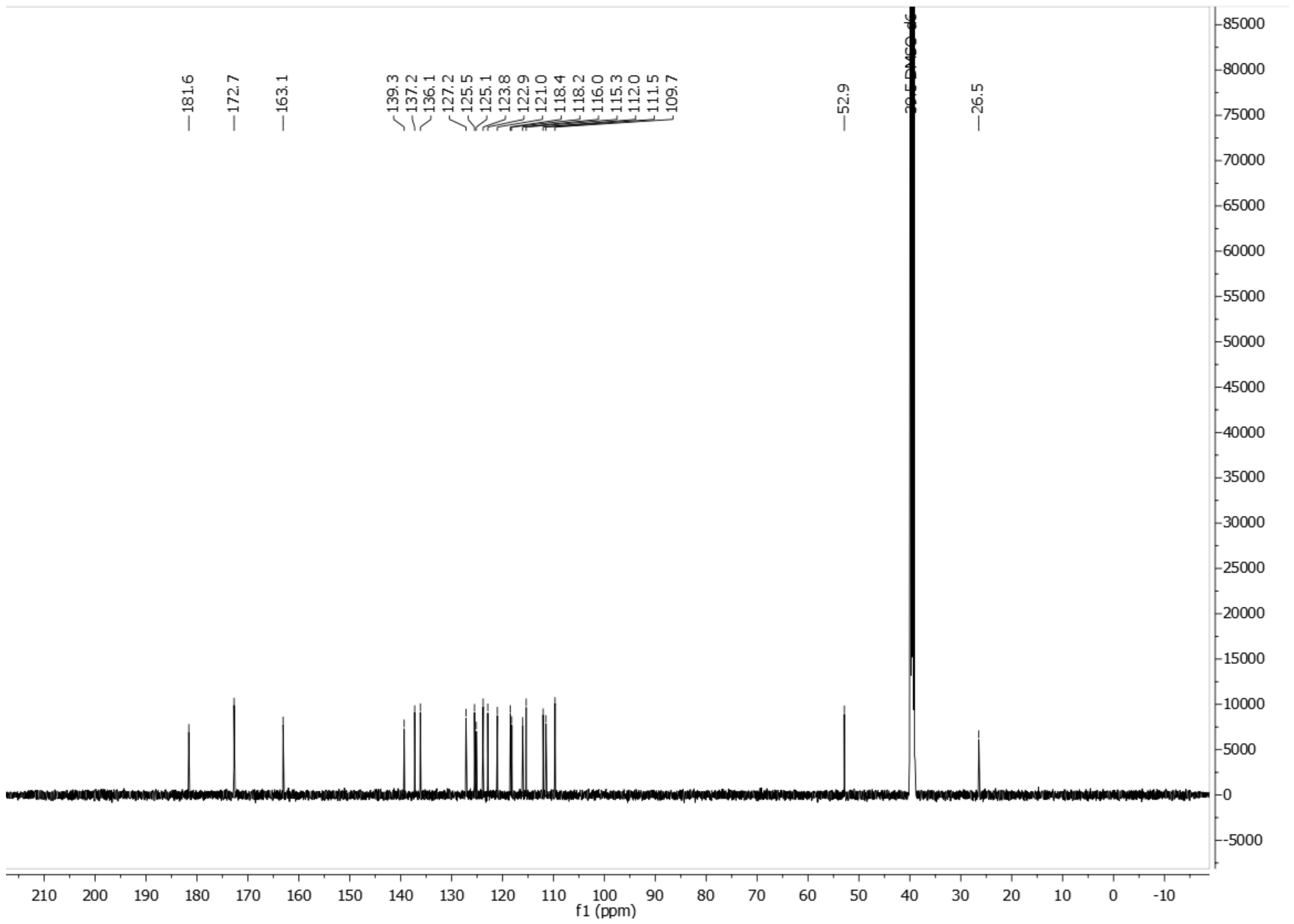

Figure S95. ^{13}C NMR spectrum for 6-bromoindolyl-3-glyoxyl-L-tryptophan (**47**) recorded in DMSO-d_6

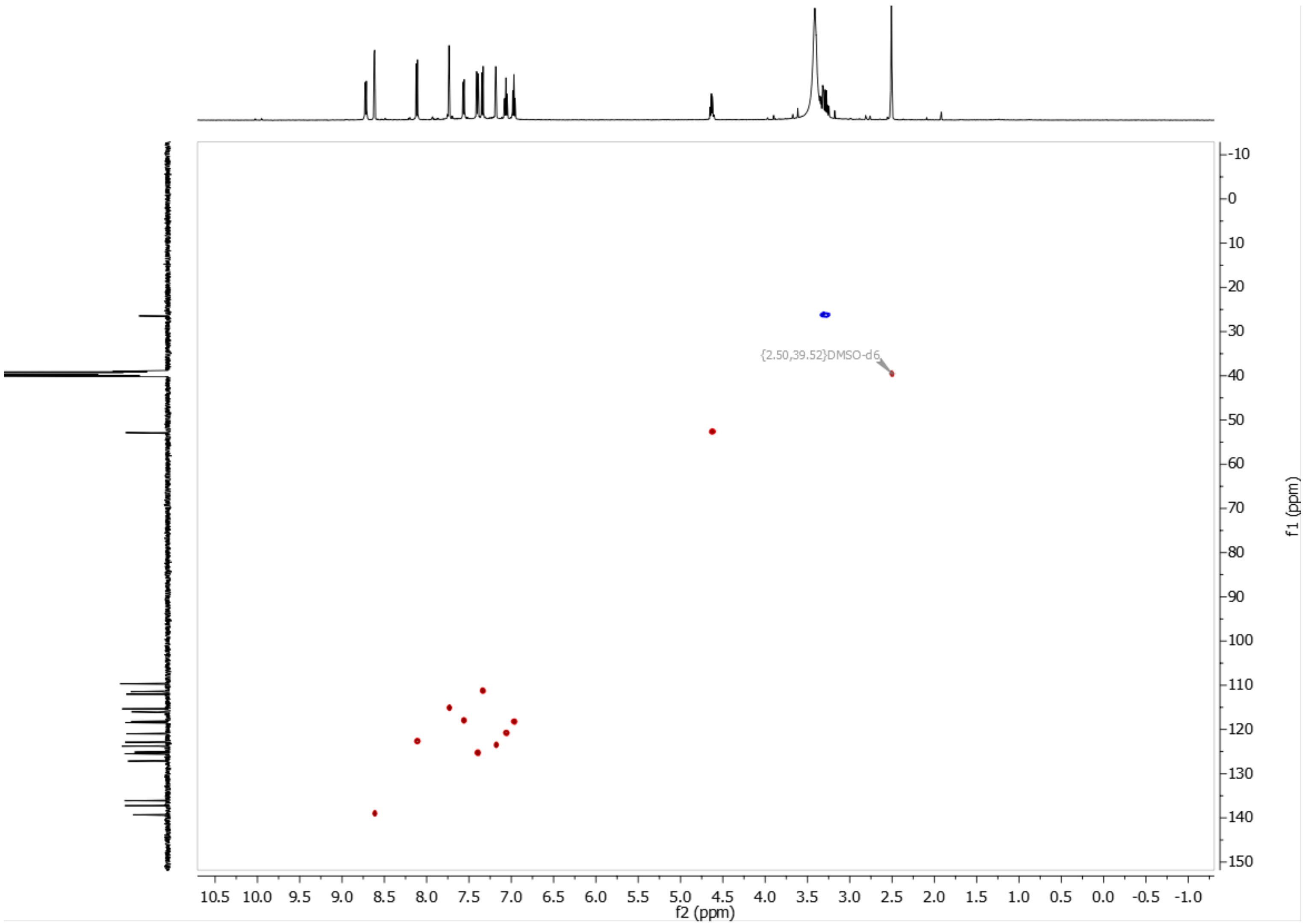

Figure S96. HSQC NMR spectrum for 6-bromoindolyl-3-glyoxyl-L-tryptophan (**47**) recorded in DMSO-*d*$_6$

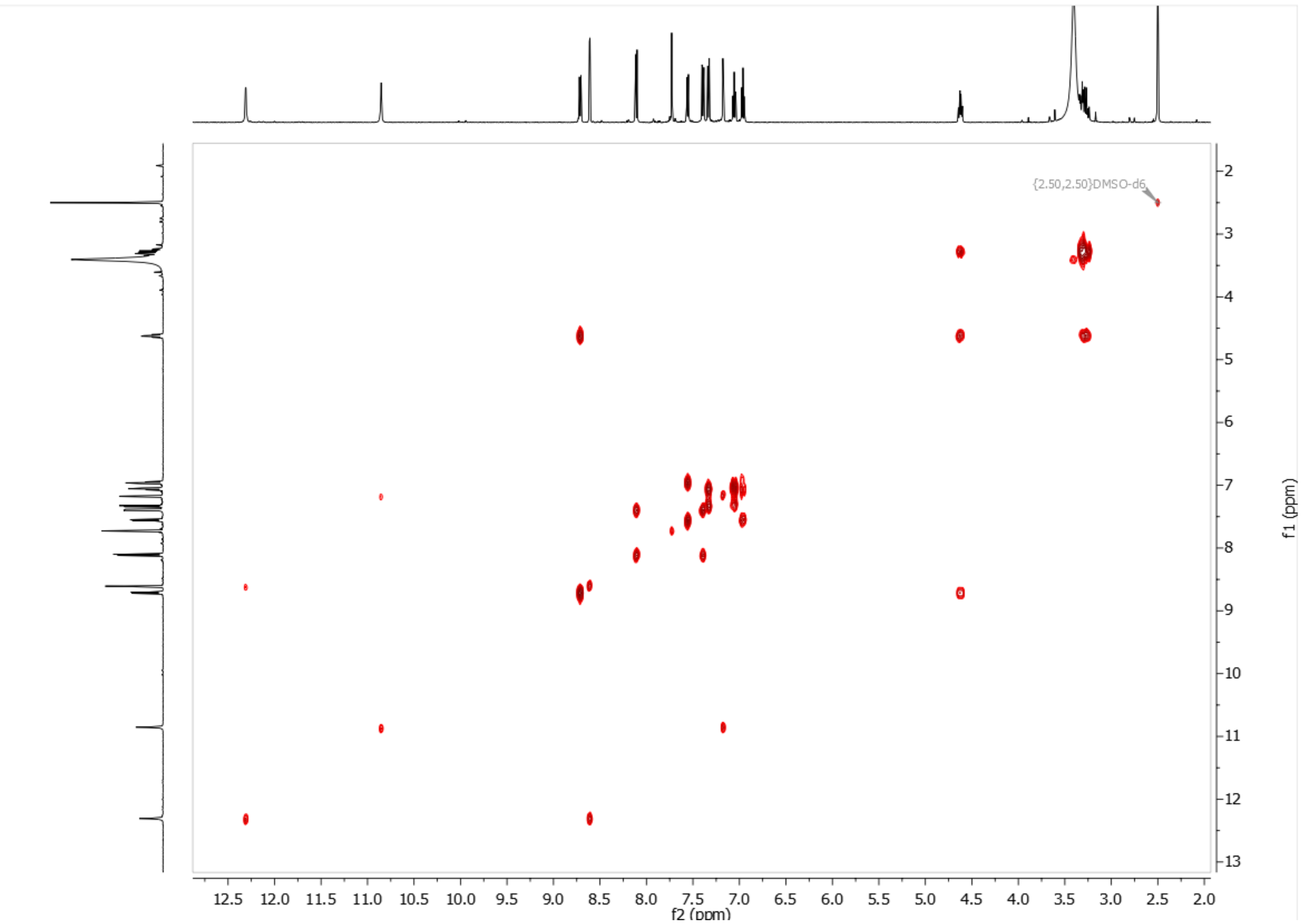

Figure S97. COSY NMR spectrum for 6-bromoindolyl-3-glyoxyl-L-tryptophan (**47**) recorded in DMSO-d_6

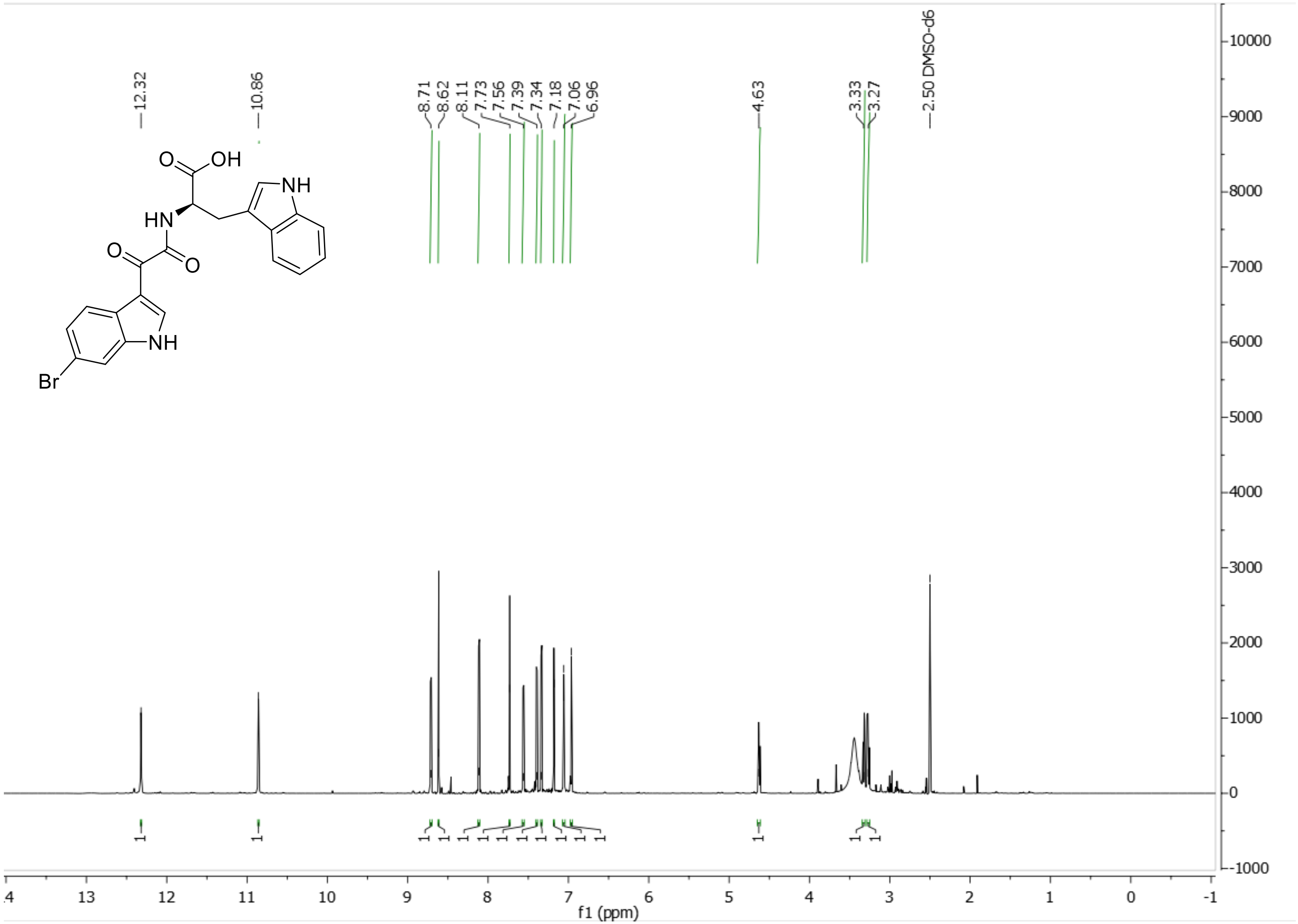

Figure S98. ^{1}H NMR spectrum for 6-bromoindolyl-3-glyoxyl-D-tryptophan (**48**) recorded in DMSO-d_6

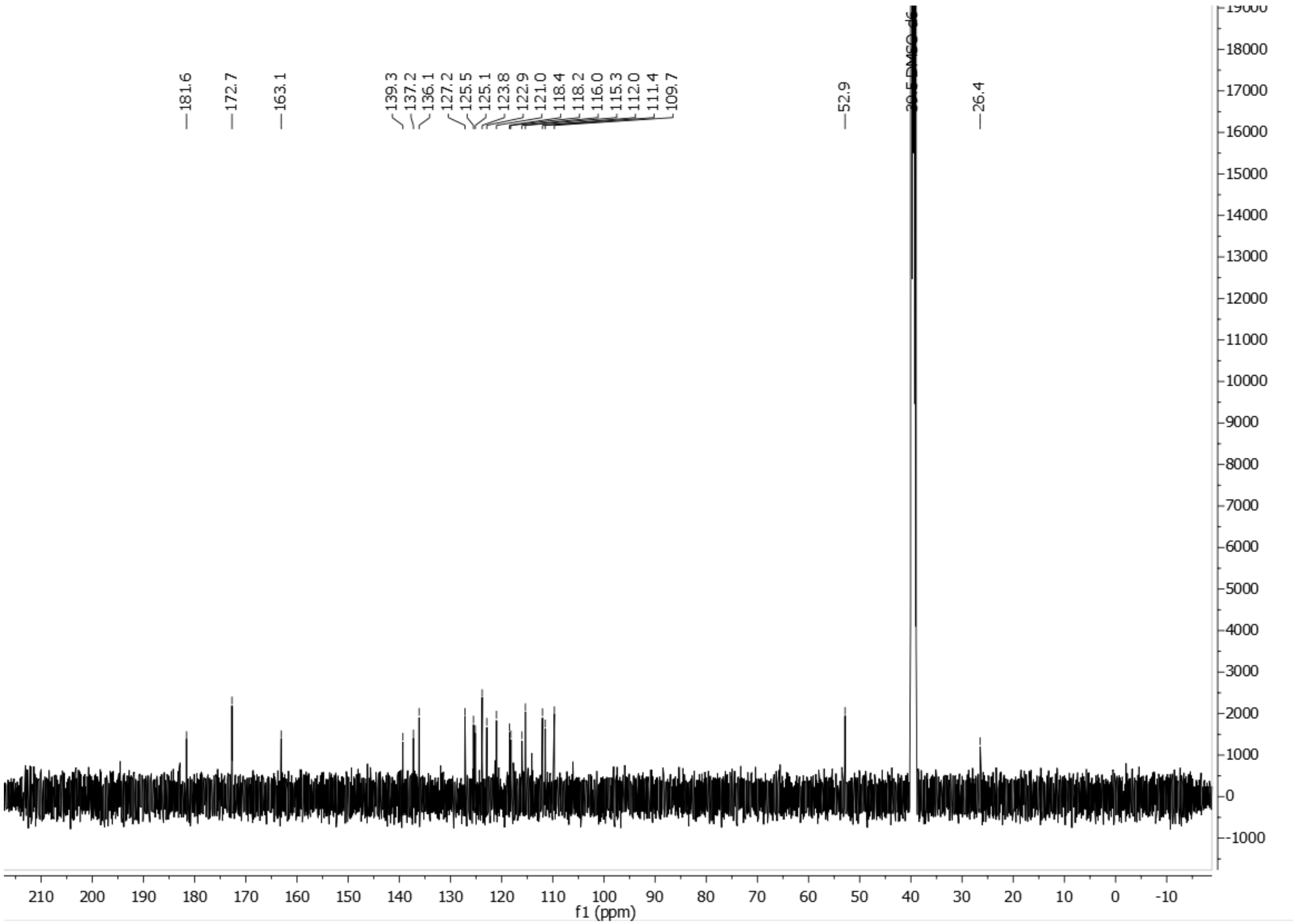

Figure S99. ^{13}C NMR spectrum for 6-bromoindolyl-3-glyoxyl-D-tryptophan (**48**) recorded in DMSO-d_6

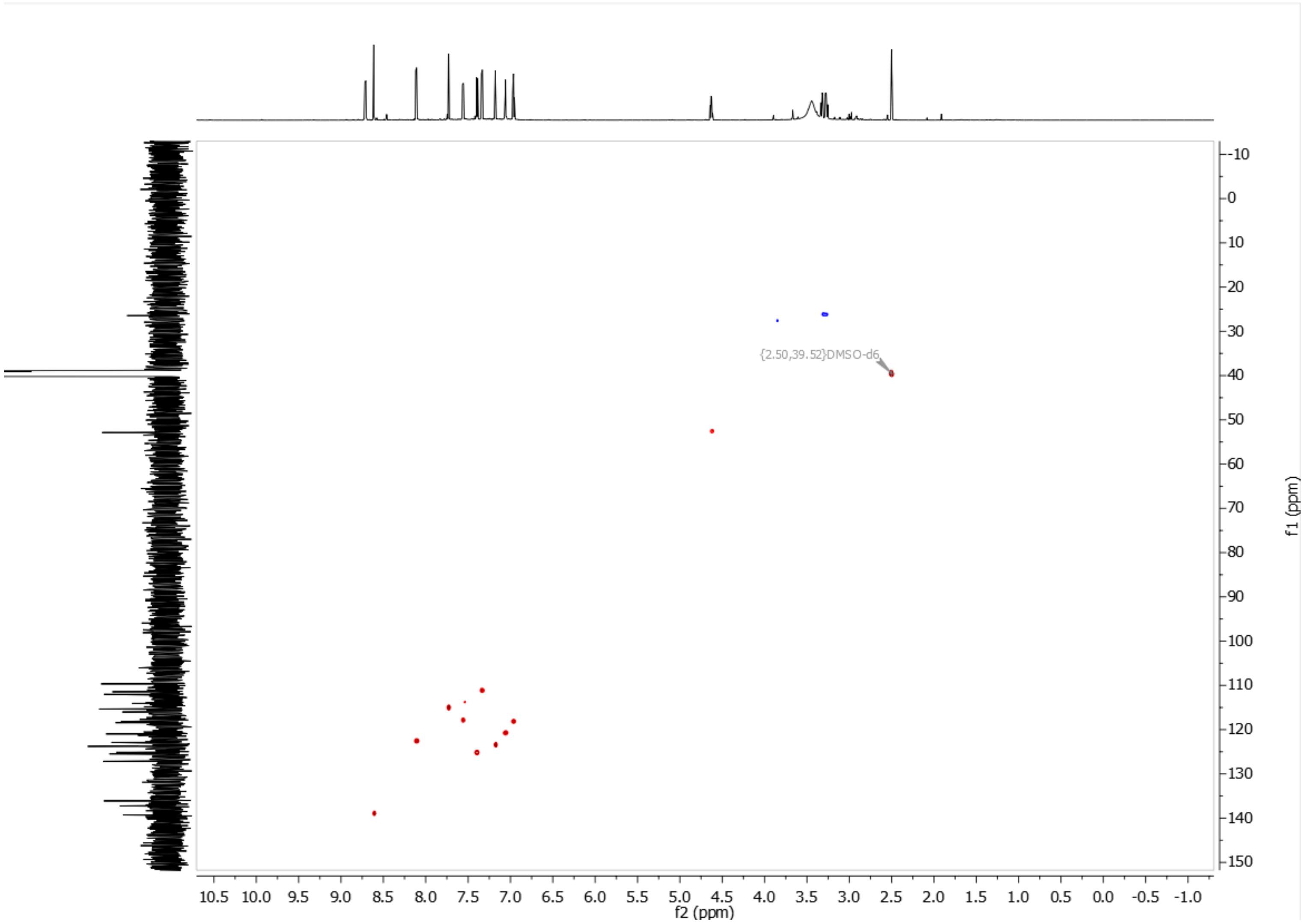

Figure S100. HSQC NMR spectrum for 6-bromoindolyl-3-glyoxyl-D-tryptophan (**48**) recorded in DMSO-d_6

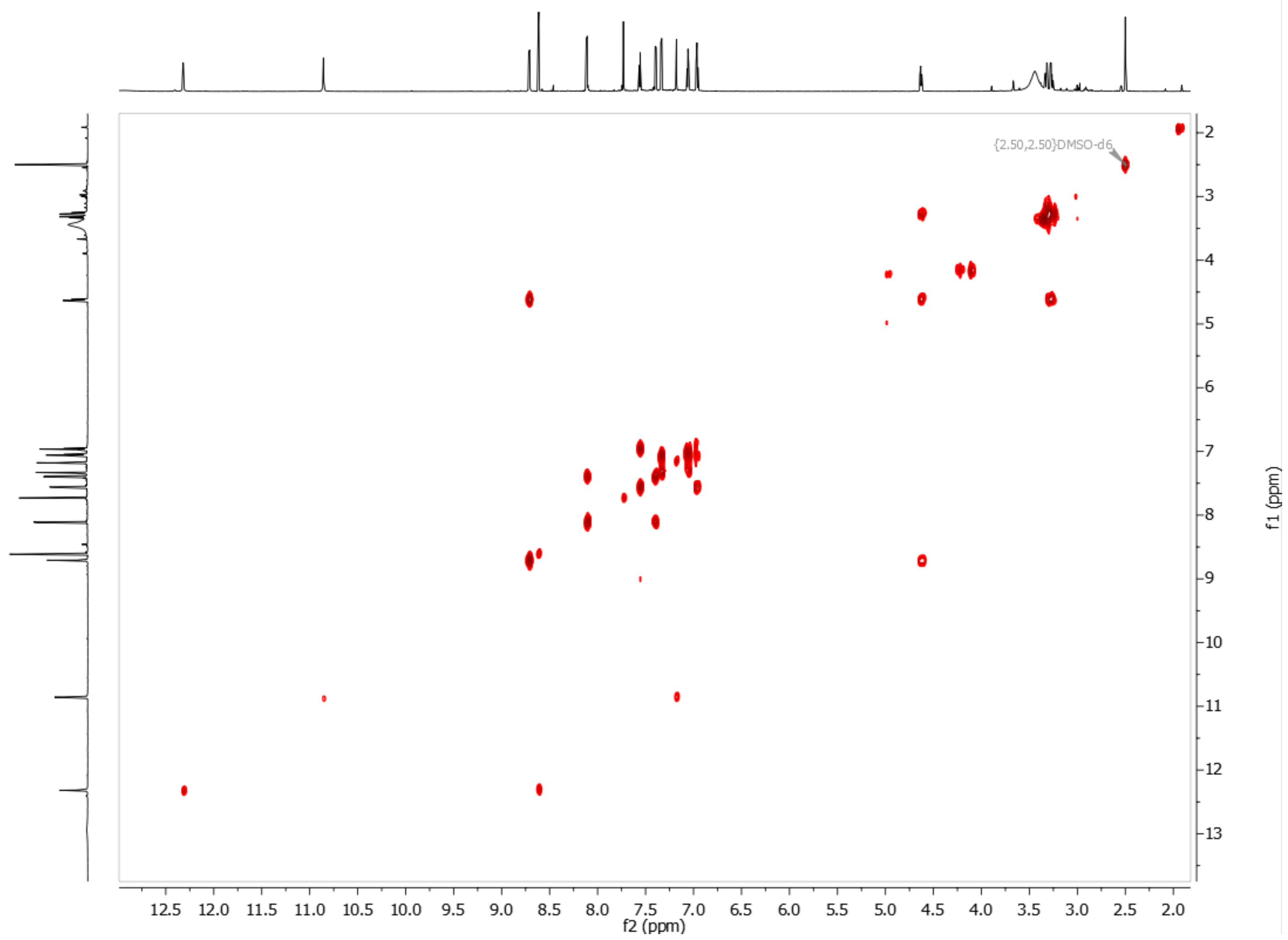

Figure S101. COSY NMR spectrum for 6-bromoindolyl-3-glyoxyl-D-tryptophan (**48**) recorded in DMSO-d_6

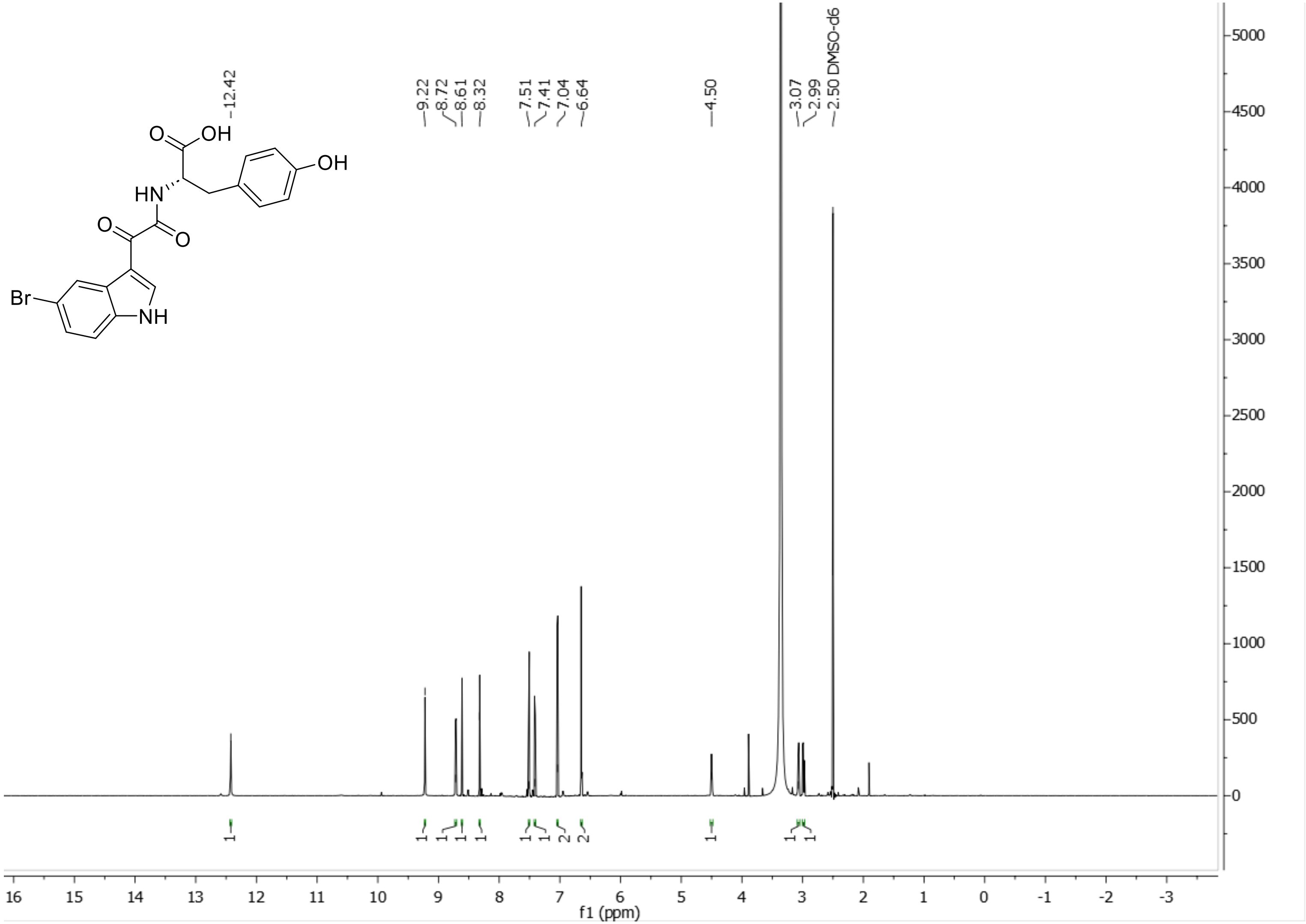

Figure S102. ^{1}H NMR spectrum for 5-bromoindolyl-3-glyoxyl-L-tyrosine (**49**) recorded in DMSO-d_6

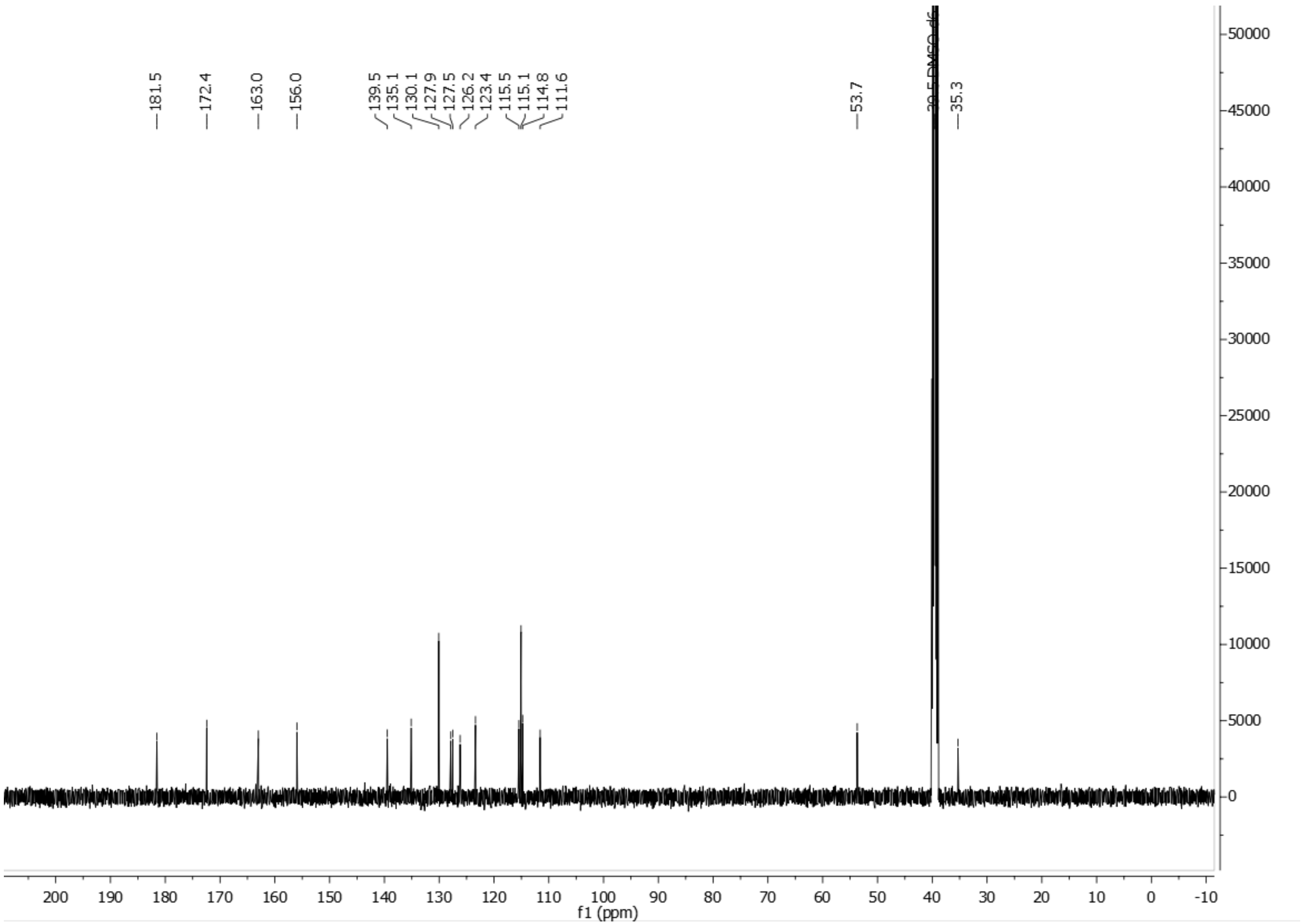

Figure S103. ^{13}C NMR spectrum for 5-bromoindolyl-3-glyoxyl-L-tyrosine (**49**) recorded in DMSO-d_6

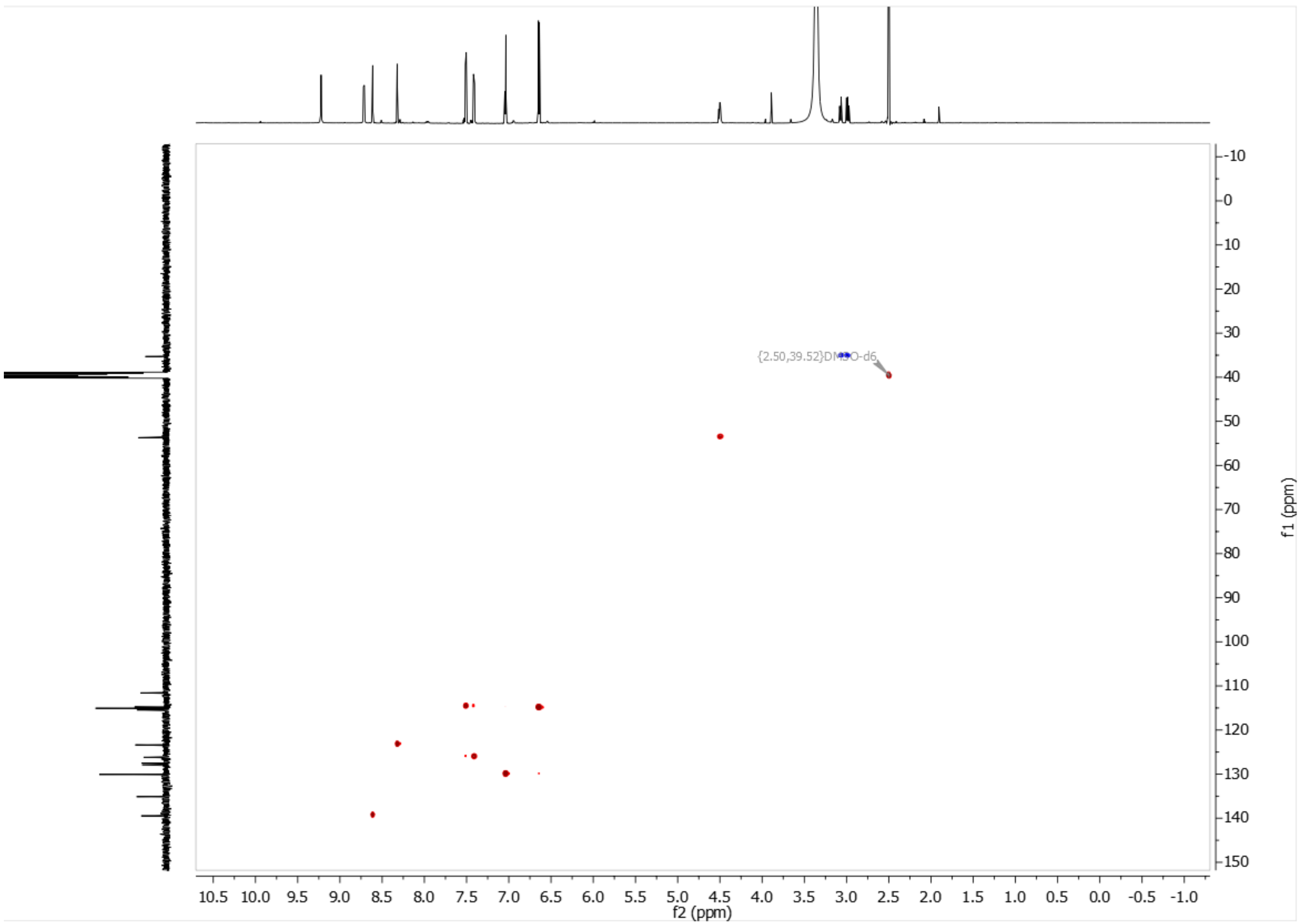

Figure S104. HSQC NMR spectrum for 5-bromoindolyl-3-glyoxyl-L-tyrosine (**49**) recorded in DMSO-d_6

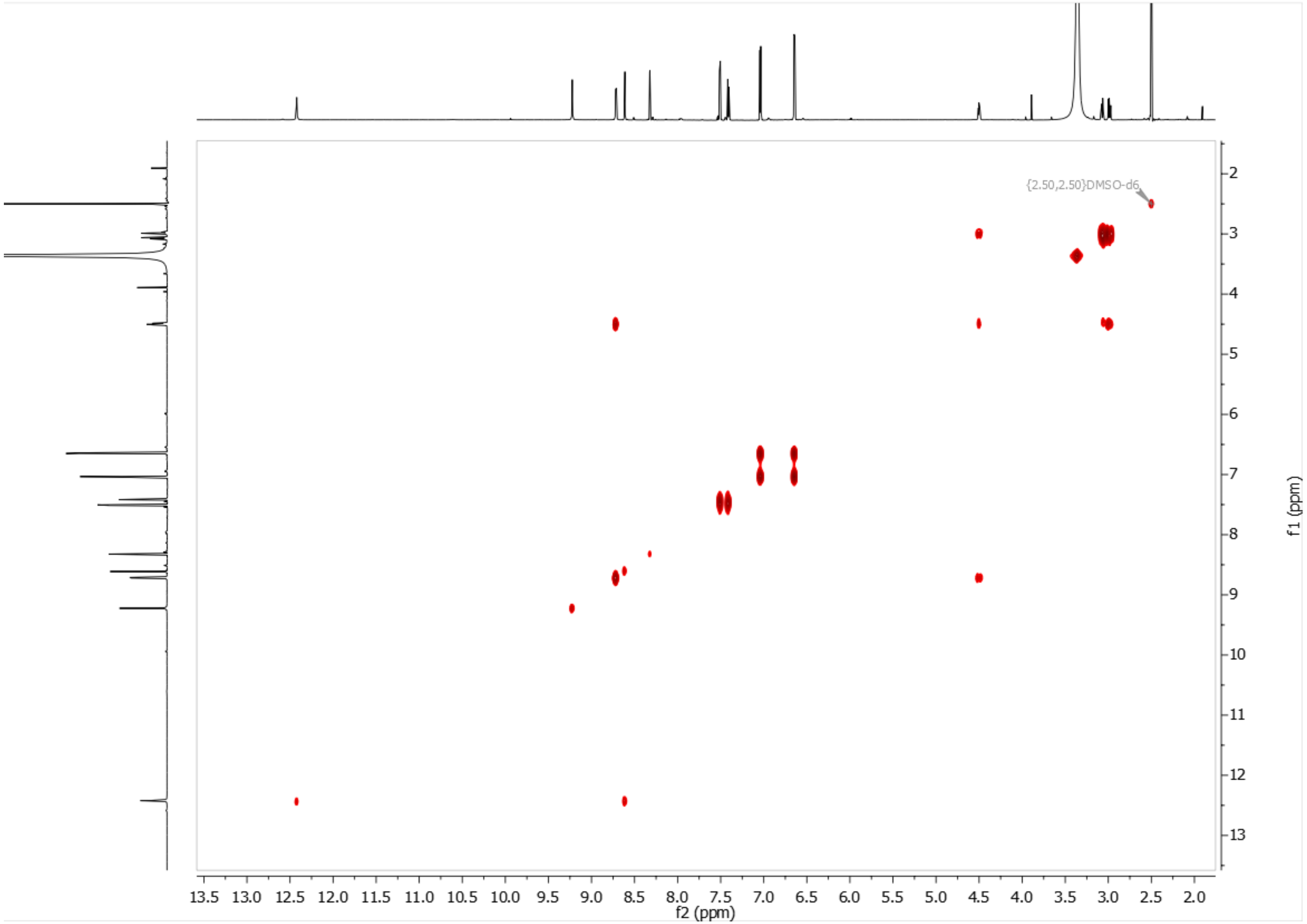

Figure S105. COSY NMR spectrum for 5-bromoindolyl-3-glyoxyl-L-tyrosine (**49**) recorded in DMSO-d_6

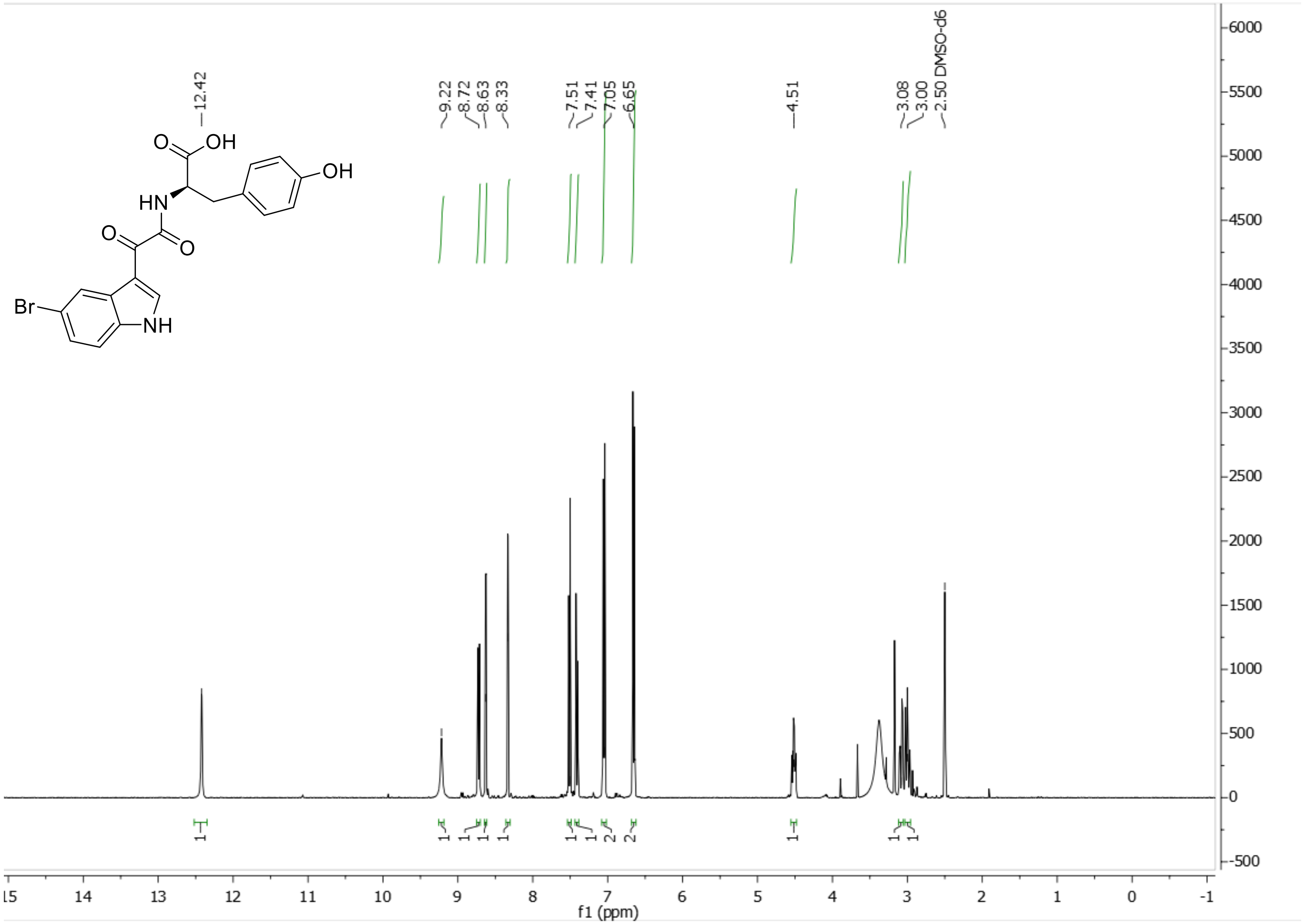

Figure S106. ^{1}H NMR spectrum for 5-bromoindolyl-3-glyoxyl-D-tyrosine (**50**) recorded in DMSO-d_6

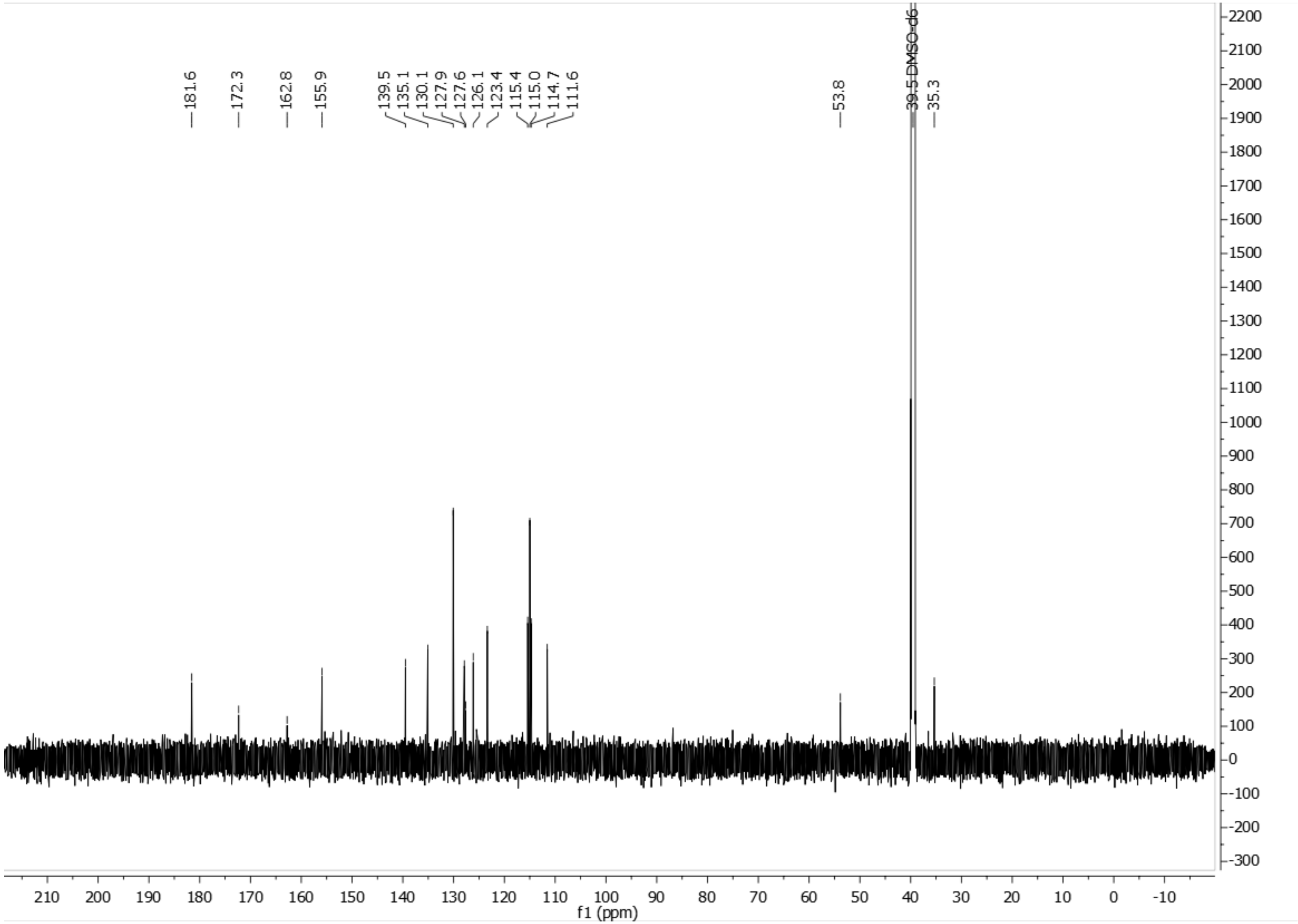

Figure S107. ^{13}C NMR spectrum for 5-bromoindolyl-3-glyoxyl-D-tyrosine (**50**) recorded in DMSO-d_6

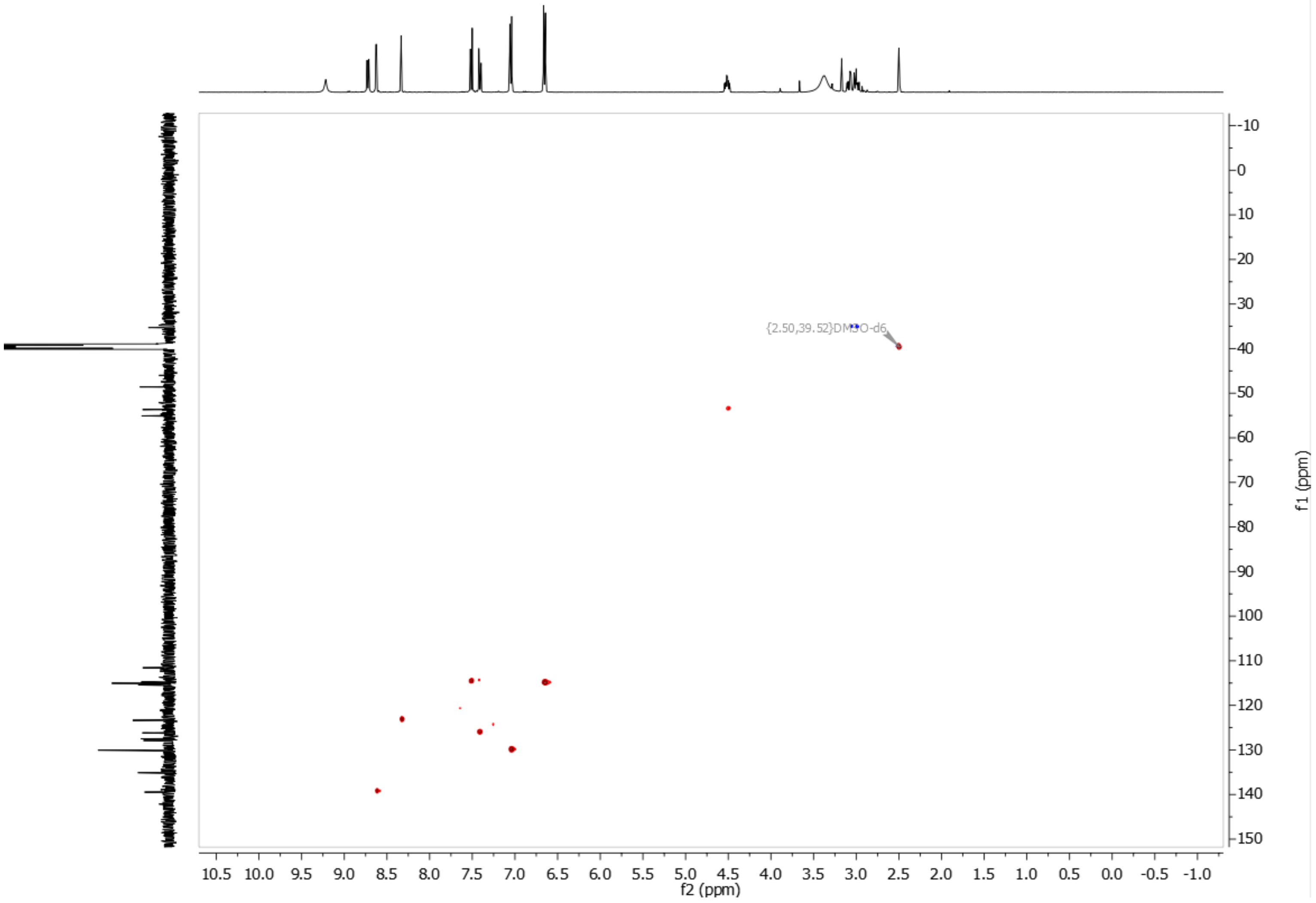

Figure S108. HSQC NMR spectrum for 5-bromoindolyl-3-glyoxyl-D-tyrosine (**50**) recorded in DMSO-*d*6

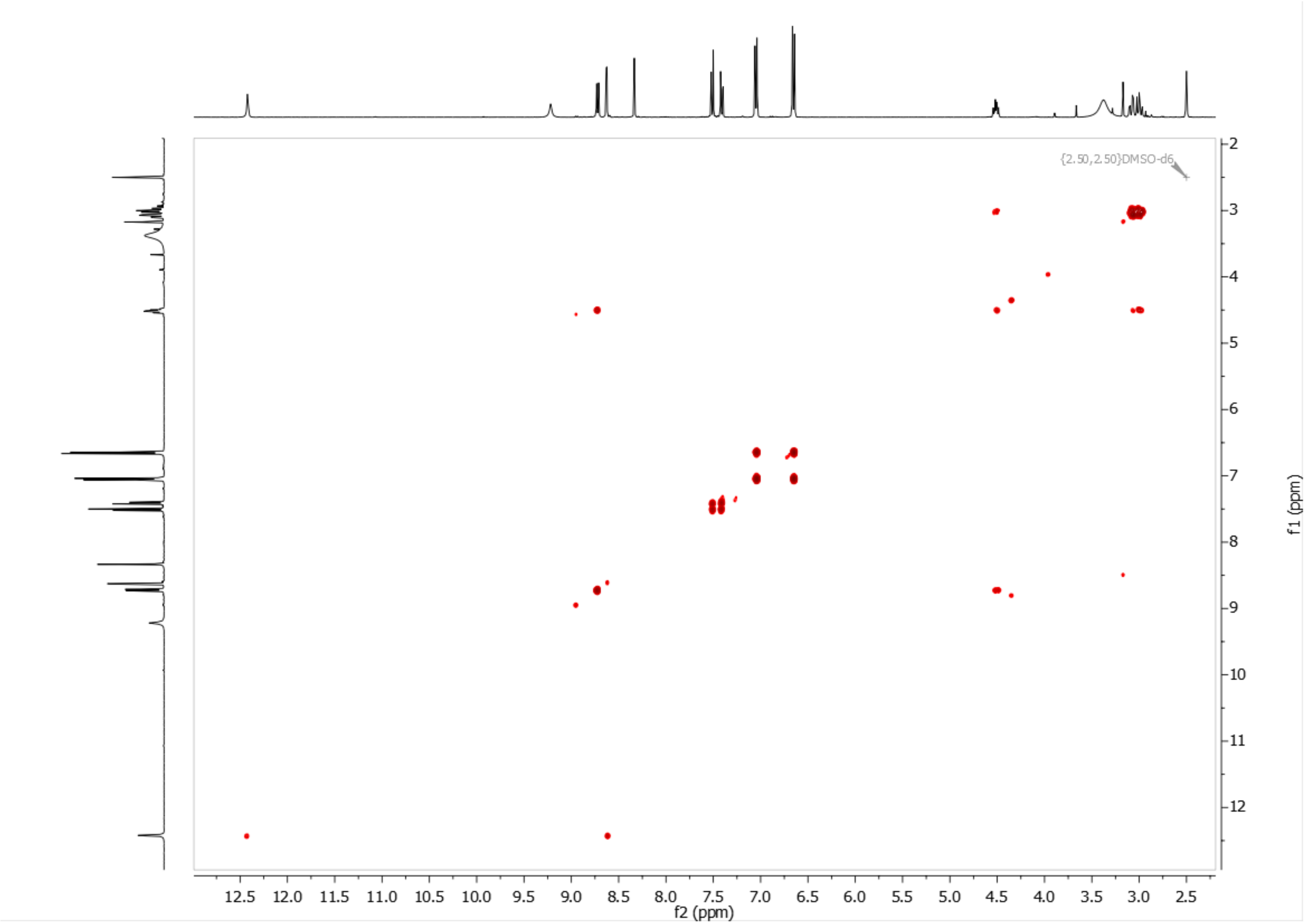

Figure S109. COSY NMR spectrum for 5-bromoindolyl-3-glyoxyl-D-tyrosine (**50**) recorded in DMSO-d_6

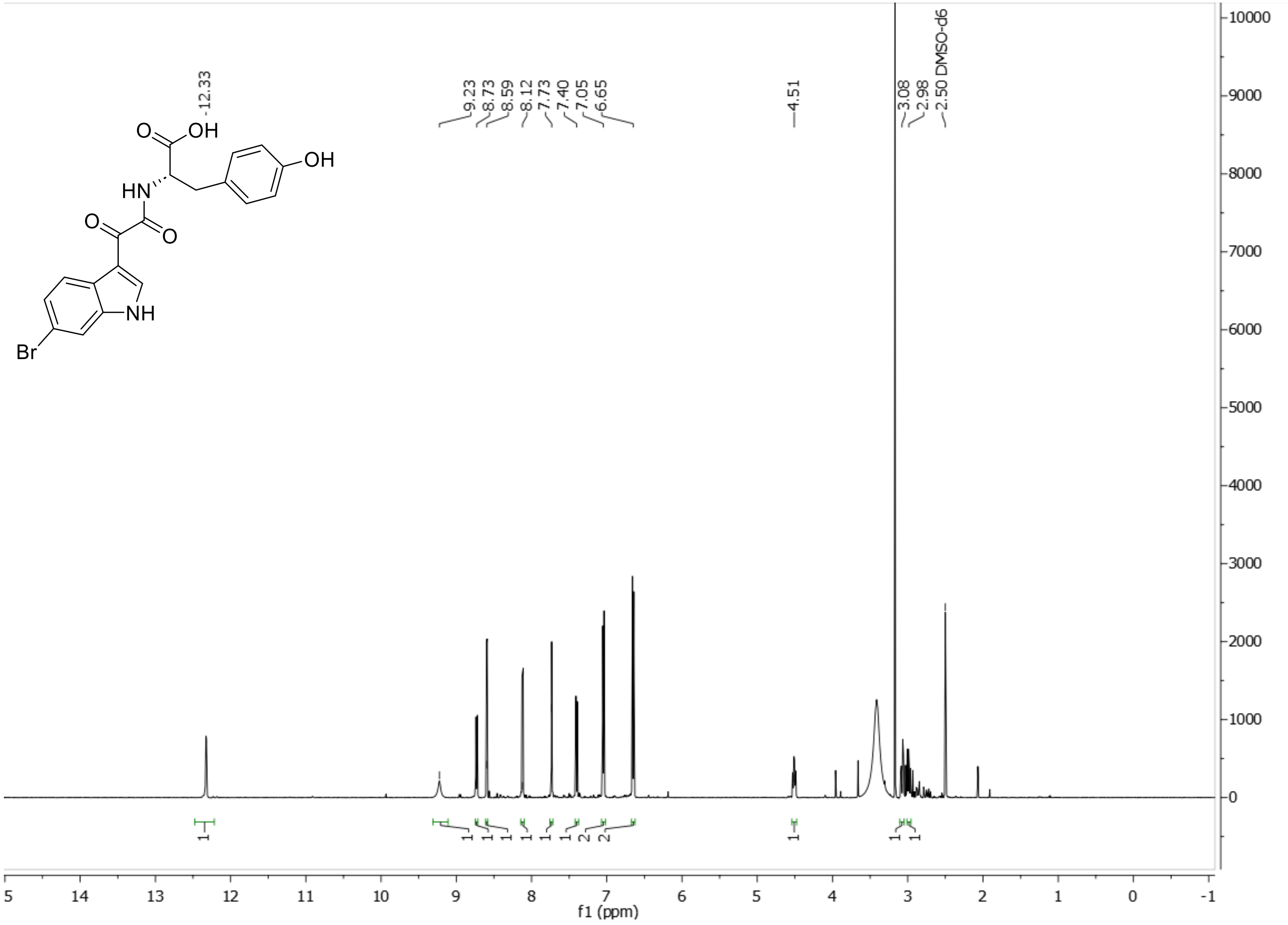

Figure S110. ^{1}H NMR spectrum for 6-bromoindolyl-3-glyoxyl-L-tyrosine (**51**) recorded in DMSO-d_6

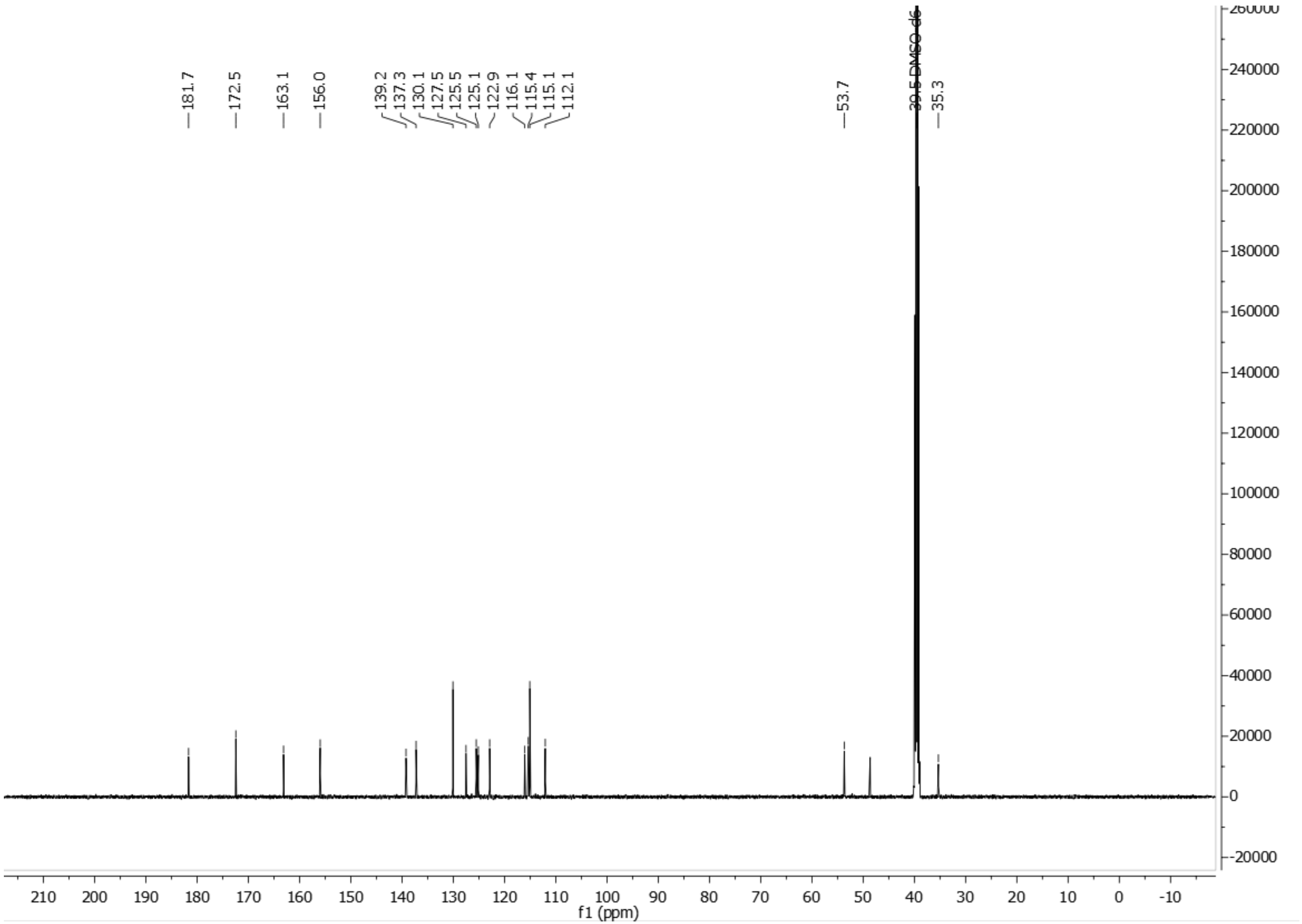

Figure S111. ^{13}C NMR spectrum for 6-bromoindolyl-3-glyoxyl-L-tyrosine (**51**) recorded in DMSO-d_6

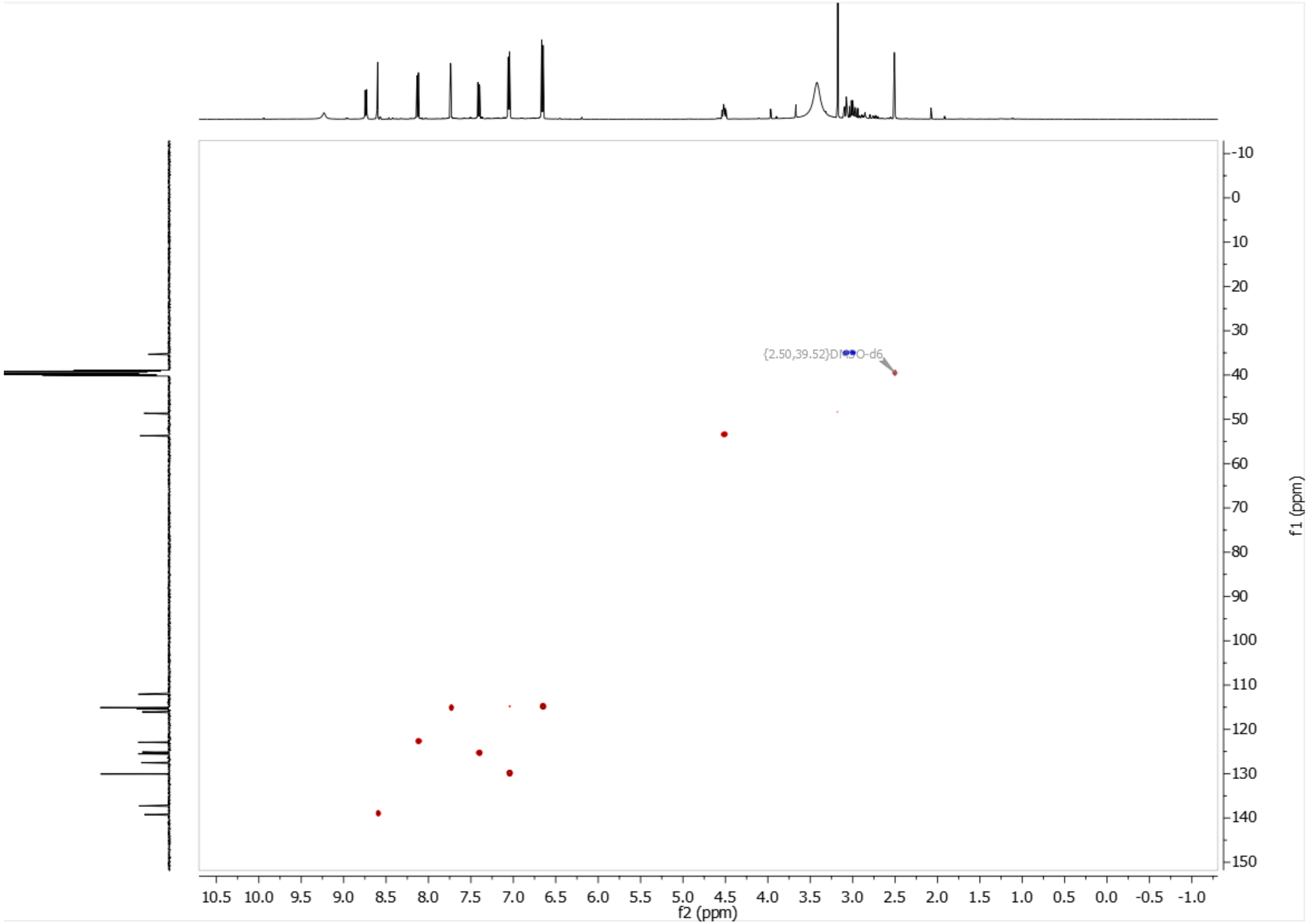

Figure S112. HSQC NMR spectrum for 6-bromoindolyl-3-glyoxyl-L-tyrosine (**51**) recorded in DMSO-d_6

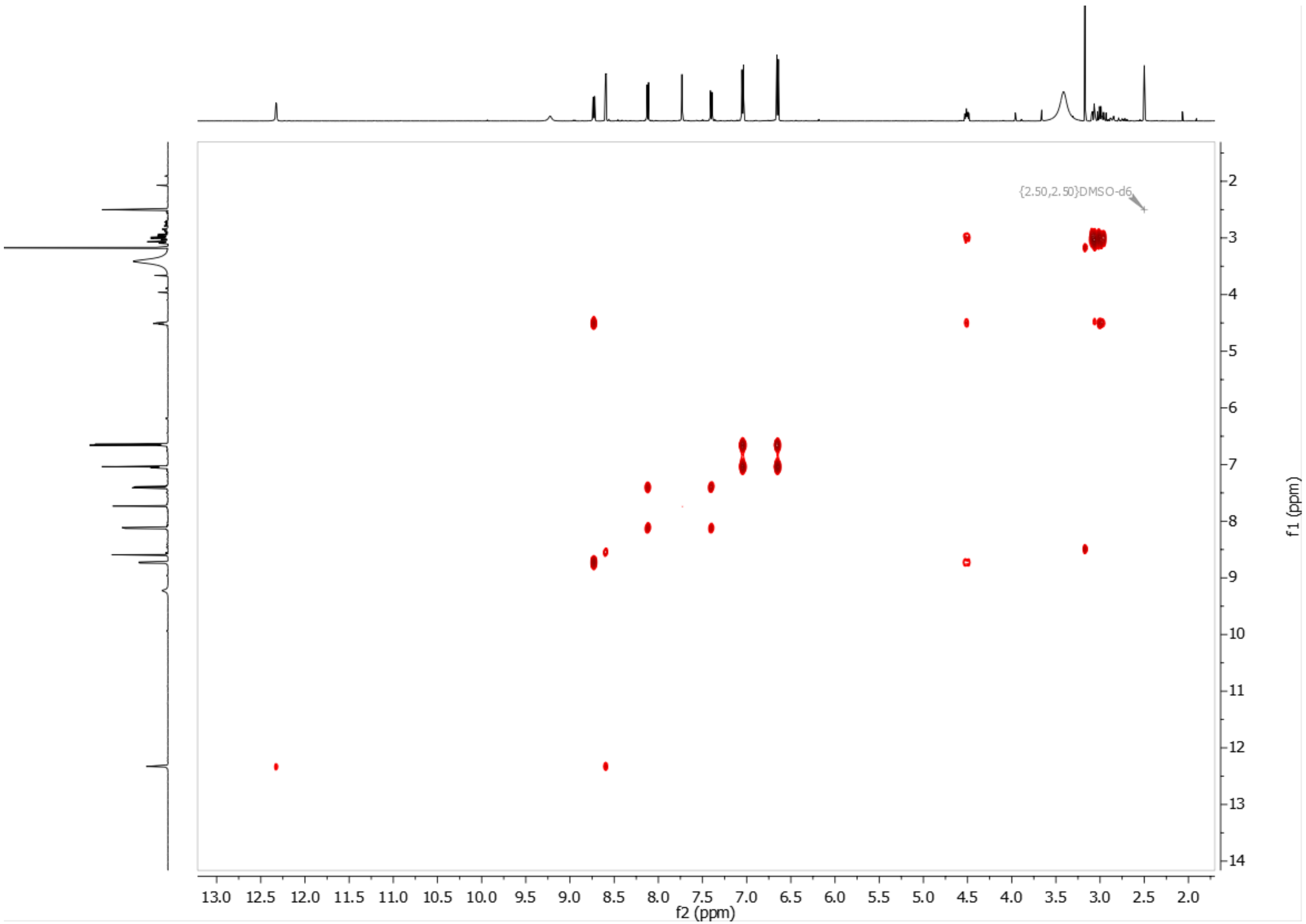

Figure S113. COSY NMR spectrum for 6-bromoindolyl-3-glyoxyl-L-tyrosine (**51**) recorded in DMSO-d_6

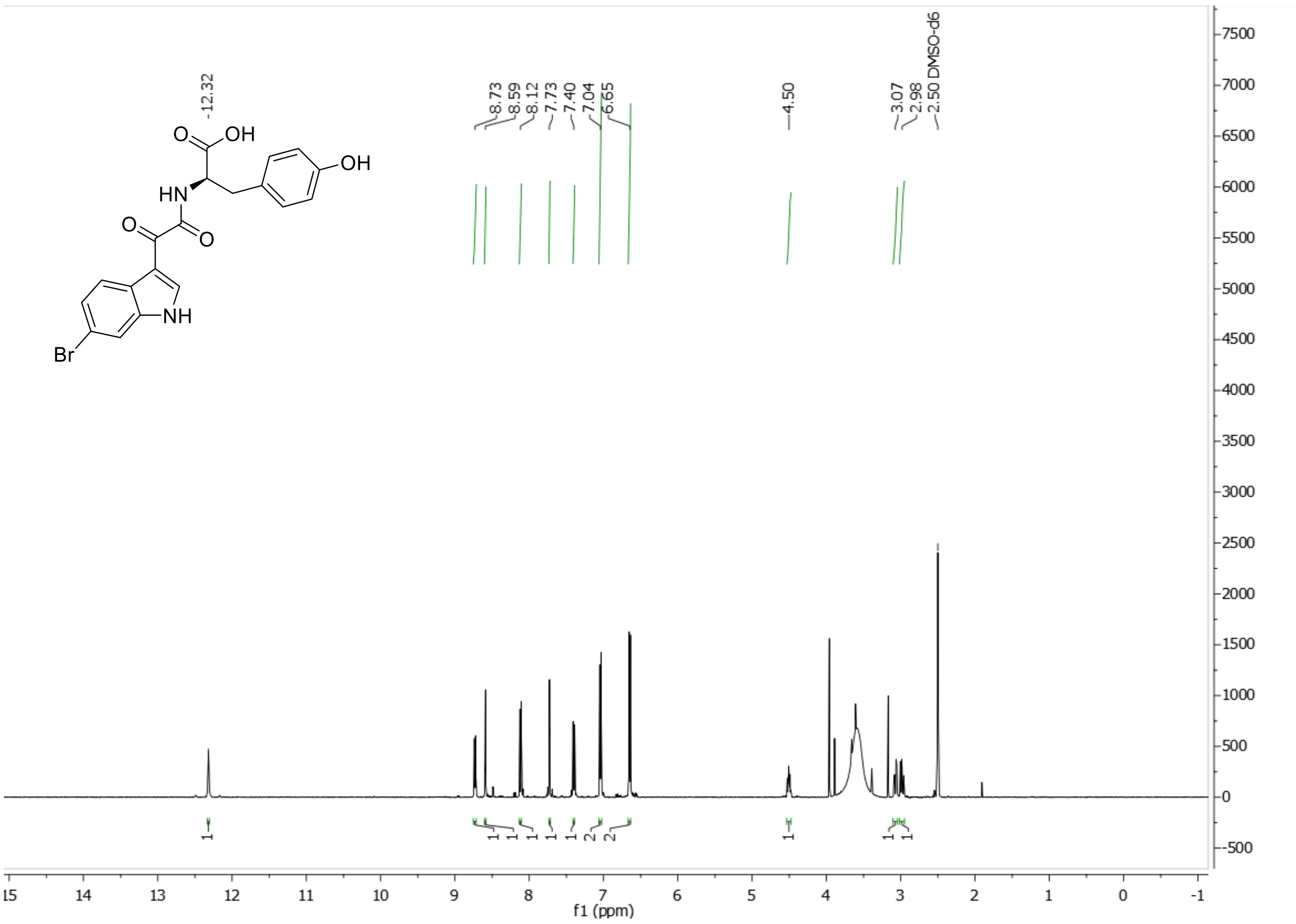

Figure S114. ^{1}H NMR spectrum for 6-bromoindolyl-3-glyoxyl-D-tyrosine (**52**) recorded in DMSO-d_6

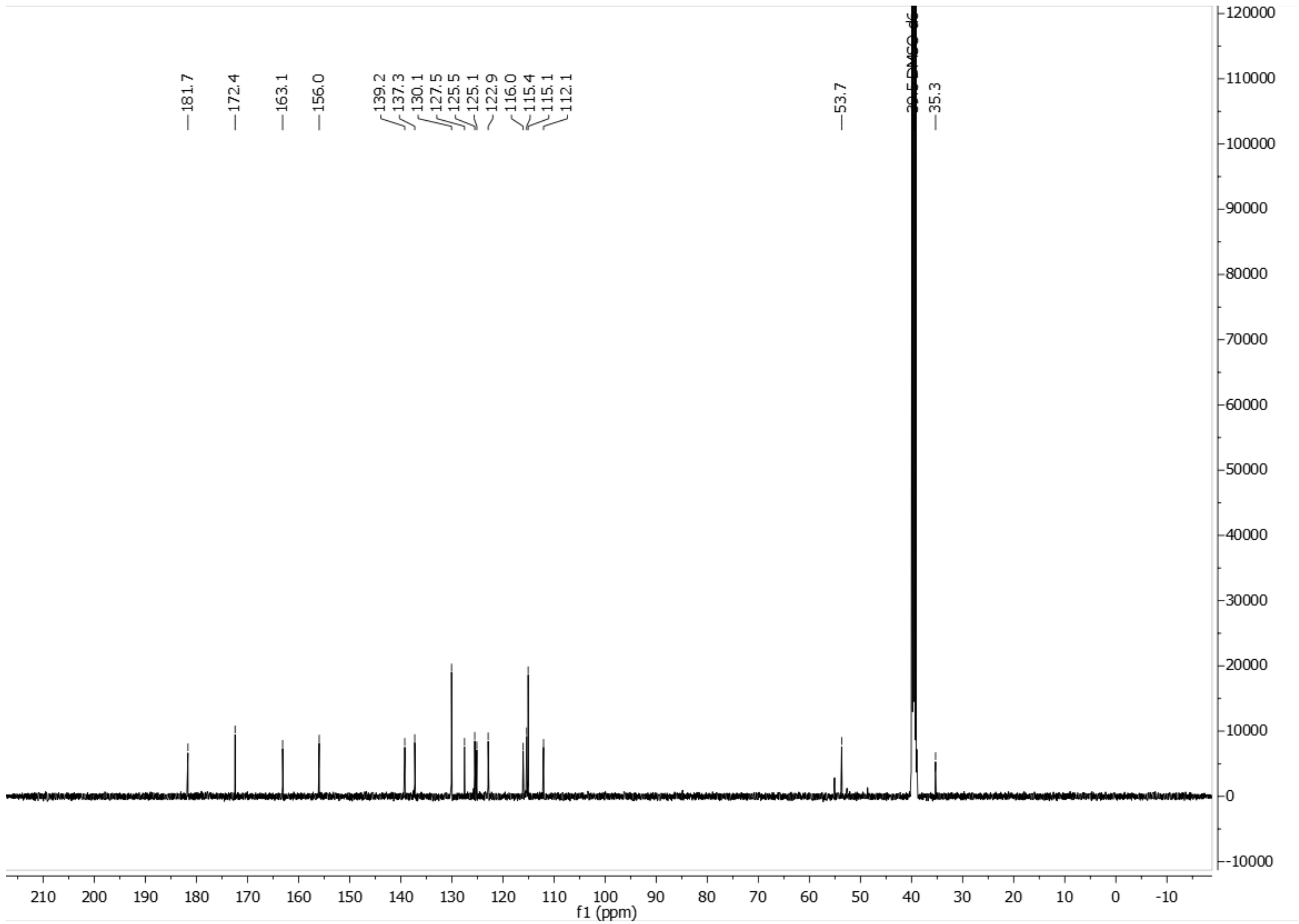

Figure S115. ^{13}C NMR spectrum for 6-bromoindolyl-3-glyoxyl-D-tyrosine (**52**) recorded in DMSO-d_6

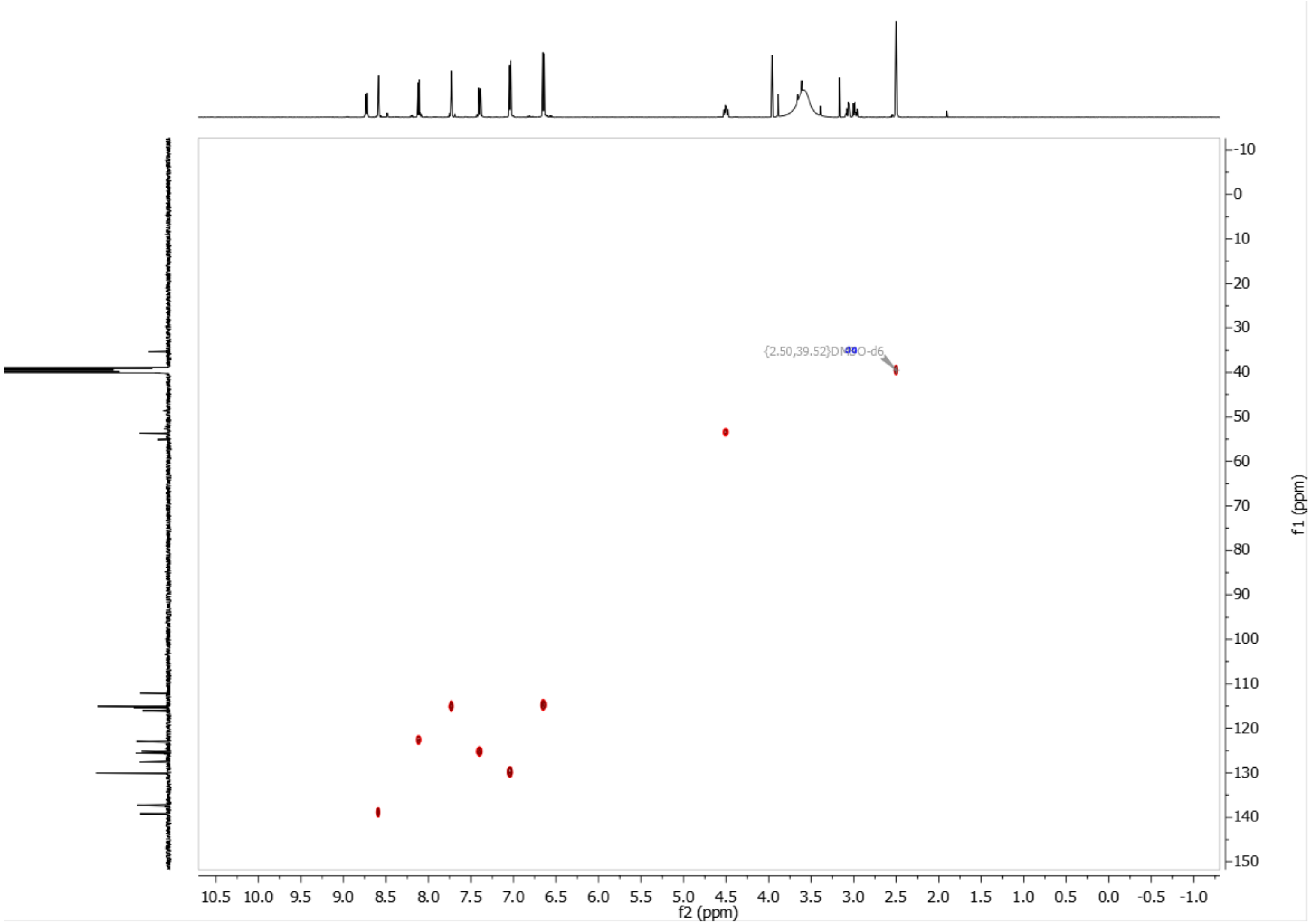

Figure S116. HSQC NMR spectrum for 6-bromoindolyl-3-glyoxyl-D-tyrosine (**52**) recorded in DMSO-d_6

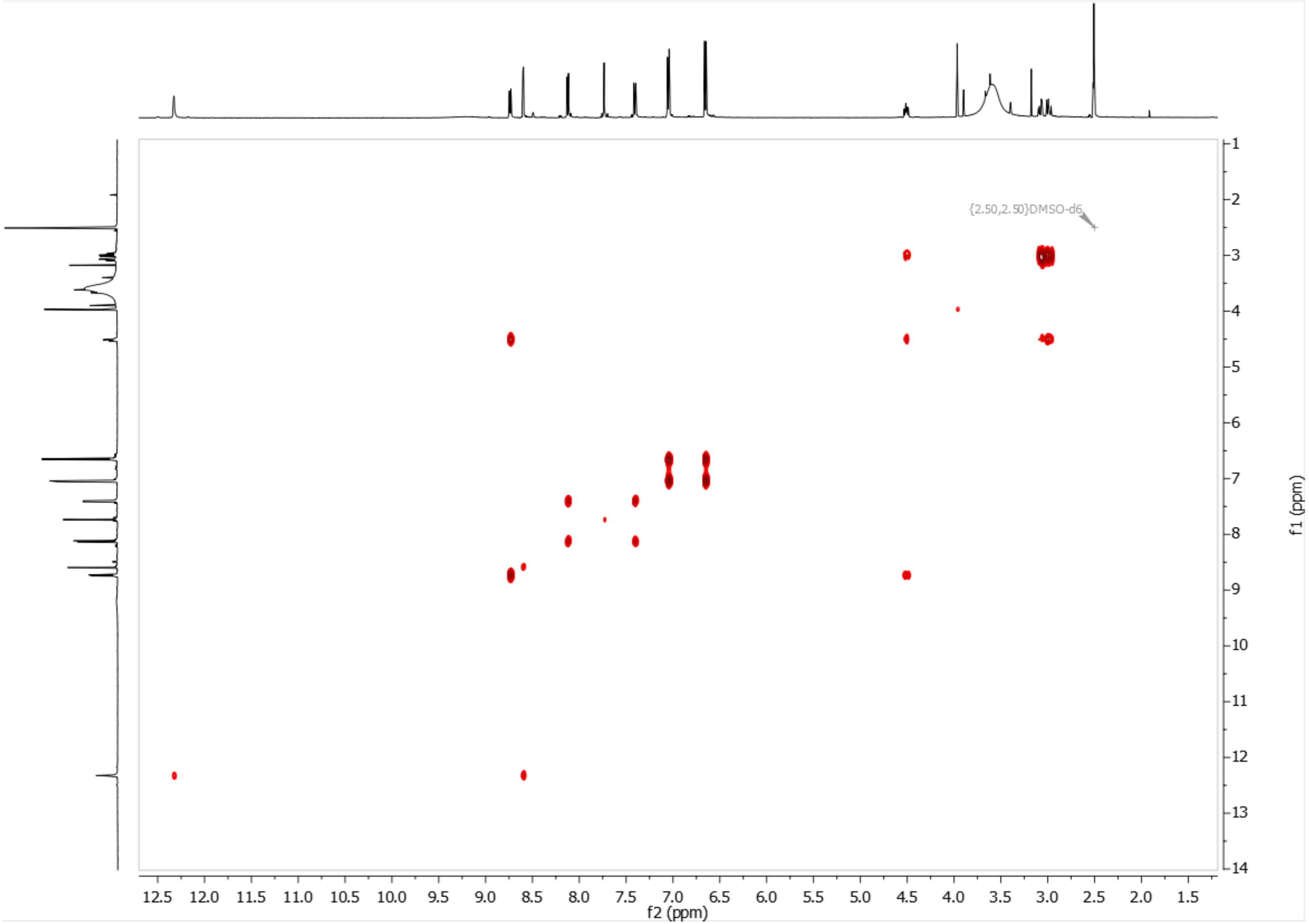

Figure S117. COSY NMR spectrum for 6-bromoindolyl-3-glyoxyl-D-tyrosine (**52**) recorded in DMSO-d_6

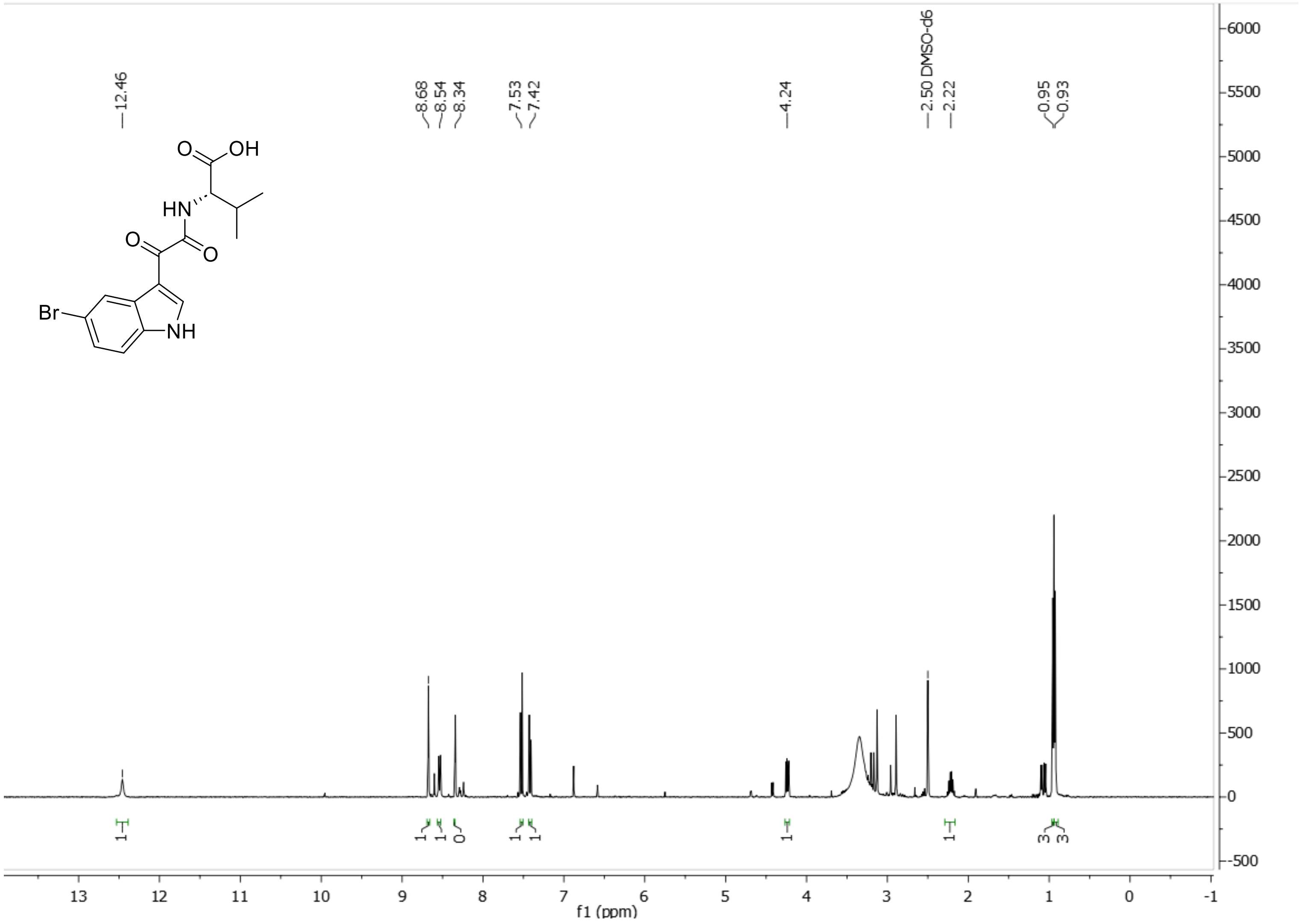

Figure S118. ^{1}H NMR spectrum for 5-bromoindolyl-3-glyoxyl-L-valine (**53**) recorded in DMSO-d_6

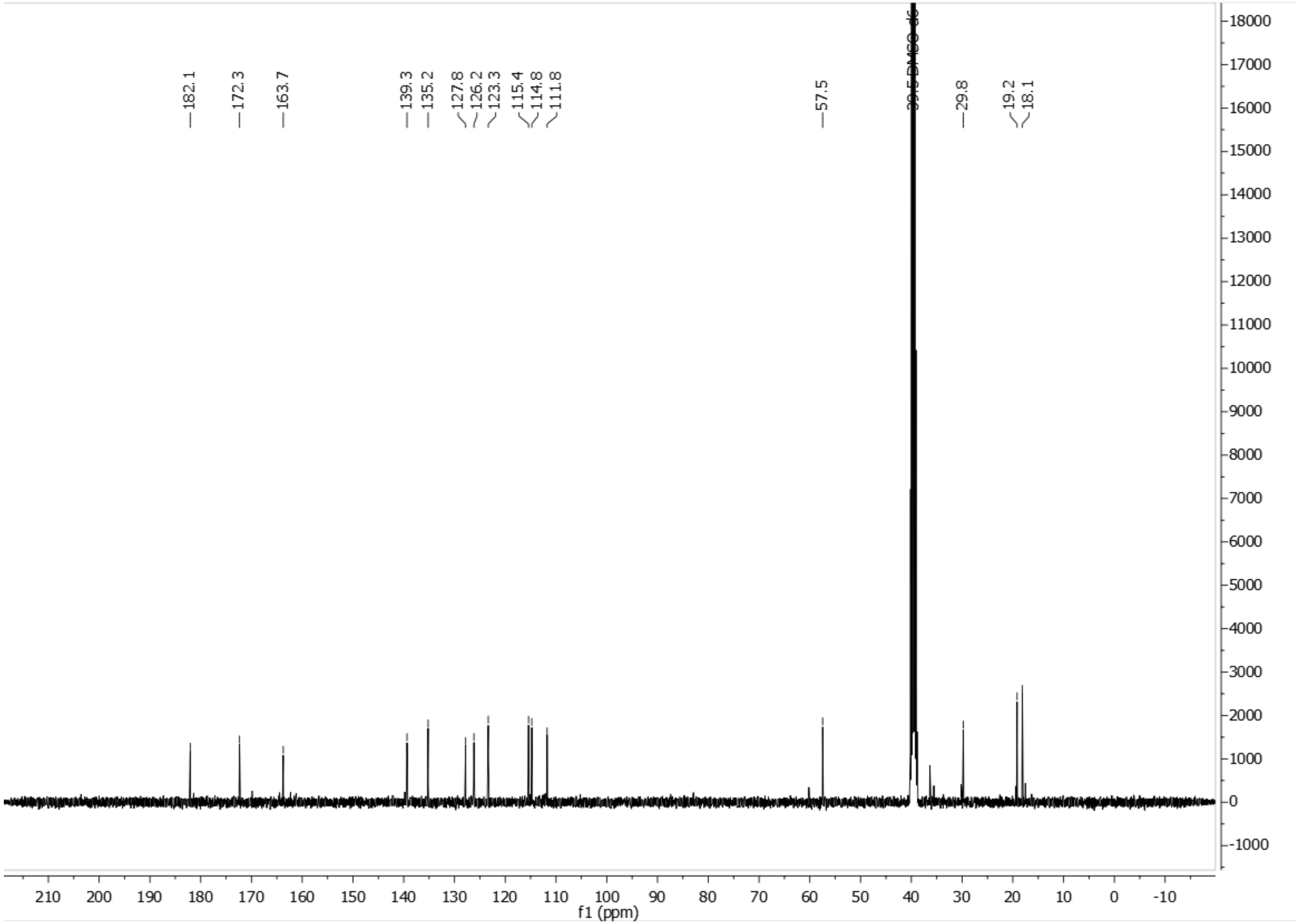

Figure S119. ^{13}C NMR spectrum for 5-bromoindolyl-3-glyoxyl-L-valine (**53**) recorded in DMSO-d_6

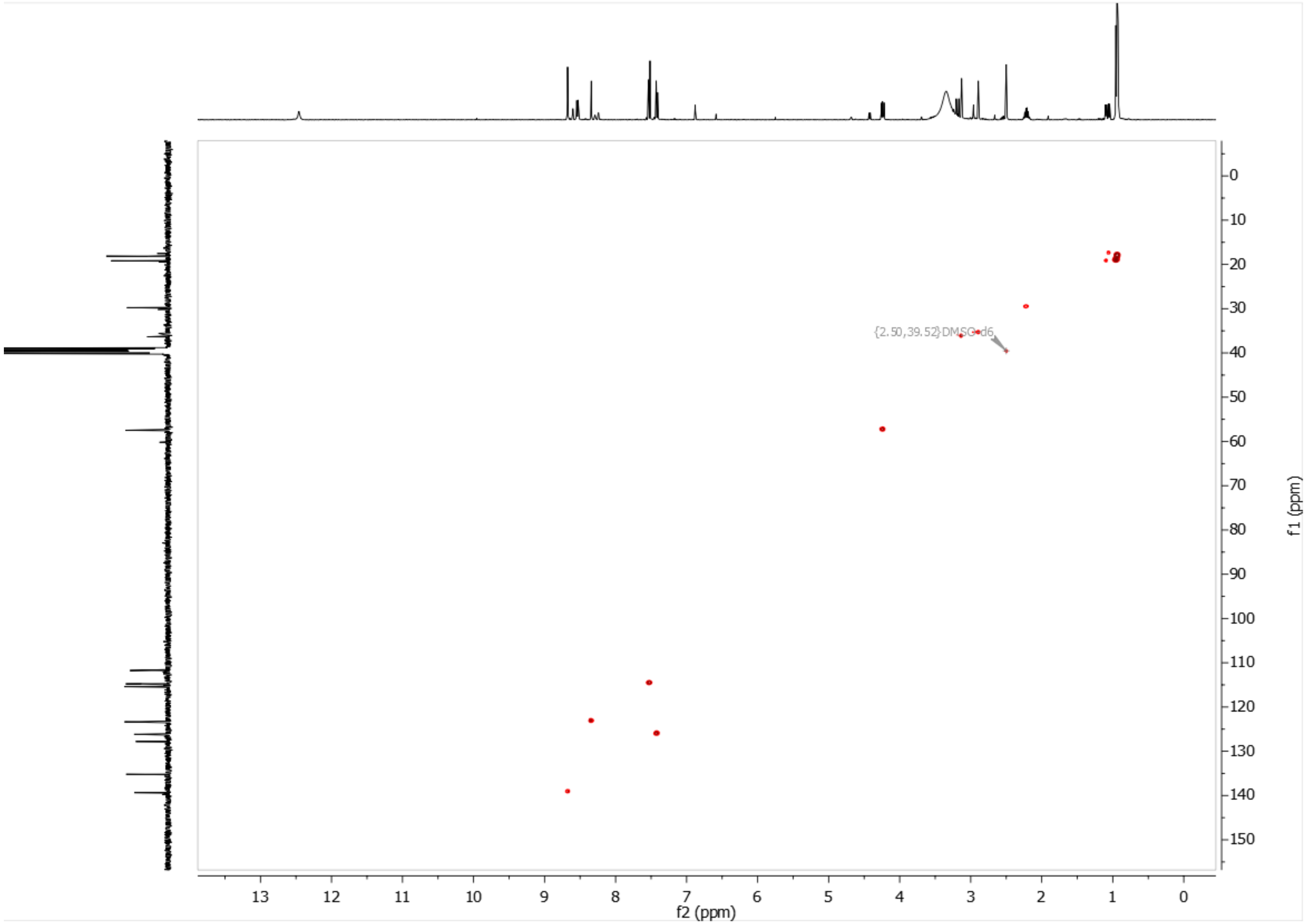

Figure S120. HSQC NMR spectrum for 5-bromoindolyl-3-glyoxyl-L-valine (**53**) recorded in DMSO-d_6

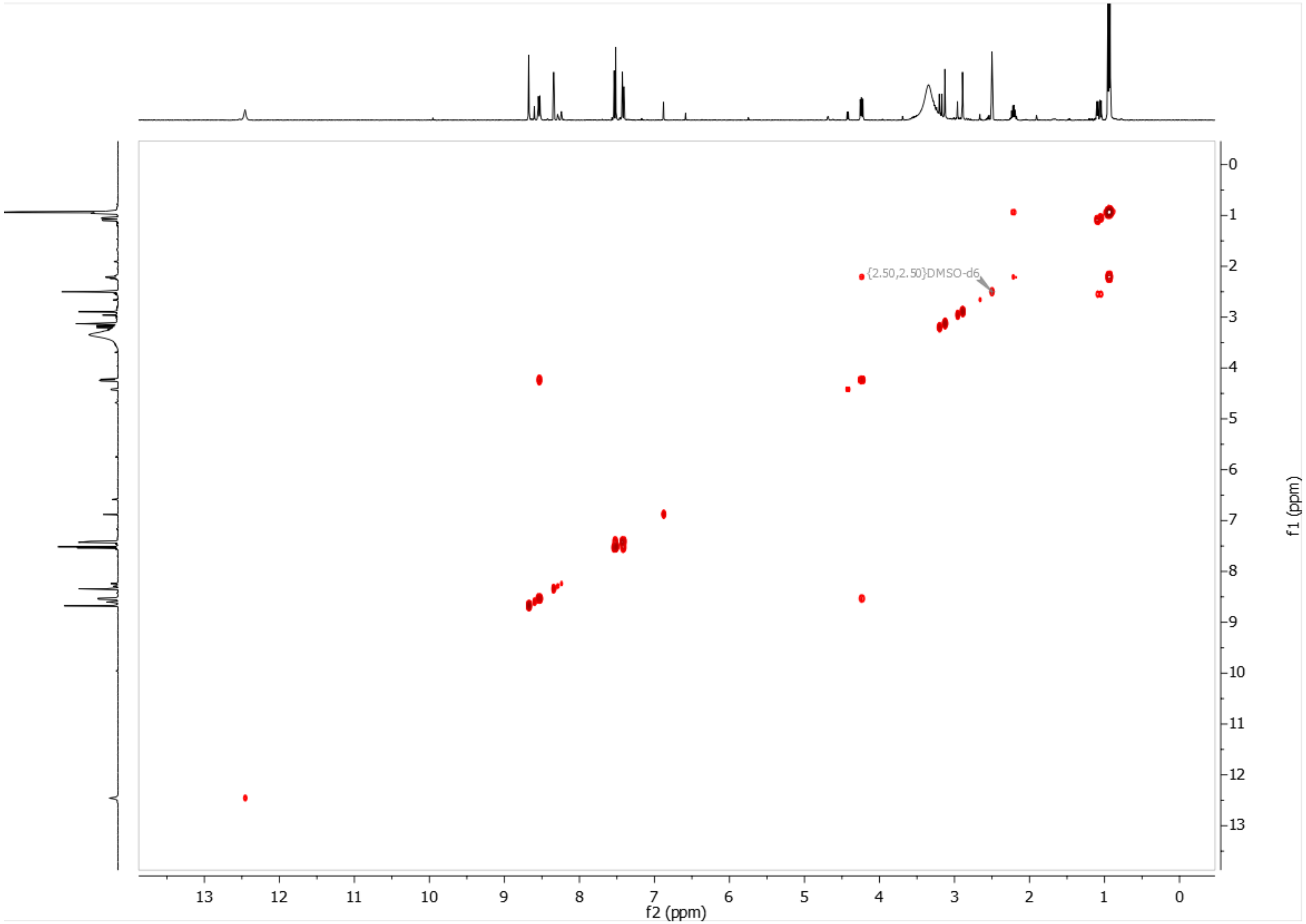

Figure S121. COSY NMR spectrum for 5-bromoindolyl-3-glyoxyl-L-valine (**53**) recorded in DMSO-d_6

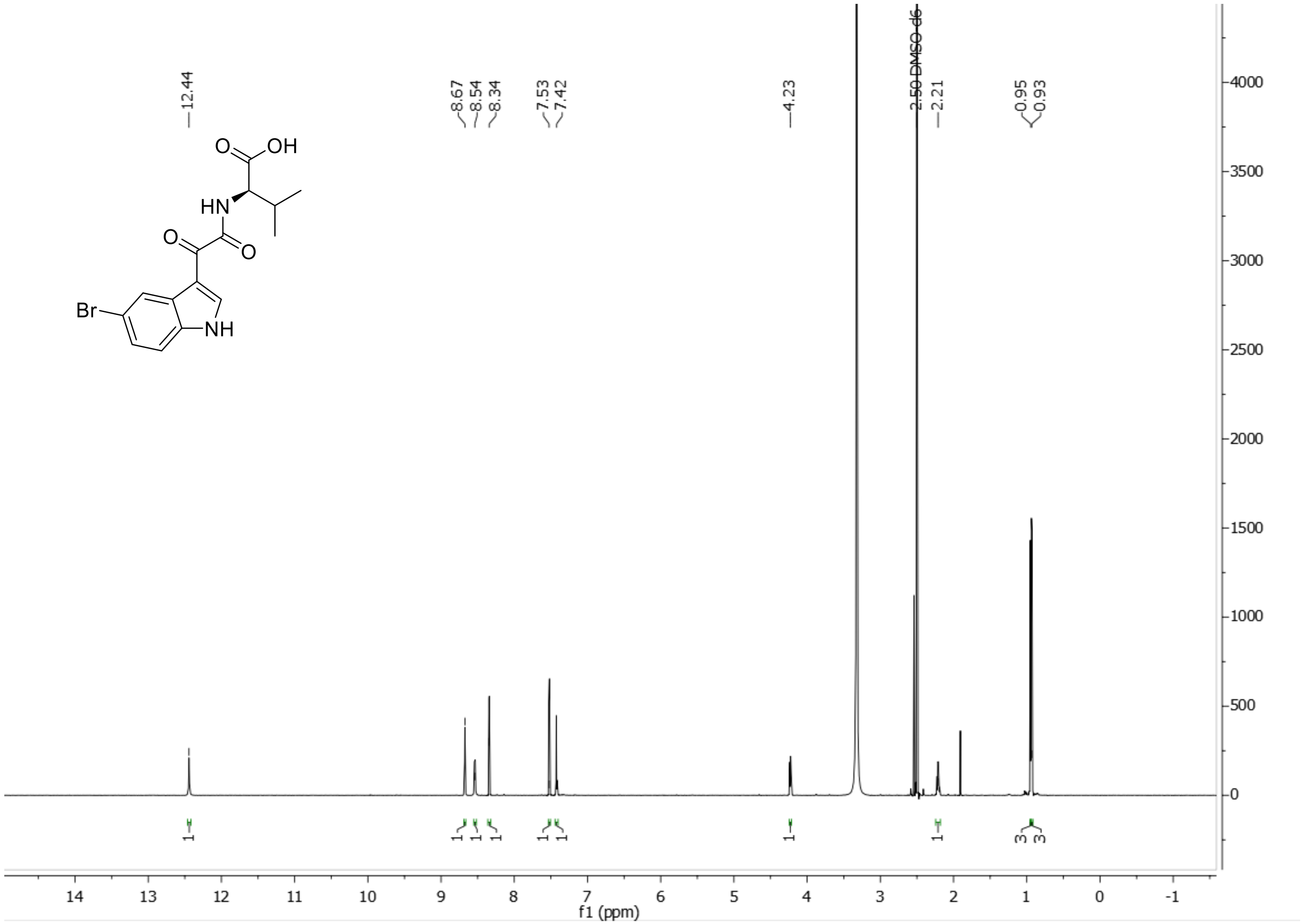

Figure S122. ^{1}H NMR spectrum for 5-bromoindolyl-3-glyoxyl-D-valine (**54**) recorded in DMSO-d_6

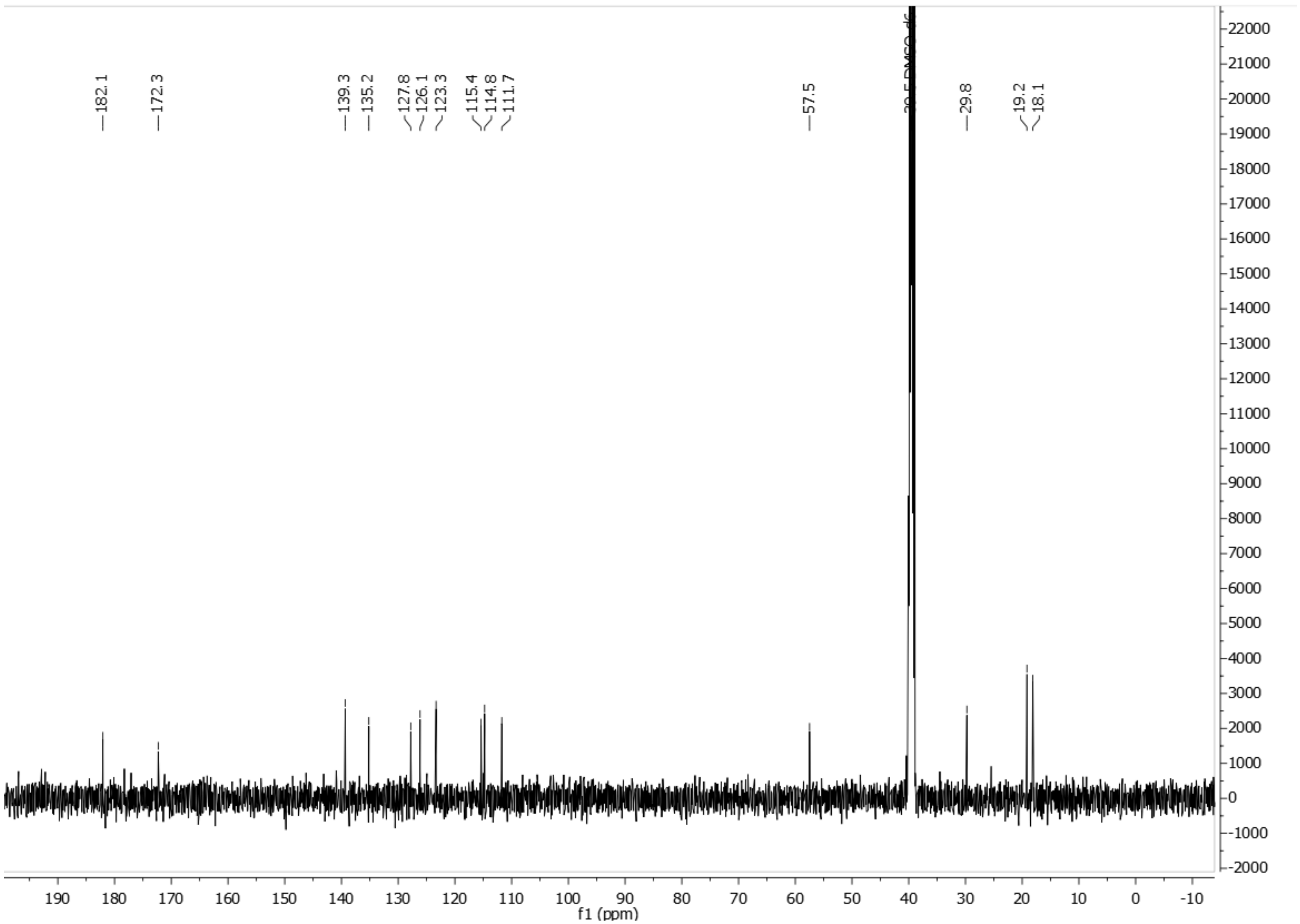

Figure S123. ^{13}C NMR spectrum for 5-bromoindolyl-3-glyoxyl-D-valine (**54**) recorded in DMSO-d_6

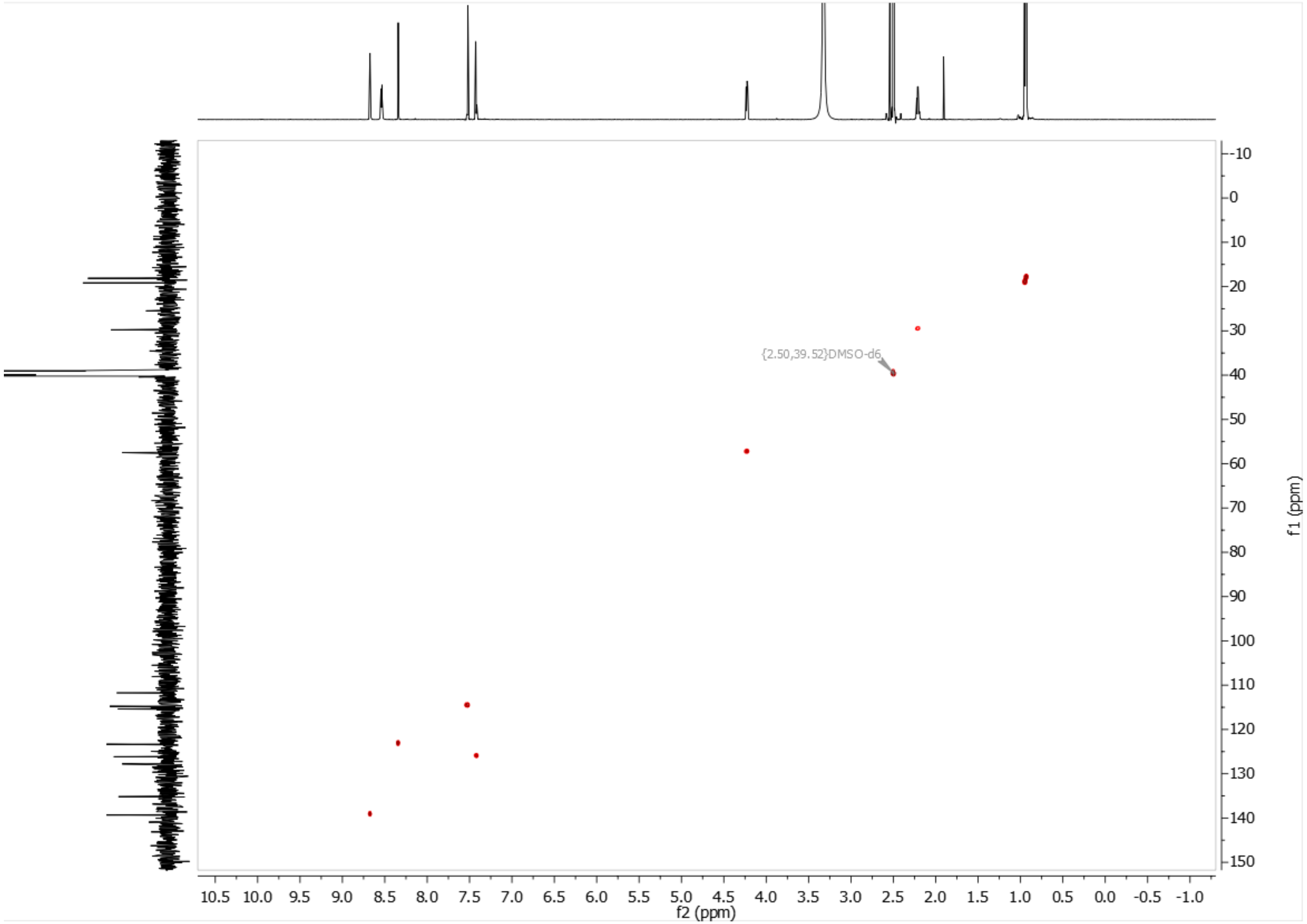

Figure S124. HSQC NMR spectrum for 5-bromoindolyl-3-glyoxyl-D-valine (**54**) recorded in DMSO-d_6

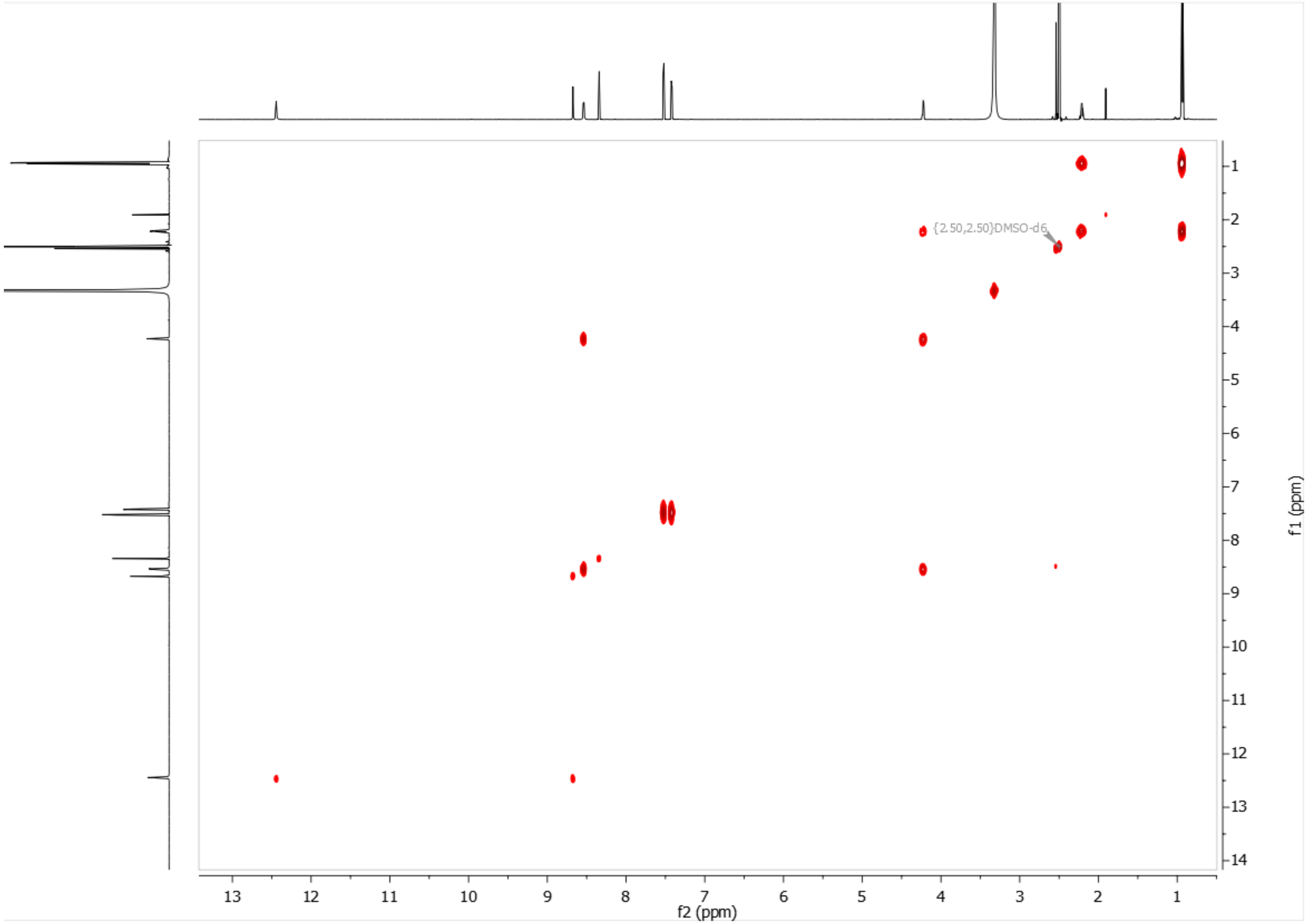

Figure S125. COSY NMR spectrum for 5-bromoindolyl-3-glyoxyl-D-valine (**54**) recorded in DMSO-d_6

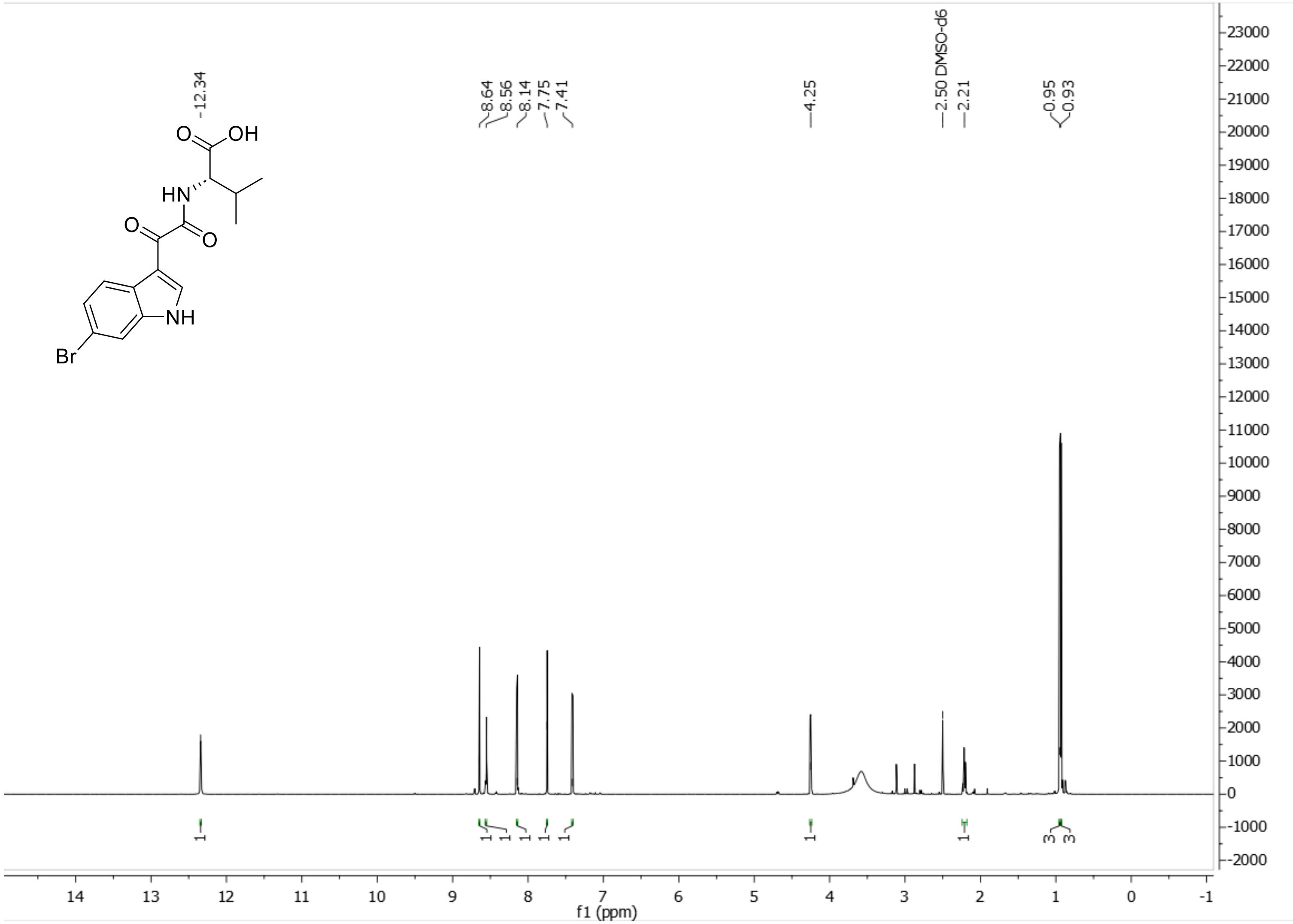

Figure S126. ^{1}H NMR spectrum for 6-bromoindolyl-3-glyoxyl-L-valine (**55**) recorded in DMSO-d_6

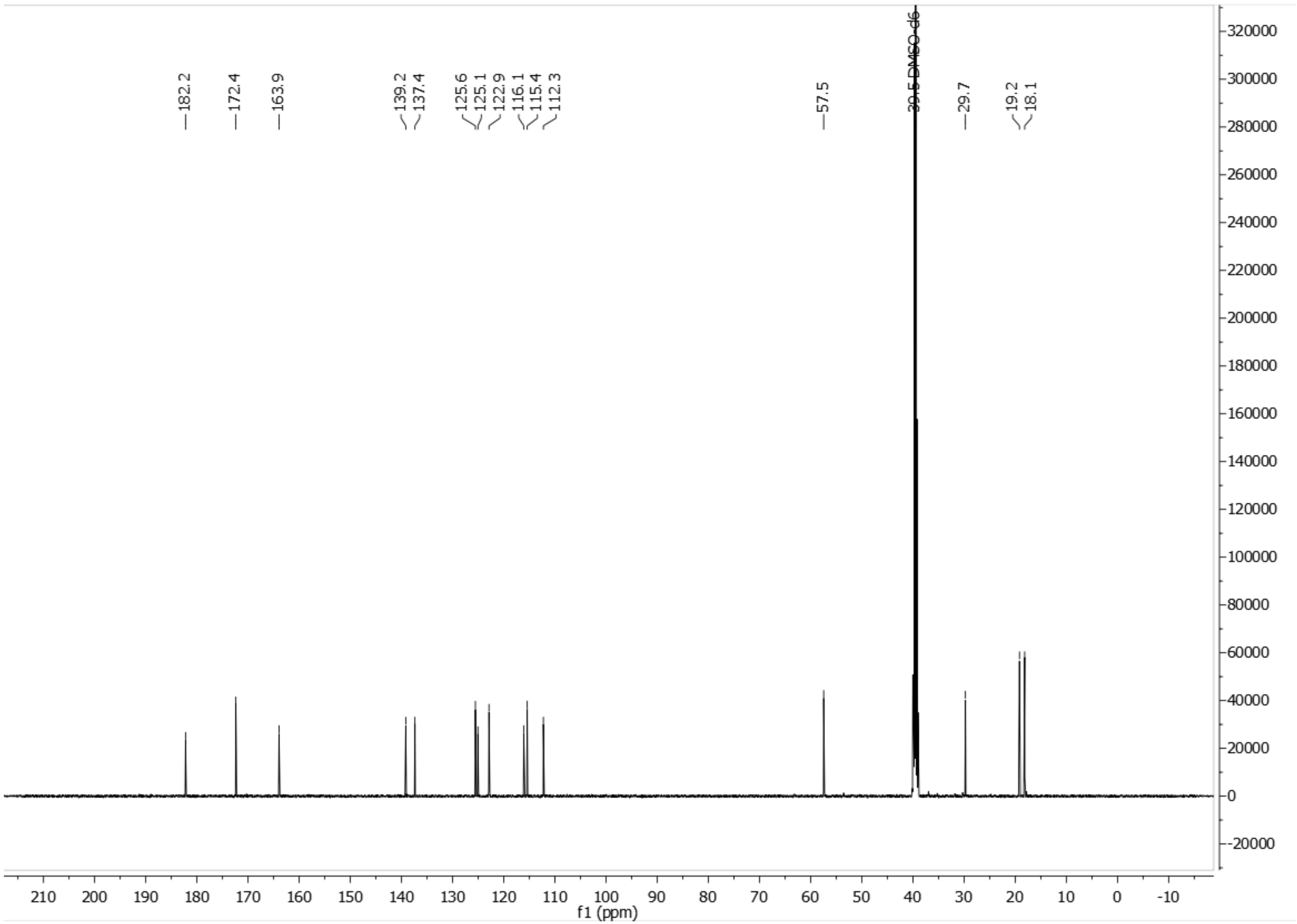

Figure S127. ^{13}C NMR spectrum for 6-bromoindolyl-3-glyoxyl-L-valine (**55**) recorded in DMSO-d_6

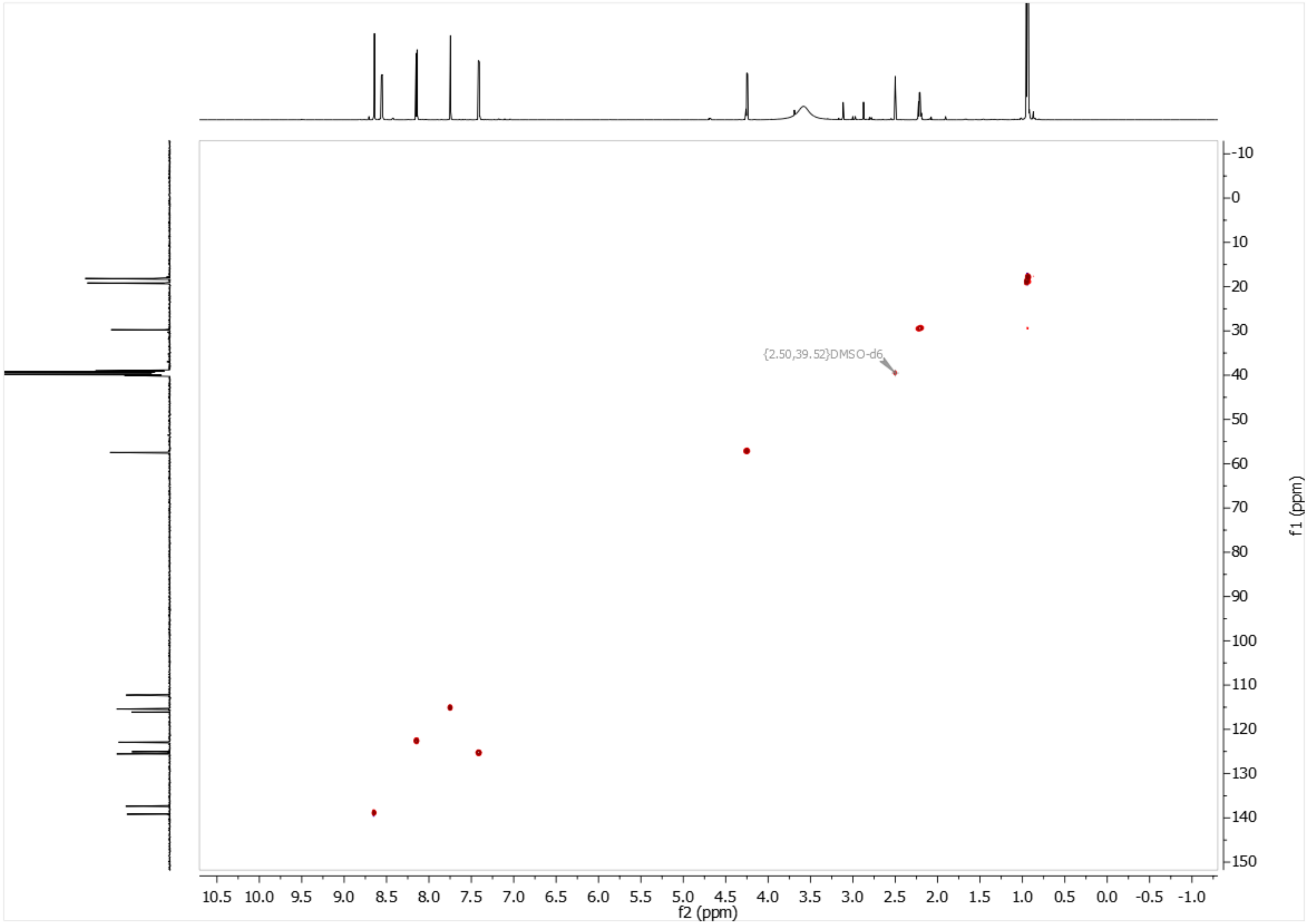

Figure S128. HSQC NMR spectrum for 6-bromoindolyl-3-glyoxyl-L-valine (**55**) recorded in DMSO-d_6

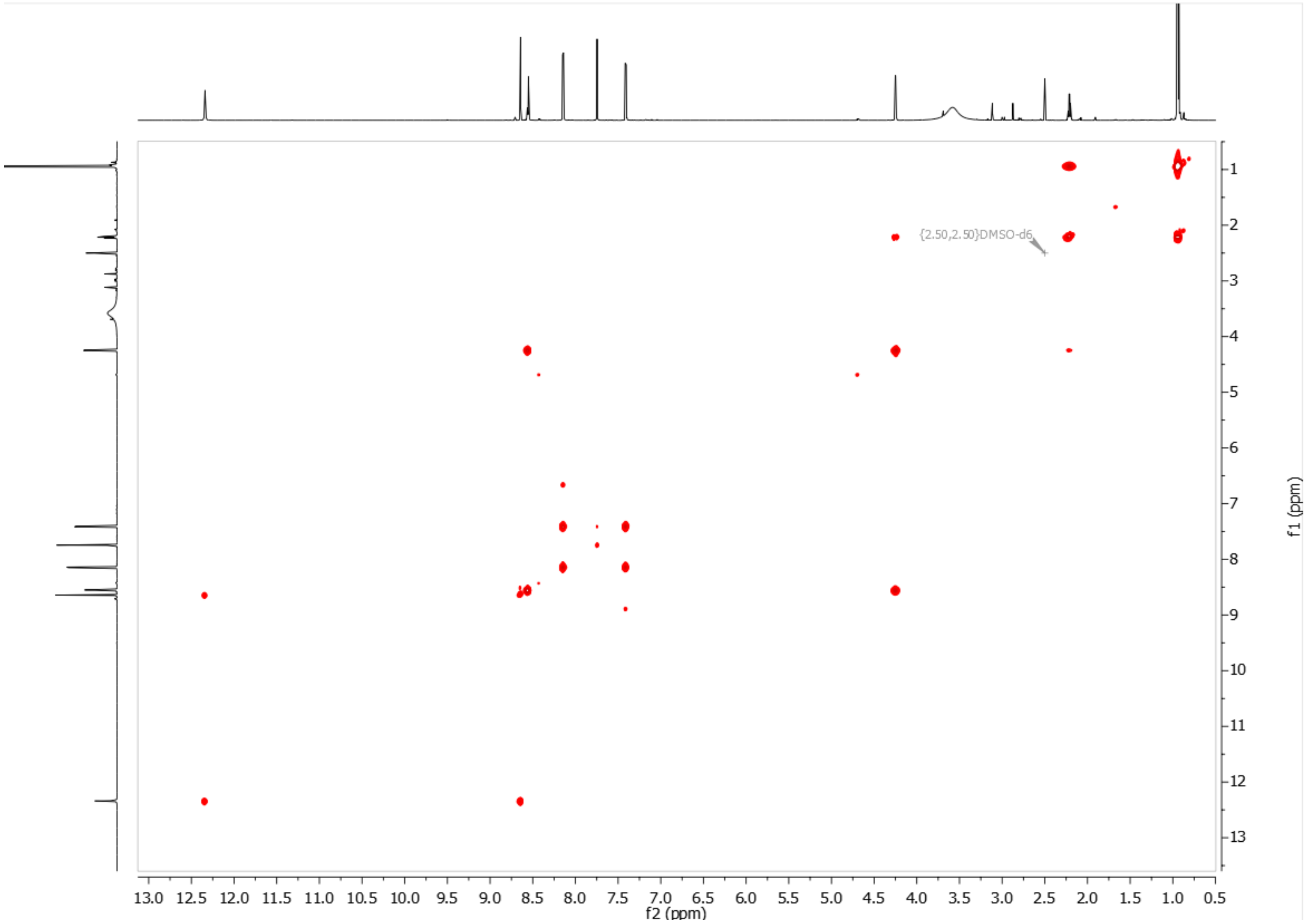

Figure S129. COSY NMR spectrum for 6-bromoindolyl-3-glyoxyl-L-valine (**55**) recorded in DMSO-d_6

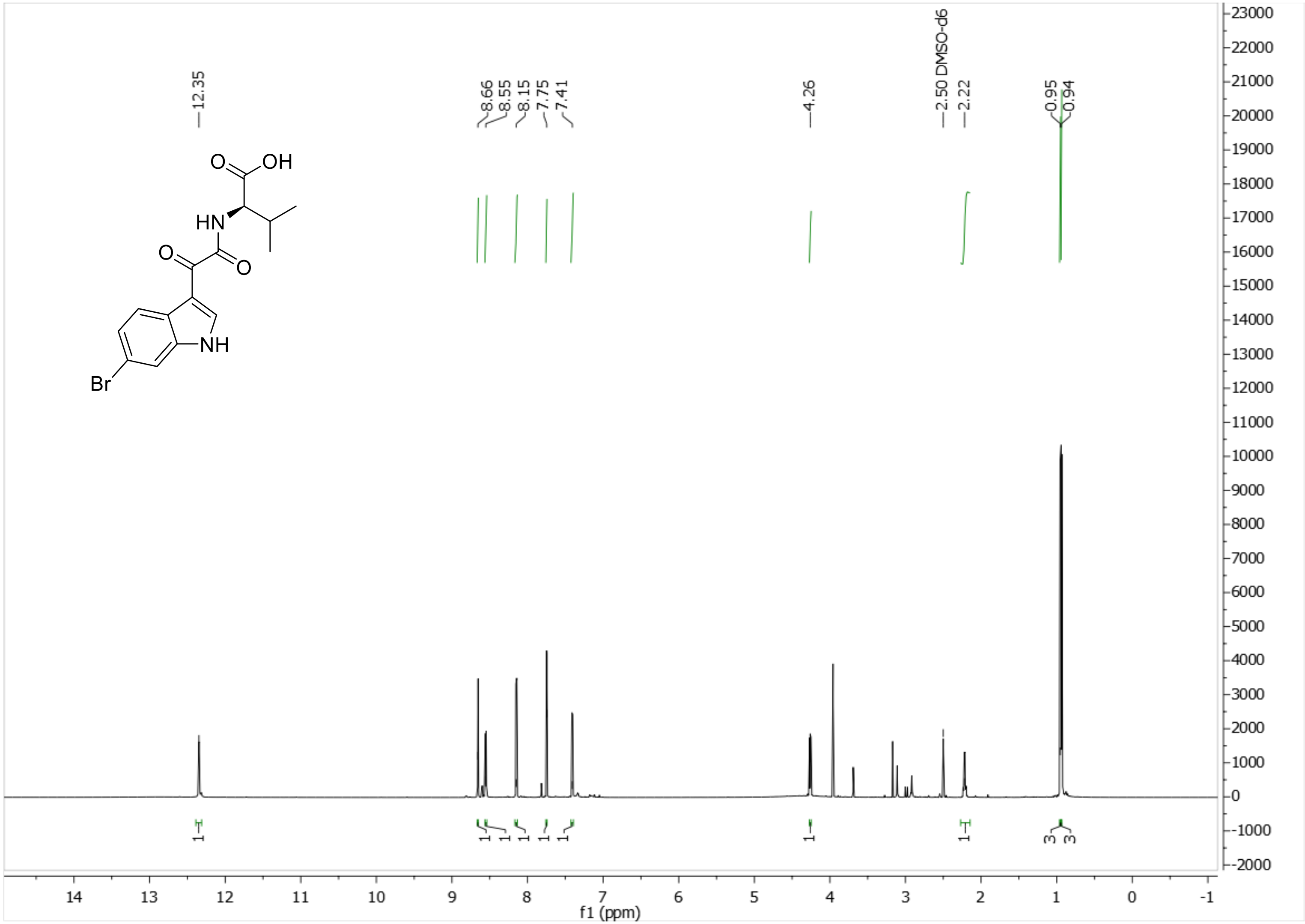

Figure S130. ^{1}H NMR spectrum for 6-bromoindolyl-3-glyoxyl-D-valine (**56**) recorded in DMSO-d_6

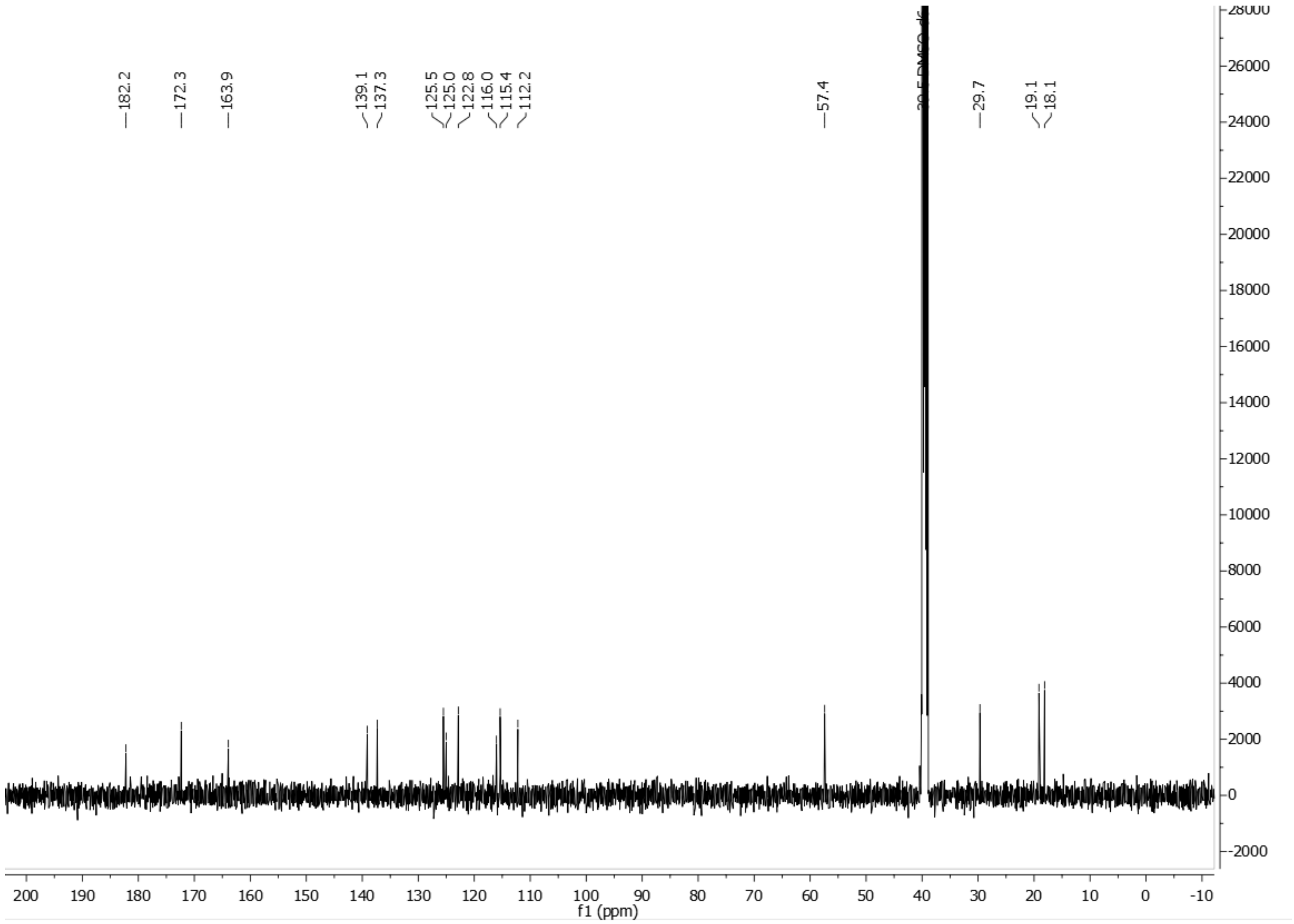

Figure S131. ^{13}C NMR spectrum for 6-bromoindolyl-3-glyoxyl-D-valine (**56**) recorded in DMSO-d_6

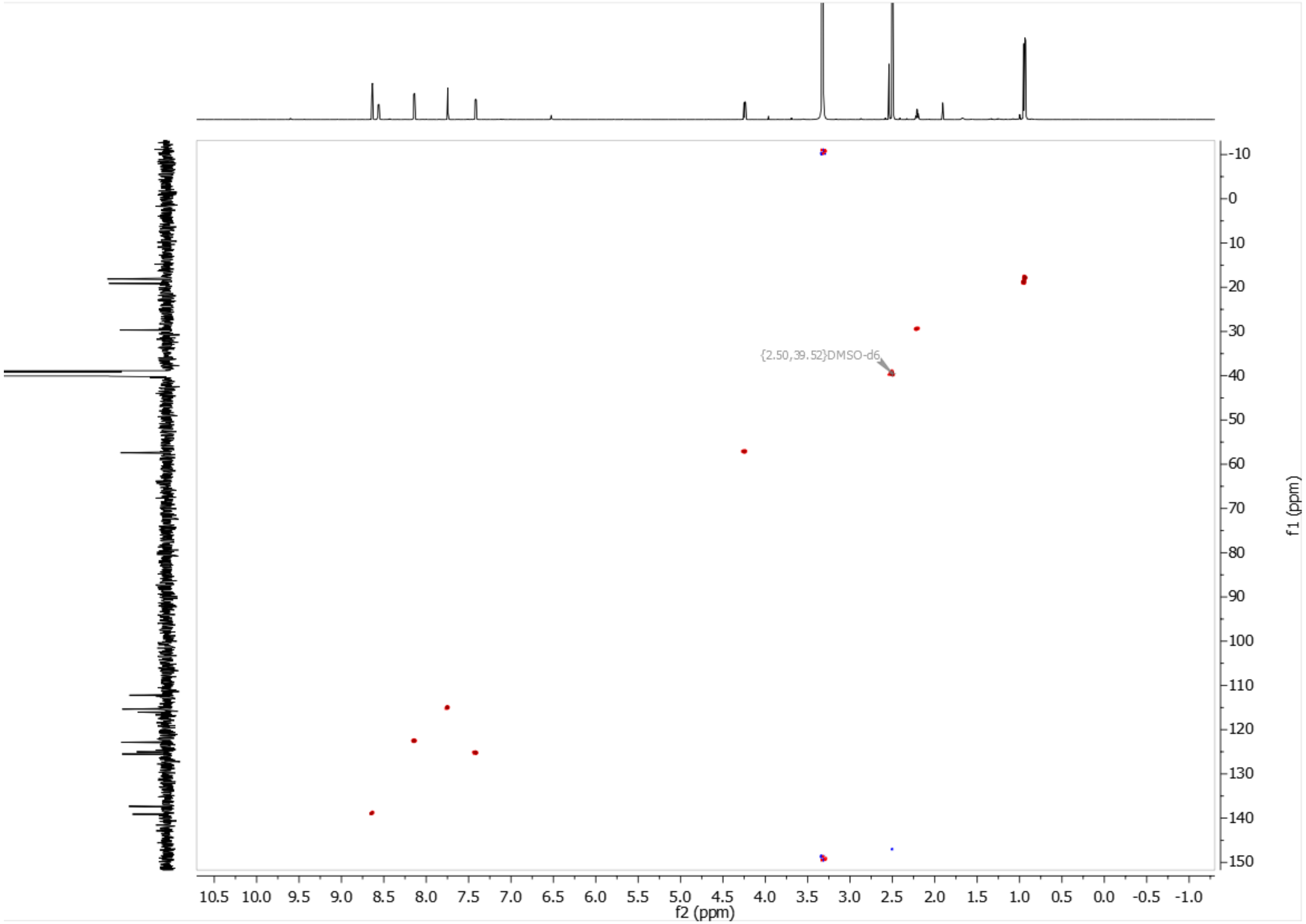

Figure S132. HSQC NMR spectrum for 6-bromoindolyl-3-glyoxyl-D-valine (**56**) recorded in DMSO-d_6

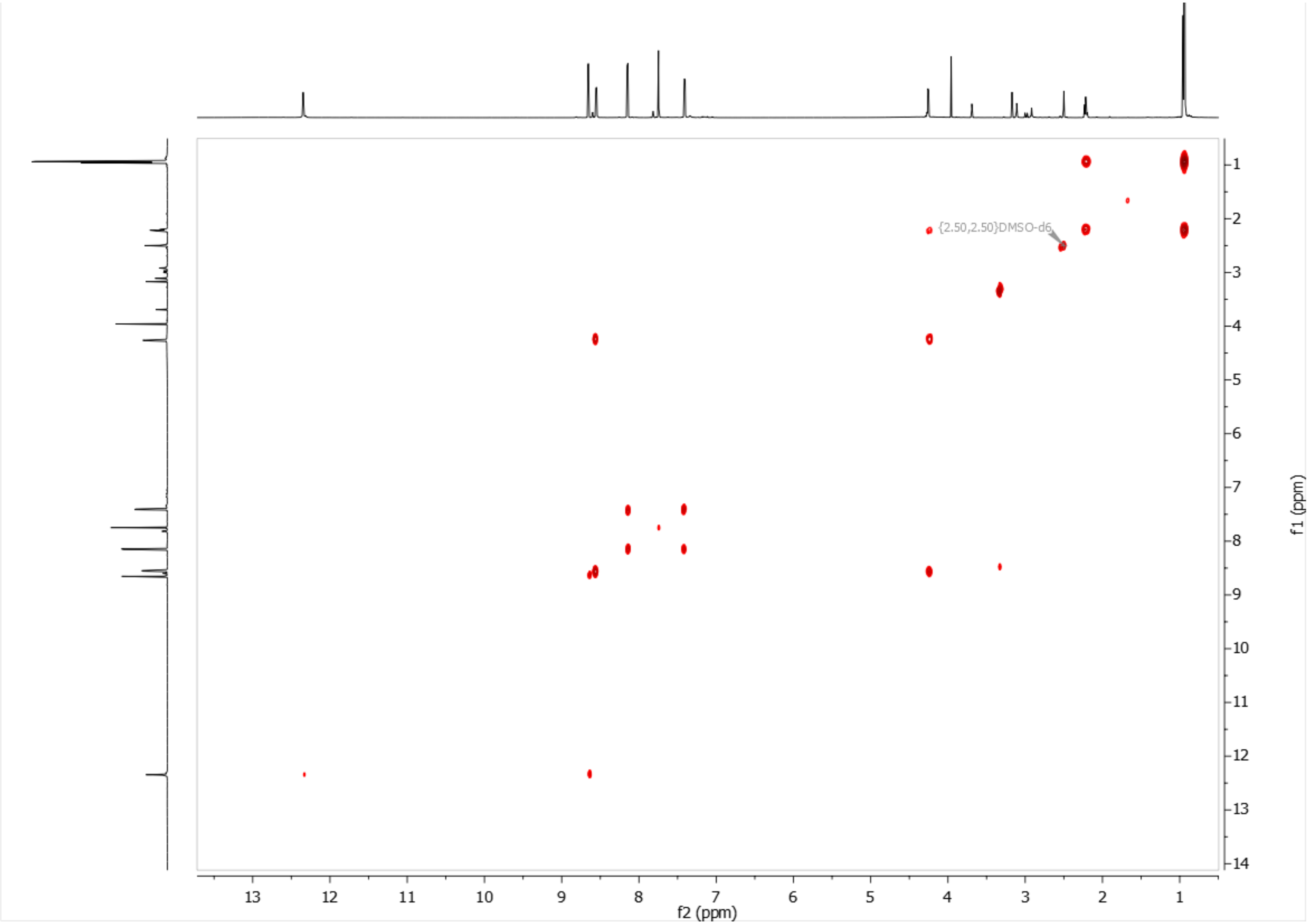

Figure S133. COSY NMR spectrum for 6-bromoindolyl-3-glyoxyl-D-valine (**56**) recorded in DMSO-d_6

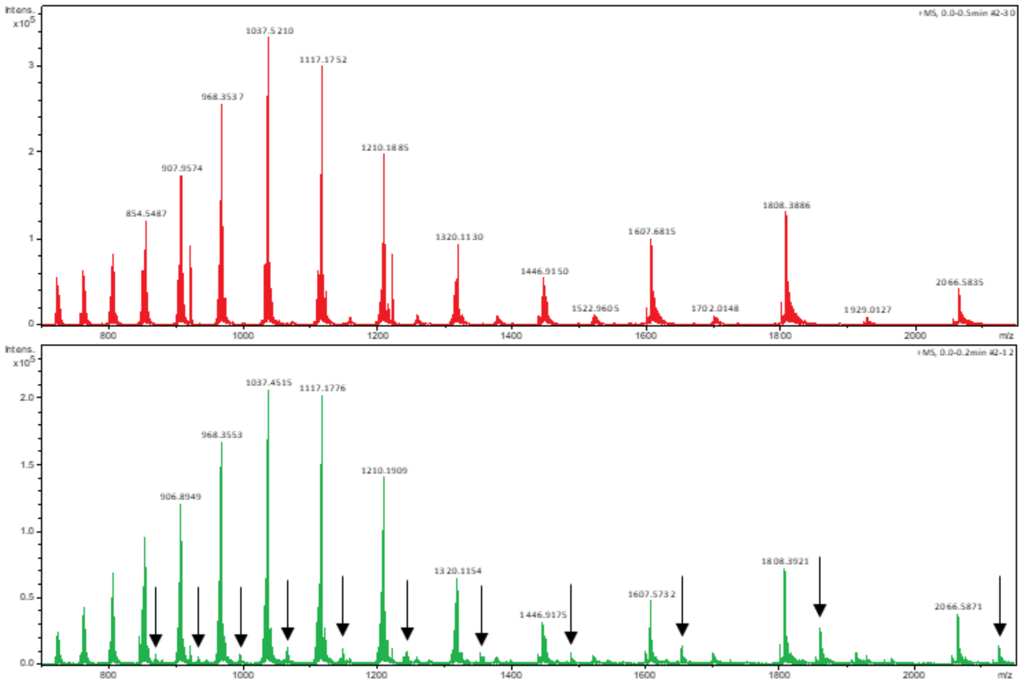

Figure S134. α-synuclein binding assay. (a) Mass spectrum for untreated α-syn, with the peaks marked by their charged state. (b) Mass spectrum for α-syn treated with 6-bromoindolyl-3-glyoxyl-L-arginine (**25**), with arrows indicating the additional peaks representing a 1:1 α-synuclein: 6-bromoindolyl-3-glyoxyl-L-arginine (**25**) complex.

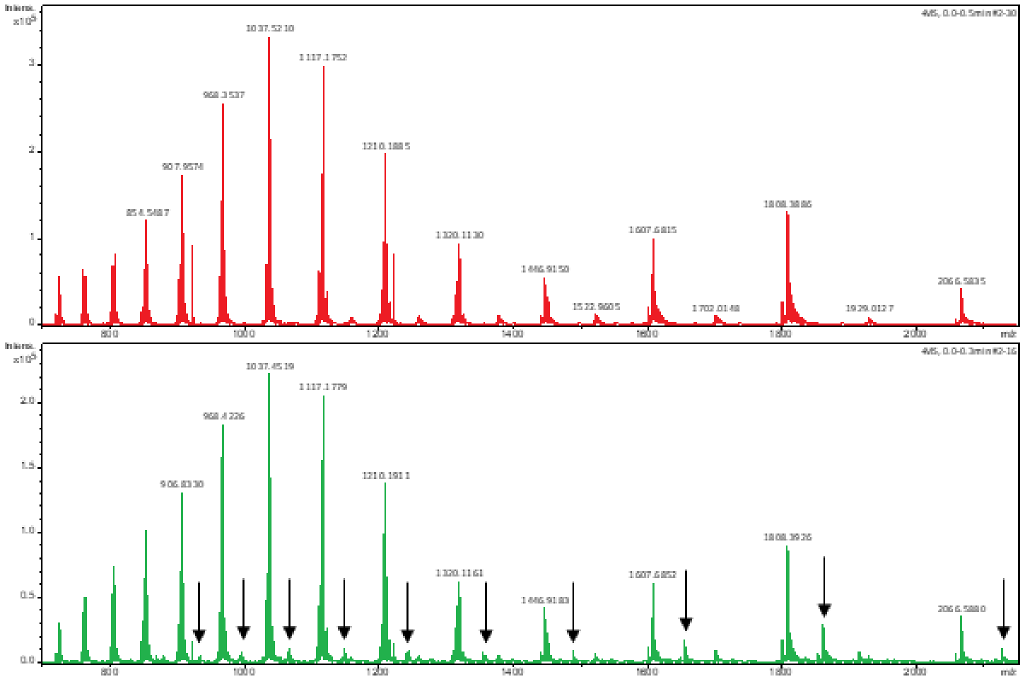

Figure S135. α-synuclein binding assay. (a) Mass spectrum for untreated α-syn, with the peaks marked by their charged state. (b) Mass spectrum for α-syn treated with 6-bromoindolyl-3-glyoxyl-D-arginine (**26**), with arrows indicating the additional peaks representing a 1:1 α-synuclein:6-bromoindolyl-3-glyoxyl-D-arginine (**26**) complex.

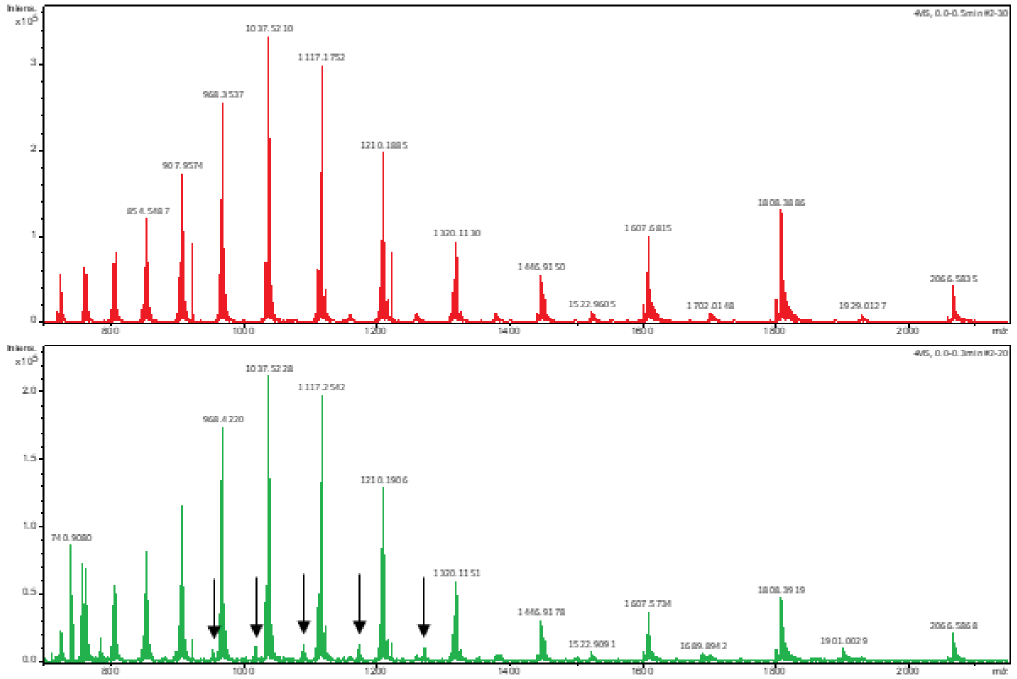

Figure S136. α-synuclein binding assay. (a) Mass spectrum for untreated α-syn, with the peaks marked by their charged state. (b) Mass spectrum for α-syn treated with 6-bromoindolyl-3-glyoxyl-L-cysteine (**34**), with arrows indicating the additional peaks representing a 1:1 α-synuclein:6-bromoindolyl-3-glyoxyl-L-cysteine (**34**) complex.

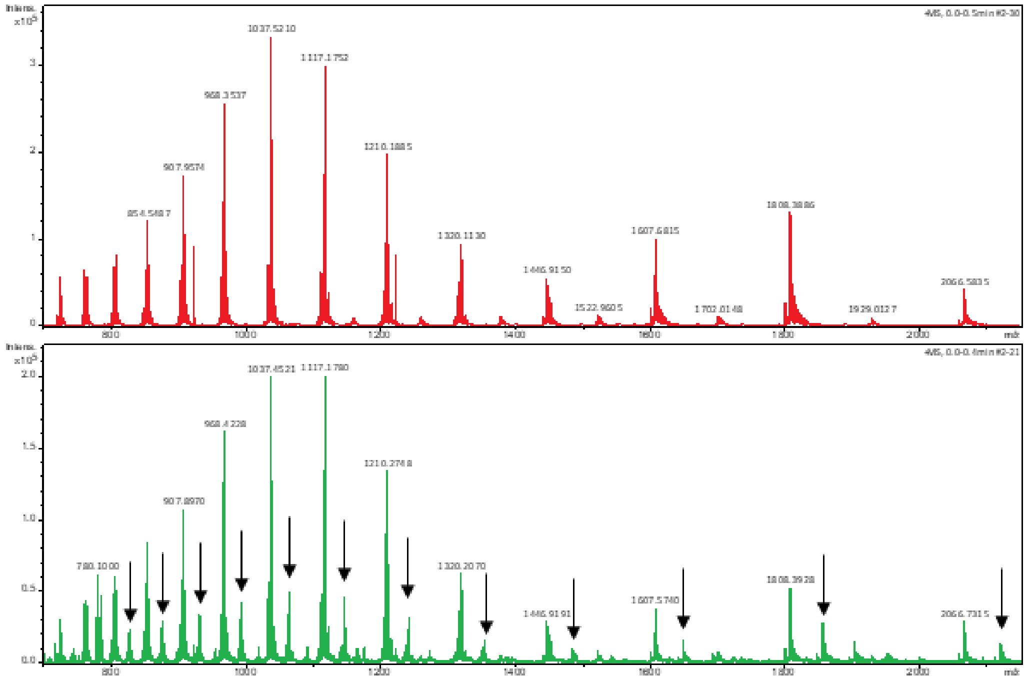

Figure S137. α-synuclein binding assay. (a) Mass spectrum for untreated α-syn, with the peaks marked by their charged state. (b) Mass spectrum for α-syn treated with 5-bromoindolyl-3-glyoxyl-L-isoleucine (**41**), with arrows indicating the additional peaks representing a 1:1 α-synuclein:5-bromoindolyl-3-glyoxyl-L-isoleucinecysteine (**41**) complex.

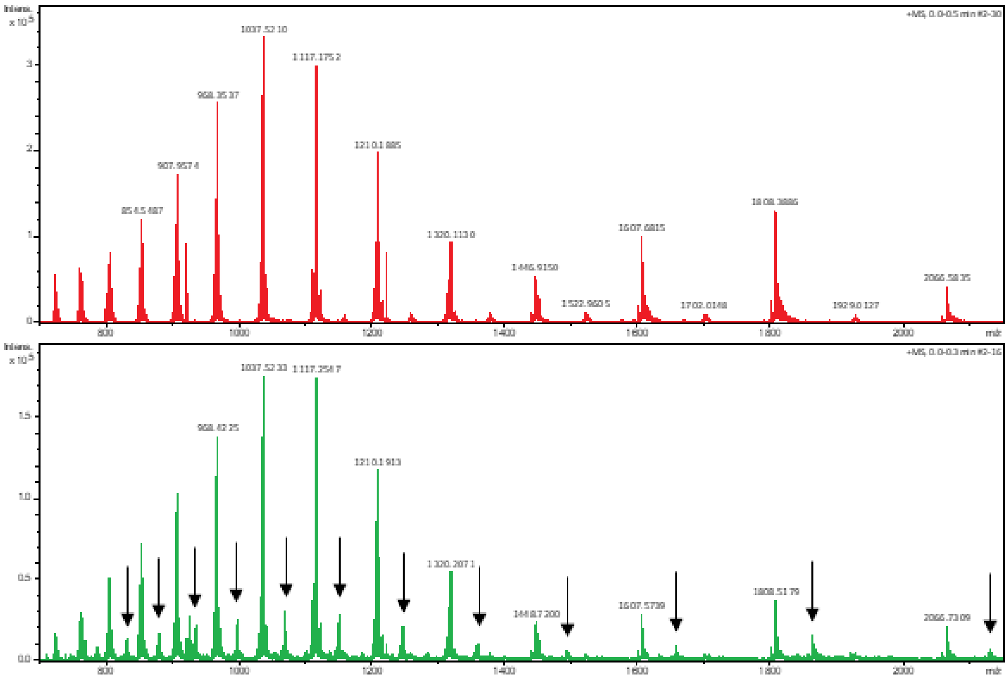

Figure S138. α-synuclein binding assay. (a) Mass spectrum for untreated α-syn, with the peaks marked by their charged state. (b) Mass spectrum for α-syn treated with 5-bromoindolyl-3-glyoxyl-L-tryptophan (**45**), with arrows indicating the additional peaks representing a 1:1 α-synuclein:5-bromoindolyl-3-glyoxyl-L-tryptophan (**45**) complex.

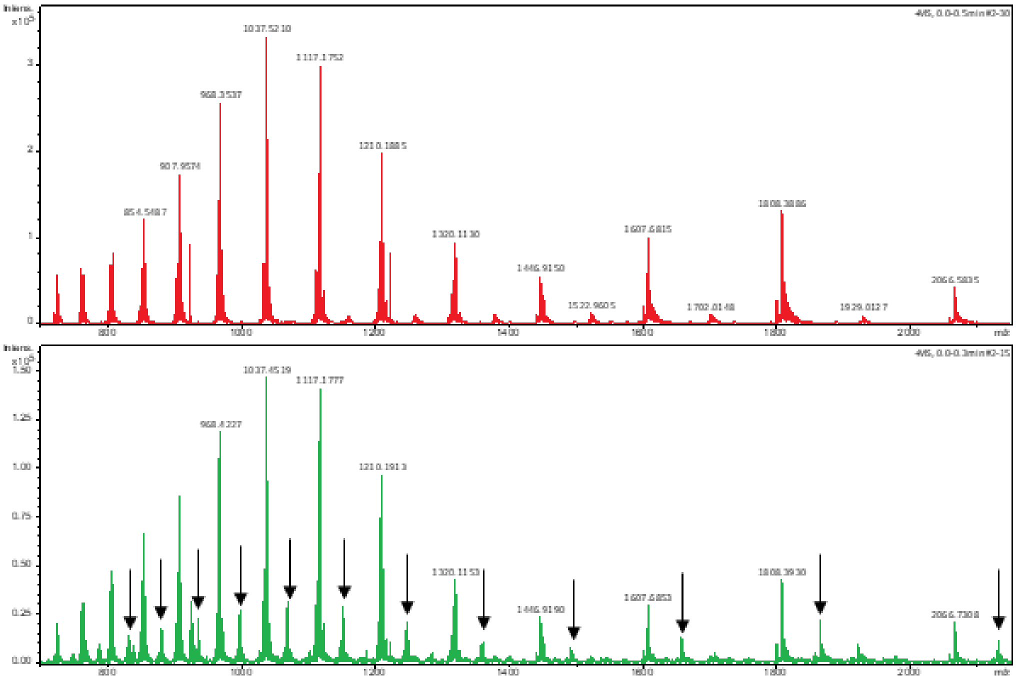

Figure S139. α-synuclein binding assay. (a) Mass spectrum for untreated α-syn, with the peaks marked by their charged state. (b) Mass spectrum for α-syn treated with 5-bromoindolyl-3-glyoxyl-D-tryptophan (**46**), with arrows indicating the additional peaks representing a 1:1 α-synuclein:5-bromoindolyl-3-glyoxyl-D-tryptophan (**46**) complex.

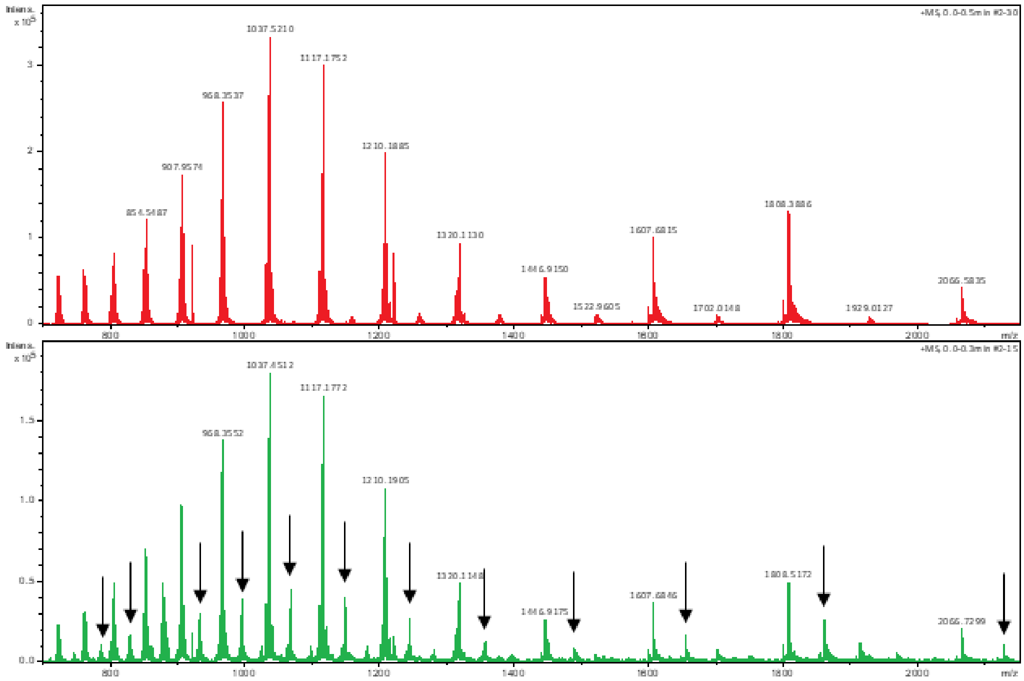

Figure S140. α-synuclein binding assay. (a) Mass spectrum for untreated α-syn, with the peaks marked by their charged state. (b) Mass spectrum for α-syn treated with 6-bromoindolyl-3-glyoxyl-L-tyrosine (**51**), with arrows indicating the additional peaks representing a 1:1 α-synuclein:6-bromoindolyl-3-glyoxyl-L-tyrosine (**51**) complex.

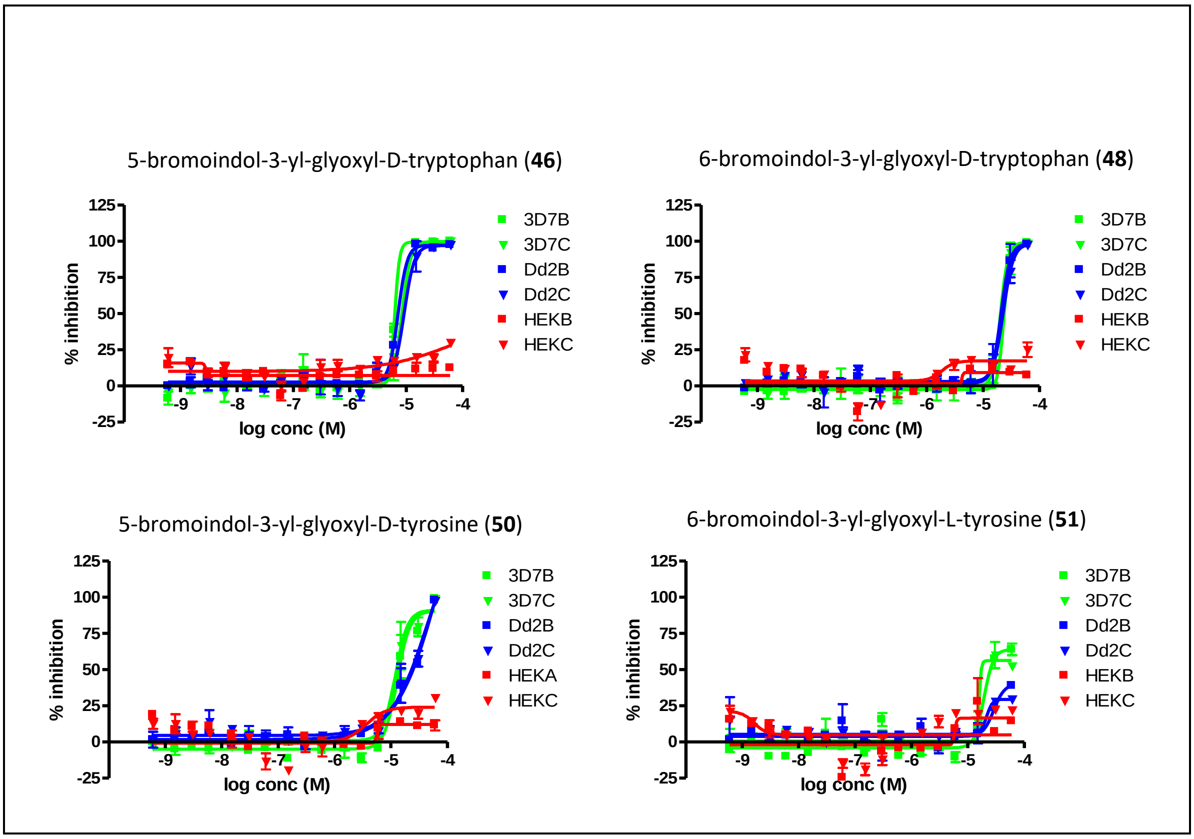

Figure S141. Dose-response curves for synthetic indoles **46**, **48**, **50**, and **51** against chloroquine-sensitive (3D7) and -resistant (Dd2) *Plasmodium falciparum* parasite strains and a human embryonic cell line (HEK293).

2. References

(1) MarinLit http://pubs.rsc.org/marinlit/ (accessed May 11, 2024).

(2) Holland, D. C.; Carroll, A. R. Marine Indole Alkaloid Diversity and Bioactivity. What Do We Know and What Are We Missing? *Nat. Prod. Rep.* **2023**. https://doi.org/10.1039/d2np00085g.
